# Anion‐Dependent Hydrogen‐Bond Polarity Switching in Ethylene‐bridged Urea Oligomers

**DOI:** 10.1002/chem.202302210

**Published:** 2023-09-29

**Authors:** David P. Tilly, David T. J. Morris, Jonathan Clayden

**Affiliations:** ^1^ School of Chemistry University of Bristol Cantock's Close Bristol BS8 1TS UK; ^2^ Department of Chemistry University of Manchester Oxford Road Manchester M13 9PL UK

**Keywords:** dynamic foldamer, allostery, urea, conformation, hydrogen bond

## Abstract

The reversible coordination of anions to an *N*,*N’*‐disubstituted 3,5‐bis(trifluoromethyl)phenylurea located at a terminus of a linear chain of ethylene‐bridged hydrogen‐bonded ureas triggers a cascade of conformational changes. A series of hydrogen‐bond polarity reversals propagates along the oligomer, leading to a global switch of its hydrogen‐bond directionality. The induced polarity switch, transmitted through four reversible urea groups, results in a change in emission and excitation wavelengths of a fluorophore located at the opposite terminus of the oligomer. The molecule thus behaves as a chemical sensor with a relayed remote spectroscopic response to variations in anion concentration. The polarity switch induced by anion concentration constitutes an artificial communication mechanism for conveying information through oligomeric structures.

## Graphical abstract

**The reversible coordination** of various anions to a binding site at one terminus of a urea oligomer leads to a global switch in polarity of a linear chain of hydrogen‐bonded ureas. The fluorescence response of a hydrogen‐bond‐responsive dithiomaleimide fluorophore at the opposite terminus reveals a relayed change in local hydrogen bonding directionality.

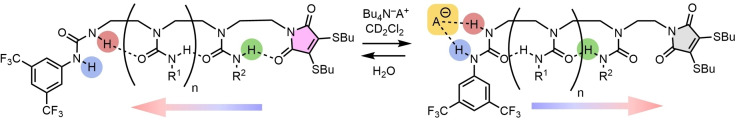

## Introduction

**Figure 1:**
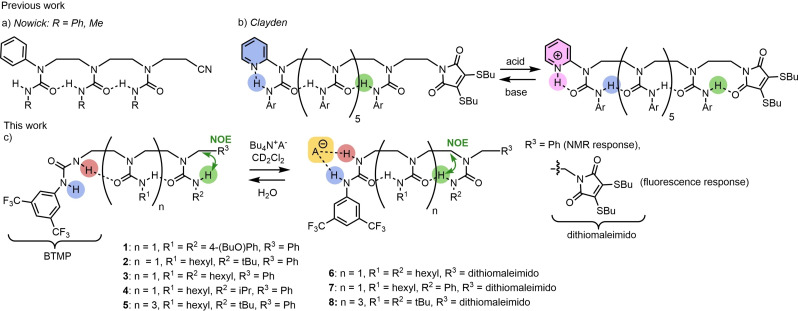
Hydrogen bond polarity in ethylene‐bridged oligoureas. a) Chemical models for protein β‐sheets (Nowick). b) Molecular communication channel with pH‐sensitive polarity control. c) Molecular communication channel with anion‐sensitive polarity control.

Induced conformational changes are ubiquitous biological signal transduction mechanisms.^[1]^ Allosteric enzymes,^[2]^ such as G‐protein‐coupled receptors,^[3]^ photoreceptor proteins,^[4]^ hemoglobin,^[5]^ and phosphorylases^[6]^ all use conformational signaling to regulate their functions. Dynamic foldamers^[7]^ are synthetic oligomeric structures that similarly undergo well defined conformational changes in response to specific stimuli^[8]^ such as solvent polarity,^[9]^ ligand,^[10]^ light^[11]^ and pH.^[12]^ To date, most dynamic foldamers have conveyed information through space using stereochemical signals, such as a helical screw‐sense preference.^[13]^ We recently described the switchable polarity of a linear chain of hydrogen‐bonded ureas as an intramolecular communication channel.^[14]^ The regular spatial recurrence of identical carbamoyl substituents along an oligoethylenediamine backbone programs the oligomers to form a coherent intramolecular linear chain of hydrogen bonds running the full length of the structure in non‐polar solvents,^[15]^ with each of the carbamoyl substituents forming 9‐membered intramolecular hydrogen‐bonded rings with adjacent carbamoyl substituents.^[16]^ The dynamic conformation of each individual carbamoyl substituent is influenced by the conformations of the adjacent carbamoyl groups, so that the opportunity for hydrogen bonding is maximised. In a constitutionally symmetrical system the hydrogen‐bond chain exists as two equally populated, degenerate conformers in dynamic equilibrium, with each terminus fluctuating between hydrogen‐bond donor (NH, or N terminus) and acceptor (C=O, or C terminus) status. A preferred hydrogen‐bond polarity can be induced by structural alterations at one of the termini. Thus a phenyl^[17]^ or pyridyl[^14a^, ^14b^] group at one terminus induces (Figure 1a and b) a conformational preference in the adjacent carbamoyl group that is relayed along the chain. We showed that the global hydrogen‐bond polarity of the chain may be switched reversibly by pH stimuli or the presence of acetate, a strong hydrogen‐bond acceptor. A hydrogen‐bond donating thiourea may also serve as a hydrogen‐bond polarity inducer: a thiourea terminus was used in a pH‐sensitive device for the catch‐and‐release of a phosphine oxide ligand.^[14b]^

Here we report in full the design, synthesis and conformational analysis of ethylene‐bridged urea oligomers bearing a terminal *N*,*N*’‐disubstituted urea that functions both as a global polarity controller and as binding site for anions. The regioselective binding of anionic ligands (acetate, chloride, and phosphate) is characterised by ^1^H NMR spectroscopy for short and then longer urea oligomers. The formation of an anion‐foldamer complex is shown by DOSY NMR spectroscopy and by mass spectrometry. The induced polarity switch in the urea oligomers is characterised by ^1^H NMR spectroscopy, by NOE analyses, and by a modulation of the fluorescence properties of a remote fluorophore.

## Results and Discussion

### Establishing a native global hydrogen‐bond polarity preference

**Figure 2:**
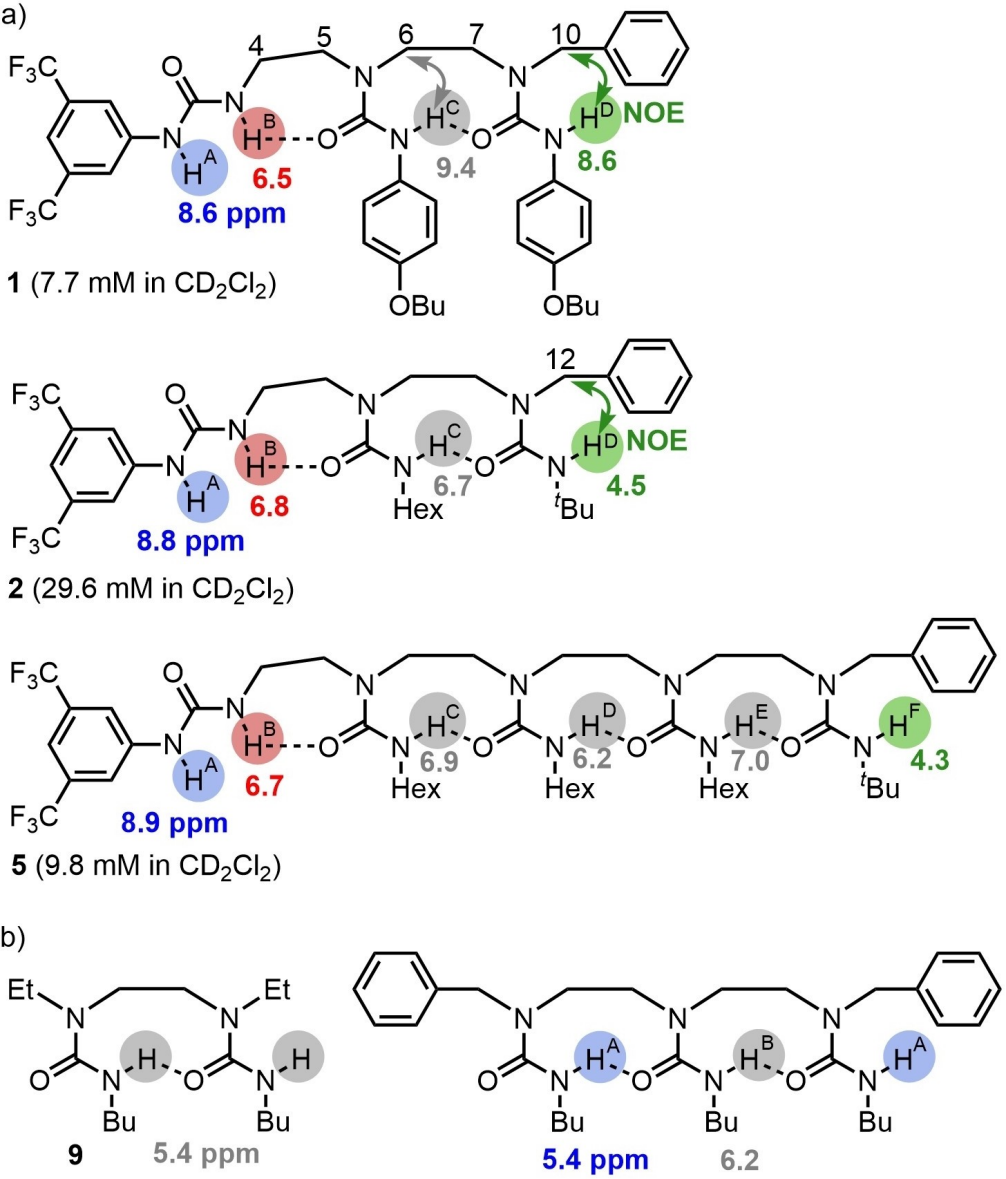
Urea oligomers synthesized. a) Conformationally diagnostic ^1^H NMR chemical shifts of NH groups and NOEs in CD_2_Cl_2_ at 25 °C and b) control compounds synthesised.

We began by synthesizing a series of ethylene‐bridged urea oligomers bearing either *N*‐aryl or *N*‐alkyl carbamoyl substituents, incorporating at one terminus of the chain a disubstituted 3,5‐bis(trifluoromethyl)phenylurea (BTMP urea, Figure 2a, Schemes S1‐S3) as a strong hydrogen bond donor.^[18]^ We assessed the population of conformers in CD_2_Cl_2_ by ^1^H NMR spectroscopy to characterize the polarity‐inducing influence of the BTMP urea on the hydrogen‐bonded chain. At low concentrations, the ^1^H NMR chemical shift values of ureido NHs are diagnostic of their hydrogen‐bonded states, and the terminal benzyl substituent remote from the BTMP urea allowed NOESY to reveal the spatial arrangement between the terminal carbamoyl NH and the benzylic methylene group. For all the compounds tested (Figure 2), ^1^H NMR spectra consistently showed a single well‐defined conformer, irrespective of the urea substituents (see in detail in supporting information part 5).

A BTMP urea is a stronger hydrogen‐bond donor and weaker hydrogen‐bond acceptor than either the 4‐butoxyphenyl or alkyl ureas that constitute the oligourea chain. This difference in hydrogen‐bonding capabilities translated into a unidirectional hydrogen bond chain in **1** (R^1^=R^2^=4‐BuOC_6_H_4_, Figures S1–S8), **2** (R^1^=hexyl, R^2^=^*t*^Bu, Figures S9–S16) and its analogues **3**, (R^1^=R^2^=hexyl, Figures S24–S27), **4 (**R^1^=hexyl, R^2^=^*I*^Pr, Figures S28–S32). In **1** the ^1^H NMR chemical shifts values of the internal ureido NH in CD_2_Cl_2_ at 25 °C indicate that they are engaged in hydrogen bonding (Figure 2a). The ^1^H NMR chemical shift values of ureido NH^B^ (6.5 ppm) and NH^A^ (8.6 ppm) in CD_2_Cl_2_ at 25 °C (7.71 mM) are significantly downfield from the values reported for the non‐hydrogen‐bonded ureido NH in *N*‐3,5‐di(trifluoromethyl)phenyl‐*N*’‐butylurea urea (4.67 ppm, 6.55 ppm, Figure S5).^[19]^ The ^1^H NMR chemical shift value of ureido NH^C^ (9.4 ppm) is downfield from known values for non‐hydrogen‐bonded ureido NHs of branched *p*‐methoxyphenylureas.^[14a]^ Also, NOESY experiments (30 mM in CD_2_Cl_2_) show correlation between NH^D^ and benzylic methylene signal H^10^ but no correlation with either H^6^ or H^7^. Correlation between NH^C^ and both H^6^ and H^7^ is evident, but not between NH^C^ and either H^5^ or H^4^ (Figure S4). A correlation between NH^B^ and CH^10^ is also observed, which suggests some head‐to‐tail self‐association at that concentration. Dilution of **1** in CD_2_Cl_2_ (30 mM to 1.5 mM) shifted all ureido NH signals upfield, also indicative of some degree of self‐association at higher concentration. VT ^1^H NMR of **1** between 25 °C and −80 °C in CD_2_Cl_2_ showed a downfield shift for all the ureido NH signals upon cooling, without signal decoalescence (Figures S7 and S8). Overall, these data are consistent with the preferred hydrogen‐bond polarity being as shown in Figure 2, with the BTMP urea located at the C terminus of the hydrogen‐bonded chain of ureas.

For triurea **2**, the ^1^H NMR chemical shift values of the internal ureido NH indicate their hydrogen bonding states (Figure 2a). ^1^H NMR spectra of **2** recorded in CD_2_Cl_2_ between 5 °C and 35 °C clearly show a non‐hydrogen‐bonded NH^D^ (4.5 ppm, Figure S12). NOESY at 30 °C shows correlation between NH^D^ and H^12^, indicating the directionality of the hydrogen bond chain (Figure S13). ^1^H‐^15^N HSQC experiment confirmed the position of the NH signals. Only one conformer is evident in CD_2_Cl_2_ at ambient temperature. Based on the ^1^H NMR chemical shift values of the ureido NH signals, the hydrogen‐bond directionality of **2** was identical in CD_2_Cl_2_, CDCl_3_, and benzene. The conformational preferences of **3** (R^2^=Bn, R^3^=^*n*^Bu) and **4** (R^2^=Bn, R^3^=^*i*^Pr) matched those of **2**. The terminal ureido NH was more difficult to detect in **3** and **4**, so a terminal ^*t*^Bu urea was chosen for the rest of the study. (Intermolecular interactions by head‐to‐tail hydrogen‐bond formation can occur at high concentrations and low temperature for these triureas, reversing the hydrogen‐bond chain polarity within the self‐aggregates).^[20]^

We synthesized pentaurea **5**, homologous with **2**, to assess the persistence of conformational preference over longer chains. Comparison of the ^1^H NMR chemical shift values of its ureido NH signals with those of shorter analogues (Figure 2a) indicates that the hydrogen‐bond directionality of the chain in **5** is likewise well controlled (Figures S33–S35).

### Conformational response to anion binding

Having established that a terminal BTMP urea induces uniform polarity in a urea oligomer, we investigated switching the directionality of the hydrogen‐bond chain by coordination of different anions at the urea terminus. *N*,*N*’*‐*Disubstituted ureas form stable hydrogen bonded complexes with anions such as carboxylate, chloride or phosphate[^14d^, ^21^] so we envisaged that the BTMP urea that acts as a controller of native directionality in **1**, **2**, **5**, could also act as a binding partner for a range of different anions. With BTMP NH groups being more acidic than *N*‐alkylcarbamoyl NH groups, the reversible coordination of anionic ligands at the most acidic BTMP NH is expected to leave only the carbonyl group of the 3,5‐bis(trifluoromethyl)phenyl urea available for hydrogen bonding with the adjacent urea, leading to a relayed switch of the global conformation of the hydrogen‐bond chain. A trisubstituted urea (with only one free NH group) placed at the opposite terminus of the chain avoids competitive coordination of anionic ligands at both termini, with an *N‐*benzyl substituent also serving as an NMR probe to monitor the directionality of the chain by NOE. We chose as ligands anions of diverse geometries (tetrahedral phosphate diester, planar carboxylate, and spherical chloride) and charge densities, with the tetrabutylammonium counterion ensuring solubility in organic solvents and mitigating any competitive electrostatic binding.

**Figure 3:**
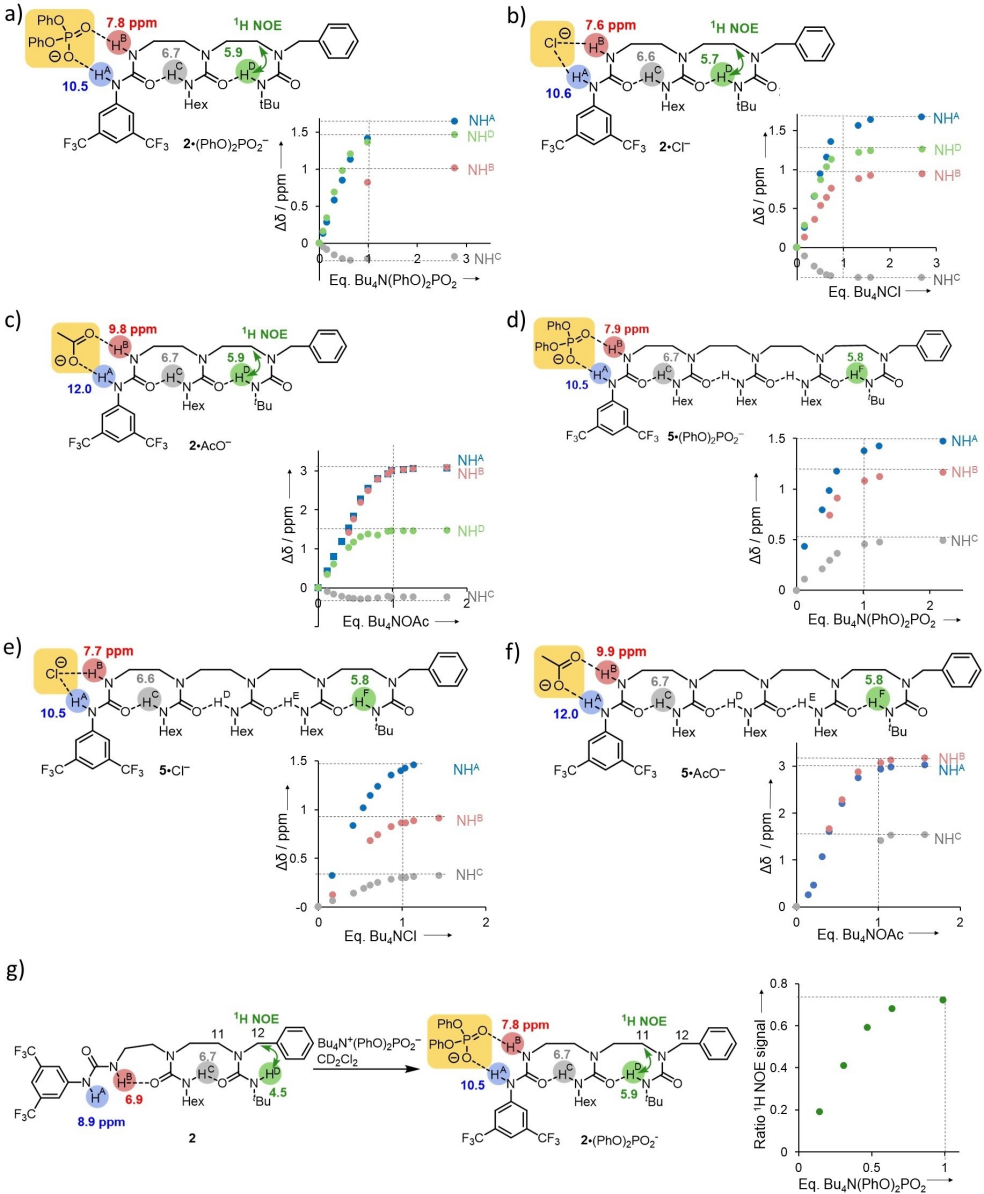
Titration of 2 and 5 (CD_2_Cl_2_ at 25 °C) with anions. ^1^H NMR chemical shift of **2** NH signals in the presence of a) Bu_4_N^+^ (PhO)_2_PO_2_^−^ (3 equiv.), b) Bu_4_N^+^ Cl^−^ (3 equiv.), c) Bu_4_N^+^ AcO^−^ (2 equiv.) and ^1^H NMR chemical shift variation of **2** NH signals upon titration with a) Bu_4_N^+^ (PhO)_2_PO_2_^−^ (K=480^+/−^ 29 % M^−1^), b) Bu_4_N^+^ Cl^−^ (K=1260^+/−^ 40 % M^−1^), c) Bu_4_N^+^ AcO^−^ (K=840^+/−^ 25 % M^−1^). ^1^H NMR chemical shift of **5** NH signals in the presence of d) Bu_4_N^+^ (PhO)_2_PO_2_^−^ (2.2 equiv.), e) Bu_4_N^+^ Cl^−^ (1.5 equiv.), f) Bu_4_N^+^ AcO^−^ (1.6 equiv.) and ^1^H NMR chemical shift variation of **5** NH signals upon titration with d) Bu_4_N^+^ (PhO)_2_PO_2_^−^, e) Bu_4_N^+^ Cl^−^ (K=842 ^+/−^ 28 % M^−1^), f) Bu_4_N^+^ AcO^−^ (K=684^+/−^ 29 % M^−1^. g) Ratio (intensity of ^1^H NMR NOE signal of H^11^ with NH^D^)/(intensity of ^1^H NMR NH NOE signal of H^11^ with H^12^) as a function of quantity of added Bu_4_N^+^ (PhO)_2_PO_2_^−^ for **2** (K=540^+/−^ 25 % M^−1^ ).

We titrated triurea **2** (14.8 mM in CD_2_Cl_2_ at 25 °C, Figure S36) with tetrabutylammonium diphenylphosphate and monitored the conformational effects induced in the oligomer by ^1^H NMR (Figure 3a). Simultaneous progressive downfield shifts of both NH^A^ (Δ*δ* 1.64 ppm) and NH^B^ (Δ*δ* 1.01 ppm) signals occurred upon successive additions of ligand from 0 to 1 equivalent, adding more ligands had minimal effect on the variations of the chemical shifts. The observation is consistent with the regioselective coordination of the ligand to **2**, forming intermolecular hydrogen bonds at both NH^A^ and NH^B^. The progressive shifts of NH signals correspond to weak binding of the ligand. Further evidence of the formation of a complex between **2** and the ligand was provided by a DOSY OneShot ^1^H NMR experiment at 25 °C on compound **2** (17 mM in CD_2_Cl_2_, Figures S43–S45). The diffusion coefficient of **2** changed from 21.5×10^−10^ m^2^ s^−1^ without ligand to 15.8×10^−10^ m^2^ s^−1^ with 1.8 equivalents of tetrabutylammonium di(*p*‐butoxyphenyl)phosphate. Mass spectrometry of a sample of **2** mixed with 1.5 equivalents of tetrabutylammonium diphenyl phosphate using nanospray TOF MS ESI+ also detected the formation of a 1 : 1 complex between the host and the ligand (Figure S46). An association constant was estimated by non‐linear curve fitting analysis of the titration curves of the variation in chemical shift of NH^A^ (Table 1).^[22]^ The ^1^H NMR chemical shift variation of NH^C^ (Δ*δ*=0.18 ppm) during the titration of **2** is small since NH^C^ is engaged in intramolecular hydrogen bonds in both directionalities of the hydrogen‐bond chain. The ^1^H NMR signal for NH^D^ progressively moved downfield (Δ*δ*=1.46 ppm) upon addition of 0 to 1 equivalents of ligand, addition of more of the ligand had minimal effect. The shift of NH^D^ signal being concomitant with the shifts of ^1^H NMR signals of NH^A^ and NH^B^, the ligand stoichiometry is consistent with a switch of global hydrogen‐bond chain directionality induced by coordination of the ligand at the binding site. A NOESY experiment in CD_2_Cl_2_ at 25 °C also supports a ligand‐induced directionality switch. Without ligand, a strong NOE correlation is observed between NH^D^ and H^12^, in addition to a weaker correlation between NH^D^ with H^11^ (Figures S13–S15). In contrast, **2** mixed with 2.76 equivalents of tetrabutylammonium diphenylphosphate displays no NOE correlation between NH^D^ and H^12^ and a strong NOE correlation between NH^D^ and H^11^ (Figures S40). The variation of ratio (intensity of NOE signal of H^11^ with NH^13^)/(intensity of NOE signal of H^11^ with H^12^) as a function of the number of equivalents of ligand is presented in Figure 3g), the NOE signal between H^12^ and H^11^ is expected to be of constant intensity during the titration. The progressive change in polarity of the hydrogen‐bond chain upon addition of ligand resulted in a gradual increase in the intensity of NOE signal between NH^D^ with NH^13^. The association constant calculated by non‐linear curve fitting analysis of the variation of relative intensities of this NOE signal (*K*_a_=539 M^−1^, Table 1, Figures S41 and S42) is of similar order of magnitude to the value obtained by exploiting the ^1^H NMR chemical shift variations at NH^A^; this illustrates the direct correlation between the binding event and the variation of NOE signal at the remote terminus. A simple aqueous wash of the mixture of **2** with ligand restored the initial polarity of **2**.

**Table 1:** Binding constants for oligoureas **2**, **5** with various anions. [a] Determined by non‐linear curve fitting to a 1 : 1 binding model based on NOE signal decay.

Compound	Anion	*K*_1:1_ [M^−1^]	*K*_1:2_ [M^−2^]
**2**	(PhO)PO_2_^−^	480±29 %	38±44 %
**2**	(PhO)PO_2_^−^	539^[a]^ ±25 %	–
**2**	AcO^−^	841±25 %	37±38 %
**2**	Cl^−^	1256±40 %	121±50 %
**5**	AcO^−^	684±29 %	76±31 %
**5**	Cl^−^	842±28 %	50 ±38 %

The titration of **2** (17.8 mM in CD_2_Cl_2_ at 25 °C) with tetrabutylammonium chloride (Figures 3b, S53, ESI Section 6.1.3.) and tetrabutylammonium acetate (Figures 3c, S47, ESI Section 6.1.2.) monitored by ^1^H NMR spectroscopy provided similar results (Table 1). For example, 2D NOESY NMR data of a sample of **2** mixed with either tetrabutylammonium chloride (Figure S57) or acetate (Figure S51) showed a correlation signal of weak intensity between NH^D^ and H^12^, and a correlation of high intensity between NH^D^ and H^11^, confirming the reversal of hydrogen bond chain directionality upon dynamic anion coordination to the BTMP urea. The NOESY spectra illustrate the population‐weighted average over time of all the dynamic conformers – coordinated or not‐ in solution. Mass spectrometry of a sample of **2** with 1.5 equivalents of either ligand detected 1 : 1 complexes between **2** and the ligand (Figures S52 and S58). The titration with tetrabutylammonium acetate led to higher chemical induced shifts for NH^A^ and NH^B^ compared those observed with either tetrabutylammonium chloride or diphenylphosphate (Figure 3c and f), reflecting the greater affinity of BTMP urea towards more basic acetate anions, as already reported for both ureas and squaramides.^[23]^ An aqueous wash of the mixture of **2** and ligands also restored the initial directionality of **2**.

^1^H NMR titrations were conducted on longer homologue **5** with tetrabutylammonium acetate (Figures S74–S78), chloride (Figures S79–S82), and diphenylphosphate (Figures S83–S86), and in each case, binding constants were found of similar values to those with **2** (Table 1). All instances of anion binding resulted in a long‐range hydrogen‐bond directionality switch that was reversed after an aqueous wash (Figures 3d–f).

Control experiments ruled out the possibility that the chemical shift variations measured during the titrations arise because of a direct interaction between the ligands and the hydrogen‐bond chain. Titration of **9** (Figure 2) in CD_2_Cl_2_ at 25 °C with tetrabutylammonium chloride (Figure S68), acetate (Figure S69), and diphenylphosphate (Figure S70) led to significantly less variation of the ^1^H NMR shifts of the ureido NH signals than during the titration of triurea **2** with similar amounts of ligands (for **9**, Δ*δ*=0.08 ppm for chloride, 0.11 ppm for acetate, 0.17 ppm for diphenylphosphate at 1.1 equivalents of ligand added). The titrations of *N,N’’*‐bis(benzyl)‐*N,N’,N’’*‐tri(butylcarbamoyl)‐diethylenetriamine **10** in CD_2_Cl_2_ at 25 °C also induced little variation in the ^1^H NMR chemical shifts of ureido NH signals (Δ*δ*=0.23 ppm for chloride Figure S71, 0.31 ppm for acetate Figure S72, 0.13 ppm for diphenylphosphate (Figure S73 at 1.1 equivalents of ligand added)

### A remote fluorescence response

Having shown that these dynamic foldamers switch their native conformation upon regioselective binding of anions, we used a 2,3‐bis(butylsulfanyl)maleimide fluorophore as a hydrogen bond‐sensitive reporter to reveal changes in hydrogen‐bond polarity remote from the binding terminus (Scheme S5).^[14a]^ This fluorophore is a weak hydrogen bond acceptor exhibiting changes in its nominal emissive wavelength in response to its immediate environment.^[24]^

**Figure 4:**
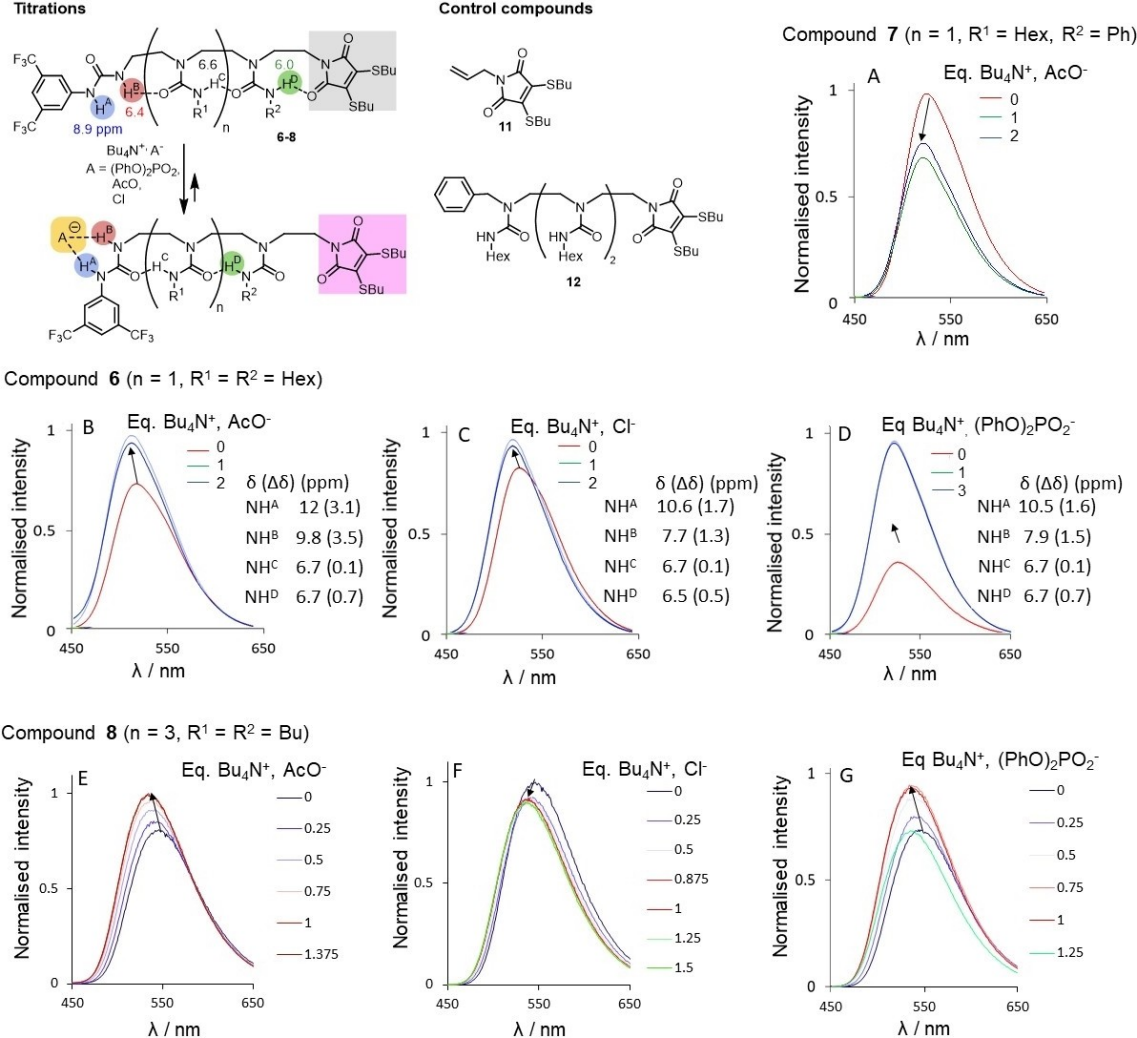
Fluorescence response of compounds 6, 7, 8 in the presence of anions. A) Overlay of fluorescence emission spectra (excitation at 405 nm, 20 °C) of compound **7** (1 mM in CD_2_Cl_2_ at 25 °C) in the presence of Bu_4_N^+^, AcO^−^ (from 0 to 2 equiv.), B–D) of compound **6** (1 mM in CD_2_Cl_2_ at 25 °C) in the presence of either B) Bu_4_N^+^ AcO^−^ (from 0 to 3 equiv.), C) Bu_4_N^+^ Cl^−^ (from 0 to 3 eq.), D) Bu_4_N^+^ (PhO)_2_PO_2_^−^ (from 0 to 3 equiv.); E–G) of compound **8** (1 mM in CD_2_Cl_2_ at 25 °C) in the presence of E) Bu_4_N^+^, AcO^−^ (from 0 to 3 equiv.), F) Bu_4_N^+^, Cl^−^ (from 0 to 3 equiv.), G) Bu_4_N^+^, (PhO)_2_PO_2_^−^ (from 0 to 2 equiv). ^1^H NMR chemical shift (CD_2_Cl_2_ at 25 °C) of the NHs of **6** after addition of ligands Bu_4_N^+^ (PhO)_2_PO_2_^−^ (3.8 equiv.), Bu_4_N^+^ Cl^−^ (2.6 equiv.), Bu_4_N^+^ AcO^−^ (2 equiv.) are indicated (chemical induced shift values are in brackets).

We first checked that the incorporation of the fluorophore does not alter the conformational behaviour of the molecules. **6** (11.7 mM in CD_2_Cl_2_) adopts a native hydrogen‐bond chain conformation identical to its analogues **3** (R^2^=Bn). The ^1^H NMR spectrum of **6** displays one signal for each ureido NH, and their chemical shift values are diagnostic of their hydrogen bonded state (Figure 4, NH^A^ 8.9 ppm, NH^B^ 6.4 ppm, NH^C^ 6.7 ppm, NH^D^ 5.96 ppm, Figures S92, Table S9).

The chemical shift of NH^D^ is higher than the values recorded for parent compound **3**, which is attributed to a weak H‐bonding between NH^D^ with the carbonyl group of the fluorescent reporter. ^1^H NMR spectra of **6** were recorded after addition of tetrabutylammonium diphenylphosphate, acetate or chloride, to assess the binding regioselectivity and polarity switch of hydrogen‐bond chain. The addition of 3.8 equivalents of diphenylphosphate to **6** (11.7 mM in CD_2_Cl_2_ at 25 °C) led to a downfield shift of ^1^H NMR signals for ureido NH^A^ (Δ*δ* 1.56 ppm) and NH^B^ (Δ*δ* 1.47 ppm), attributed to the regioselective coordination of the ligand at the binding site (Figure S93). No chemical induced shift was observed for the inner ureido NH^C^. The ^1^H NMR signal for NH^D^ shifted downfield upon addition of ligand (Δ*δ* 0.72 ppm). Adding 2 equivalents of acetate to **6** (11.6 mM in CD_2_Cl_2_) led to downfield shifts for NH^A^ (Δ*δ* 3.06 ppm), NH^B^ (CI Δ*δ* 3.44 ppm), and NH^D^ (Δ*δ* 0.69 ppm) consistent with a change of the directionality of the hydrogen bond chain (Figure S95). Adding 2.6 equivalents of chloride to **6** (11.6 mM in CD_2_Cl_2_ Figure 4) also led to downfield shifts for NH^A^ (Δ*δ* 1.69 ppm), NH^B^ (Δ*δ* 1.32 ppm), NH^D^ (Δ*δ* 0.52 ppm) (Figure S97). Overall, the ^1^H NMR data are consistent with a regioselective binding of the anions at the BTMP urea concomitant with the switch of global hydrogen‐bond directionality.

Fluorescence spectra of **6** (1 mM in CD_2_Cl_2_) were recorded at 20 °C both before and after addition of tetrabutylammonium diphenylphosphate, acetate, or chloride (from 0 to 3 equivalents, Figure 4A–C). Emission spectra were recorded at 405 nm (the excitation wavelength that provides an emission signal of maximum intensity). For all three ligands, the addition of one equivalent of ligand to a solution of **6** shifted the wavelength corresponding to the maximum intensity of emission from 529 nm (no ligand) to 521 nm (1 equivalent of ligand); the wavelength did not vary further upon addition of more ligand (up to 3 equivalents) (Figures 4, S94, S96, S98). Changing the urea directly adjacent to the fluorophore from an *N*‐butylurea to an *N*‐phenylurea (**7**) did not increase the shift of wavelength of maximal emissive fluorescence, shifting from 526 nm (no ligand) to 521 nm with one equivalent of tetrabutylammonium acetate (Figures 4G, S99).

Control experiments ruled out the possibility that the change in the emissive wavelengths arose from direct interaction of the ligands with the fluorophore. The fluorescence emission spectrum of fluorophore **11** (1 mM in CH_2_Cl_2_ at 25 °C, excitation at 405 nm) was recorded in the absence and then in the presence of either tetrabutylammonium acetate, chloride or diphenylphosphate (3 equivalents). The wavelength of maximum fluorescence emission remained constant at 529 nm before and after addition of ligands (Figure S100). In another control experiment, the fluorescence emission spectra (excitation at 405 nm) and fluorescence excitation spectra (emission wavelength 530 nm) of triurea **12**, which is not equipped with a binding site for anions, (1 mM in CH_2_Cl_2_ at 25 °C) were recorded both before and after addition of increments of ligands from 0 to 2 equivalents. There was minimal variation of the wavelengths of maximum excitation and emission during the addition of ligands (Figures S101–S103).

We synthesized a longer sensor **8** in which four ureas separate the fluorophore from the anion binding site (Scheme S5). The ^1^H NMR chemical shift values for the ureido NH of **8** indicate a native hydrogen‐bond chain directionality identical to its shorter analogue **6** (Figure S107, NH^F^ 6.1 ppm). The titrations of **8** with increments of ligands (tetrabutylammonium acetate, chloride or diphenylphosphate, from 0 to 3 equivalents) were monitored by fluorescence emission and excitation spectroscopy (excitation wavelength at 405 nm, emission wavelength at 530 nm respectively). For all three ligands, the wavelength corresponding to the maximum fluorescence emission shifted from 546 nm with no ligand to 535 nm with 1 equivalent of ligand (Figures 4, S108–S114). Addition of more than 1 equivalent of the ligands did not alter further the wavelength of maximum fluorescence emission.

## Conclusions

A reversible polarity switch in a chain of hydrogen bonds in a dynamic ethylene‐bridged oligourea foldamer is triggered by chloride, acetate, or phosphate anions. An *N*,*N*’‐disubstituted urea serves as a binding site for anionic ligands, and reversible non‐covalent intermolecular coordination of anionic ligands to this urea binding site induces a local conformational change that propagates through the oligomer and induces a change in the fluorescence emission of a fluorophore reporter located at the other terminus of the foldamer. The device thus converts information encoded in the form of anion concentration to spectroscopic outputs at a distal site. The simplicity of the design will allow future addition of further features to create more elaborate functional devices able to perform complex tasks, with potential applications in molecular communication devices and synthetic biology.

## Conflict of interest

The authors declare no conflict of interest.

1

## Supporting information

As a service to our authors and readers, this journal provides supporting information supplied by the authors. Such materials are peer reviewed and may be re‐organized for online delivery, but are not copy‐edited or typeset. Technical support issues arising from supporting information (other than missing files) should be addressed to the authors.

Supporting Information

## Data Availability

The data that support the findings of this study are available in the supplementary material of this article.
