# Supplementary material for: Anion‐Dependent Hydrogen‐Bond Polarity Switching in Ethylene‐bridged Urea Oligomers
Source: Chemistry. 2023 Sep 29;29(62):e202302210. doi: 10.1002/chem.202302210 (PMC10946793; doi:10.1002/chem.202302210)
Supplement: Supplementary file 1 — Supporting Information [file CHEM-29-0-s001.pdf]

Chemistry–A European Journal

Supporting Information

Anion-Dependent Hydrogen-Bond Polarity Switching in Ethylene-bridged Urea Oligomers

David P. Tilly,* David T. J. Morris, and Jonathan Clayden*

Contents

1. General Information 5
2. General synthetic schemes 6
2.1. Synthesis of triureas **2** and **4**.....6
2.2. Synthesis of triureas **1** and **3**.....7
2.3. Synthesis of urea **5** and **s23**.....7
2.4. Synthesis of 2,3-bis(butylsulfanyl)maleimide-N-acetaldehyde **s27** 8
2.5. Synthesis of ureas **6**, **7**, **8**.....8
3. Synthetic procedures and characterisation.....9
3.1. Synthesis of compound **5**.....9
Compound **s24**.....9
Compound **s24 deprotected**.....10
Compound **5**.....10
3.2. Synthesis of oligoureas **6**, **7**.....11
Compound **s28**.....11
Compound **s30**.....11
Compound **s32**.....11
Compound **s33**.....12
Compound **s33di**.....13
Compound **s35**.....13
Compound **s6**.....14
Compound **s7**.....14
3.3. Synthesis of ureas **9** and **12**.....15
Compound **36**.....15
Compound **12**.....16
Compound **9**.....16
3.4. Synthesis of phosphate ligands.....17
4. NMR spectra of synthesised compounds.....19
5. NMR spectroscopy studies establishing native directionalities.....38
5.1. Compound **1**.....38
Assignment of ^{1}H NMR signals.....38
Assignment of directionality.....40
Dilution study in CD_2Cl_2 at 25 °C.....42
Variable temperature ^{1}H NMR study (20 °C to -80 °C).....43
5.2. Compound **2**.....43
Assignment of ^{1}H NMR signals in $CDCl_3$.....43
Assignment of directionality in $CDCl_3$.....47
Assignment of ^{1}H NMR signals and directionality in benzene d6.....47
Assignment of ^{1}H NMR signals in thf d8.....49

5.3. Compound **3**..52
Assignment of ^{1}H NMR signals in CD_2Cl_2..52
Assignment of directionality in CD_2Cl_2..54
5.4. Compound **4**..55
Assignment of ^{1}H NMR signals in CD_2Cl_2..55
Assignment of directionality in CD_2Cl_2..57
Dilution study in CD_2Cl_2 at 25 °C..58
5.5. Compound **5**..58
Assignment of ^{1}H NMR signals in CD_2Cl_2..58
Assignment of directionality in CD_2Cl_2..58
6. Conformational response to anion binding..61
6.1. Compound **2**..61
6.1.1. Titration with tetrabutylammonium diphenylphosphate in CD_2Cl_2 at 25 °C 61
- Monitoring by ^{1}H NMR experiment..61
- Assignment of directionality change by NOE experiments..64
- Characterisation of complex formation by DOSY and HRMS..66
6.1.2. Titration with tetrabutylammonium acetate in CD_2Cl_2 at 25 °C..69
- Monitoring by ^{1}H NMR experiment..69
- Assignment of directionality change by NOESY..72
- Characterisation of complex formation by HRMS..73
6.1.3. Titration with tetrabutylammonium chloride in CD_2Cl_2 at 25 °C..74
- Monitoring by ^{1}H NMR experiment..74
- Assignment of directionality change by NOESY..77
- Characterisation of complex formation by HRMS..78
6.2. Compound **3**..79
6.2.1. Titration with tetrabutylammonium diphenylphosphate in CD_2Cl_2 at 25 °C..79
6.2.2. Titration with tetrabutylammonium acetate in CD_2Cl_2 at 25 °C..82
6.3. Compound **9**..85
6.3.1. Titration with tetrabutylammonium chloride in CD_2Cl_2 at 25 °C..85
6.3.2. Titration with tetrabutylammonium acetate in CD_2Cl_2 at 25 °C..87
6.3.3. Titration with tetrabutylammonium diphenylphosphate in CD_2Cl_2 at 25 °C..88
6.4. Compound **10**..89
6.4.1. Titration with tetrabutylammonium chloride in CD_2Cl_2 at 25 °C..89
6.4.2. Titration with tetrabutylammonium acetate in CD_2Cl_2 at 25 °C..90
6.4.3. Titration with tetrabutylammonium diphenylphosphate in CD_2Cl_2 at 25 °C..91
6.5. Compound **5**..92
6.5.1. Titration with tetrabutylammonium acetate in CD_2Cl_2 at 25 °C..92
6.5.2. Titration with tetrabutylammonium chloride in CD_2Cl_2 at 25 °C..96
6.5.3. Titration with tetrabutylammonium diphenylphosphate in CD_2Cl_2 at 25 °C..99
6.6. Compound **1 -** Titration with tetrabutylammonium acetate in CD_2Cl_2 at 25 °C..103

7. Remote fluorescence response……………………………………………………………………..………..107
7.1. Compound **6**………………………………………………………………………………….…….107
7.1.1. Assignment of native directionality by ^{1}H NMR spectroscopy in CD_2Cl_2 at 25 °C…………………..…...…107
7.1.2. Titration with tetrabutylammonium diphenylphosphate in CD_2Cl_2 at 25 °C……………………...….108
- Monitoring of directionality change by ^{1}H NMR spectroscopy…………………………………….…108
- Fluorescence study……………………………………………………………………………………109
7.1.3. Titration with tetrabutylammonium acetate in CD_2Cl_2 at 25 °C……………………………….….…109
- Monitoring of directionality change by ^{1}H NMR spectroscopy…………………………………….…109
- Fluorescence study……………………………………………………………………..……….110
7.1.4. Titration with tetrabutylammonium chloride in CD_2Cl_2 at 25 °C………………………………………111
- Monitoring of directionality change by ^{1}H NMR spectroscopy……………………………………...111
- Fluorescence study……………………………………………………………………..……...112
7.2. Compound **7**……………………………………………………………………………………...113
- Fluorescence study…………………………………………………………………………….....113
7.3. Control experiments…………………………………………………………………………….....114
7.3.1. Fluorescence study on compound **11** in the absence and presence of ligands…………………….…..114
7.3.2. Fluorescence study on compound **12** in the absence and presence of ligands……………………...…114
7.4. Compound **8**……………………………………………………………………………………...118
7.4.1. ^{1}H NMR study in CD_2Cl_2 at 25 °C……………………………………………………………..…..118
7.4.2. Fluorescence study in the presence of ligands………………………………………………………119

1. General Information

All reactions were performed under a nitrogen atmosphere. All reagents and chemicals were obtained from chemical suppliers and used without further purification. Anhydrous solvents were dispensed under nitrogen from a solvent purification system (Innovative Technologies PureSolve PS-MP-5) or were purchased from commercial suppliers.

Thin-layer chromatography (TLC) was performed using pre-coated plates (Macherey-Nagel Polygram SIL G/UV254). Visualisation was achieved by way of UV light (at 254 nm), and staining with either potassium permanganate, phosphomolybdic acid (in ethanol), or ninhydrin (in ethanol) as stains. Stained TLC plates were heated for visualization. Flash column chromatography was carried out either manually using Fluorochem 60 silica (40-60 μm particle size) or using an automated Biotage® Isolera Spektra Four with gradient elution on pre-packed silica gel Biotage® SNAP Ultra columns or ZIP Sphere columns.

Nuclear Magnetic Resonance spectra (^{1}H NMR and ^{13}C NMR) were recorded on Bruker Nano 400, Jeol ECS 400 or Bruker Avance III HD 500 Cryo with 5 mm DCH 13C–1H/D Cryo Probe (500 MHz) spectrometers. DOSY OneShot NMR experiments were conducted using a Varian VNMRS 500 MHz Direct Drive Spectrometer with Agilent OneNMR probe (500 MHz). Low temperature experiments were recorded on a Jeol ECS 300 spectrometer. Some of the 2D NOESY experiments were recorded on a Bruker Avance III HD 700 (1.7mm micro-cryo) spectrometer. All NMR characterisation experiments were performed at 25 °C and 1 atm unless otherwise stated. Chemical shifts (δ) are quoted in parts per million (ppm) relative to the specified deuterated solvent. Spectra were calibrated using the residual solvent peaks for $CDCl_3$ (δH: 7.26 ppm; δC: 77.16 ppm), CD_2Cl_2 (δH: 5.32 ppm; δC: 53.84 ppm) and $(CD_3)_2SO$ (δH: 2.50 ppm; δC: 39.52 ppm) as appropriate. Coupling constants (J) are quoted in Hz and are rounded to the nearest 0.1 Hz. Splitting patterns are abbreviated to: singlet (s), doublet (d), triplet (t), quartet (q), multiplet (m) or some combination thereof.

Infrared spectra were recorded on a Perkin Elmer Spectrum Two FT-IR spectrometer with samples applied as neat films. Absorptions maxima (νmax) of interest are quoted in wavenumbers as ν in cm-1 for the most intense bands.

Fluorescence spectra were recorded using a PerkinElmer LS45 fluorimeter in a Marco Fluorescence Quartz Cuvette in anhydrous DCM. All fluorescence spectral measurements represent the average of 2 accumulation scans. The photomultiplier voltage is 700 V. All fluorescence spectral measurements represent the average of 2 accumulation scans.

High resolution spectrometry experiments (HR-MS) were recorded by staff at the University of Bristol on a Synapt G2S Waters for nanospray experiments, MicrOTOFII Bruker Daltonics and Orbitrap Elite Thermo Scientific for electrospray ionisation experiments.

Melting points were measured on a Stuart SMP10 melting point apparatus and are uncorrected.

2. General synthetic schemes

2.1. Synthesis of triureas 2 and 4

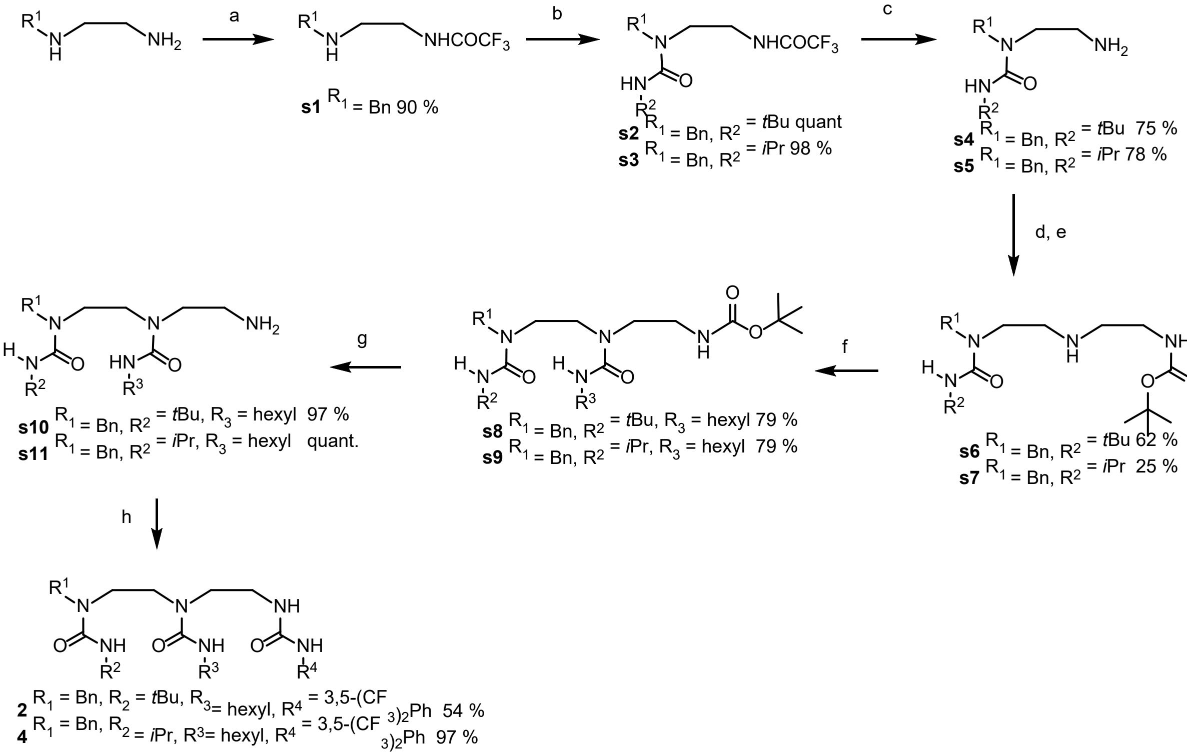

Scheme S1. Synthesis of triureas 2 and 4. Compound **2** was made following reported procedure.[1] Compound **4** was made following reported procedure.[2] Reagents and conditions: (a) CF_3CO_2Et (1 equiv.), MeOH, -78 °C to RT; (b) R^2NCO (1.2 equiv.), 1,2-DCE, 50 °C; (c) NaOH aq. 0.2 M (1 equiv.), MeOH, 40 °C, 12 h.; (d) *N*-boc-2-aminoacetaldehyde (1.5 equiv.), MeOH, RT, 12 h.; (e) $NaBH_4$ (3 equiv.), RT, 5 h.; (f) R^3NCO (1.5 equiv.), CH_2Cl_2, RT, 12 h.; (g) CF_3CO_2H (18 equiv.), CH_2Cl_2, RT, 12 h.; (h) R^4NCO (1.1 equiv.), CH_2Cl_2, RT, 16 h.

2.2. Synthesis of triureas 1 and 3

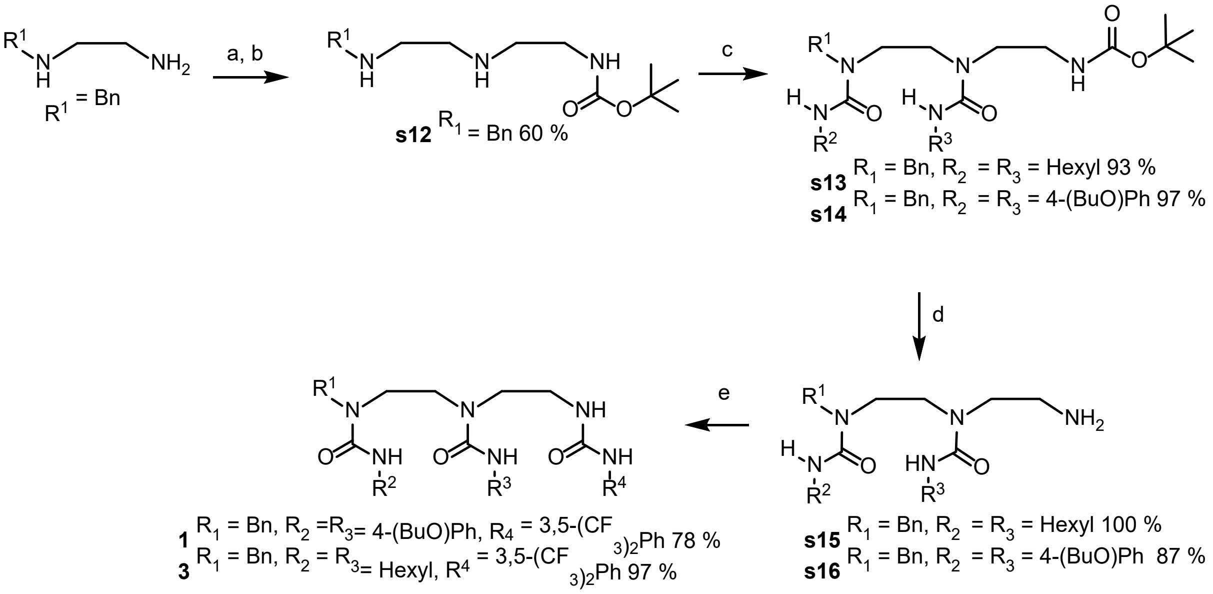

Scheme S2. Synthesis of triureas 1 and 3 were made following reported procedure.[2] Reagents and conditions: (a) *N*-boc-2-aminoacetaldehyde (0.67 equiv.), MeOH, RT, 12 h.; (b) $NaBH_4$ (1.35 equiv.), RT, 5 h.; (c) RNCO (3 equiv.), CH_2Cl_2, RT, 12 h.; (d) CF_3CO_2H (18 equiv.), CH_2Cl_2, RT, 12 h.; (e) R^4NCX (X = O, S) (1.1 equiv.), CH_2Cl_2, RT, 16 h.

2.3. Synthesis of urea 5 and s23

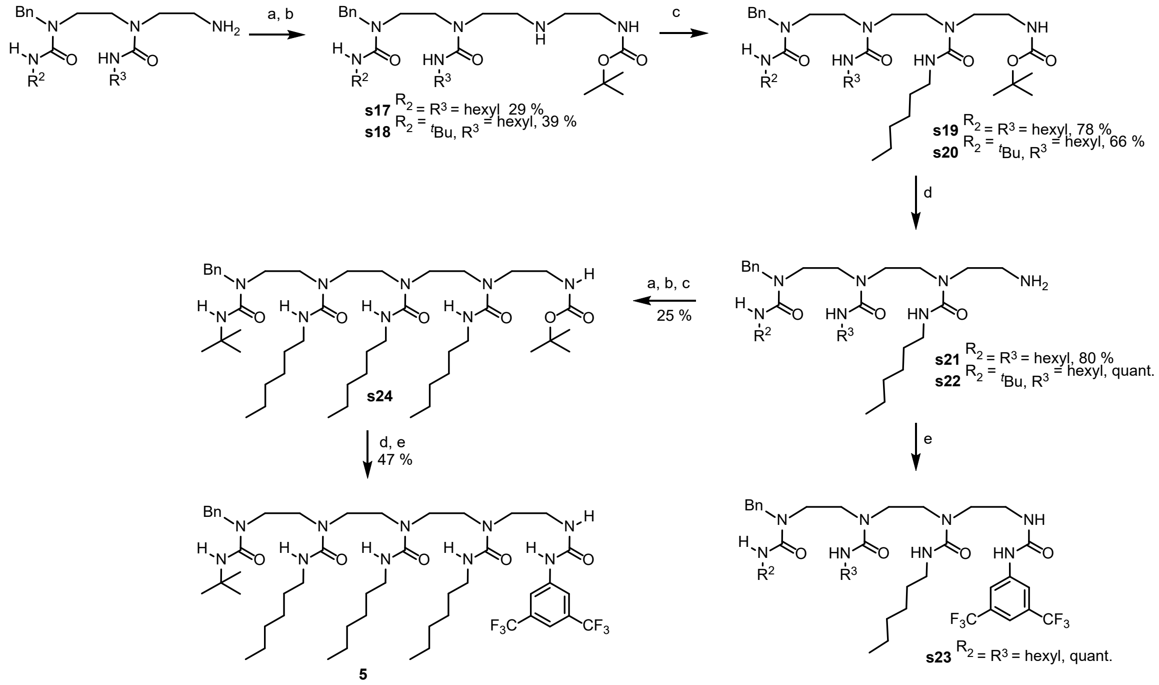

Scheme S3. Synthesis of compound **5**. Reagents and conditions: (a) *N*-boc-2-aminoacetaldehyde (1.5 equiv.), MeOH, RT, 12 h.; (b) $NaBH_4$ (3 equiv.), RT, 5 h.; (c) HexylNCO (1.5 equiv.), CH_2Cl_2, RT, 12 h.; (d) CF_3CO_2H (18 equiv.), CH_2Cl_2, RT, 12 h.; (e) $(CF_3)_2PhNCO$ (1.2 equiv.), CH_2Cl_2, RT, 4 h.

2.4. Synthesis of 2,3-b*is*(butylsulfanyl)maleimide-*N*-acetaldehyde s27

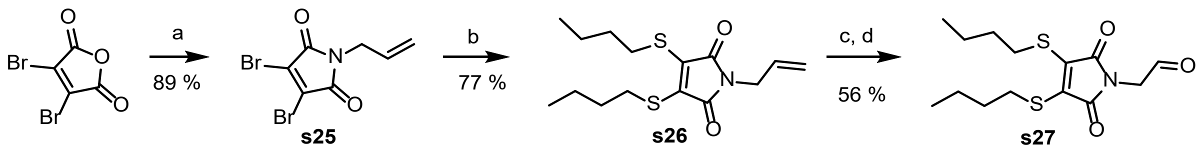

Scheme S4. Synthesis of 2,3-b*is*(butylsulfanyl)maleimide-*N*-acetaldehyde **s27** made following reported procedure.[3] Reagents and conditions: (a) Allylamine (1 equiv.), AcOH, reflux, 5 h.; (b) Et_3N (2.1 quiv.), 20 °C, Et_2O, BuSH (2.1 equiv.), 16 h; (c) OsO_4 (0.1 equiv.), $NaIO_4$ (1 equiv.), 2,6-lutidine (2 equiv.), 1,4-dioxane:water (3:1); (d) $Na_2S_2O_3$ (aq).

2.5. Synthesis of ureas 6, 7, 8

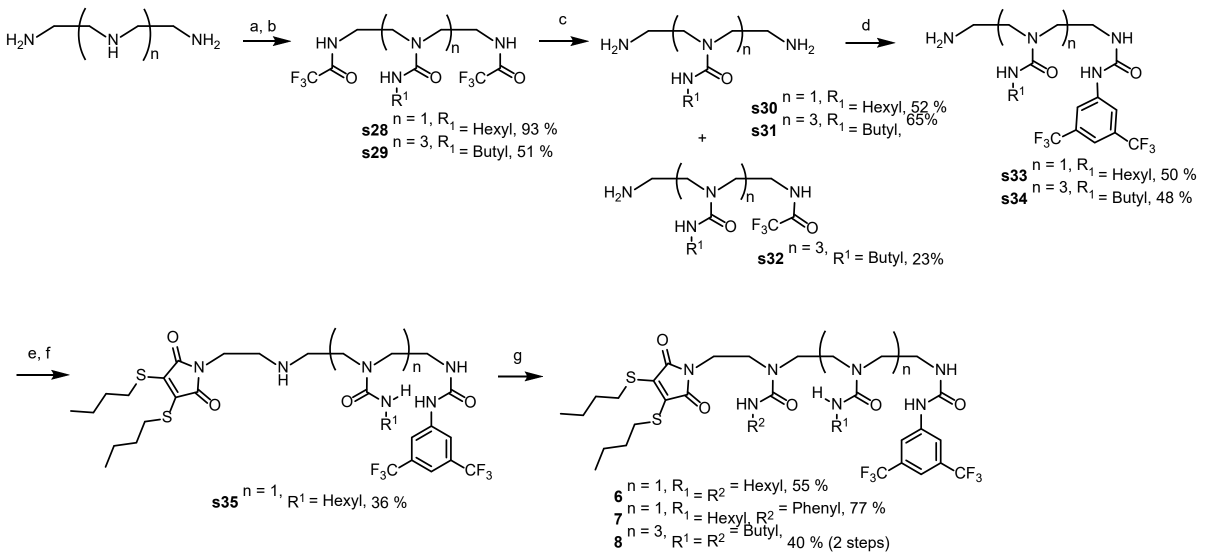

Scheme S5. Synthesis of the oligoureas **6**, **7**, **8** bearing 2,3-b*is*(butylsulfanyl)maleimide. Reagents and conditions: (a) CF_3CO_2Et (2 equiv.), MeOH, -78 °C to RT; (b) R^1NCO, CH_2Cl_2, RT; (c) NaOH aq. 0.2 M (2.2 equiv.), MeOH, 35 °C, 12 h.; (d) $(CF_3)_2PhNCO$ (0.7 equiv.), CH_2Cl_2, RT, 12 h.; (e) 2,3-b*is*(butylsulfanyl)maleimide-*N*-acetaldehyde (1.1 equiv.), CH_2Cl_2, RT, 6h.; (f) 2) $NaBH(OAc)_3$ (1.4 equiv), 40 °C, 12h.; (g) R^2NCO, CH_2Cl_2, RT.

3. Synthetic procedures and characterisation

Compound **2** was made following reported procedure.[1] Compounds **1**, **3**, **4, s23** were made following reported procedure.[2] Compounds **10**, **s29**, **s31**, **s34**, **8**, were made following reported procedure.[3]

3.1. Synthesis of compound 5

Compound s24: *N*'',*N*'',N''''-tri(hexylcarbamoyl)-*N*-benzyl-*N*-(*tert*-butylcarbamoyl)-*N*'-(*tert*-butoxycarbonyl)tetraethylenepentamine

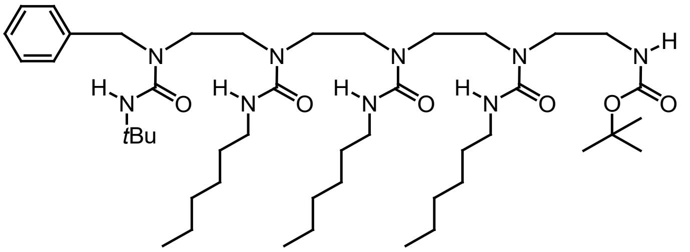

A 0.1 M solution of *N*-benzyl-*N*-(*tert*-butylcarbamoyl)-*N*'',N'''-b*is*(hexylcarbamoyl) triethylenetetramine **s21** (made following reported procedure[2]) (100 mg, 0.17 mmol) in anhydrous MeOH and *N*-boc-2-aminoacetaldehyde (37 mg, 0.23 mmol, 1.5 equiv.) was stirred at 20 °C for 12 hours. Sodium borohydride $NaBH_4$ (10 mg, 0.25 mmol, 3 equiv.) was then added at 0 °C and the resulting mixture was stirred for 5 hours at 20 °C. Water (20 mL) was added and the mixture was stirred for 30 minutes at 20 °C, then was concentrated under reduced pressure to remove MeOH. Brine was added to the resulting aqueous phase and was extracted with $CHCl_3$:*i*PrOH 2:1 mixture, the organic layer was dried over Na_2SO_4, filtered and concentrated under reduced pressure. *N*'',*N*'''-b*is*(hexylcarbamoyl)-*N*-benzyl-*N*-(*tert*-butylcarbamoyl)-*N*'-(*tert*-butoxycarbonyl)tetraethylenepentamine (117 mg) was used without purification as the products tends to degrade on silica. A 0.1M solution of secondary amines in CH_2Cl_2 and hexyl isocyanate (70 µL, 0.48 mmol) was stirred at 20 °C for 12 hours. Water (10 mL) was then added, the resulting mixture was extracted using CH_2Cl_2. The organic layer was dried over Na_2SO_4, filtered and concentrated under reduced pressure. The crude product was purified by silica gel flash chromatography (CH_2Cl_2:MeOH 95:5) to afford the title compound **s24** (35 mg, 0.042 mmol, 25% over 2 steps) as a colourless oil after purification. **^{1}H NMR** (500 MHz, $CDCl_3$) δ 7.36 (m, 2H, 2 x CH_{Ar}), 7.29 (m, 1H, CH_{Ar}), 7.23 – 7.16 (m, 2H, 2 x CH_{Ar}), 6.81 (s, 1H, N*H*$_{Hexyl}$), 6.60 (s, 1H, N*H*$_{Hexyl}$), 6.31 (s, 1H, N*H*$_{Hexyl}$), 5.11 (s, 1H, N*H*Boc), 4.39 (s, 2H, C*H*$_2$Ph), 4.69 (bs, 1H, N*H*tBu), 3.45 – 3.05 (m, 22H, 11 x CH_2), 1.61 – 1.49 (m, 6H, 3 x C*H*$_{2\ hexyl}$), 1.42 (s, 9H, 3 x C*H*$_3$, *t*Bu$_{Boc}$), 1.26 – 1.40 (m, 18H, 9 x C*H*$_{2\ hexyl}$), 1.23 (s, 9H, 3 x C*H*$_3$, *t*Bu$_{urea}$), 0.92 – 0.79 (m, 9H, 3 x CH_3). **^{13}C NMR** (126 MHz, $CDCl_3$) δ 158.9 (3C, 3 x C=O), 158.0 (C=O$_{tBu}$), 156.9 (C=O$_{Boc}$), 137.5 (C_{Ar}), 129.2 (2C, 2 x *C*H$_{Ar}$), 127.9 (*C*H$_{Ar}$), 126.6 (2C, 2 x *C*H$_{Ar}$), 79.9 (*C*(CH_3)$_{3\ Boc}$), 52.5 (CH_2Ph), 51.1 (*C*(CH_3)$_{3\ urea}$), 48.0 – 47.0 (7C, 7 x *C*H$_2$), 42.2 (3C, 3 x *C*H$_2$), 40.2 (*C*H$_2$NHBoc), 31.8 (3C, 3 x *C*H$_2$), 30.1 (*C*H$_2$), 30.0 (*C*H$_2$), 29.9 (*C*H$_2$), 29.3 (3C, 3 x *C*H$_3$C), 28.5 (3C, 3 x *C*H$_{3Boc}$), 26.9 (*C*H$_2$), 26.9 (*C*H$_2$), 26.8 (*C*H$_2$), 22.8 (*C*H$_2$), 22.7 (2 x *C*H$_2$), 14.2 (2C, 2 x *C*H$_3$), 14.2 (*C*H$_3$). **FTIR (neat)** vmax = 3295, 2957, 2928, 2857, 1628, 1538, 1266 cm^{-1}. **HRMS** (ESI, positive ion mode) – *m/z* for $[C_{46}H_{85}N_9O_6+H]^+$ 860.6696, observed 860.6696.

Compound deprotected s24: *N*'',*N*'''',*N*''''''-Tri(hexylcarbamoyl)-*N*-benzyl-*N*-(*tert*-butylcarbamoyl)tetraethylenepentamine

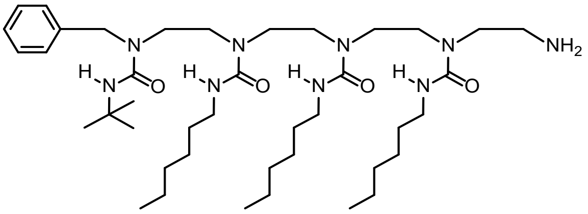

A 0.05 M solution of *N*'',*N*''',N''''-tri(hexylcarbamoyl)-*N*-benzyl-*N*-(*tert*-butylcarbamoyl)-*N*'-(*tert*-butoxycarbonyl)tetraethylenepentamine **s24** (25 mg, 0.03 mmol) in CH_2Cl_2 and trifluoroacetic acid (18 equiv.) was stirred for 12 hours at 20 °C. Water then CH_2Cl_2 were added, the organic layer was washed with aqueous $NaHCO_3$, dried over Na_2SO_4, filtered and concentrated under reduced pressure. The residue was purified by flash chromatography on silica gel (eluent: CH_2Cl_2:MeOH 95:5) to give the unprotected amine (13 mg, 0.017 mmol, 60%) that was not purified further due to degradation on silica.

Compound 5: *N*'',*N*''',N''''-tri(hexylcarbamoyl)-*N*-benzyl-*N*-(*tert*-butylcarbamoyl)-*N'*-(3,5-b*is*(trifluoromethyl)phenylcarbamoyl)tetraethylenepentamine

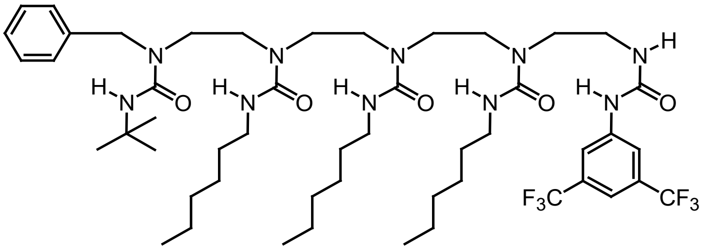

To a 0.05 M solution of primary amine *N*'',*N*''',N''''-tri(hexylcarbamoyl)-*N*-benzyl-*N*-(*tert*-butylcarbamoyl)tetraethylenepentamine (**deprotected s24**) (13 mg, 0.017 mmol) in CH_2Cl_2 was added dropwise at 20 °C 3,5-b*is*(trifluoromethyl)phenyl isocyanate (3 µL, 1.2 equiv. per primary amine), the mixture was stirred for 4 hours. Addition of water was followed with extraction of the aqueous layer using CH_2Cl_2. The organic layer was dried over Na_2SO_4, filtered and concentrated under reduced pressure. The residue was purified by flash chromatography (eluent: CH_2Cl_2:MeOH 95:5) on silica to give the title compound **5** isolated as a colourless oil (8 mg, 0.008 mmol, 47%). **^{1}H NMR** (500 MHz, CD_2Cl_2) δ 8.96 (bs, 1H, NH_eAr), 7.97 (s, 2H, 2 x CH_{Ar}), 7.42 (s, 1H, CH_{Ar}), 7.35 (dd, 2H, 2 x CH_{Ar}), 7.27 (t, 1H, CH_{Ar}), 7.21 (d, *J* = 7.6 Hz, 2H, 2 x CH_{Ar}), 7.01 (bt, 1H, NH_b), 6.93 (bt, 1H, NH_d), 6.73 (bt, 1H, NH_f), 6.23 (bt, 1H, NH_c), 4.40 (bs, 2H, CH_2Ph), 4.29 (s, 1H, NH_a*t*Bu), 3.49 – 3.02 (m, 22H, 3 x *CH₂*NH, 8 x CH_2N), 1.60 – 1.43 (m, 6H, 3 x CH_2), 1.39 – 1.17 (m, 27H, *t*Bu, 9 x CH_2), 0.91 – 0.85 (m, 6H, 2 x CH_3), 0.83 (t, 3H, CH_3). **^{13}C NMR** (126 MHz, CD_2Cl_2) δ 159.4 (2 x *C*=O), 159.3 (*C*(O)NH_d), 158.6 (*C*(O)NH*t*Bu), 156.4 (*C*(O)NHAr), 142.6 (C_{Ar}), 138.2 (C_{Ar}), 132.2 (q, *J* = 32.9 Hz, 2C, 2 x *C*CF_3), 129.5 (CH_{Ar}), 128.2 (*C*H_{Ar}), 126.9 (*C*H_{Ar}), 124.1 (q, *J* = 272.5 Hz, 2C, 2 x *C*F_3), 118.2 (CH_{Ar}), 114.9 (CH_{Ar}), 53.2 (*C*H_2Ph), 51.5 (*C*(CH_3)), 49.2 (N*C*H_2), 48.5 (2 x N*C*H_2), 48.3 (2 x N*C*H_2), 48.2 (N*C*H_2), 48.0 (N*C*H_2), 41.7 (*C*H_2), 41.6 (*C*H_2), 41.6 (*C*H_2), 40.3 (N*C*H_2), 32.2 (*C*H_2), 32.2(*C*H_2), 32.1 (*C*H_2), 30.5 (*C*H_2), 30.4 (*C*H_2), 30.3 (*C*H_2), 29.5 (3 x *C*H_3C), 27.3 (*C*H_2), 27.3(*C*H_2), 27.2 (*C*H_2), 23.2 (*C*H_2), 23.2 (*C*H_2), 23.1 (*C*H_2), 14.4 (*C*H_3), 14.4 (*C*H_3), 14.3 (*C*H_3). **FTIR (neat)** νmax = 3299, 2957, 2929, 2869, 1630, 1559, 1388, 1276, 1135 cm^{-1}. **HRMS** (ESI, positive ion mode) – *m/z* for $[C_{50}H_{80}F_6N_{10}O_5+H]^+$ 1015.6290, observed 1015.6268, – *m/z* for $[C_{50}H_{80}F_6N_{10}O_5+Na]^+$ 1037.6110, observed 1037.6086.

3.2. Synthesis of oligoureas 6, 7, 8

Compound s28: *N,N'-bis*(trifluoroacetyl)-*N*''-(hexylcarbamoyl)diethylenetriamine

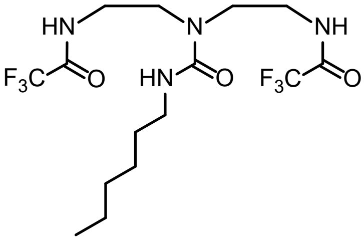

A solution of diethylenetriamine (1 mL, 9.2 mmol, 1.00 equiv, 1.6 M) in anhydrous MeOH was cooled to -78 °C then ethyl trifluoroacetate (2.2 mL, 18.5 mmol, 1 equiv. per *primary* amine) dissolved in methanol was added dropwise over 30 minutes. The resultant solution was stirred for 12 hours while allowing the temperature to raise to 20 °C. The mixture was concentrated under reduced pressure then purified using silica gel flash chromatography (eluent: 100% MeOH). The resulting *bis*-trifluoroacetamide *N,N'*-b*is*(trifluoroacetyl)diethylenetriamine was obtained as a colourless solid (2.566 g, 8.74 mmol, 95%). The *bis*-trifluoroacetamide (1.86 g, 6.28 mmol) was dissolved in anhydrous CH_2Cl_2 to a concentration of 0.5M, then hexyl isocyanate (1 mL, 6.9 mmol) was added to the mixture and the resultant solution was stirred at 40 °C for 16 hours. After concentration under reduced pressure, the residue was purified using silica gel flash chromatography (eluent: 97:3 CH_2Cl_2:MeOH), providing the title compound **s28** as yellow oil (2.57 g, 6.09 mmol, 97%). **^{1}H NMR** (400 MHz, $CDCl_3$) δ 8.17 (m, 2H, 2 x N*H*$COCF_3$), 5.59 (t, 1H, *J* = 5.3 Hz, N*H*), 3.45 (m, 8H, C*H*$_2$N), 3.18 (td, *J* = 7.3, 5.3 Hz, 2H, C*H*$_2$), 1.55 – 1.42 (m, 2H, C*H*$_2$), 1.36 – 1.19 (m, 6H, 3 x C*H*$_{2\ hexyl}$), 0.91 – 0.80 (m, 3H, C*H*$_{3\ hexyl}$). **^{13}C NMR** (101 MHz, $CDCl_3$) δ 159.3 (*C*=O), 159.2 (q, *J* = 37.5 Hz, 2C, 2x (*C=O)*CF_3), 115.7 (q, *J* = 287.1 Hz, 2C, 2 x *C*F_3), 46.1 (2C, 2 x *C*H_2N), 41.5 (*C*$H_{2\ hexyl}$), 39.8 (2C, 2 x *C*H_2N), 31.6 (*C*$H_{2\ hexyl}$), 29.8 (*C*$H_{2\ hexyl}$), 26.6 (*C*$H_{2\ hexyl}$), 22.6 (*C*$H_{2\ hexyl}$), 14.1 (*C*H_3 $_{hexyl}$). **FTIR (neat)** vmax = 1554, 1504, 1432 cm^{-1}. **HRMS** (ESI, positive ion mode) – *m/z* for $[C_{15}H_{24}F_6N_4O_3+H]^+$ 423.1825, observed 423.1833.

Compound s30: *N*''-(hexylcarbamoyl)diethylenetriamine

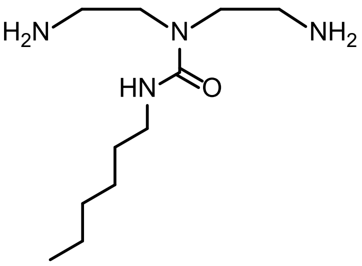

To a solution of *N,N'*-b*is*(trifluoroacetyl)-*N*''-(hexylcarbamoyl)diethylenetriamine **s28** (2.326 g, 5.49 mmol) in MeOH (50 mL) was added 2.2 equiv. of an aqueous solution of sodium hydroxide (0.2 M, 58 mL). The resultant mixture was stirred for 12 hours at 35 °C. The methanol was removed under reduced pressure, the aqueous layer was extracted using a mixture ($CHCl_3$:*i*PrOH 3:1), the combined organic extracts concentrated under reduced pressure to give the crude primary amine. The residue was purified using silica gel flash chromatography (eluent gradient from CH_2Cl_2:MeOH 90:10 to MeOH:NEt_3 99:1), the title diamine **s30** was obtained (645 mg, 2.85 mmol, 52%) as a colourless oil. **^{1}H NMR** (400 MHz, $CDCl_3$) δ 6.53 (t, *J* = 5.3 Hz, 1H, NH_{urea}), 3.20 (t, *J* = 6.0 Hz, 4H, 2 x C*H*$_2$N), 3.04 (td, *J* = 7.1, 5.3 Hz, 2H, C*H*$_{2\ hexyl}$), 2.75 (t, *J* = 6.0 Hz, 4H, 2 x C*H*$_2$$NH_2$), 1.76 – 1.38 (m, 4H, 2 x N*H*$_2$), 1.42 – 1.32 (m, 2H, C*H*$_2$), 1.25 – 1.12 (m, 6H, 3 x C*H*$_{2\ hexyl}$), 0.89 – 0.64 (m, 3H, C*H*$_{3\ hexyl}$). **^{13}C NMR** (101 MHz, $CDCl_3$) δ 160.1 (*C=O*), 50.8 (2C, 2 x *C*H_2N), 41.0 (*C*$H_{2\ hexyl}$), 40.7 (2 C, 2 x

CH_2N), 31.5 ($CH_{2\ hexyl}$), 30.1 ($CH_{2\ hexyl}$), 26.7 ($CH_{2\ hexyl}$), 22.5 ($CH_{2\ hexyl}$), 13.9 ($CH_{3\ hexyl}$). **FTIR (neat)** νmax = 3403, 1628, 1541 cm^{-1}. **HRMS** (ESI, positive ion mode) – *m/z* for $[C_{11}H_{26}N_4O+H]^+$ 231.2179, observed 231.2174.

Compound s32: ***N*-(trifluoroacetyl)- *N*'',*N*''',*N*''''-tri(butylcarbamoyl)tetraethylenepentamine**

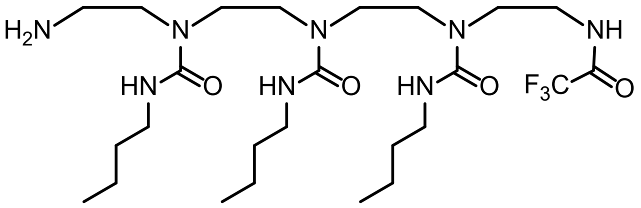

Colourless oil. **¹H NMR** (400 MHz, DMSO – d_6) δ 9.54 (s, 1H, N*H*COCF₃), 7.77 (s, 2H, N*H*₂), 6.79 (t, *J* = 4.6 Hz, 2H, N*H* butyl), 6.58 (s, 1H, N*H* butyl), 3.35 (m, 6H, 3 x C*H*₂N), 3.22 (d, *J* = 5.8 Hz, 8H, 4 x C*H*₂N), 3.04 (m, 6H, 3 x C*H*₂ butyl), 2.87 (t, *J* = 6.3 Hz, 2H, C*H*₂NH₂), 1.50 – 1.35 (m, 6H, 3 x C*H*₂ butyl), 1.36 – 1.20 (m, 6H, 3 x C*H*₂ butyl), 0.87 (t, *J* = 7.3 Hz, 9H, 3 x C*H*₃ butyl). **¹³C NMR** (126 MHz, DMSO – d_6) δ 158.1 (3 x *C*=O urea), 158.0 (*C*=OCF₃), 116.1 (q, *J* = 288.15 Hz, 2C, 2 x *C*F₃), 46.2 (C*H*₂ N), 46.2 (C*H*₂ N), 46.1 (3C, 3 x C*H*₂ N), 45.7 (C*H*₂ N), 45.3 (C*H*₂ N), 40.1 (C*H*₂ butyl), 39.9 (C*H*₂ butyl), 39.8 (C*H*₂ butyl), 38.6 (C*H*₂ butyl), 38.4 (C*H*₂N), 32.0 (2C, 2 x C*H*₂ butyl), 31.9 (C*H*₂ butyl), 19.8 (C*H*₂ butyl), 19.7 (2C, 2 x C*H*₂ butyl), 13.9 (3 x C*H*₃ butyl). **FTIR (neat)** νmax = 3343, 2925, 1714, 1624, 1546, 1219 cm^{-1}. **HRMS** (ESI, positive ion mode) – *m/z* for $[C_{25}H_{49}F_3N_8O_4+H]^+$ 583.3902, observed 583.3912.

Compound s33: ***N*-(3,5-b*is*(trifluoromethyl)phenylcarbamoyl)- *N*''-(hexylcarbamoyl)diethylenetriamine**

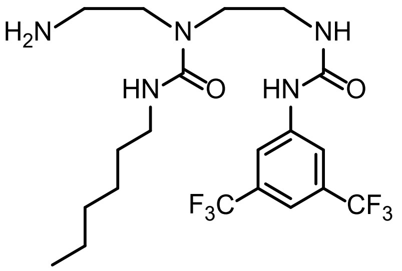

To a solution of *N*''-(hexylcarbamoyl)diethylenetriamine **s30** (305 mg, 1.34 mmol) in anhydrous CH_2Cl_2 (25 mL) was added dropwise a solution of 3,5-b*is*(trifluoromethyl)phenyl isocyanate (160 µL, 0.94 mmol, 0.7 equiv.) dissolved in CH_2Cl_2 (5 mL) then the mixture was stirred for 12 hours at 20 °C. After concentration under reduced pressure, the residue was purified using silica gel flash chromatography (gradient CH_2Cl_2:MeOH 90:10 to 0:100) to afford the title compound **s33** as a white solid (228 mg, 0.67 mmol, 50%) along with some disubstituted compound **s33di** as a white solid (220 mg, 0.42 mmol, 32%). **¹H NMR** (400 MHz, $CDCl_3$) δ 9.19 (s, 1H, NH), 7.92 (s, 2H, 2 x C*H*Ar), 7.39 (s, 1H, C*H*Ar), 7.32 (s, 1H, N*H*), 6.88 (s, 1H, N*H*), 3.47 – 3.32 (m, 6H, 3 x C*H*₂N), 3.11 (td, *J* = 7.1, 5.0 Hz, 2H, C*H*₂NH Ar urea), 2.96 (m, 2H, C*H*₂NH₂), 2.39 (s, 2H, N*H*₂), 1.42 (m, 2H, C*H*₂ hexyl), 1.32 – 1.04 (m, 6H, 3 x C*H*₂ hexyl), 0.79 (t, *J* = 6.9 Hz, 3H, C*H*₃ hexyl). **¹³C NMR** (101 MHz, $CDCl_3$) δ 161.1 (C=O hexylurea), 156.3 (C=O Ar urea), 141.9 (*C*Ar), 132.0 (q, *J* = 32.9 Hz, 2C, 2 x *C*CF₃), 123.5 (q, *J* = 272.8 Hz, 2C, 2 x *C*F₃), 117.9 (2C, 2 x *C*HAr), 114.8 (*C*HAr), 51.5 (*C*H₂N), 47.6 (*C*H₂N), 41.4 (*C*H₂N), 41.3 (*C*H₂ hexyl), 38.9 (*C*H₂N), 31.5 (*C*H₂ hexyl), 29.9 (*C*H₂ hexyl), 26.8 (*C*H₂ hexyl), 22.6 (*C*H₂ hexyl), 14.0 (*C*H₃ hexyl). **FTIR (neat)** νmax = 3301, 2931, 2850, 1567, 1386, 1275, 1127 cm^{-1}. **HRMS** (ESI, positive ion mode) – *m/z* for $[C_{20}H_{29}F_6N_5O_2+Na]^+$ 508.2118, observed 508.2122. **MP** 132-134 °C.

Compound s33di: ***N,N'*-b*is*(3,5-b*is*(trifluoromethyl)phenylcarbamoyl)-*N*''-(hexylcarbamoyl)diethylenetriamine**

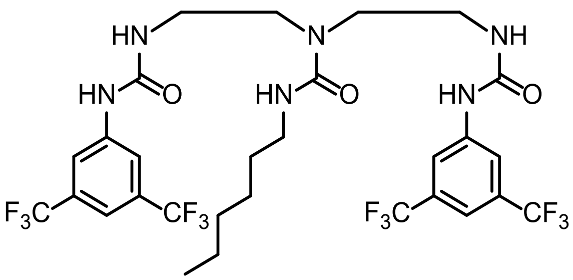

^{1}H NMR (400 MHz, DMSO – d_6) δ 9.79 – 9.54 (s, 2H, 2 x N*H*Ar), 8.18 – 7.94 (s, 4H, 4 x C*H*$_{Ar}$), 7.54 – 7.34 (s, 2H, 2 x C*H*$_{Ar}$), 6.81– 6.54 (t, *J* = 5.5 Hz, 2H, 2 x NH), 6.52 – 6.22 (t, *J* = 5.4 Hz, 1H, N*H*Hexyl), 3.33 – 3.27 (d, *J* = 6.4 Hz, 4H, 2 x C*H*$_2$N), 3.27 – 3.09 (q, *J* = 6.8, 6.3 Hz, 4H, 2 x C*H*$_2$N), 3.09 – 2.94 (m, 2H, C*H*$_2$N), 1.49 – 1.29 (m, 2H, C*H*$_{2\ hexyl}$), 1.29 – 1.05 (m, 6H, 3 x C*H*$_{2\ hexyl}$), 0.86 – 0.59 (t, *J* = 6.8 Hz, 3H, C*H*$_{3\ hexyl}$). **^{13}C NMR** (101 MHz, DMSO – d_6) δ 157.7 (C=O), 155.3 (2 x C=O), 142.6 (*C*$_{Ar}$), 130.6 (q, *J* = 32.5 Hz, 4C, 4 x *C*CF$_3$), 123.4 (q, *J* = 272.7 Hz, 4C, 4 x *C*F$_3$), 117.1 (4C, 4 x *C*H$_{Ar}$), 113.3 (2C, 2 x *C*H$_{Ar}$), 46.6 (2C, 2 x *C*H$_2$N), 40.3 (2C, 2 x *C*H$_2$N), 38.5 (2C, 2 x *C*H$_2$NH), 31.0 (*C*H$_{2\ hexyl}$), 29.6 (*C*H$_{2\ hexyl}$), 26.1 (*C*H$_{2\ hexyl}$), 22.0 (*C*H$_{2\ hexyl}$), 13.7 (*C*H$_{3\ hexyl}$). **FTIR (neat)** νmax = 3387, 1692, 1567, 1388, 1276 cm^{-1}. **HRMS** (ESI, positive ion mode) – *m/z* for $[C_{29}H_{32}F_{12}N_6O_3+H]^+$ 741.2417, observed 741.2407. **MP** 241-242 °C.

Compound s35: ***N*-(3,5-b*is*(trifluoromethyl)phenylcarbamoyl)-*N*'-(2,3-b*is*(butylsulfanyl)maleimidoethyl)-*N*''-(hexylcarbamoyl)diethylenetriamine**

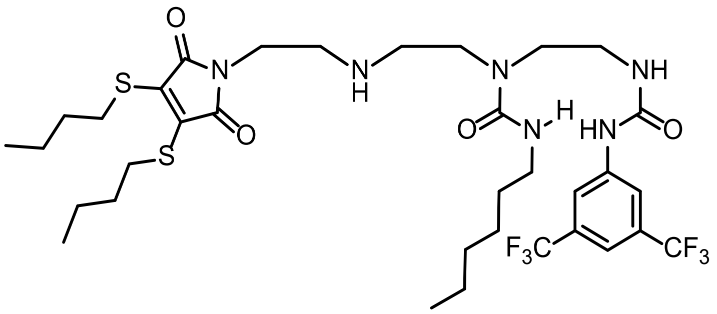

A solution of *N*-(3,5-b*is*(trifluoromethyl)phenylcarbamoyl)-*N*''-(hexylcarbamoyl)diethylenetriamine **s33** (35 mg, 0.072 mmol) and 2,3-b*is*(butylsulfanyl)maleimide-*N*-acetaldehyde **s27** (made following reported procedure[3]) (27 mg, 0.08 mmol) in anhydrous CH_2Cl_2 (2 mL) was stirred for 6 hours at 20 °C. To this solution was added sodium triacetoxyborohydride NaBH(OAc)$_3$ (25 mg, 0.12 mmol, 1.4 equiv.) and the mixture was stirred at 40 °C for 12 hours. The solution was diluted with saturated aqueous sodium hydrogen carbonate (10 mL). The aqueous phase was extracted with CH_2Cl_2 (3 x 10 mL) and the combined organic extracts were dried (Na_2SO_4), filtered and concentrated under reduced pressure. The residue was purified using silica gel flash chromatography (CH_2Cl_2:MeOH 85:15) to afford the title compound **s35** as a yellow oil (20 mg, 0.026 mmol, 36%). **^{1}H NMR** (400 MHz, $CDCl_3$) δ 9.04 – 8.31 (s, 1H, N*H*), 8.03 – 7.80 (s, 2H, C*H*$_{Ar}$), 7.40 – 7.30 (s, 1H, C*H*$_{Ar}$), 6.97 – 6.24 (m, 2H, 2 x N*H*), 3.62 – 3.49 (t, *J* = 5.7 Hz, 2H, C*H*$_2$N), 3.39 – 3.33 (d, *J* = 5.2 Hz, 2H, C*H*$_2$N), 3.32 – 3.28 (d, *J* = 5.5 Hz, 2H, C*H*$_2$N), 3.28 – 3.20 (t, *J* = 5.3 Hz, 2H, C*H*$_2$N), 3.23 – 3.14 (m, 4H, 2 x CH$_2$S), 3.09 – 2.99 (m, 2H, C*H*$_2$NH), 2.85 – 2.72 (m, 4H, 2 x C*H2*), 1.61 – 1.46 (m, 4H, 2 x C*H*$_2$), 1.45 – 1.27 (m, 6H, 3 x C*H*$_2$), 1.23 – 1.00 (m, 6H, 3 x C*H*$_2$), 0.91 – 0.76 (t, *J* = 7.3 Hz, 6H, 2 x C*H*$_{3\ Butyl}$), 0.76 – 0.62 (t, *J* = 6.8 Hz, 3H, C*H*$_{3\ Hexyl}$). **^{13}C NMR** (101 MHz, $CDCl_3$) δ 167.1 (2C, 2 x *C*=O), 160.7 (*C*=O), 156.1 (*C*=O), 141.8 (*C*$_{Ar}$), 136.0 (2C, 2 x *C*S), 132.0 (q, *J* = 33.0 Hz, 2C, 2 x C$_{Ar}$CF$_3$), 123.5 (q, *J* = 272.1 Hz, 2C, 2 x *C*F$_3$), 118.1 (2C, 2 x *C*H$_{Ar}$), 114.9 (*C*H$_{Ar}$), 49.2 (*C*H$_2$N), 48.6 (*C*H$_2$N), 48.1 (*C*H$_2$N),

47.9 (CH_2N), 41.3 (CH_2N), 39.2 (CH_2N), 38.0 ($CH_{2\ hexyl}$), 32.6 (2C, 2x $CH_{2\ Butyl}$), 31.7 (2C, 2 x $CH_{2\ Butyl}$), 31.6 ($CH_{2\ hexyl}$), 30.2 ($CH_{2\ hexyl}$), 26.9 ($CH_{2\ hexyl}$), 22.7 ($CH_{2\ hexyl}$), 21.8 (2C, 2 x $CH_{2\ Butyl}$), 14.0 ($CH_{3\ hexyl}$), 13.7 (2C, 2 x $CH_{3\ Butyl}$). **FTIR (neat)** vmax = 2917, 2849, 1706, 1566, 1264 cm^{-1}. **HRMS** (ESI, positive ion mode) – *m/z* for $[C_{34}H_{50}F_6N_6O_4S_2+H]^+$ 785.3312, observed 785.3300.

Compound 6: *N*-(3,5-b*is*(trifluoromethyl)phenylcarbamoyl)-*N*'-(2,3-b*is*(butylsulfanyl)maleimidoethyl)-*N*',N''-b*is*(hexylcarbamoyl)diethylenetriamine

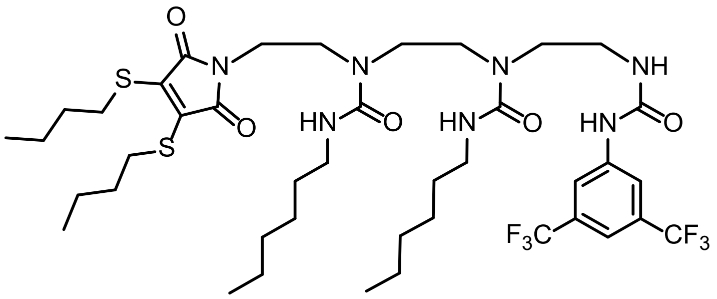

To a solution of *N*-(3,5-b*is*(trifluoromethyl)phenylcarbamoyl)-*N*'-(2,3-b*is*(butylsulfanyl)maleimidoethyl)-*N*''-(hexylcarbamoyl)diethylenetriamine **s35** (40 mg, 0.05 mmol) in anhydrous CH_2Cl_2 (1 mL) was added hexyl isocyanate (0.02 mL, 0.08 mmol) and the mixture was stirred at 20 °C for 12 hours. Water (5 mL) was added and the mixture was extracted with CH_2Cl_2. The organic layer was dried (Na_2SO_4), filtered and concentrated under reduced pressure. The residue was purified using silica gel flash chromatography (eluent: CH_2Cl_2:MeOH 95:5) to give the title compound **6** as a yellow solid (25 mg, 0.03 mmol, 55%). **^{1}H NMR** (400 MHz, CD_2Cl_2) δ 9.22– 8.60 (s, 1H, NH_{Ar}), 8.02 (s, 2H, 2 x CH_{Ar}), 7.43 (s, 1H, CH_{Ar}), 6.89 – 6.51 (m, 1H, N*H*), 6.51 – 6.20 (m, 1H, N*H*), 6.20 –5.61 (m, 1H, N*H*), 3.67 – 3.60 (t, *J* = 6.8 Hz, 2H, CH_2N), 3.53 – 3.28 (m, 10H, 5 x CH_2N), 3.28 – 3.21 (m, 4H, 2 x CH_2N), 3.21 – 3.08 (tt, *J* = 7.9, 4.9 Hz, 4H, 2 x CH_2NH), 1.67 – 1.56 (m, 4H, 2 x CH_2), 1.56 – 1.36 (m, 8H, 4 x CH_2), 1.36 – 1.15 (m, 12H, 6 x CH_2), 0.94 – 0.89 (t, 6H, *J* = 7.3 Hz, 2 x CH_3), 0.86 (t, *J* = 6.8 Hz, 3H, CH_3), 0.82 (t, *J* = 6.9 Hz, 3H, CH_3). **^{13}C NMR** (101 MHz, CD_2Cl_2) δ 167.5 (2C, 2 x C=O), 159.7 (C=O), 159.0 (C=O), 156.6 (C=O), 142.6 (C_{Ar}), 136.7 (2C, 2 x *C*S), 132.3 (q, *J* = 33.0 Hz, 2C, 2 x $C_{Ar}CF_3$), 124.1 (q, *J* = 272.6 Hz, 2C, 2 x *C*F_3), 118.3 (2C, 2 x CH_{Ar}), 115.1 (CH_{Ar}), 48.4 (CH_2), 47.5 (CH_2), 47.2 (CH_2), 46.3 (CH_2), 41.9 (CH_2), 41.8 (CH_2), 39.8 (CH_2), 37.2 (CH_2), 33.1 (2C, 2 x CH_2), 32.2 (2C, 2 x CH_2), 32.1 (CH_2), 30.5 (CH_2), 30.4 (CH_2), 30.3 (CH_2), 27.2 (CH_2), 27.2 (CH_2), 23.2 (CH_2), 23.1 (CH_2), 22.2 (2C, 2 x CH_2), 14.4 (CH_3), 14.3 (CH_3), 13.9 (2C, 2 x CH_3). **FTIR (neat)** vmax = 3299, 2958, 2929, 2859, 1708, 1632, 1564, 1387, 1277 cm^{-1}. **HRMS** (ESI, positive ion mode) – *m/z* for $[C_{41}H_{63}F_6N_7O_5S_2+H]^+$ 912.4309, observed 912.4301. **M.P.** 112-113 °C.

Compound 7: *N*-(3,5-b*is*(trifluoromethyl)phenylcarbamoyl)-*N*'-(2,3-b*is*(butylsulfanyl)maleimidoethyl)-*N*'-(phenylcarbamoyl)-N''-(hexylcarbamoyl)diethylenetriamine

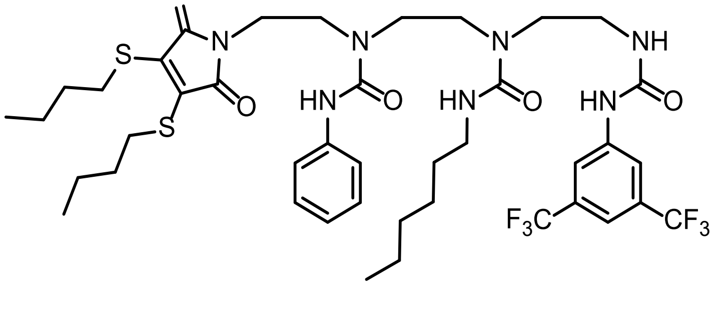

To a solution of *N*-(3,5-b*is*(trifluoromethyl)phenylcarbamoyl)-*N*'-(2,3-b*is*(butylsulfanyl)maleimidoethyl)-*N*''-(hexylcarbamoyl)diethylenetriamine **s35** (10 mg, 0.013 mmol) in anhydrous CH_2Cl_2 (0.5 mL) was added phenylisocyanate (5 µL, 0.038 mmol) and the mixture was stirred at 20 °C for 4 hours. Water (5 mL) was added and the mixture was extracted with CH_2Cl_2. The organic layer was dried (Na_2SO_4), filtered and concentrated under reduced pressure. The residue was purified using silica gel flash chromatography (eluent: CH_2Cl_2:MeOH 95:5) to give the title compound **7** as a yellow oil (9 mg, 0.01 mmol, 77%). **^{1}H NMR** (400 MHz, CD_2Cl_2) δ 9.71-9.24 (s, 1H, N*H*), 8.16 – 7.82 (s, 1H, N*H*), 8.19 – 8.06 (s, 2H, 2 x CH_{Ar}), 7.56 – 7.48 (d, 2H, *J* = 7.3 Hz, 2 x CH_{Ar}), 7.48 – 7.44 (s, 1H, CH_{Ar}), 7.30 – 7.21 (dd, 2H, *J* = 8.5, 7.3 Hz, 2 x CH_{Ar}), 7.01 (t, 1H, CH_{Ar}, *J* = 7.29 Hz), 6.78 – 6.33 (m, 1H, N*H*), 3.79 – 3.68 (t, *J* = 6.0 Hz, 2H, CH_2), 3.61 – 3.40 (m, 2H), 3.34 – 3.23 (m, 4H), 3.21 – 3.12 (m, 6H), 3.11 – 3.01 (dd, *J* = 6.8, 6.7 Hz, 2H, CH_2), 2.97 – 2.74 (m, 2H, CH_2), 1.66 – 1.47 (m, 4H, 2 x CH_2), 1.37 – 1.24 (m, 6H, 3 x CH_2), 1.20 – 1.03 (m, 6H, 3 x CH_2), 0.88 – 0.75 (m, 9H, 3 x CH_3). **^{13}C NMR** (101 MHz, CD_2Cl_2) δ 168.1 (2 x C=O), 159.9 (C=O), 157.1 (C=O), 156.6 (C=O), 142.4 (C_{Ar}), 140.4 (C_{Ar}), 137.1 (2C, 2 x *C*S), 132.2 (q, *J* = 33.0 Hz, 2C, 2 x $C_{Ar}CF_3$), 129.3 (2C, 2 x CH_{Ar}), 124.2 (q, *J* = 272.8 Hz, 2C, 2 x CF_3), 120.5 (2C, 2 x CH_{Ar}), 118.7 (2C, 2 x CH_{Ar}), 115.3 (CH_{Ar}), 48.1 (CH_2), 47.1 (CH_2), 46.3 (CH_2), 44.3 (CH_2), 41.6 (CH_2), 36.7 (CH_2), 33.0 (2C, 2 x CH_2), 32.2 (2C, 2 x CH_2), 32.0 (CH_2), 30.5 (CH_2), 30.3 (CH_2), 27.1 (CH_2), 23.0 (CH_2), 22.2 (2C, 2 x CH_2), 14.3 (CH_3), 13.8 (2C, 2 x CH_3). **FTIR (neat)** νmax = 3308, 2958, 2928, 2855, 1705, 1632, 1554, 1388, 1277, 1134 cm^{-1}. **HRMS** (ESI, positive ion mode) – *m/z* for $[C_{41}H_{55}F_6N_7O_5S_2+H]^+$ = 904.3683, observed 904.3676.

3.3. Synthesis of ureas 9 and 12

Compound s36: *N*-(benzyl)-*N*'-(2,3-b*is*(butylsulfanyl)maleimidoethyl)-*N*,N''-b*is*(hexylcarbamoyl)diethylenetriamine

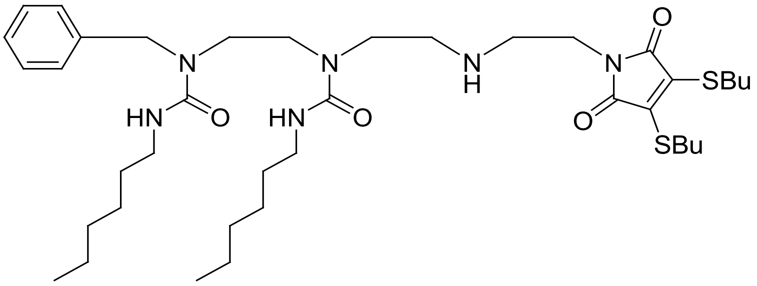

To *N,N''*-b*is*(hexylcarbamoyl)-*N*-benzyldiethylenetriamine, trifluoroacetic salt **s17** (42 mg, 0.094 mmol) in dry dichloromethane (2 mL) was added 2,3-b*is*(butylsulfanyl)maleimide-*N*-acetaldehyde **s27** (33 mg, 0.1 mmol) and the mixture was stirred in the dark under nitrogen atmosphere for 6 hours. Sodium triacetoxyborohydride (28 mg, 0.13 mmol) was added and the mixture was stirred at 40 °C for 12 hours. Saturared aqueous sodium hydrogen carbonate solution (10 mL) was added, the mixture was stirred for 30 minutes then extracted with dichrloromethane (2 x 20 mL), the organic phases were dried ($MgSO_4$), concentrated under reduced pressure, the residue was purified by chromatography on silica (eluent: dichloromethane:methanol 85:15) to afford the product **s36** as a yellow oil (39 mg, 0.052 mmol, 55%). **^{1}H NMR** (400 MHz, $CDCl_3$) δ 7.25 (t, *J* = 7.3 Hz, 2H, 2 x CH_{Ph}), 7.22 – 7.08 (m, 3H, 3 x CH_{Ph}), 6.28 (s, 1H, N*H*), 5.49 (s, 1H, N*H*), 4.42 (s, 2H, CH_2Ph), 3.56 (t, *J* = 6.0 Hz, 2H, CH_2N), 3.26 (m, 2H, CH_2N), 3.23 – 3.18 (m, 4H, 2 x CH_2), 3.18 – 3.10 (m, 7H, N*H*, 3 x CH_2), 3.09 – 3.05 (m, 2H, CH_2), 2.78 (t, *J* = 6.0 Hz, 2H, CH_2N), 2.67 (t, *J* = 5.7 Hz, 2H, CH_2N), 1.61 – 1.50 (m, 4H, 2 x CH_2), 1.47 – 1.31 (m, 8H, 4 x CH_2), 1.26 – 1.14 (m, 12H, 6 x CH_2), 0.89 – 0.83 (m, 6H, 2 x CH_3), 0.82 – 0.76 (m, 6H, 2 x CH_3). **^{13}C NMR** (101 MHz, $CDCl_3$) δ 166.9 (2C, 2 x *C*=O), 159.6 (*C*=O), 158.7 (*C*=O), 138.4 (2C, 2 x *C*S),

135.9 (C_{Ph}), 128.8 (2C, 2 x CH_{Ph}), 127.5 (CH_{Ph}), 127.3 (2C, 2 x CH_{Ph}), 51.4 (CH_2), 49.2 (CH_2), 48.2 (CH_2), 47.7 (CH_2), 46.9 (CH_2), 46.3 (CH_2), 41.2 (CH_2), 41.0 (CH_2), 37.6 (CH_2), 32.5 (2C, 2 x CH_2), 31.7 (CH_2), 31.7 (3C, 3 x CH_2), 30.3 (CH_2), 30.1 (CH_2), 26.9 (CH_2), 26.7 (CH_2), 22.8 (CH_2), 22.7 (CH_2), 21.8 (2C, 2 x CH_2), 14.2 (CH_3), 14.2 (CH_3), 14.1 (CH_3), 13.7 (CH_3). **FTIR (neat)** νmax = 3294, 2956, 2927, 2857, 1703, 1627, 1538, 1485, 1396, 1263, 1193, 736, 678 cm^{-1}. **HRMS** (ESI, positive ion mode) – *m/z* for $[C_{39}H_{66}N_6O_4S_2+H]^+$ 747.4660, observed 747.4627.

Compound 12: *N*-(benzyl)-*N*'-(2,3-b*is*(butylsulfanyl)maleimidoethyl)-*N*,N',N''-tri(hexylcarbamoyl)diethylenetriamine

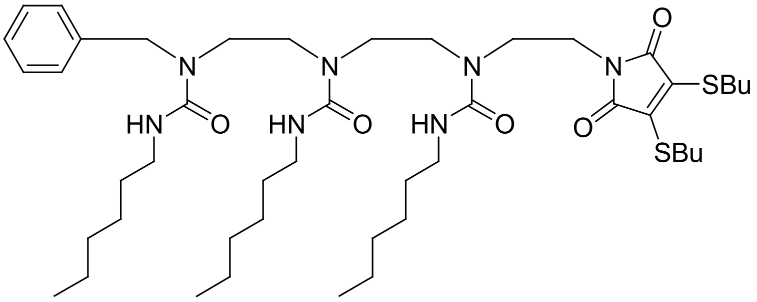

To *N*-(benzyl)-*N*'-(2,3-b*is*(butylsulfanyl)maleimidoethyl)-*N*,*N*''-b*is*(hexylcarbamoyl)diethylenetriamine **S36** (40 mg, 0.054 mmol) in dry dichloromethane (2 mL) was added hexylisocyanate (0.013 mL, 0.086 mmol) and the mixture was stirred under inert atmosphere in the dark at 20 °C for 18 hours. The resulting mixture was concentrated under reduced pressure and the residue was purified by chromatography on silica (eluent CH_2Cl_2:MeOH 95:5, Rf = 0.5) to afford the product as a yellow oil (47 mg, 0.054 mmol, quantitative). **^{1}H NMR** (400 MHz, $CDCl_3$) δ 7.28 (t, *J* = 7.3 Hz, 2H, 2 x CH_{Ph}), 7.23 – 7.14 (m, 3H, 3 x CH_{Ph}), 6.39 (t, *J* = 5.3 Hz, 1H, N*H*CH_2), 5.74 – 4.95 (bm, 2H, 2 x NH), 4.41 (s, 2H, CH_2Ph), 3.52 (t, *J* = 7.0 Hz, 2H, CH_2), 3.31 - 3.24 (m, 2H, CH_2), 3.24 – 3.17 (m, 6H, 3 x CH_2), 3.16 – 3.04 (m, 12H, 6 x CH_2), 1.55 (q, *J* = 7.5 Hz, 4H, 2 x CH_2), 1.49 – 1.41 (m, 4H, 2 x CH_2), 1.41 – 1.31 (m, 8H, 4 x CH_2), 1.29 – 1.20 (m, 8H, 4 x CH_2), 1.20 – 1.11 (m, 8 H, 4 x CH_2), 0.86 (t, *J* = 7.3 Hz, 6H, 2 x CH_3), 0.83 – 0.74 (m, 9H, 3 x CH_3). **^{13}C NMR** (101 MHz, $CDCl_3$) δ 166.6 (2C, 2 x *C*=O), 158.6 (2C, 2 x *C*=O), 157.9 (*C*=O), 137.9 (2C, 2 x *C*S), 136.1 (2C, 2 x C_{Ph}), 129.5 (2C, 2 x CH_{Ph}), 127.6 (CH_{Ph}), 127.0 (2C, 2 x CH_{Ph}), 51.8 (CH_2Ph), 47.2 (CH_2), 47.1 (CH_2), 47.0 (CH_2), 46.0 (CH_2), 41.1 (CH_2), 41.0 (CH_2), 41.0 (CH_2), 36.7 (CH_2N), 32.5 (2C, 2 x CH_2), 31.6 – 31.5 (6 CH_2), 30.0 (CH_2), 30.0 (CH_2), 29.9 (CH_2), 26.7 (CH_2), 26.7 (CH_2), 26.5 (CH_2), 22.7 (CH_2), 22.6 (CH_2), 22.6 (CH_2), 21.7 (2C, 2 x CH_2), 14.1 (CH_3), 14.1 (CH_3), 14.0 (CH_3), 13.6 (2C, 2 x CH_3). **HRMS** (ESI, positive ion mode) – *m/z* for $[C_{46}H_{79}N_7O_5S_2Na]^+$ 896.5476, observed 896.5449.

Compound 9: *N,N'*-diethyl- *N,N'*-b*is*(butylcarbamoyl)ethylenediamine

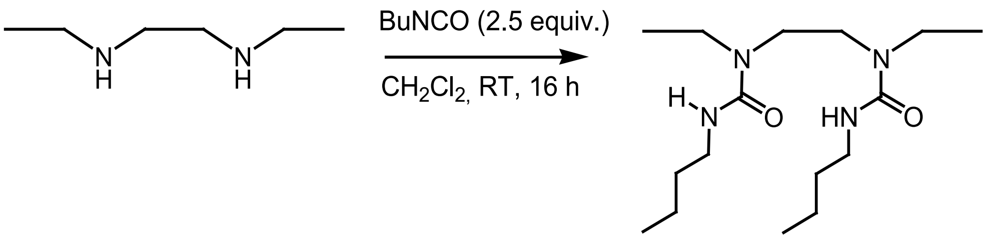

To a 0.02 M solution of *N,N'*-di(ethyl)ethylenediamine (100 μL, 0.69 mmol) in anhydrous CH_2Cl_2 was added butyl isocyanate (195 μL, 1.72 mmol, 2.5 equiv.), the mixture was stirred at 20 °C for 16 hours. Water was added, the resulting mixture was extracted with CH_2Cl_2, the organic layer was dried over $MgSO_4$, filtered and concentrated under reduced pressure. The residue was purified using flash column chromatography on silica (eluent CH_2Cl_2:MeOH 97:3) to provide

the title compound as a white solid (134 mg, 0.44 mmol, 64%). **^{1}H NMR** (400 MHz, $CDCl_3$) δ 5.19 (s, 2H, 2 x NH), 3.28 (s, 4H), 3.27 – 3.18 (m, 8H), 1.56 – 1.44 (m, 4H), 1.43 – 1.29 (m, 4H), 1.13 (t, *J* = 7.1 Hz, 6H), 0.92 (t, *J* = 7.3 Hz, 6H). **^{1}H NMR** (400 MHz, CD_2Cl_2) δ 5.43 (s, 2H, 2 x NH), 3.15 (m, 8H, 2 x C*H*$_2$N, 2 x C*H*$_{2\,Et}$), 3.13 – 3.06 (m, 4H, 2 x C*H*$_{2Bu}$), 1.48 – 1.33 (m, 4H), 1.27 (dt, *J* = 7.2 Hz, 4H, 2 x C*H*$_2$), 1.01 (t, *J* = 7.1 Hz, 6H, 2 x C*H*$_{3cEt}$), 0.84 (t, *J* = 7.3 Hz, 6H, 2 x C*H*$_{3Bu}$). **^{13}C NMR** (101 MHz, CD_2Cl_2) δ 158.5 (C=O), 47.0 (2C, 2 x *C*H$_2$), 43.3 (2C, 2 x *C*H$_2$), 41.1 (2C, 2 x *C*H$_2$), 33.0 (2C, 2 x *C*H$_2$), 20.7 (2C, 2 x *C*H$_2$), 14.5 (*C*H$_3$), 14.2 (*C*H$_3$). **FTIR (neat)** νmax = 3344, 2954, 2927, 2872, 1623, 1533, 1406, 1273 cm^{-1}. **HRMS** (ESI, positive ion mode) – *m/z* for $[C_{18}H_{36}N_2O_2+H]^+$ 315.2754, observed 315.2765. **MP** 156-158 °C.

3.4. Synthesis of phosphate ligands

***bis*(4-*n*-butoxyphenyl) hydrogen phosphate**

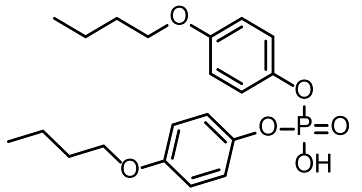

To a solution of 4-*n*-butoxyphenol (1 g, 6.02 mmol) and triethylamine (880 µL, 6.1 mmol) in toluene (30 mL) at 20 °C was added dropwise phosphoryl chloride (280 µL, 3 mmol, 0.5 equiv.). The mixture was stirred for 16 h, then water (10 mL) was added and the resulting mixture was stirred at 70 °C for 1 hour. The mixture was cooled down to 20 °C, then was extracted using CH_2Cl_2, and the organic layer was concentrated under reduced pressure. The residue was purified by silica gel flash chromatography to afford the acid as colourless oil (283 mg, 0.72 mmol, 12%). **^{1}H NMR** (400 MHz, $CDCl_3$) δ 11.77 (s, 1H, O*H*), 7.05 (d, *J* = 8.6 Hz, 4H, 4 x CH$_{Ar}$), 6.74 (d, *J* = 8.6 Hz, 4H, 4 x CH$_{Ar}$), 3.83 (m, 4H, 2 x C*H*$_2$), 1.71 (dt, *J* = 8.5 Hz, 6.6 Hz, 4H, 2 x C*H*$_2$), 1.60 – 1.33 (m, 4H, 2 x C*H*$_2$), 0.96 (t, *J* = 7.6, 6Hz , 2 x C*H*$_3$). **^{13}C NMR** (101 MHz, $CDCl_3$) δ 156.6 (2C, 2 x C$_{Ar}$), 144.1 (C$_{Ar}$), 144.0 (*C*$_{Ar}$), 121.2 (2C, 2 x *C*H$_{Ar}$), 121.1 (2C, 2 x *C*H$_{Ar}$), 115.3 (4C, 4 x *C*H$_{Ar}$), 68.2 (*C*H$_2$), 31.4 (*C*H$_2$), 19.3 (*C*H$_2$), 14.0 (*C*H$_3$). **FTIR (neat)** νmax = 2958, 2933, 2872, 1500, 1183 cm^{-1}. **HRMS** (nanospray TOF, negative ion mode) – *m/z* for $[C_{20}H_{27}O_6P - H]^-$ 393.1467, observed 393.1469.

Tetrabutylammonium diphenyl phosphate

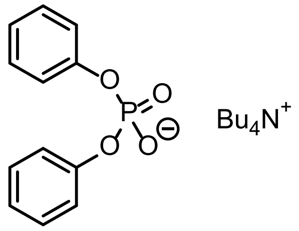

To a solution of phenyl hydrogen phosphate (200 mg, 0.8 mmol) in a 1:1 water:EtOH mixture (6 mL) was added a solution of tetrabutylammonium hydroxide (523 µL, 0.8 mmol) in water, the mixture was stirred at 20 °C for an hour before concentration under reduced pressure. The resulting tetrabutylammonium phosphate salt was dried under high vacuum for 8 hours (quantitative yield). **^{1}H NMR** (400 MHz, $CDCl_3$) δ 7.36 – 7.29 (m, 4H, 4 x C*H*$_{Ar}$), 7.20 (t, *J* = 7.8 Hz, 4H, 4 x C*H*$_{Ar}$), 6.95 (t, *J* = 7.3 Hz, 2H, 2 x C*H*$_{Ar}$), 3.36 – 3.27 (m, 8H, 4 x CH$_2$), 1.69 – 1.57 (m, 8H, 4 x CH$_2$), 1.42 (dt, *J* = 7.4 Hz, 8H, 4 x CH$_2$), 0.99 (t, *J* = 7.3 Hz, 12H, 4 x CH$_3$). **^{13}C NMR** (101 MHz, $CDCl_3$) δ 154.3 (C$_{Ar}$), 154.3 (C$_{Ar}$), 128.8 (4C, 4 x CH$_{Ar}$), 121.9 (2C, 2 x CH$_{Ar}$), 120.4 (2C, 2 x CH$_{Ar}$), 120.4 (2C, 2 x CH$_{Ar}$), 58.8 (4C, 4 x *C*H$_2$N), 24.1 (4C, 4 x *C*H$_2$), 19.8 (4C, 4 x *C*H$_2$), 13.8 (4C, 4 x *C*H$_3$).

Tetrabutylammonium di(*p*-butoxyphenyl)phosphate

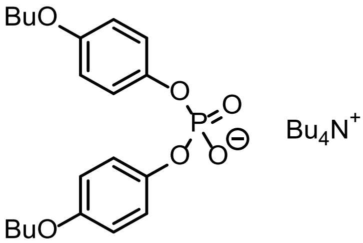

To a solution of *bis*(4-*n*-butoxyphenyl) hydrogen phosphate (163 mg, 0.42 mmol) in a 1:1 water:EtOH mixture (6 mL) was added a solution of tetrabutylammonium hydroxide (0.42 mmol) in water, the mixture was stirred at 20 °C for an hour before concentration under reduced pressure. The resulting tetrabutylammonium phosphate salt was dried under high vacuum for 8 hours (quantitative yield). **^{1}H NMR** (400 MHz, $CDCl_3$) δ 7.21 – 7.08 (m, 4H, 4 x CH_{Ar}), 6.80 – 6.52 (m, 4H, 4 x CH_{Ar}), 3.92 – 3.79 (t, *J* = 6.5 Hz, 4H, 2 x CH_2O), 3.38 – 3.17 (m, 8H, 4 x $CH_{2\ Bu4N}$), 1.77 – 1.64 (m, 4H, 2 x CH_2), 1.64 – 1.51 (m, 8H, 4 x CH_2), 1.51 – 1.29 (m, 12H, 6 x CH_2), 1.04 – 0.84 (m, 18H, 4 x $CH_{3\ Bu4N+}$, 2 x CH_3). **^{13}C NMR** (400 MHz, $CDCl_3$) δ 154.2 (2C, 2 x C_{Ar}), 148.2 (C_{Ar}), 148.1 (C_{Ar}), 121.1 (2C, 2 x CH_{Ar}), 121.1 (2C, 2 x CH_{Ar}), 114.9 (4C, 4 x CH_{Ar}), 68.3 (2C, 2 x CH_2), 59.0 (4C, 4 x CH_2), 31.6 (2C, 2 x CH_2), 24.2 (4C, 4 x CH_2), 19.9 (4C, 4 x CH_2), 19.4 (2C, 2 x CH_2), 14.0 (2C, 4 x CH_3), 13.8 (4C, 4 x $CH_{3\ Bu4N+}$).

4. NMR spectra

Compound s24: *N*'',*N*''',N''''-tri(hexylcarbamoyl)-*N*-benzyl-*N*-(*tert*-butylcarbamoyl)-*N*'-(*tert*-butoxycarbonyl)tetraethylenepentamine (in $CDCl_3$)

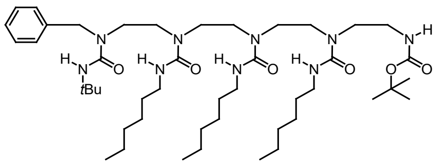

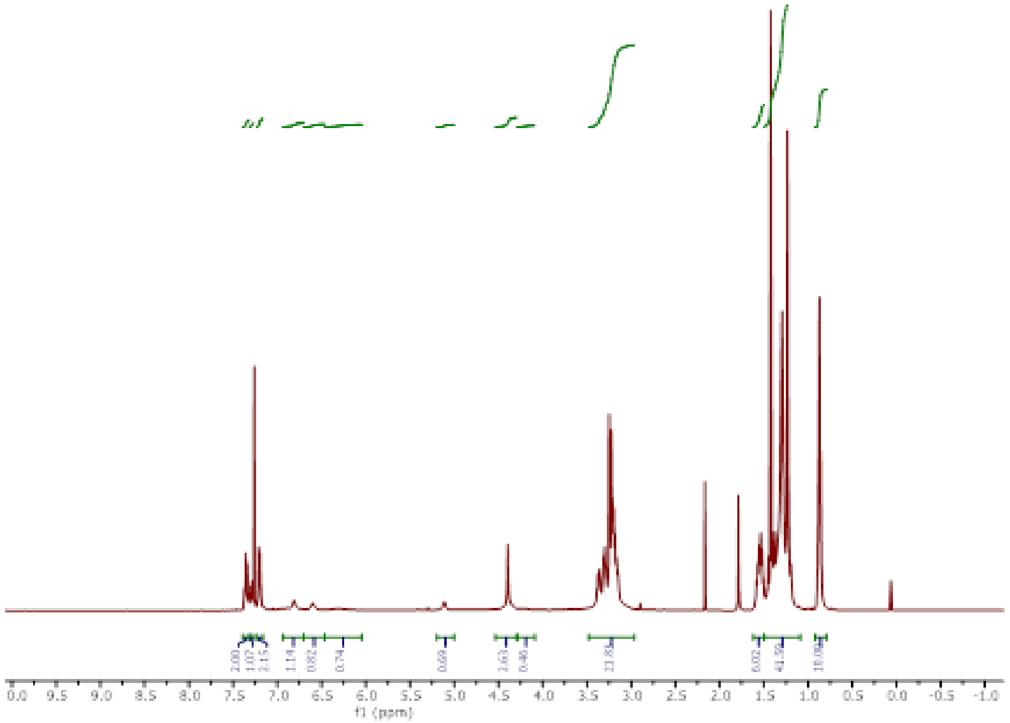

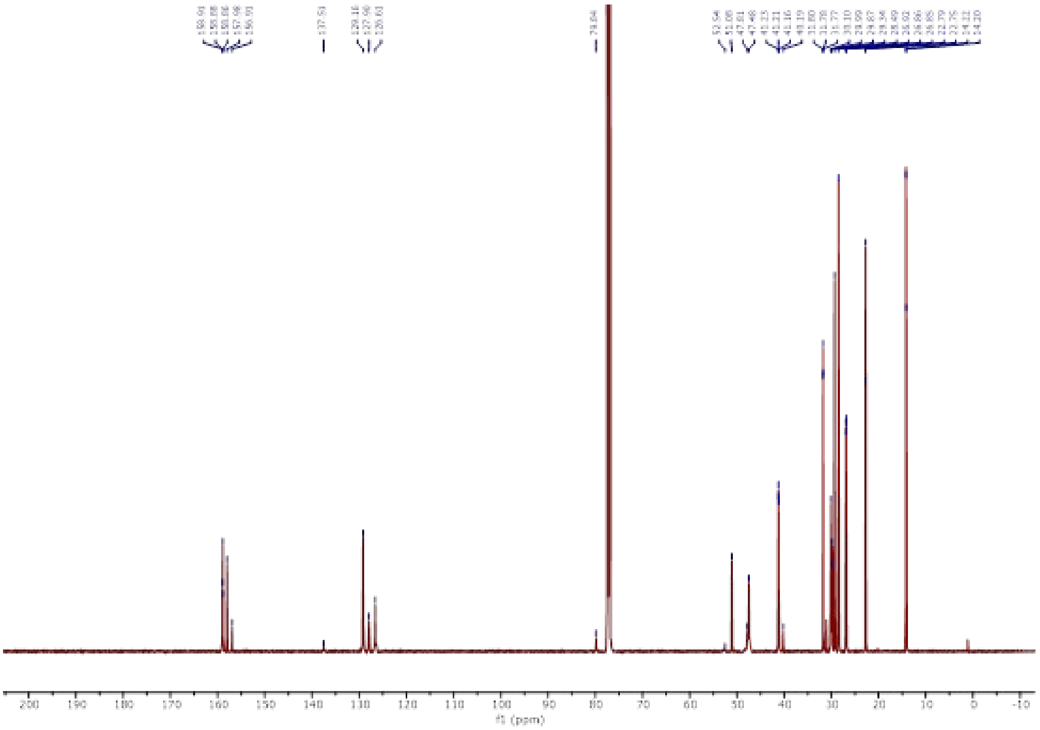

Compound s24 deprotected: *N*'',*N*''',*N*''''-Tri(hexylcarbamoyl)-*N*-benzyl-*N*-(*tert*-butylcarbamoyl)tetraethylenepentamine (in $CDCl_3$)

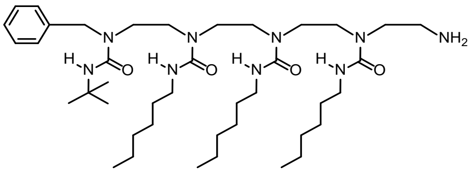

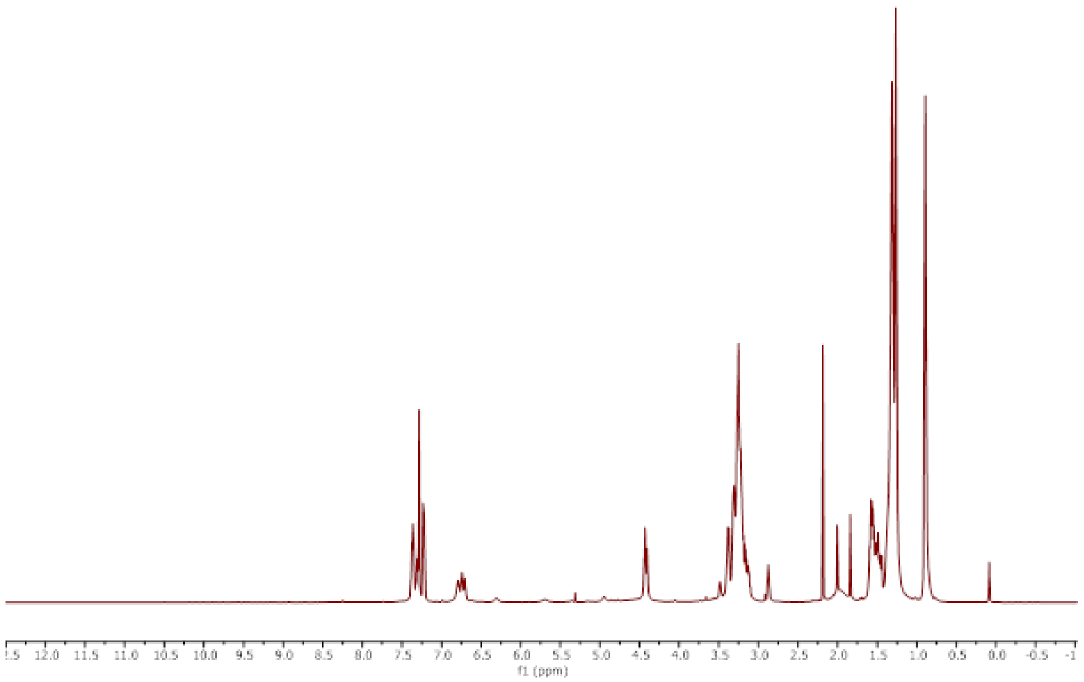

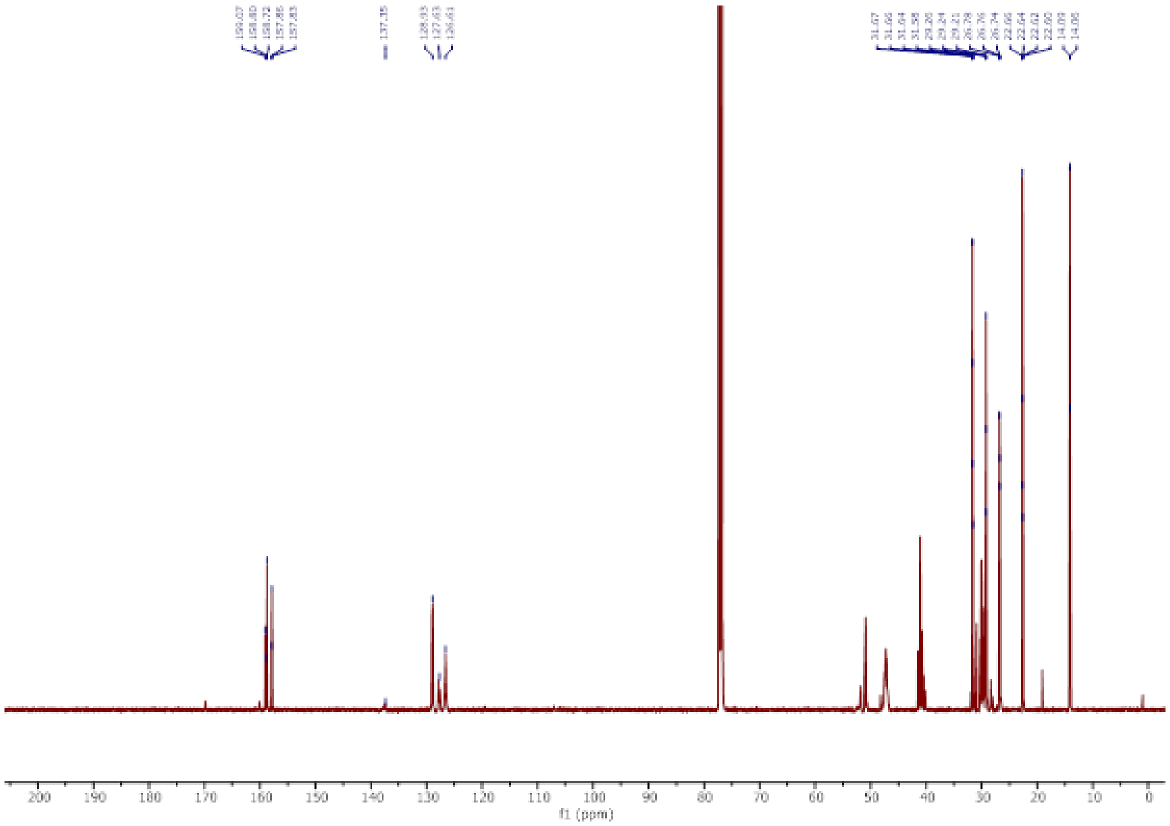

Compound s24 deprotected: *N''*,*N'''*,*N''''*-Tri(hexylcarbamoyl)-*N*-benzyl-*N*-(*tert*-butylcarbamoyl)tetraethylenepentamine (in $CDCl_3$)

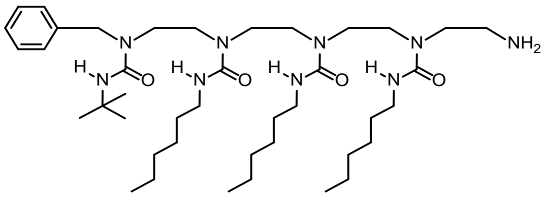

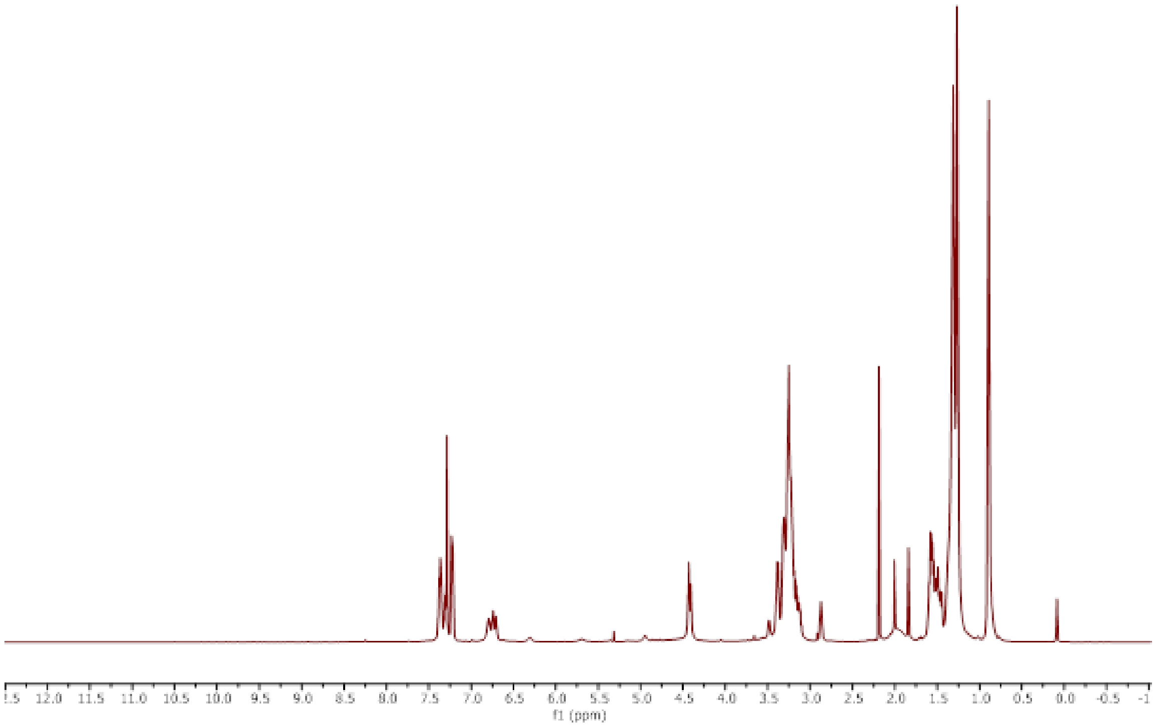

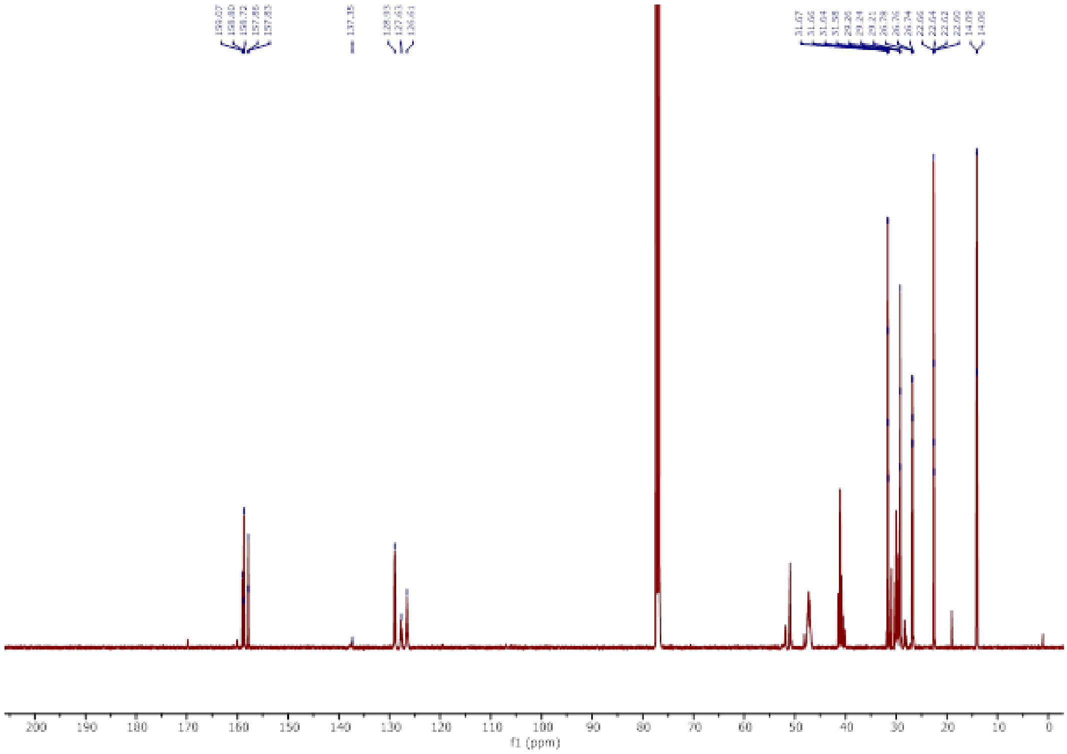

Compound 5: ***N*'',*N*''',N''''-tri(hexylcarbamoyl)-*N*-benzyl-*N*-(*tert*-butylcarbamoyl)-*N'*-(3,5-b*is*(trifluoromethyl)phenylcarbamoyl)tetraethylenepentamine (in $CDCl_3$)**

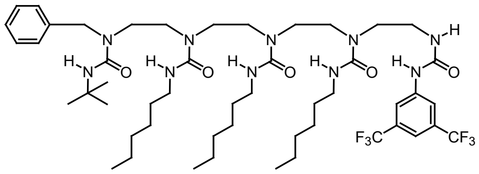

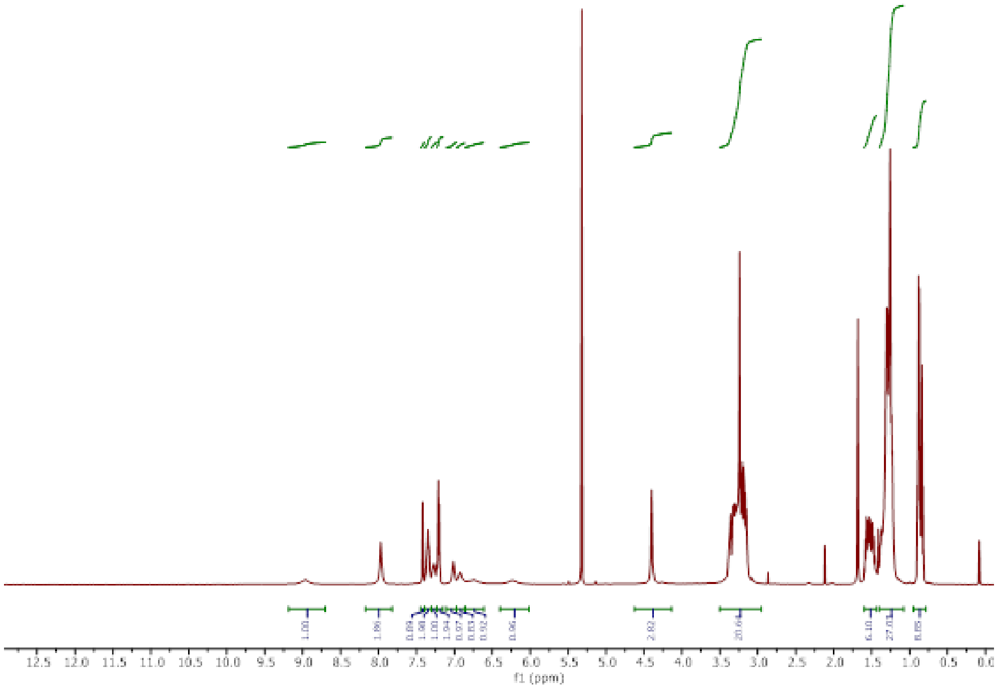

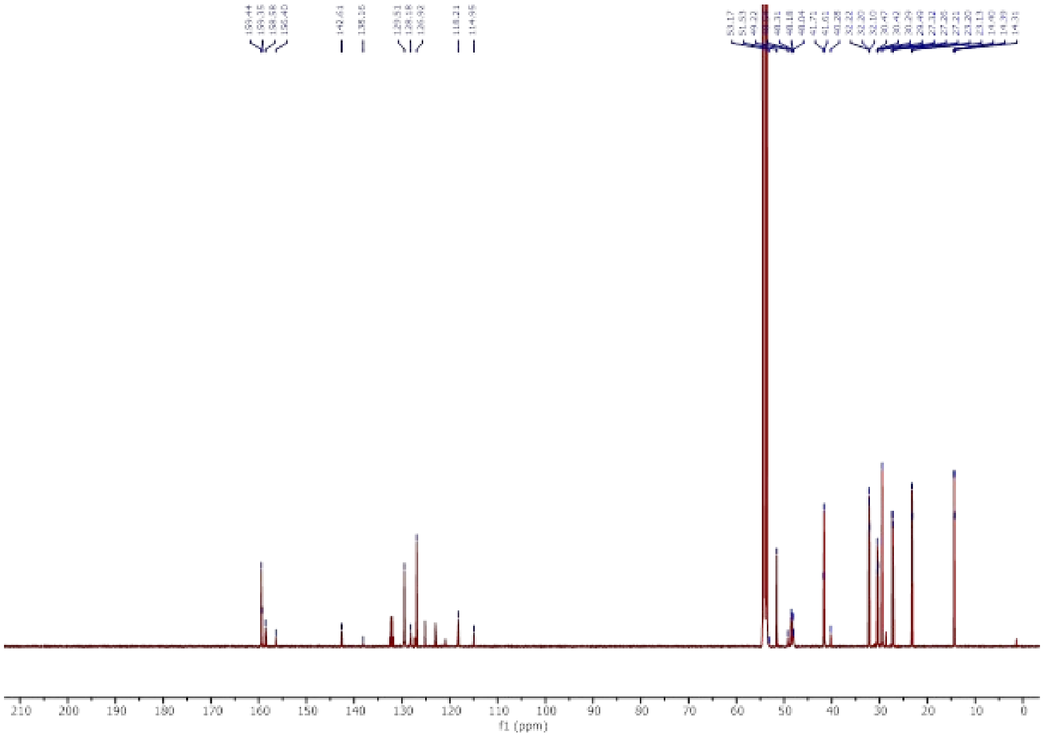

***bis*(4-*n*-butoxyphenyl) hydrogen phosphate (in CDCl$_3$)**

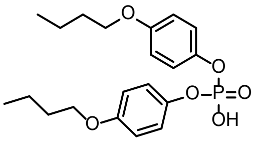

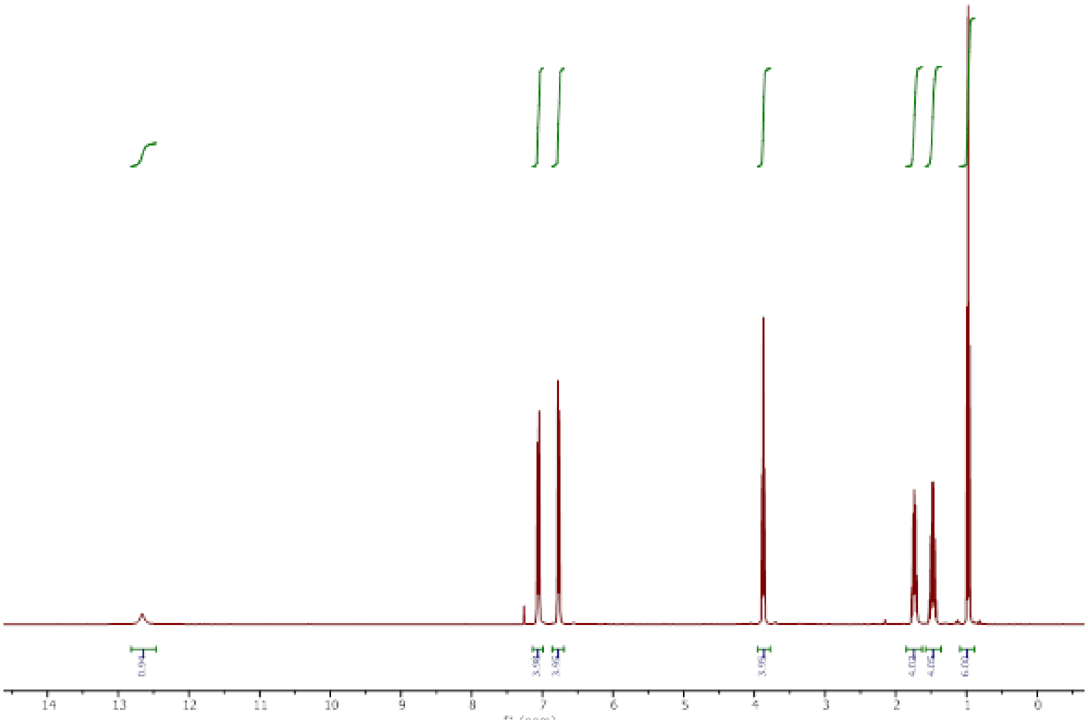

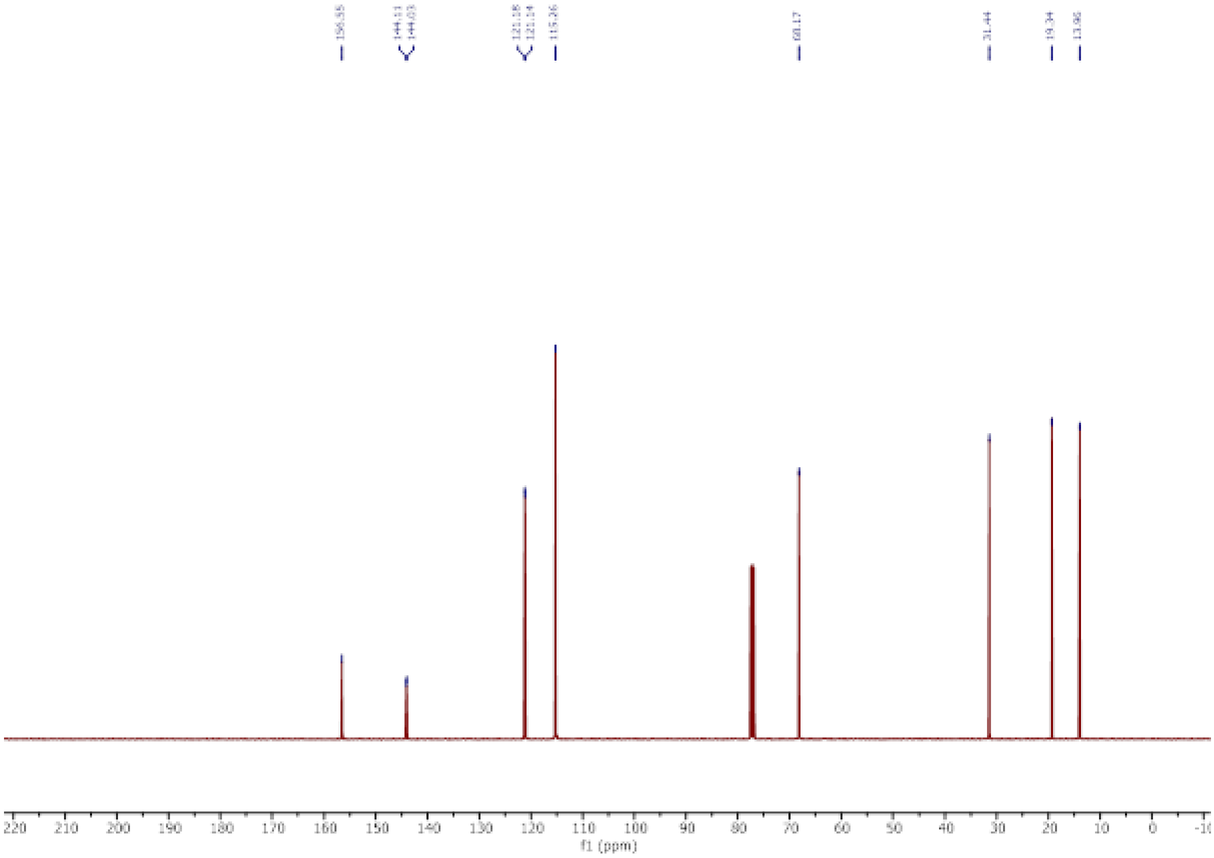

Tetrabutylammonium diphenyl phosphate (in CDCl₃)

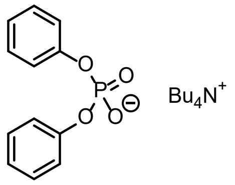

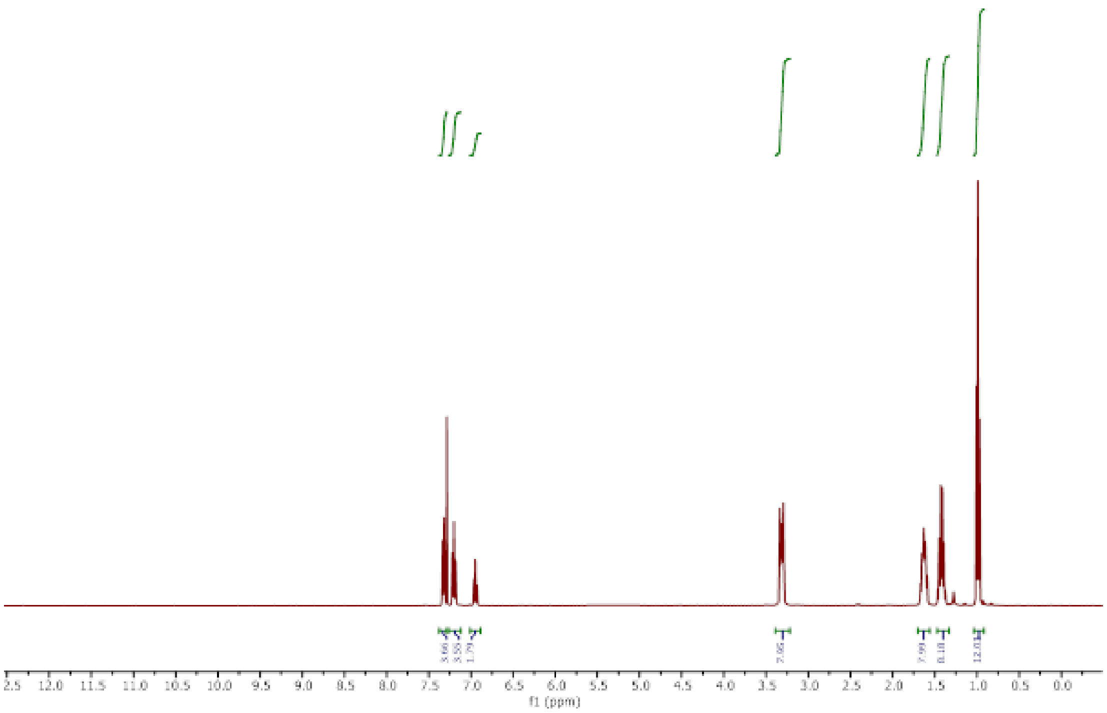

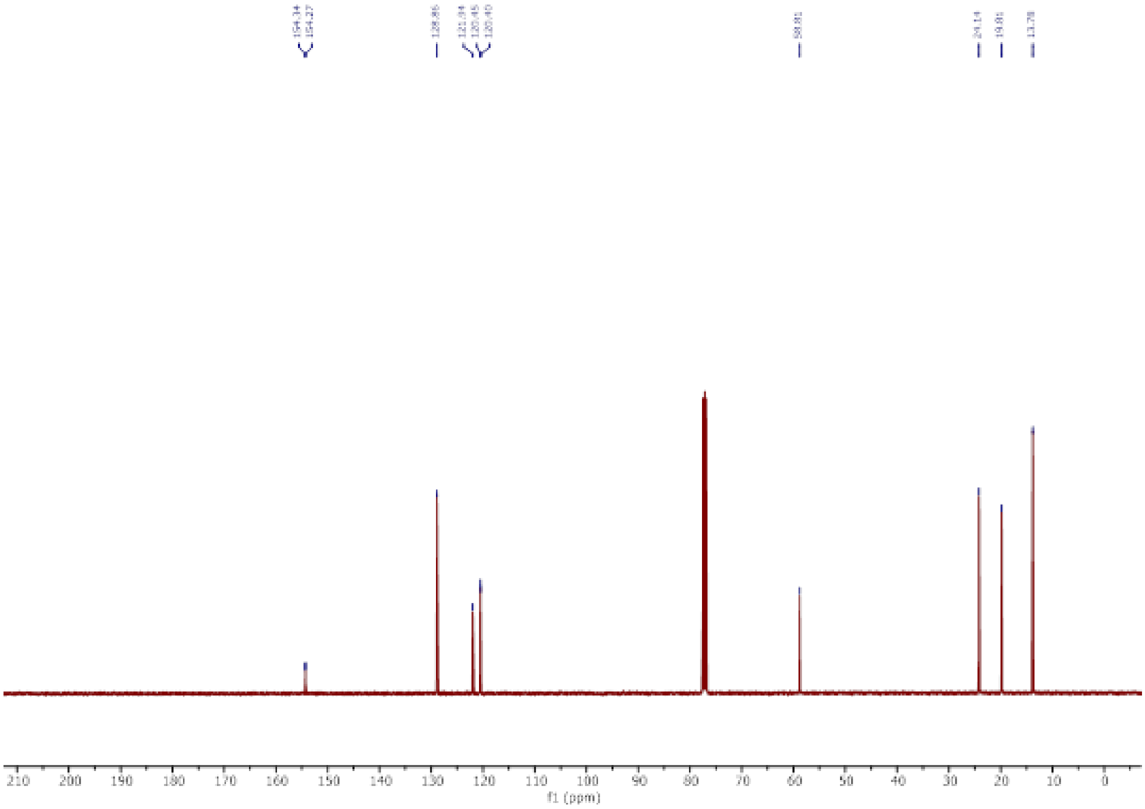

Tetrabutylammonium di(*p*-butoxyphenyl)phosphate (in $CDCl_3$)

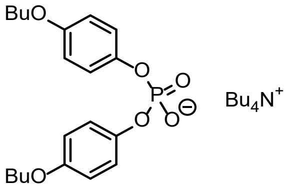

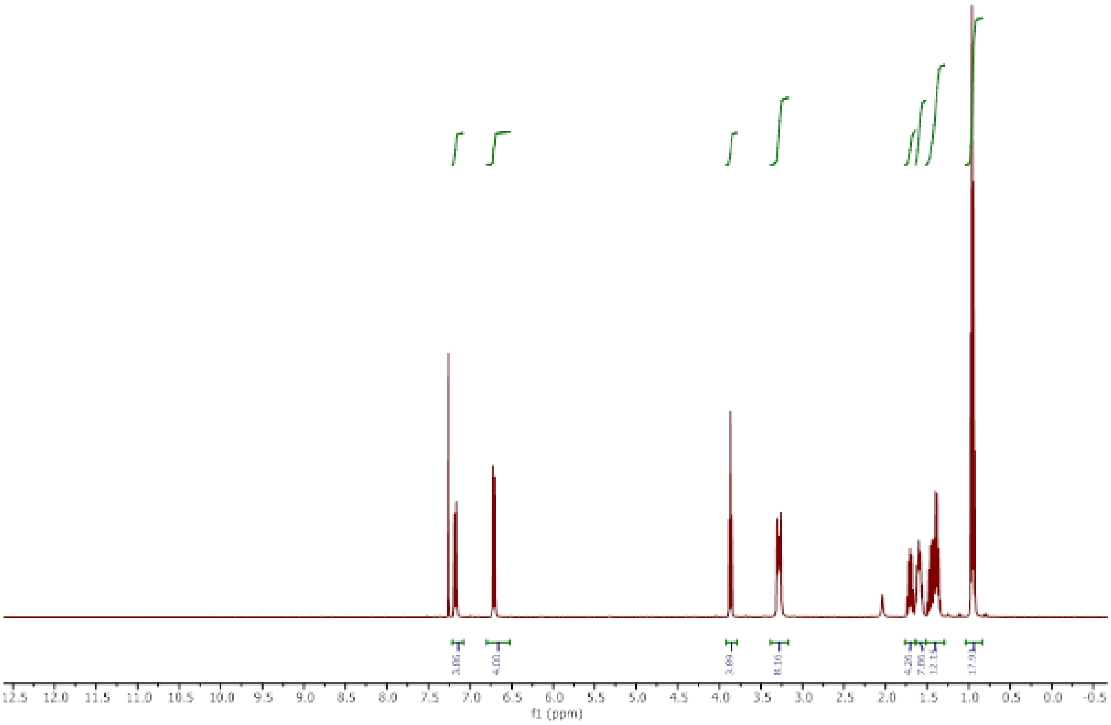

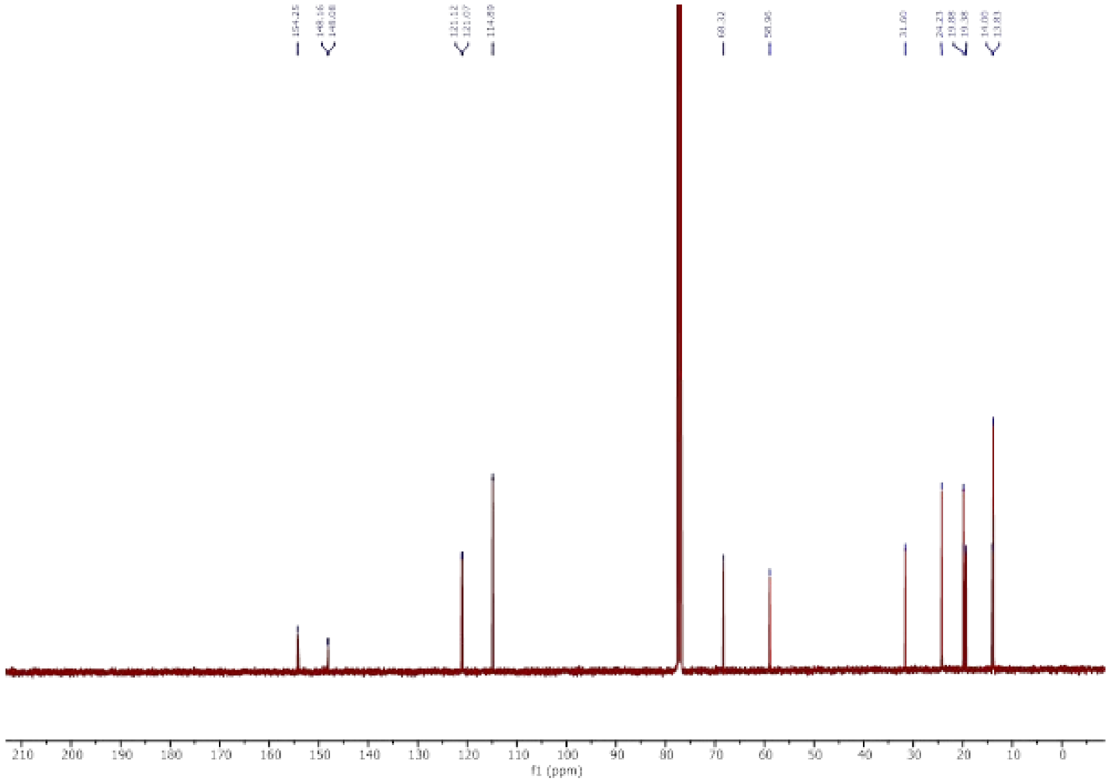

Compound s28: ***N,N'*-*bis*(trifluoroacetyl)-*N*''-(hexylcarbamoyl)diethylenetriamine** **(in $CDCl_3$)**

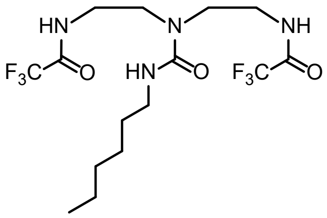

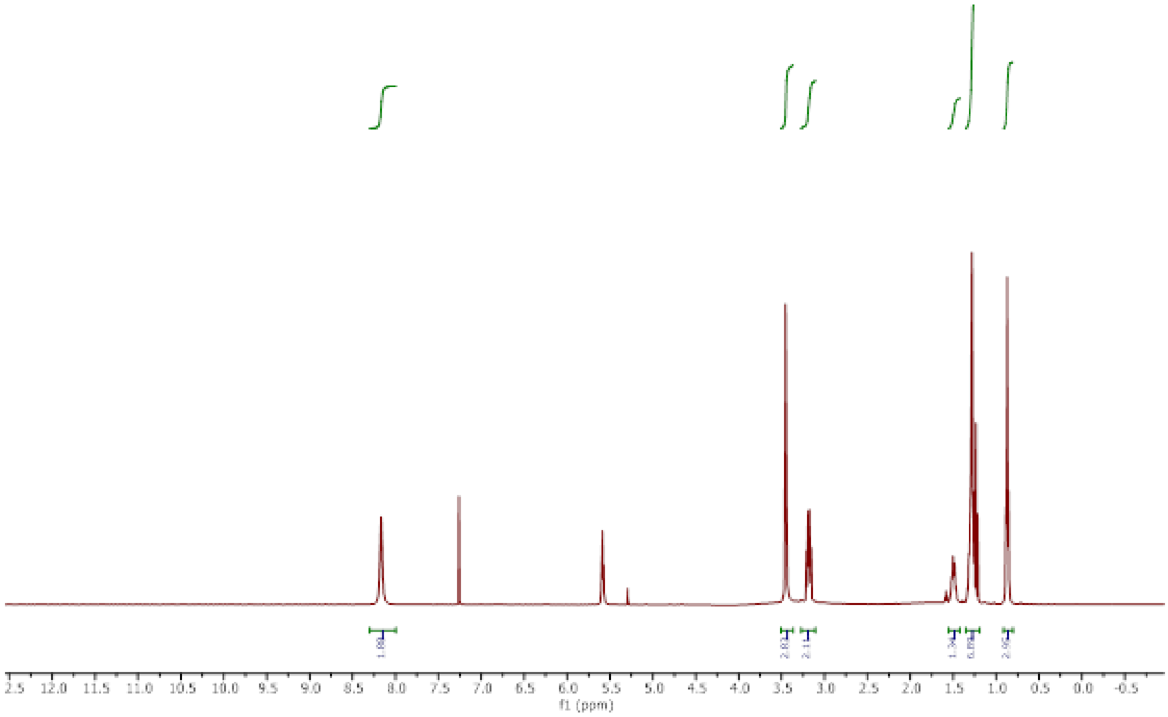

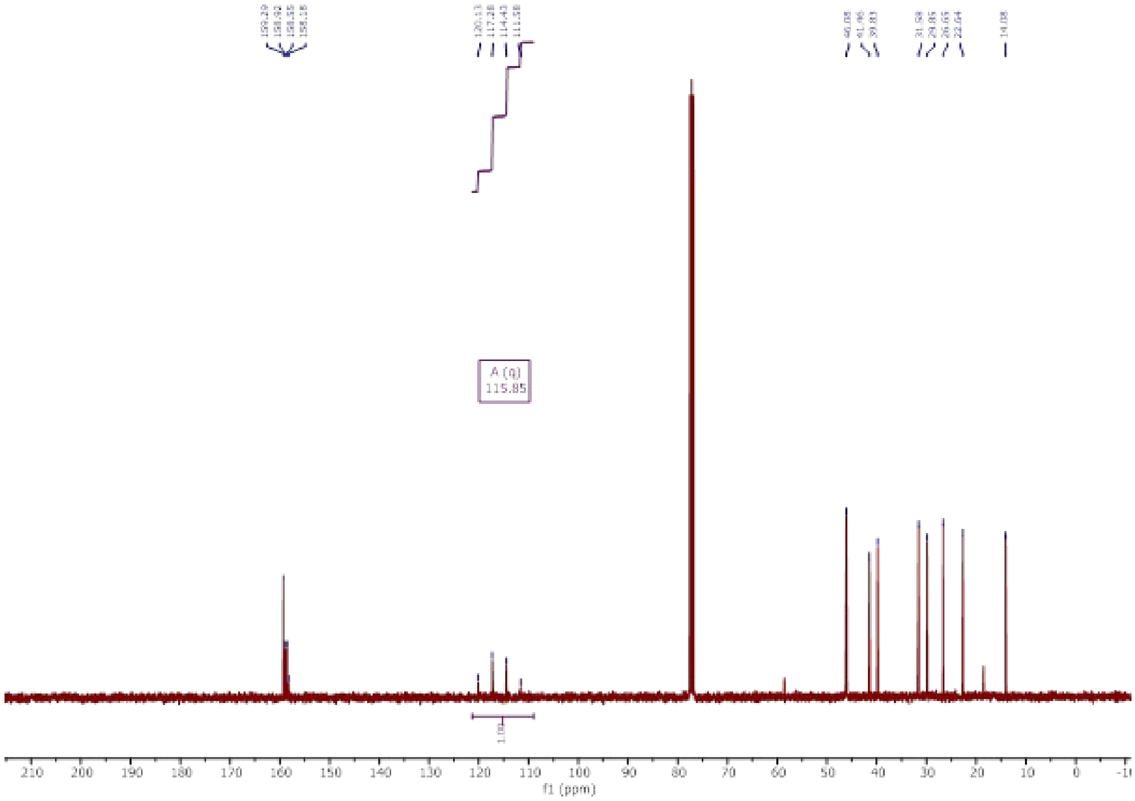

Compound s30: *N*''-(hexylcarbamoyl)diethylenetriamine (in $CDCl_3$)

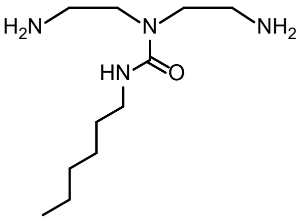

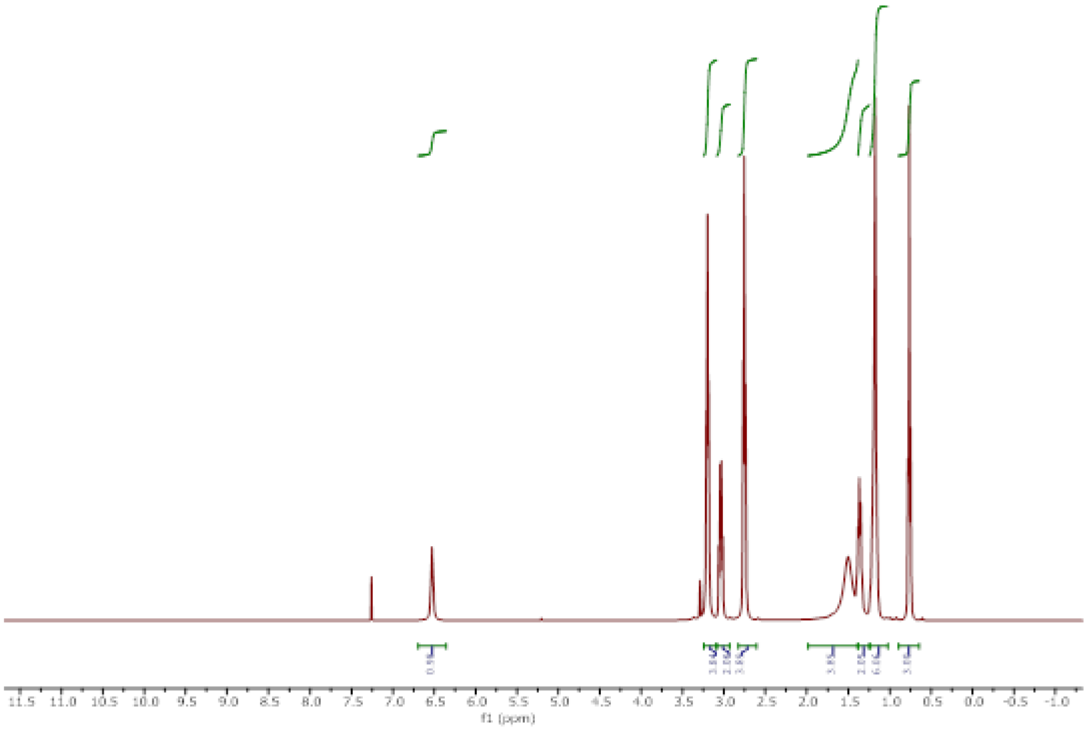

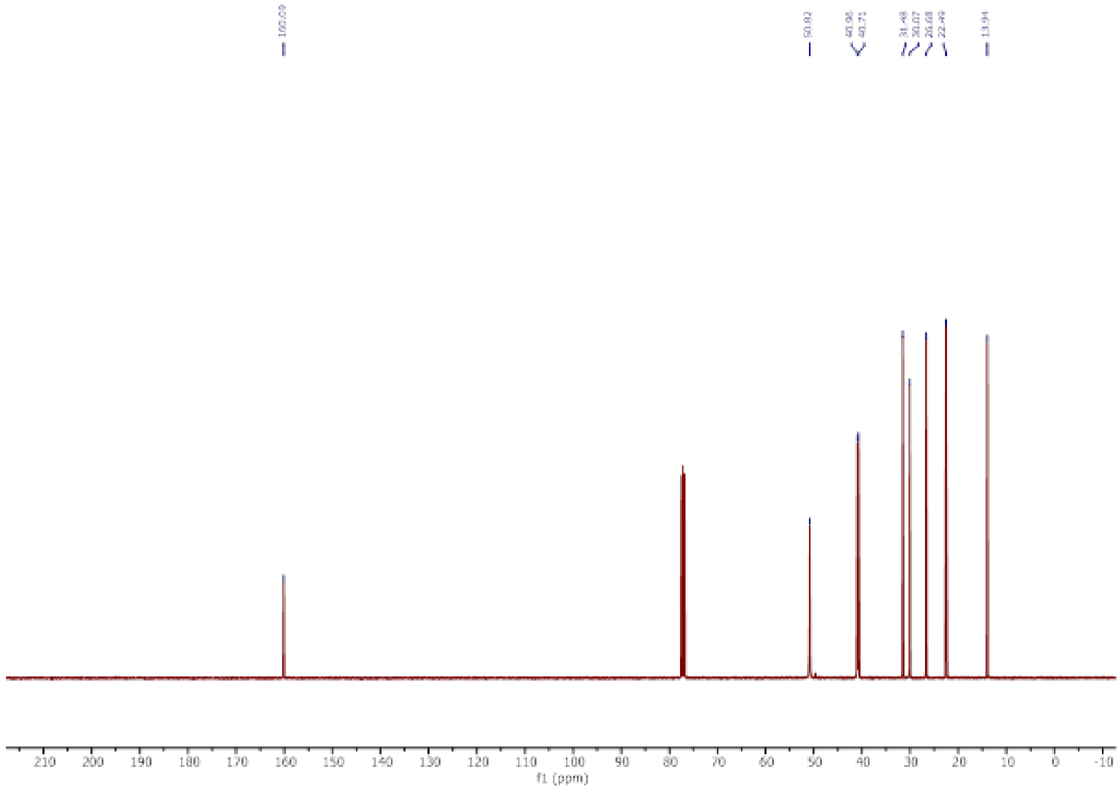

Compound s32: ***N*****-(trifluoroacetyl)- *N*'',*N*''',*N*''''-tri(butylcarbamoyl)tetraethylenepentamine (in $CDCl_3$)**

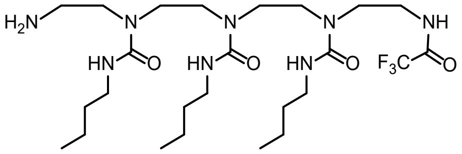

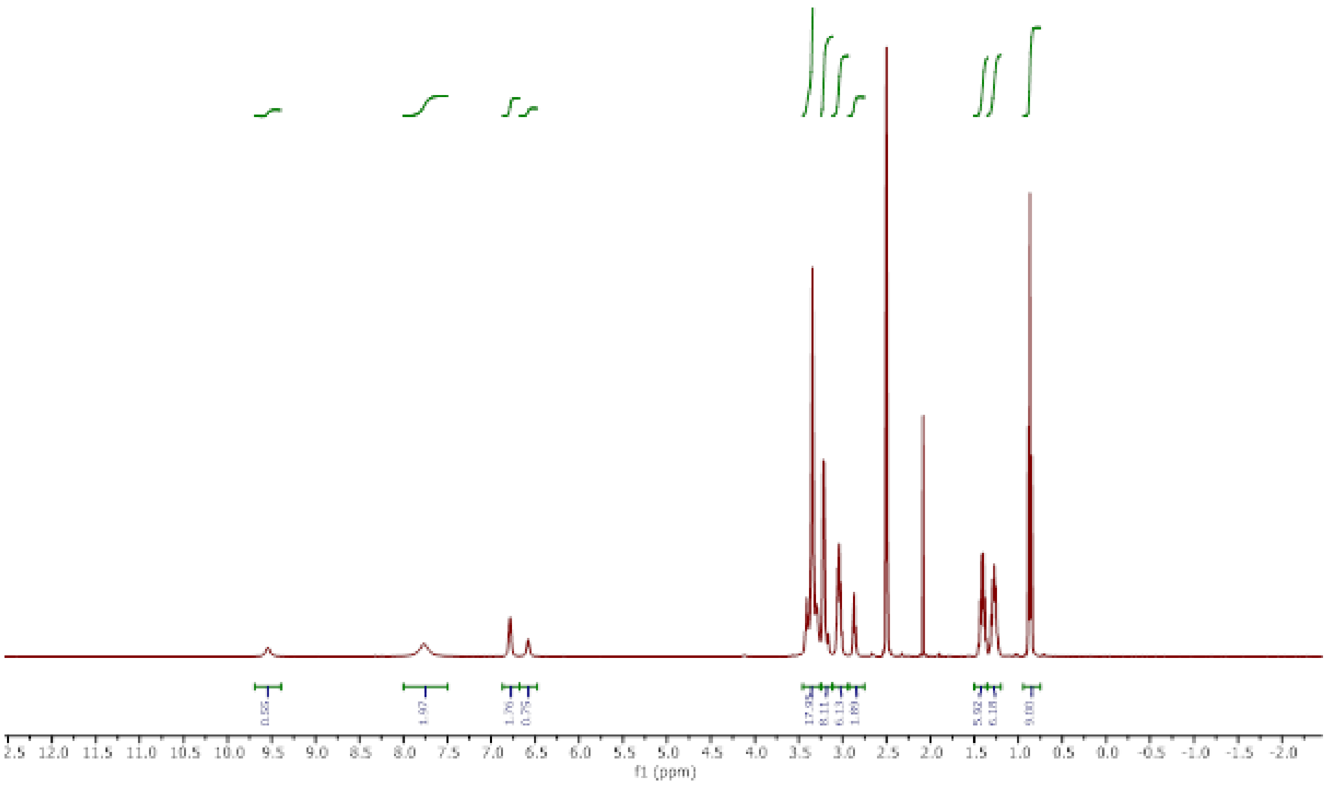

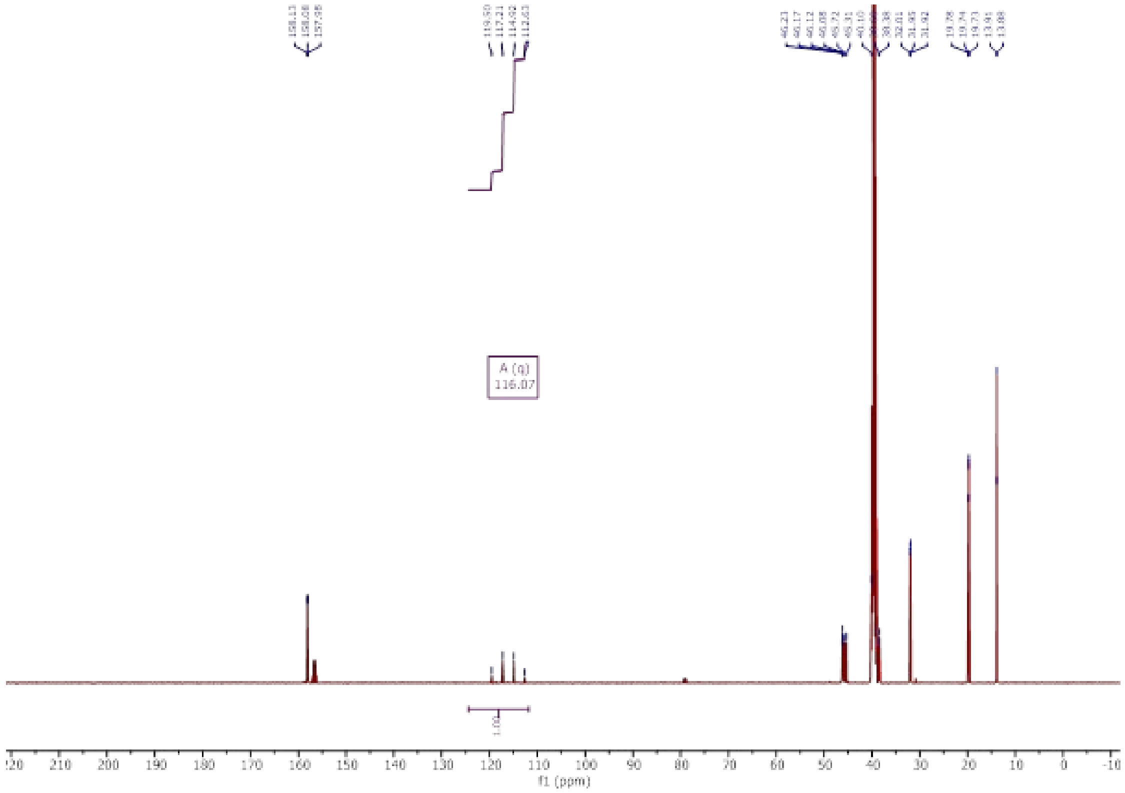

Compound s33: *N*-(3,5-b*is*(trifluoromethyl)phenylcarbamoyl)- *N*''-(hexylcarbamoyl)diethylenetriamine (in $CDCl_3$)

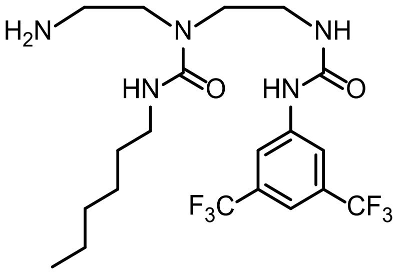

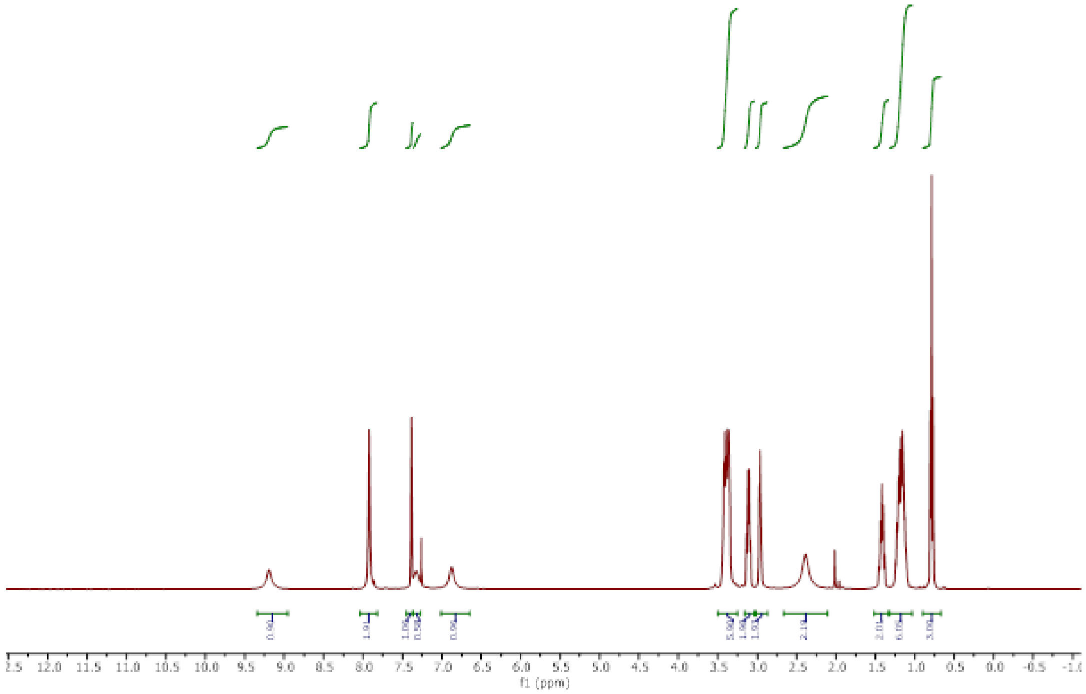

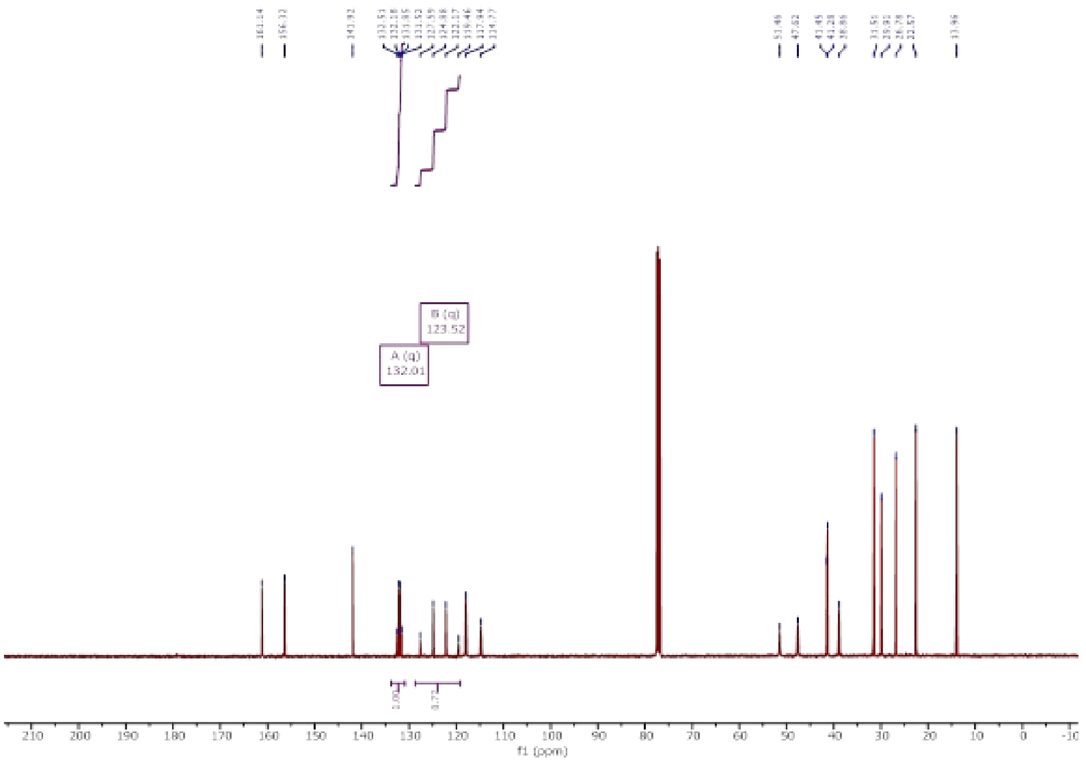

Compound s33di: ***N,N'*-b*is*(3,5-b*is*(trifluoromethyl)phenylcarbamoyl)-*N*''-(hexylcarbamoyl)diethylenetriamine (in $CDCl_3$)**

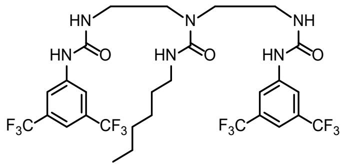

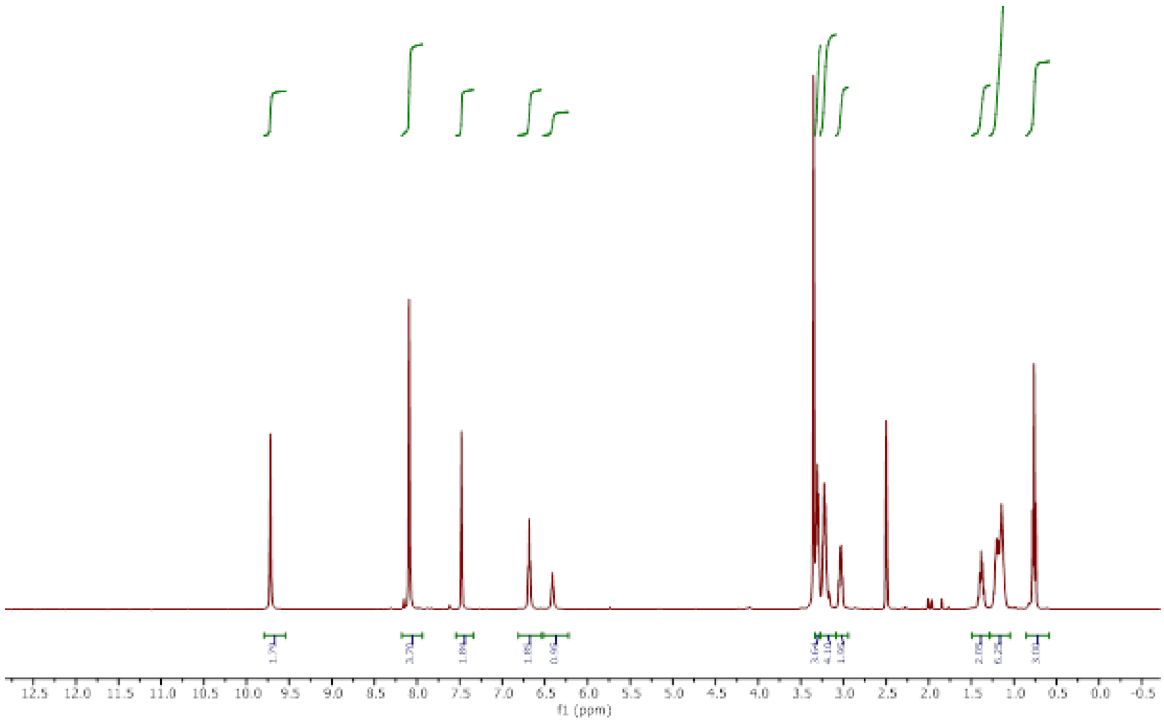

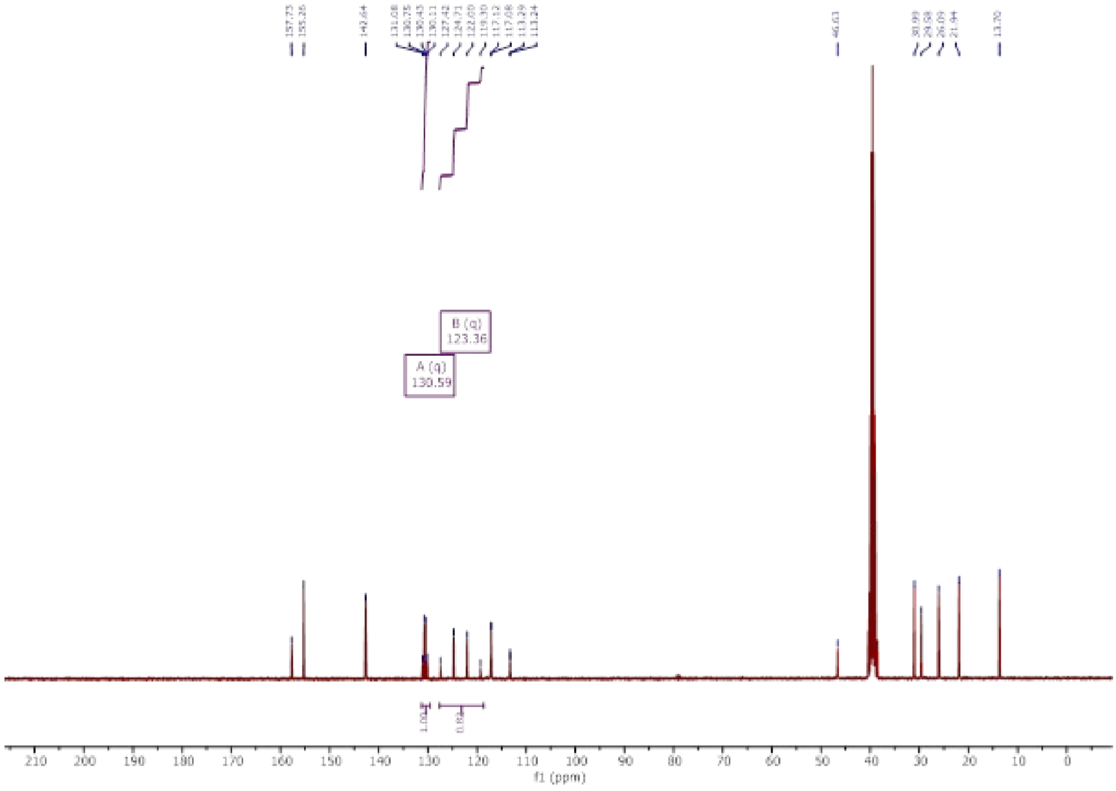

Compound s35: *N*-(3,5-b*is*(trifluoromethyl)phenylcarbamoyl)-*N*'-(2,3-b*is*(butylsulfanyl)maleimidoethyl)-*N*''-(hexylcarbamoyl)diethylenetriamine (in $CDCl_3$)

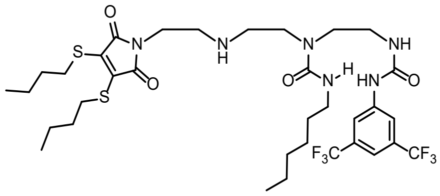

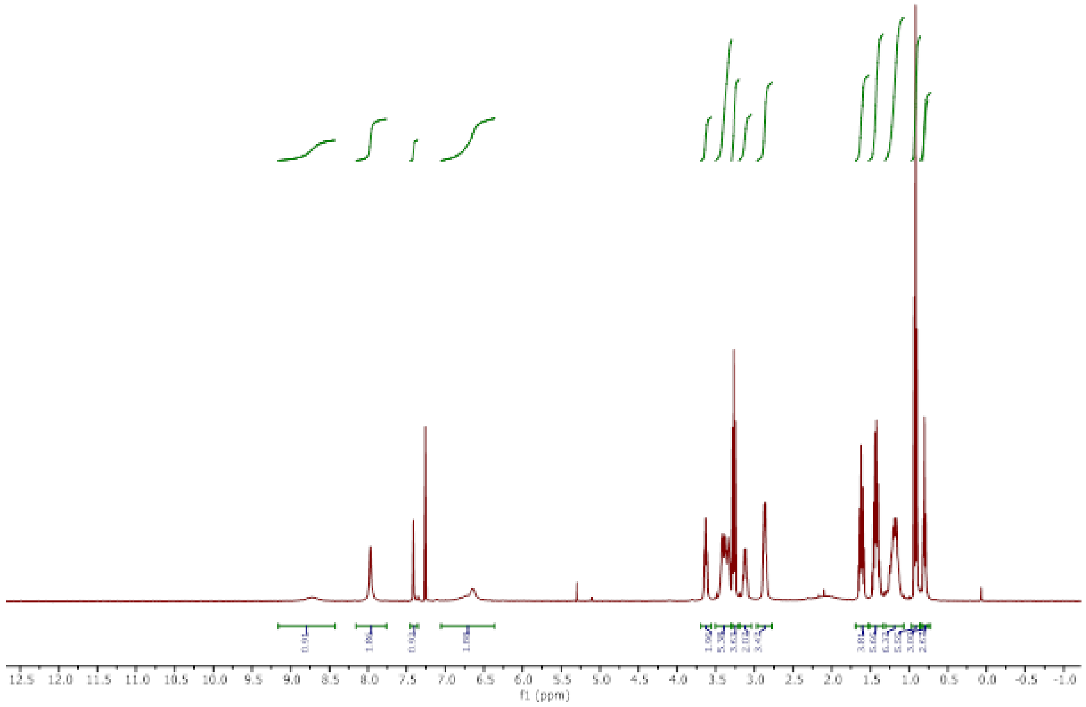

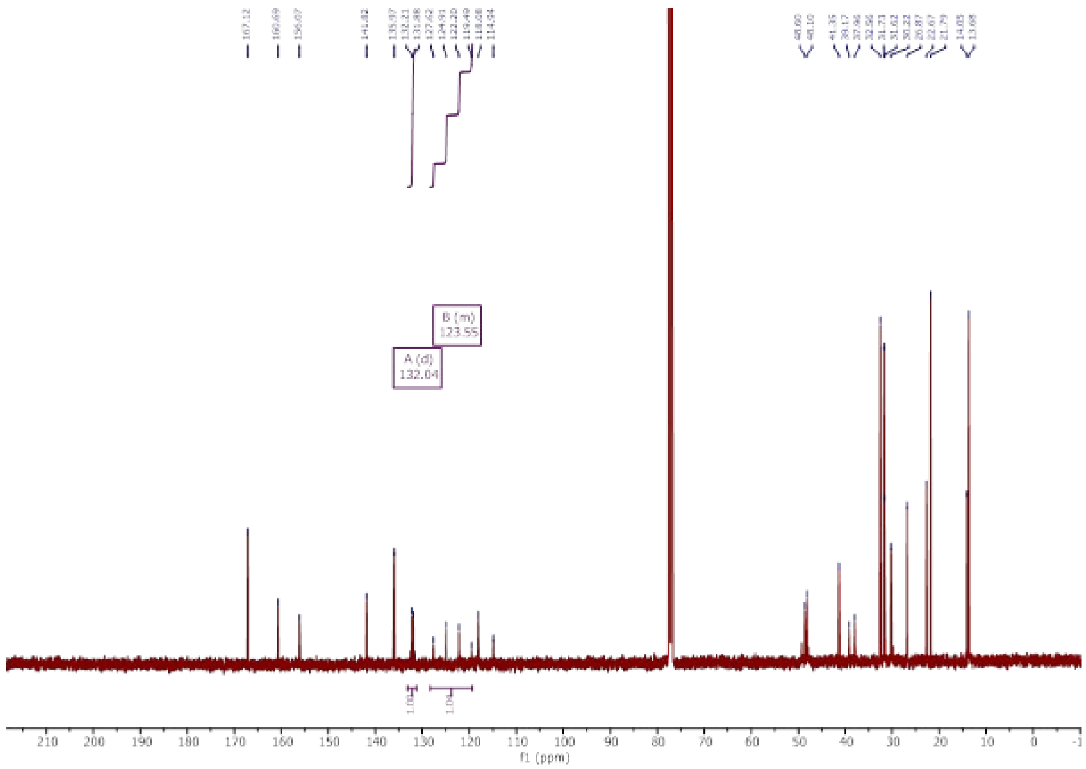

Compound 6: *N*-(3,5-b*is*(trifluoromethyl)phenylcarbamoyl)-*N'*-(2,3-b*is*(butylsulfanyl)maleimidoethyl)-*N'*,N''-b*is*(hexylcarbamoyl)diethylenetriamine (in $CDCl_3$)

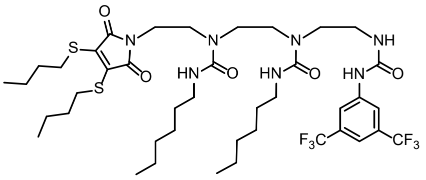

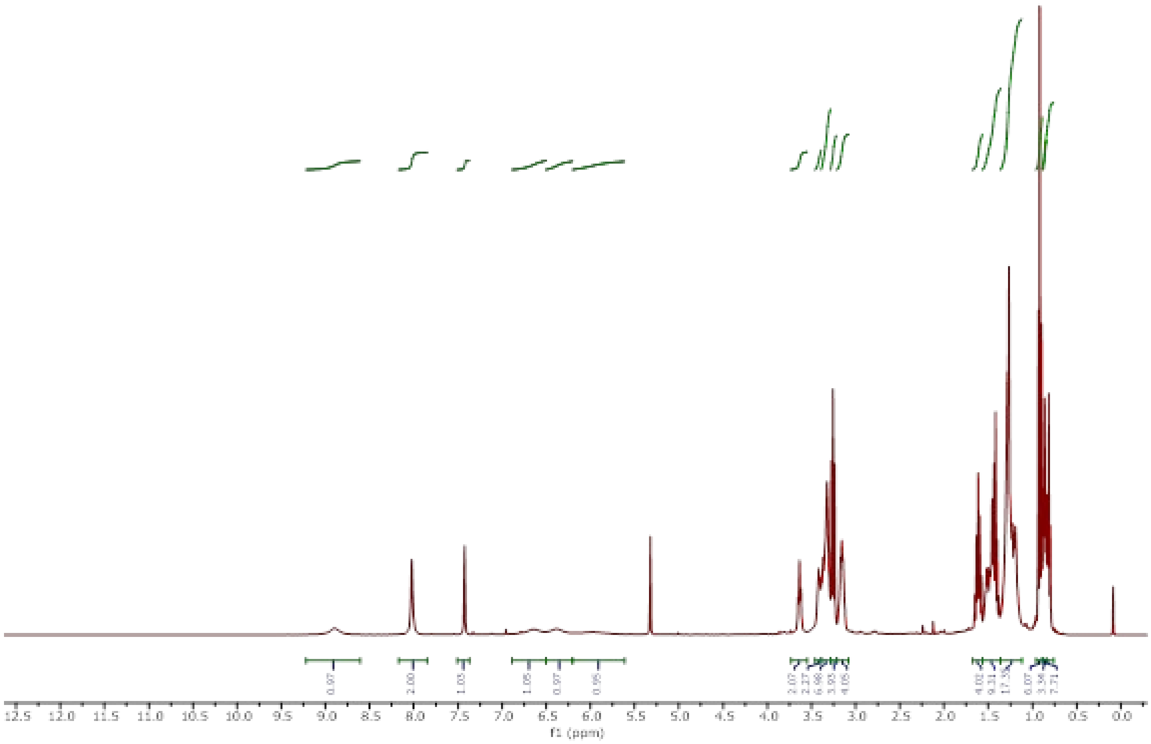

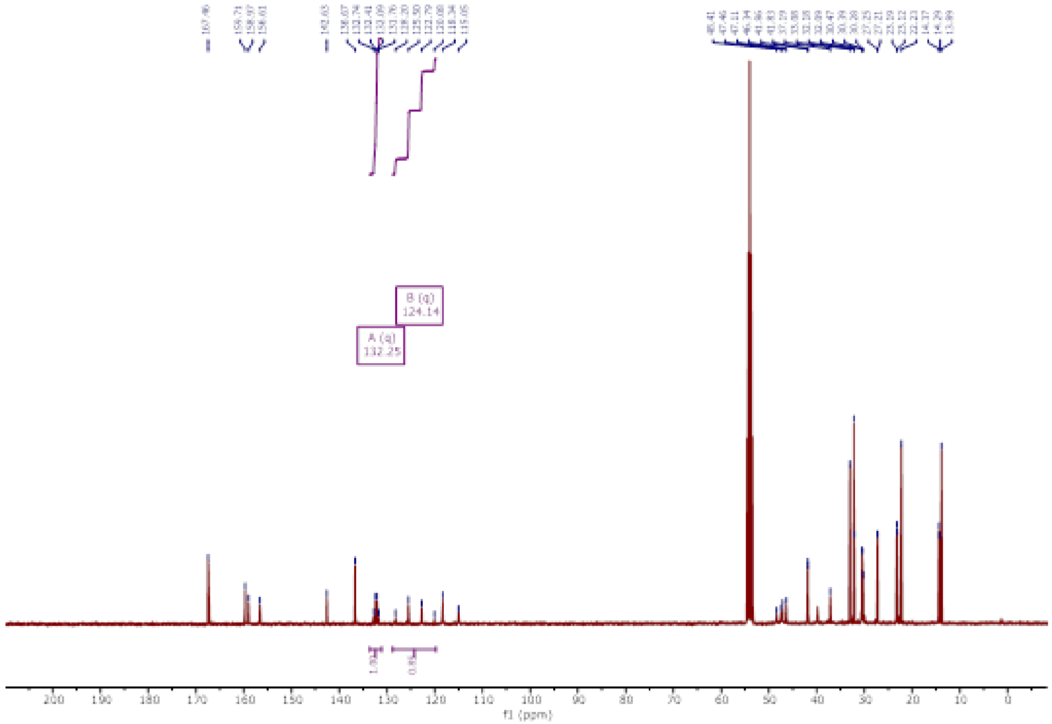

Compound 7: *N*-(3,5-b*is*(trifluoromethyl)phenylcarbamoyl)-*N*'-(2,3-b*is*(butylsulfanyl)maleimidoethyl)-*N*'-(phenylcarbamoyl)-N''-(hexylcarbamoyl)diethylenetriamine (in $CDCl_3$)

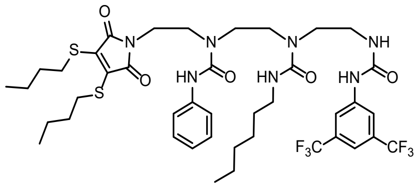

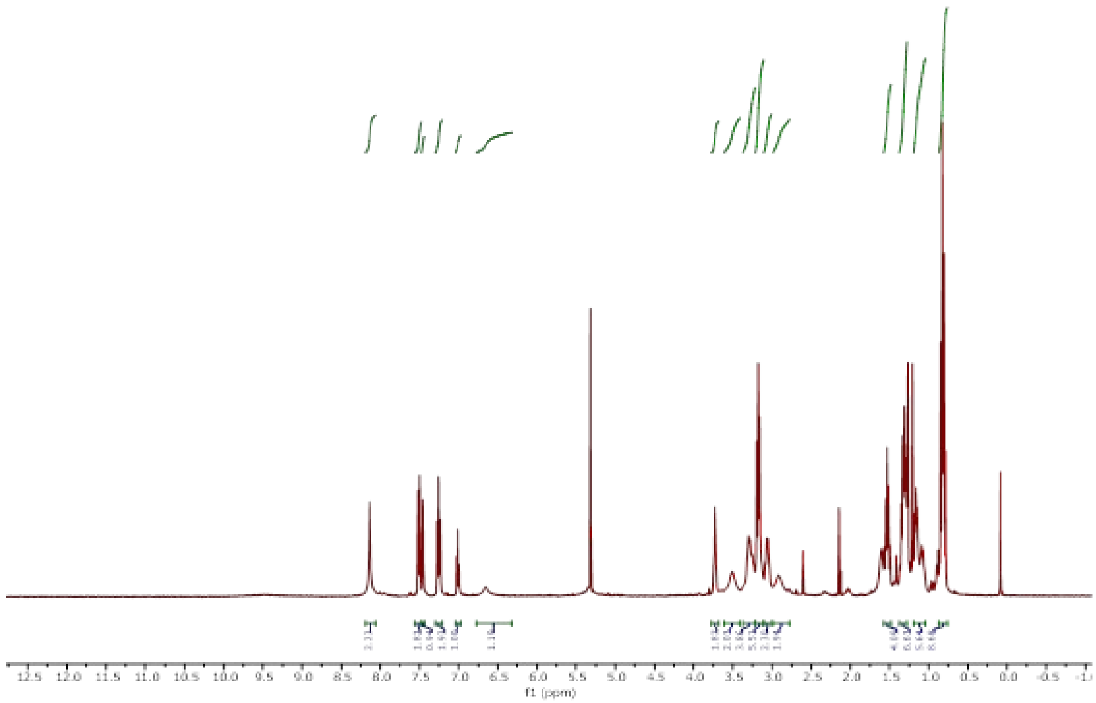

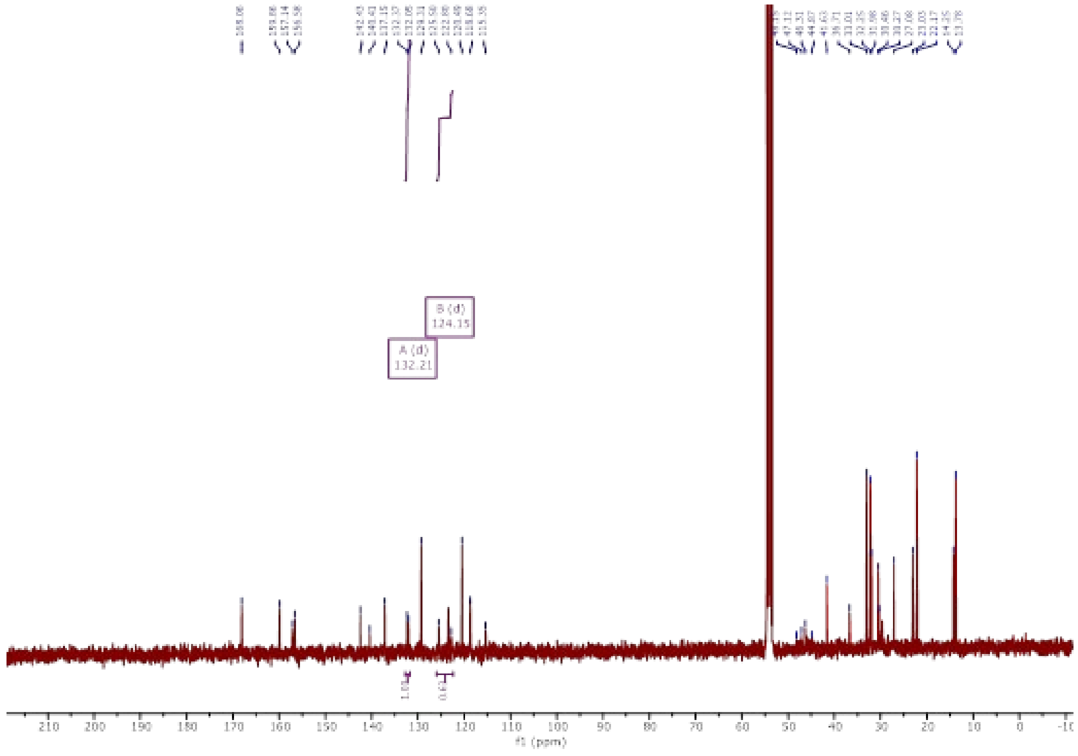

Compound s36: *N*-(benzyl)-*N*'-(2,3-b*is*(butylsulfanyl)maleimidoethyl)-*N*,N''-b*is*(hexylcarbamoyl)diethylenetriamine (in $CDCl_3$)

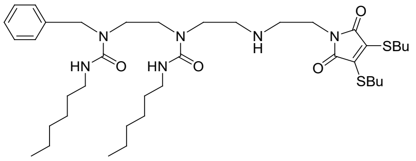

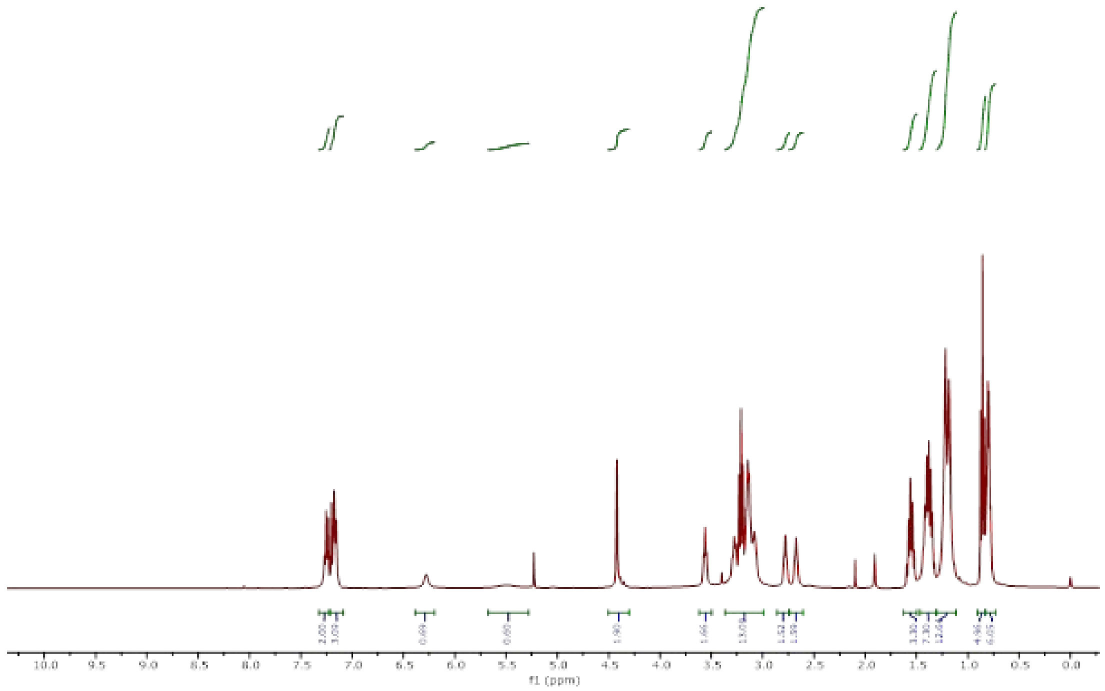

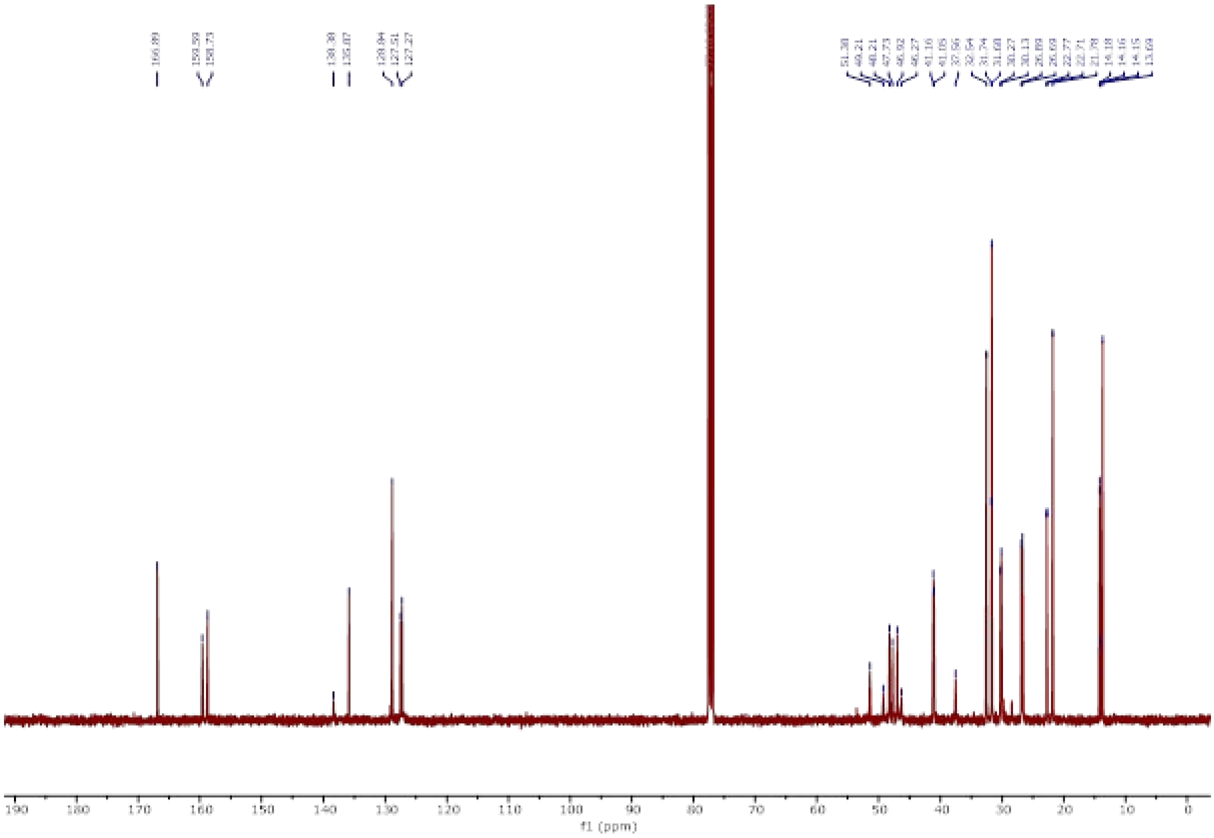

Compound 12: *N*-(benzyl)-*N*'-(2,3-b*is*(butylsulfanyl)maleimidoethyl)-*N*,N’,N’’-tri(hexylcarbamoyl)diethylenetriamine (in CDCl₃)

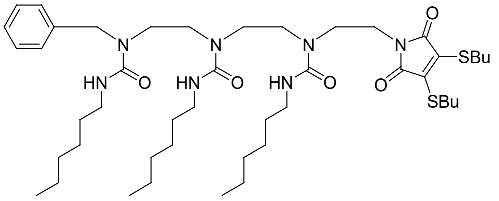

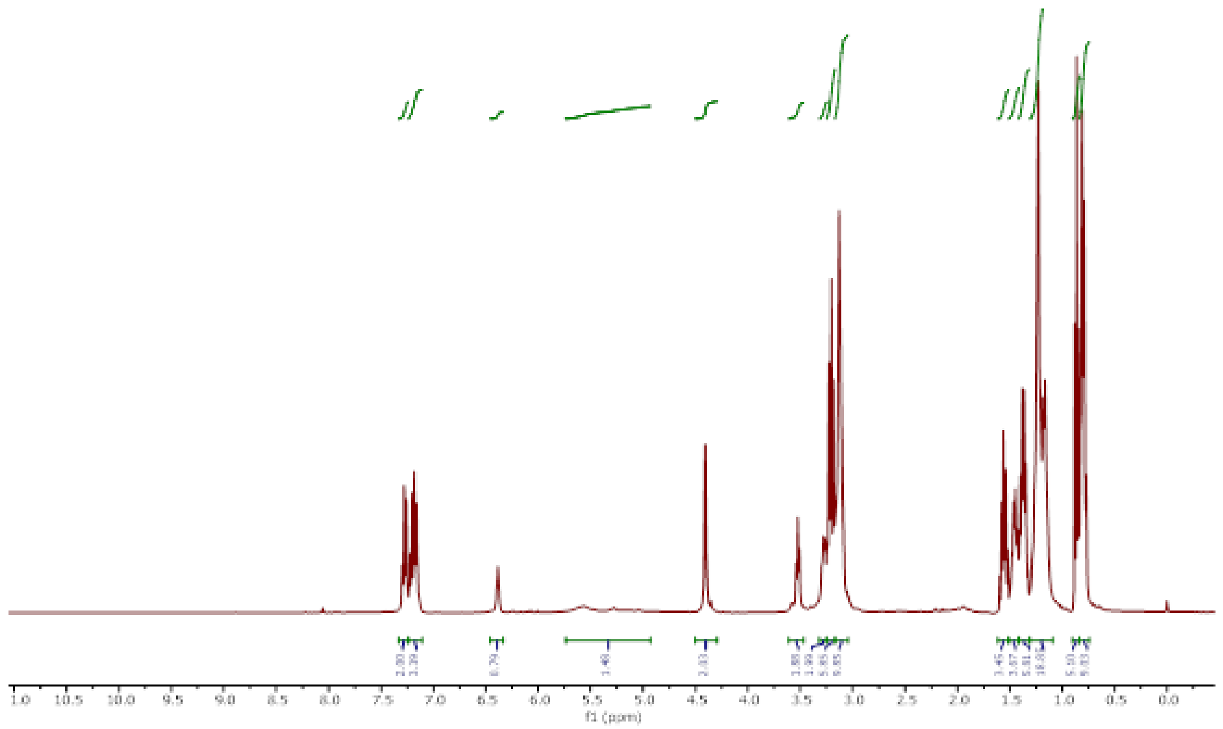

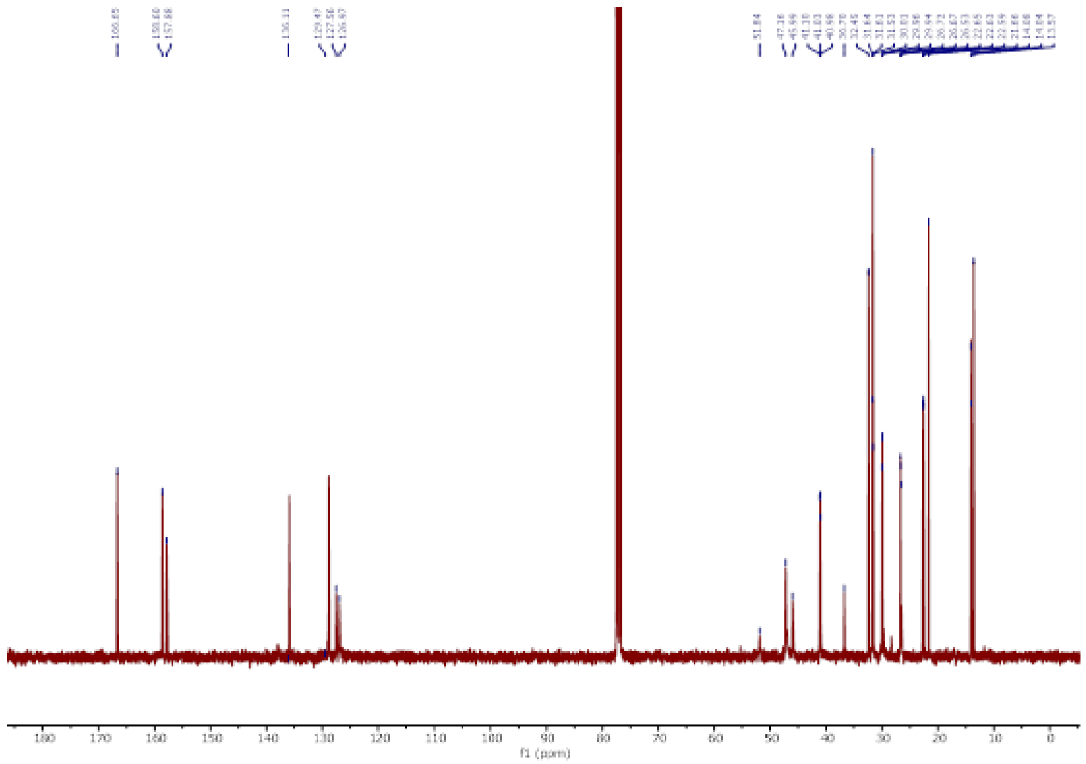

Compound 9: *N,N'*-diethyl- *N,N'*-b*is*(butylcarbamoyl)ethylenediamine (in $CDCl_3$)

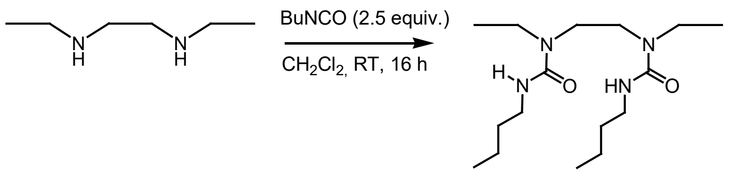

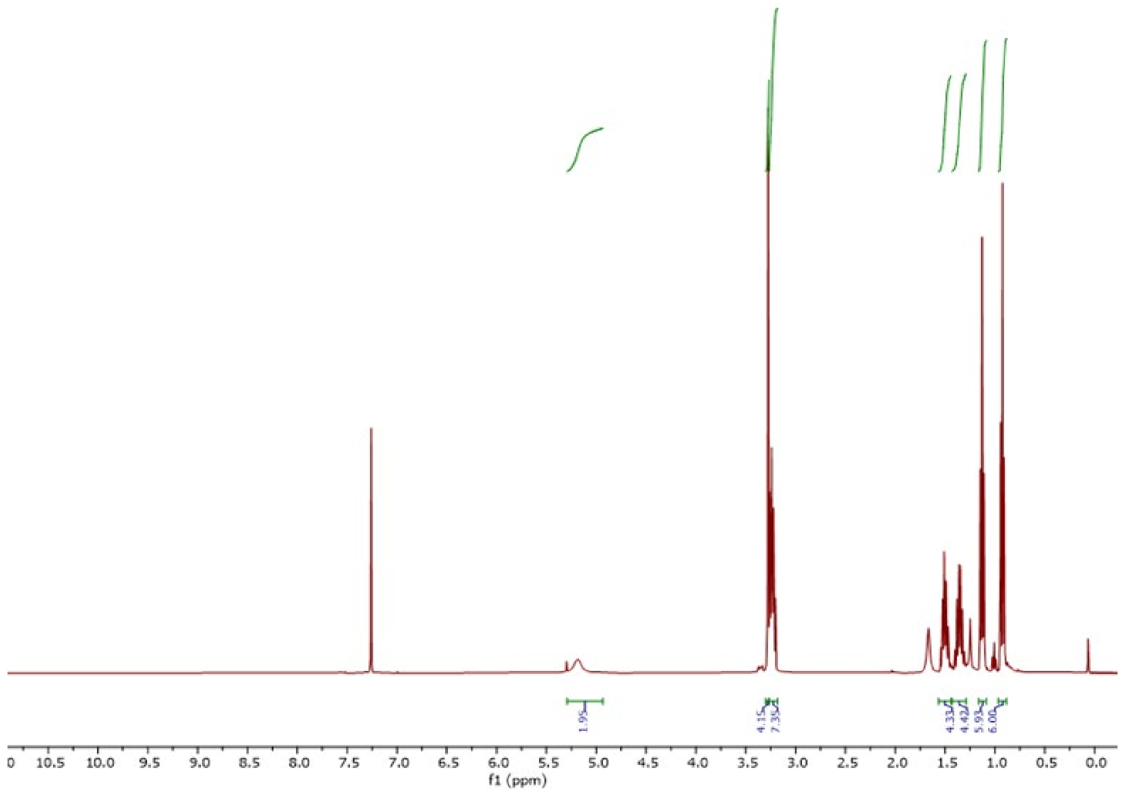

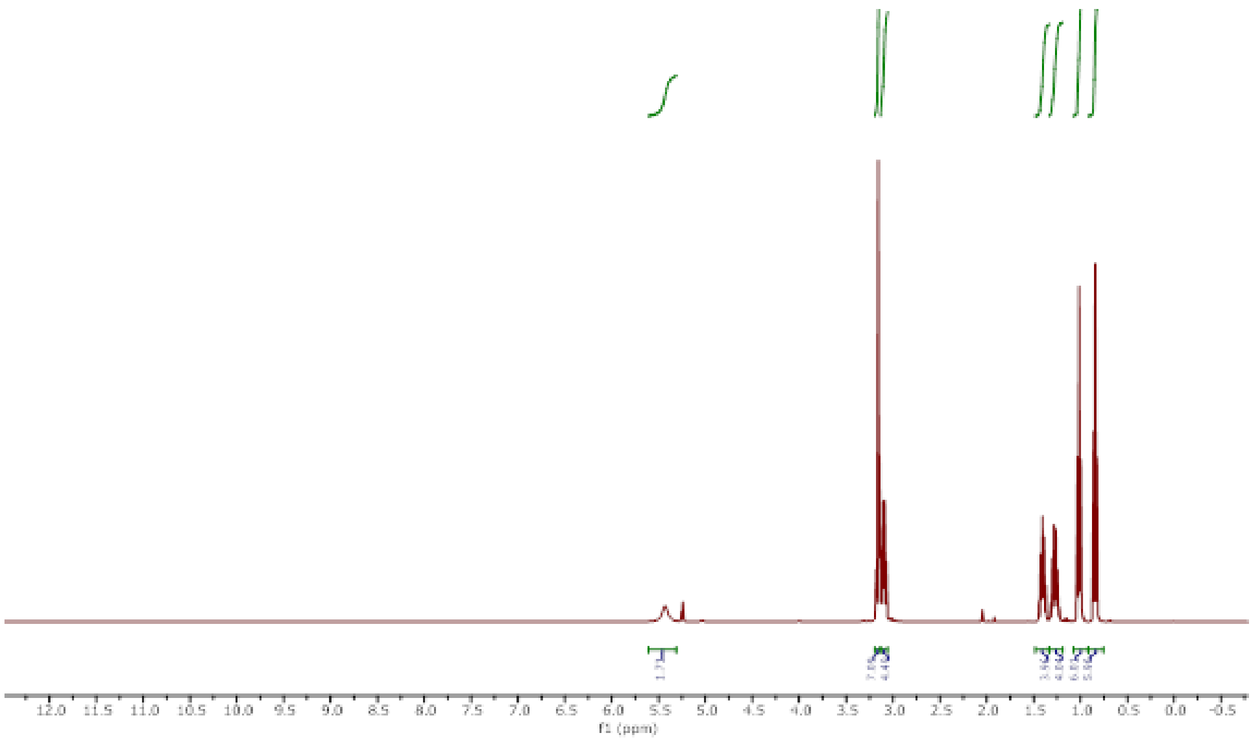

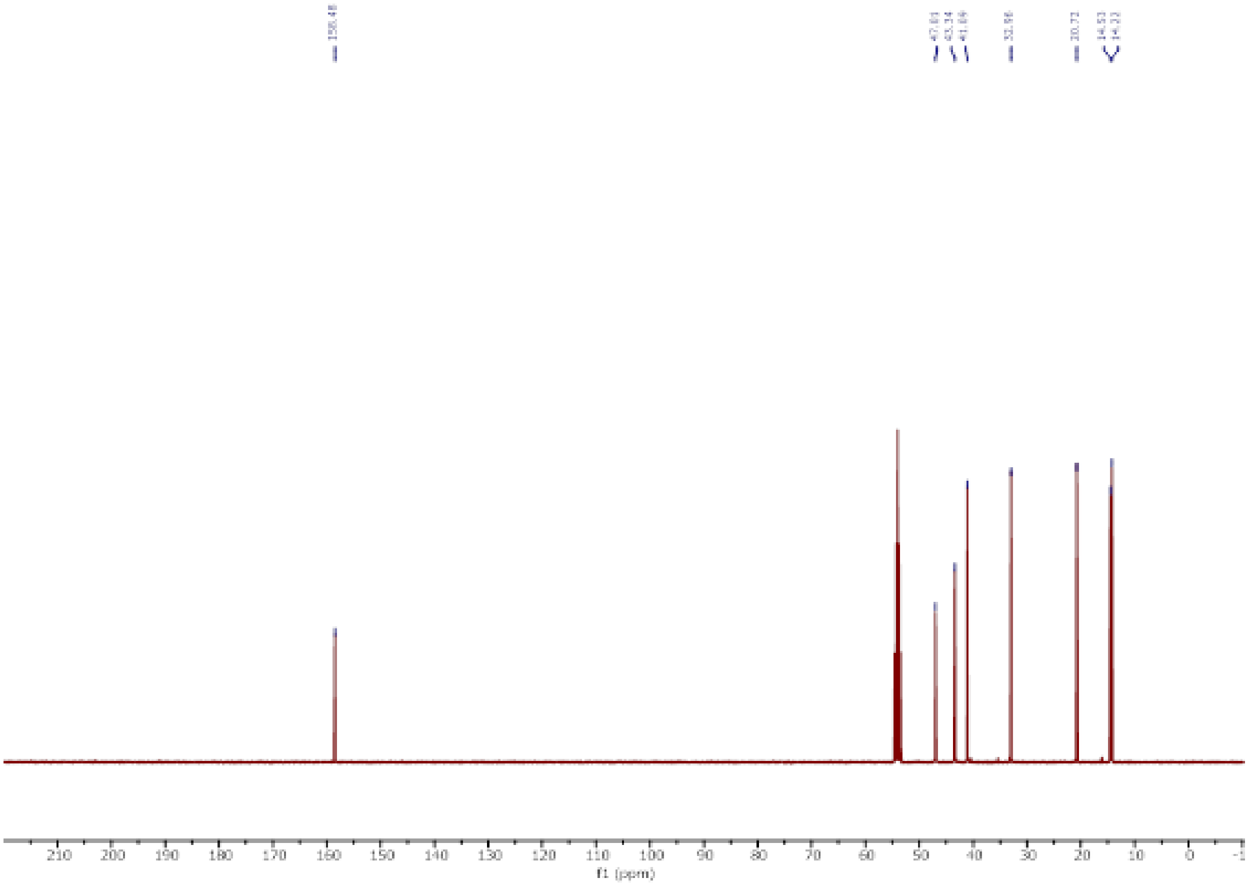
158.48
47.01
43.34
41.06
32.96
20.72
14.53
14.23
210
200
190
180
170
160
150
140
130
120
110
100
90
80
70
60
50
40
30
20
10
0
f1 (ppm)

5. NMR spectroscopy studies establishing native directionalities

5.1. Compound 1

5.1.1. Assignment of ^1H NMR signals

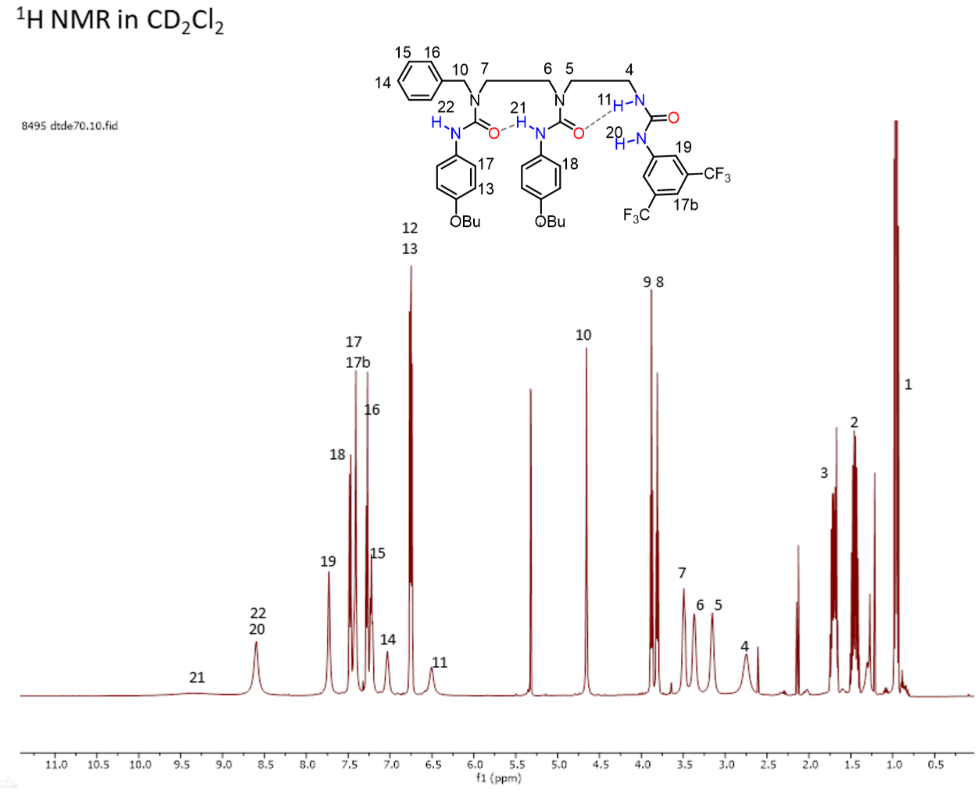

Figure S1: ^1H NMR spectrum of **1** in CD_2Cl_2 recorded at 25 °C (400 MHz).

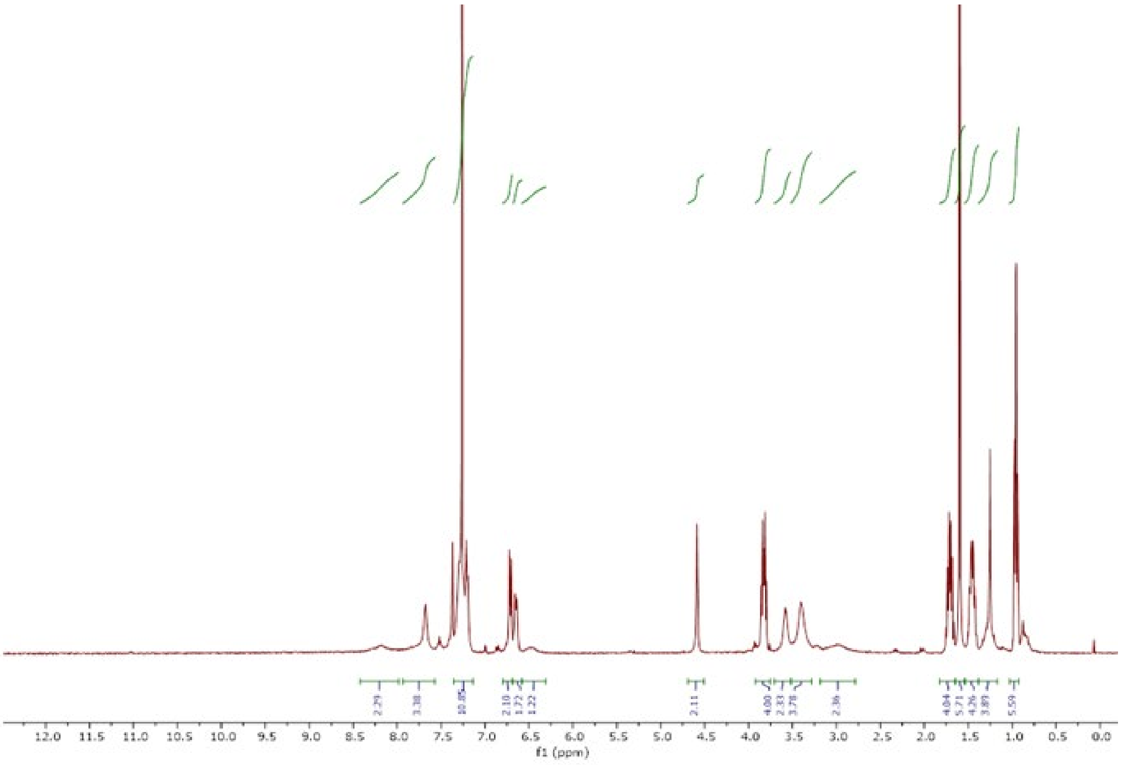

Figure S2: ^1H NMR spectrum of **1** in $CDCl_3$ recorded at 25 °C (400 MHz).

COSY in CD_2Cl_2

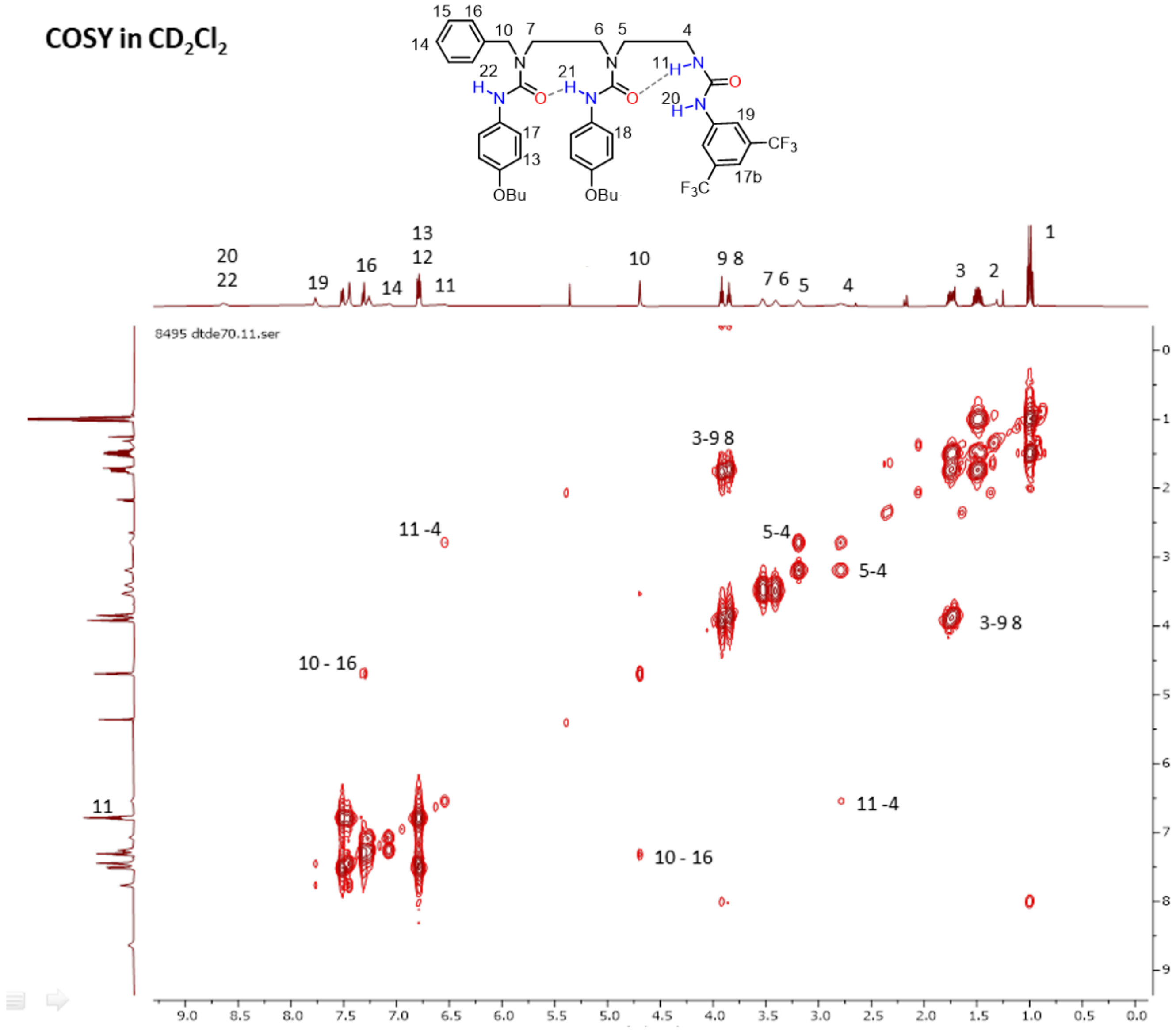

Figure S3: ^{1}H COSY NMR spectrum of **1** in CD_2Cl_2 recorded at 25 °C (400 MHz).

Signal assignment: ^{1}H COSY NMR experiment in CD_2Cl_2 at 25 °C allowed for assignment of NH^{11}, H^4, H^5 signals. Coupling between NH^{11} at 6.5 ppm and H4 at 2.75 ppm, and between H^4 and H^5 are observed. ^{1}H 2D NOESY experiments in CD_2Cl_2 at 25 °C were used to attribute other signals. Correlation signal between H^{10} and H^7 allowed for the assignment of H^7 then of H^6 (using COSY); correlation signal between NH^{11} (6.5 ppm) and NH^{20} (8.6 ppm) and correlation signal between NH^{20} and H^{19} (7.75 ppm) confirmed the NH^{20} signal assignment. Correlation signal between H^{10} and NH^{22} allowed the attribution of NH^{22} signal overlapping with NH^{20}. NH^{21} is the remaining broad NH signal at 9.3 ppm.

5.1.2. Assignment of directionality

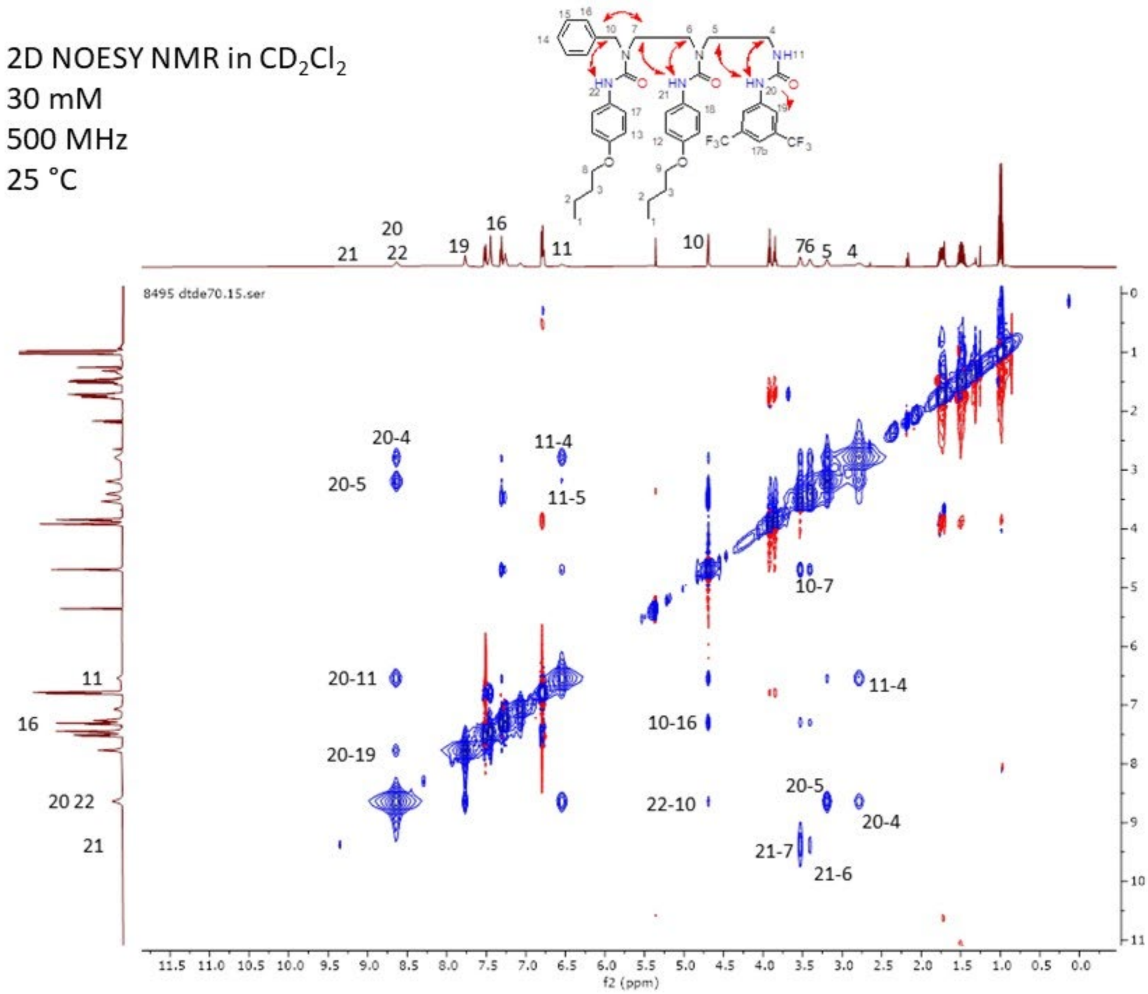

Figure S4: ^{1}H 2D NOESY NMR spectrum of **1** (30 mM in CD_2Cl_2) recorded at 25 °C (500 MHz).

2D NOESY experiment (30 mM in CD_2Cl_2) shows correlation signals between NH^{20} and both H^5 and H^4; no correlation signal is present between NH^{21} and either H^5 or H^4 but correlation signals between NH^{21} and both H^6 and H^7 are observed. Correlation signal between NH^{22} and benzylic H^{10} is present while no correlation signal between NH^{22} with either H^6 or H^7 is observed. The data are consistent with a preferential directionality of the hydrogen bond chain in the molecule as drawn. A correlation between NH^{11} and H^{10} is also observed, possibly indicative of some self-association at that concentration.[2]

The ^{1}H NMR chemical shift value of ureido NH^{11} (6.5 ppm) in CD_2Cl_2 at 25 °C (30 mM) is significantly downfield compared to the values reported for a non-hydrogen bonded ureido NH in *N*-3,5-di(trifluoromethyl)phenyl-*N*'-butylurea urea (4.67 ppm).[4] The ^{1}H chemical shift values of both ureido NH^{21} (9.33 ppm) is downfield compared to known values for non-hydrogen bonded ureido NH of branched *p*-methoxyphenylureas, indicating its hydrogen bonding state. Dilution studies were carried out to establish the effect of self-aggregation on the conformations in solution. Upon dilution in CD_2Cl_2, the observed upfield shifts for all the ureido NH protons could be attributed to some degree of self-association. VT ^{1}H NMR of **1** recorded between 25 °C and -80 °C in CD_2Cl_2 show a general downfield shift for all the ureido NH of the structure upon cooling the sample without any signal decoalescence.

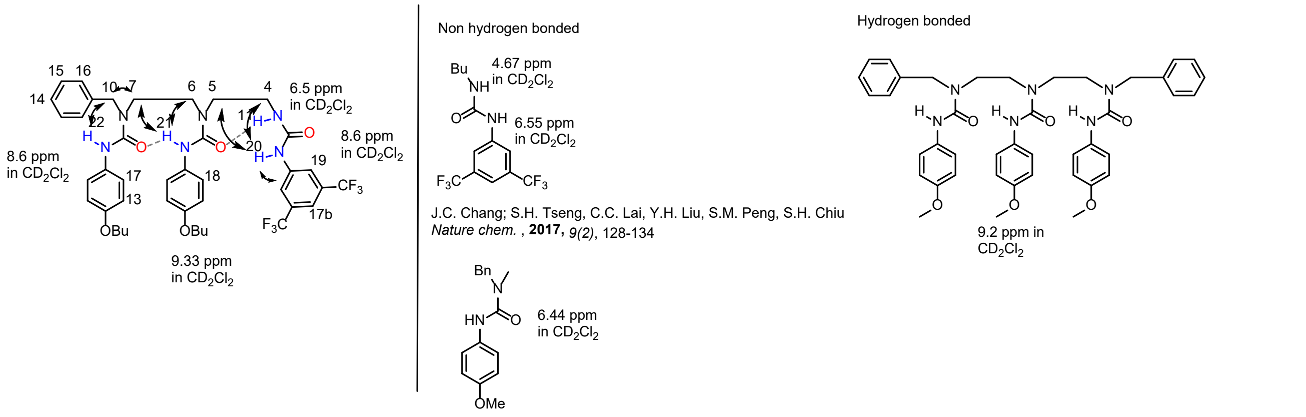

Figure S5: ^{1}H NMR chemical shift values of ureido NH for **1** in CD_2Cl_2 at 25 °C and published values of ureido NH in similar topological environments

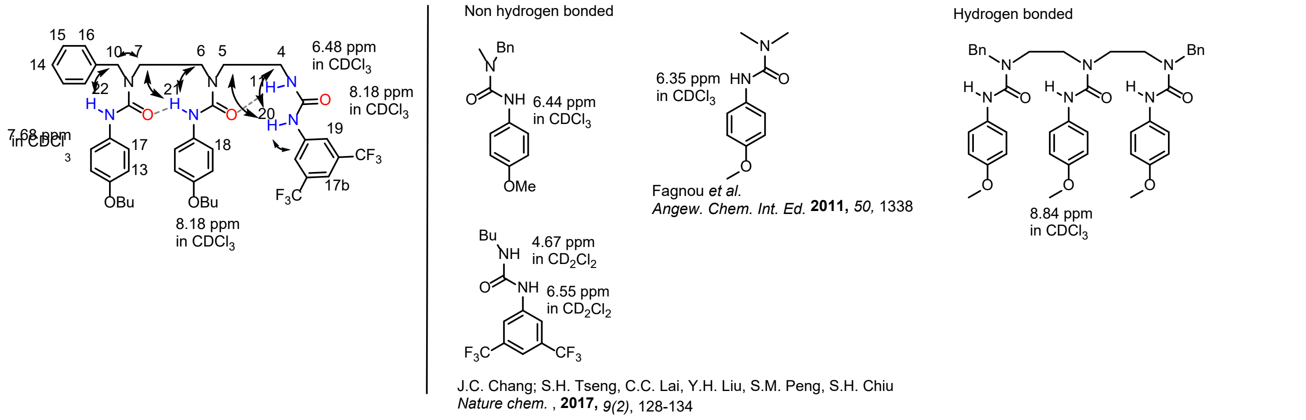

Figure S6: ^{1}H NMR chemical shift values of ureido NH for **1** in $CDCl_3$ at 25 °C and published values of ureido NH in similar topological environments

5.1.3. Dilution study in CD_2Cl_2 at 25 °C

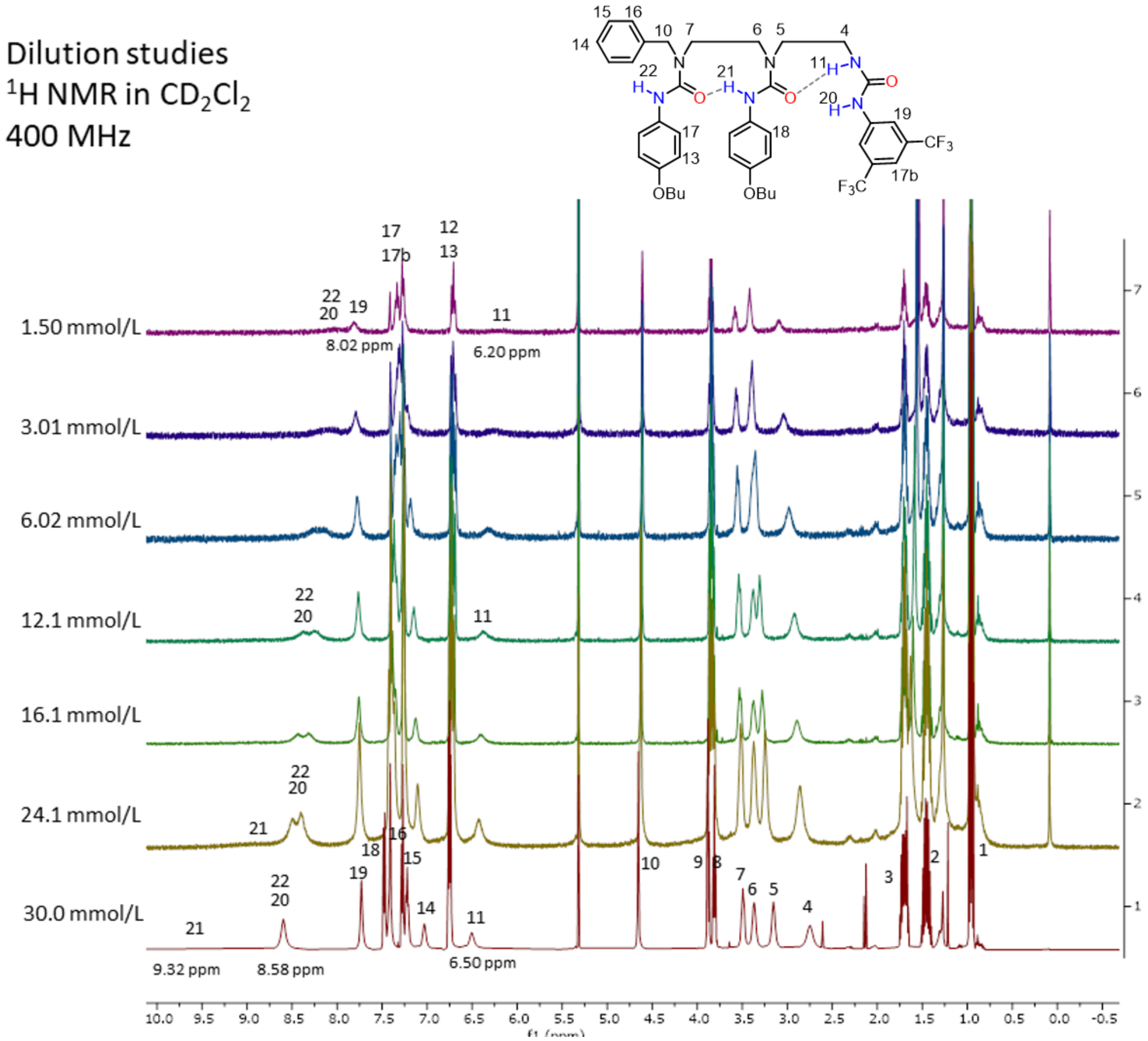

Figure S7: overlay of ^1H NMR spectra of **1** in CD_2Cl_2 at concentrations between 1.5 mM and 30 mM recorded at 25 °C (400 MHz).

5.1.4. Variable temperature ^{1}H NMR study (20 °C to -80 °C)

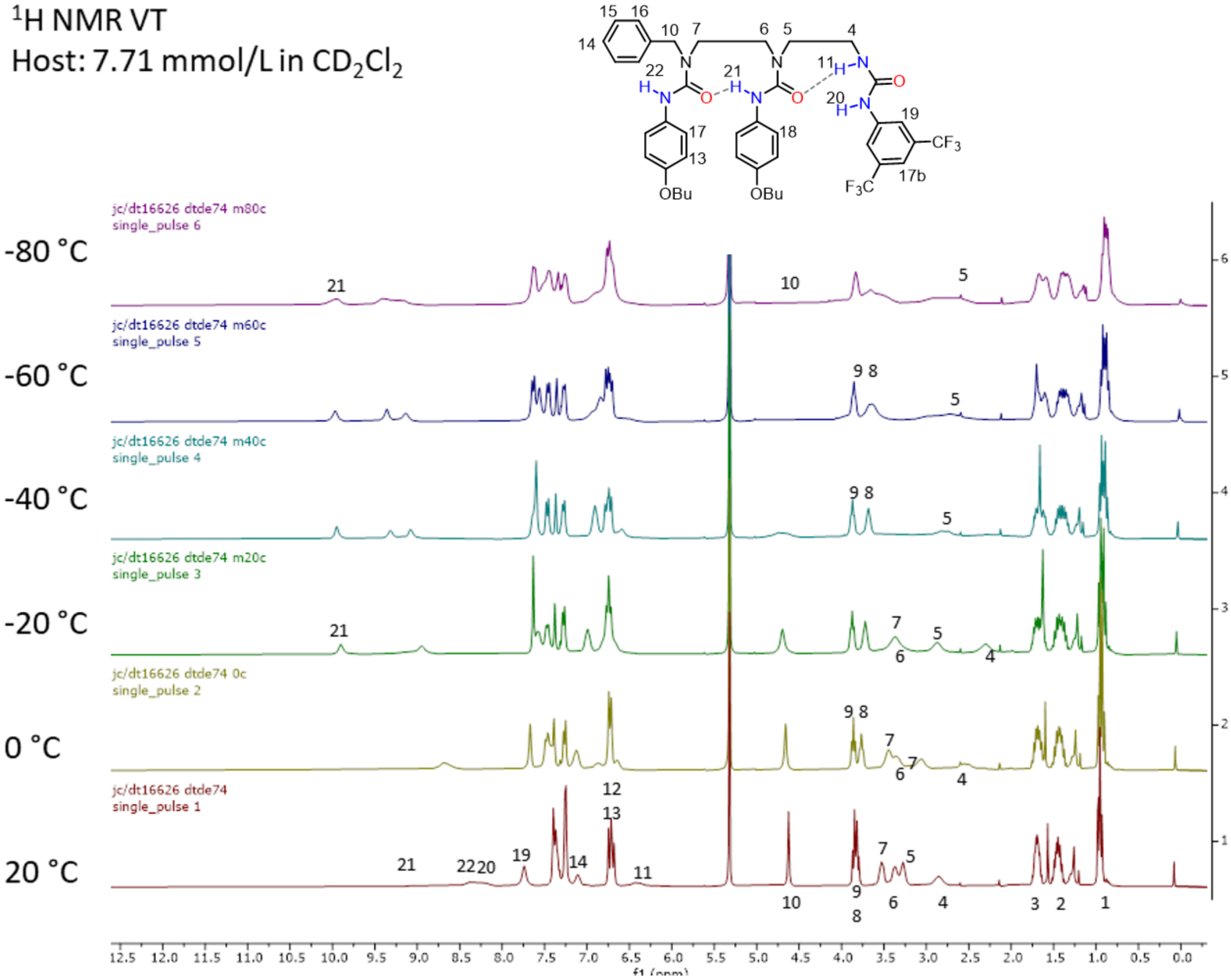

Figure S8: overlay of ^{1}H NMR spectra of **1** in CD_2Cl_2 at temperatures between 25 °C and -80 °C (300 MHz).

5.2. Compound 2

5.2.1. Assignment of ^{1}H NMR signals in $CDCl_3$

Assignment of signals: ^{1}H COSY NMR experiment in $CDCl_3$ shows couplings between NH^{14} and H^{8}, between NH^{15} and H^{7}, allowing for the assignment of both NH^{14} and NH^{15} signals. Overlapping of NH^{13} signal with the methylene signal of the benzyl group was observed at 25 °C. To overcome this overlapping, a range of temperatures was screened in CD_2Cl_2. Variable temperature ^{1}H NMR between 5 °C and 35 °C clearly showed the NH^{13} signal. 2D NOESY experiment shows correlation signals between NH^{13} and H^{12}, between ^{t}butyl H^{2} and H^{12}, allowing for assignment of NH^{13} signal and giving indication on the directionality of the hydrogen bond chain. ^{15}N HSQC experiment confirmed the position of the NH signals.

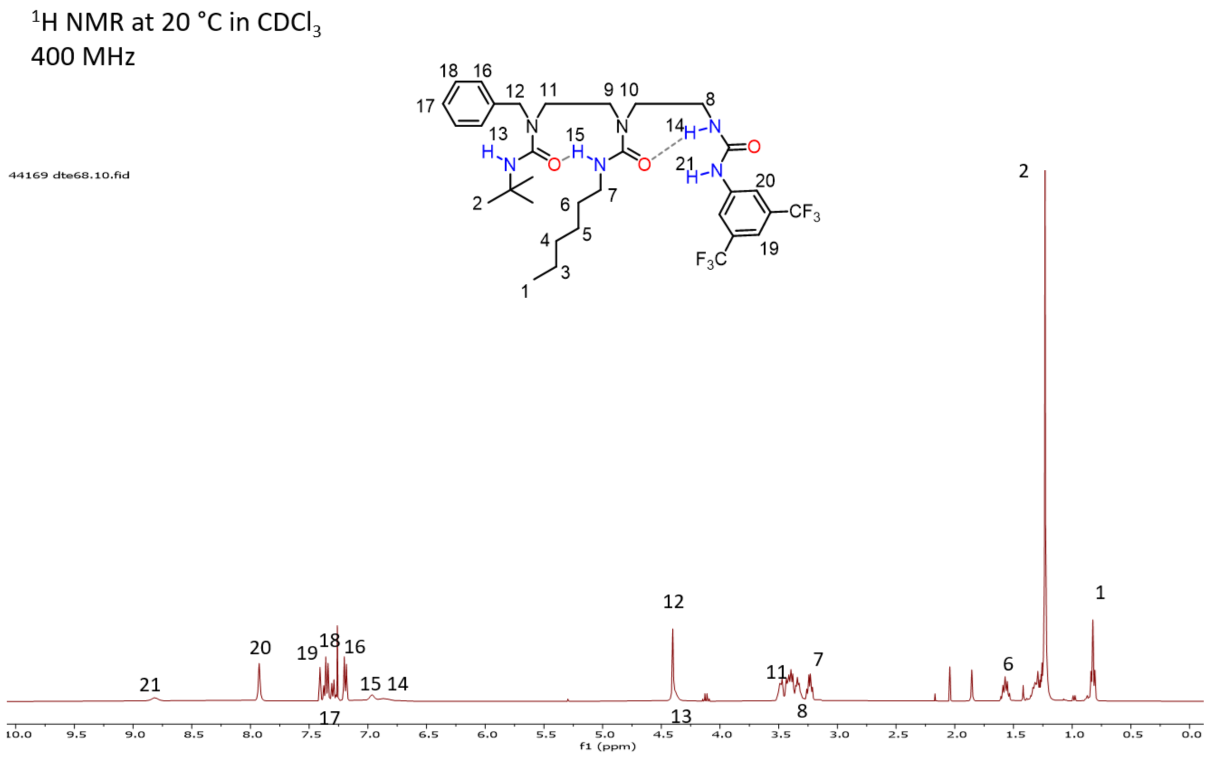

Figure S9: ^{1}H NMR spectrum of **2** in $CDCl_3$ at 25 °C (400 MHz).

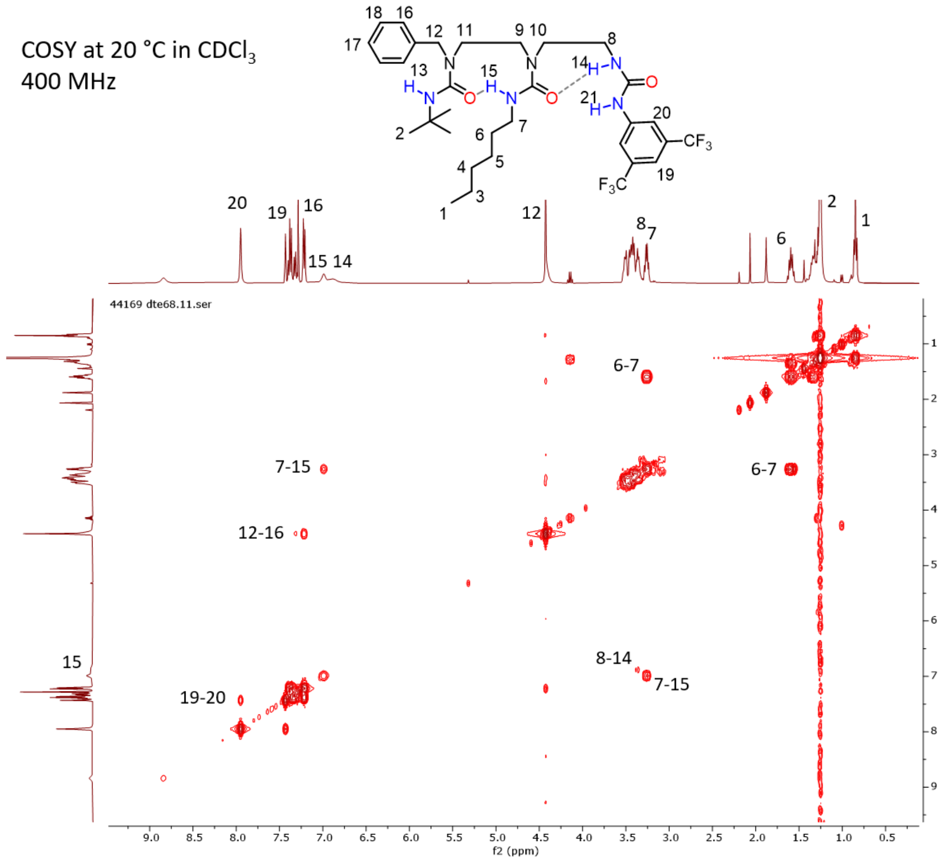

Figure S10: ^{1}H COSY NMR spectra of **2** in $CDCl_3$ at 25 °C (400 MHz).

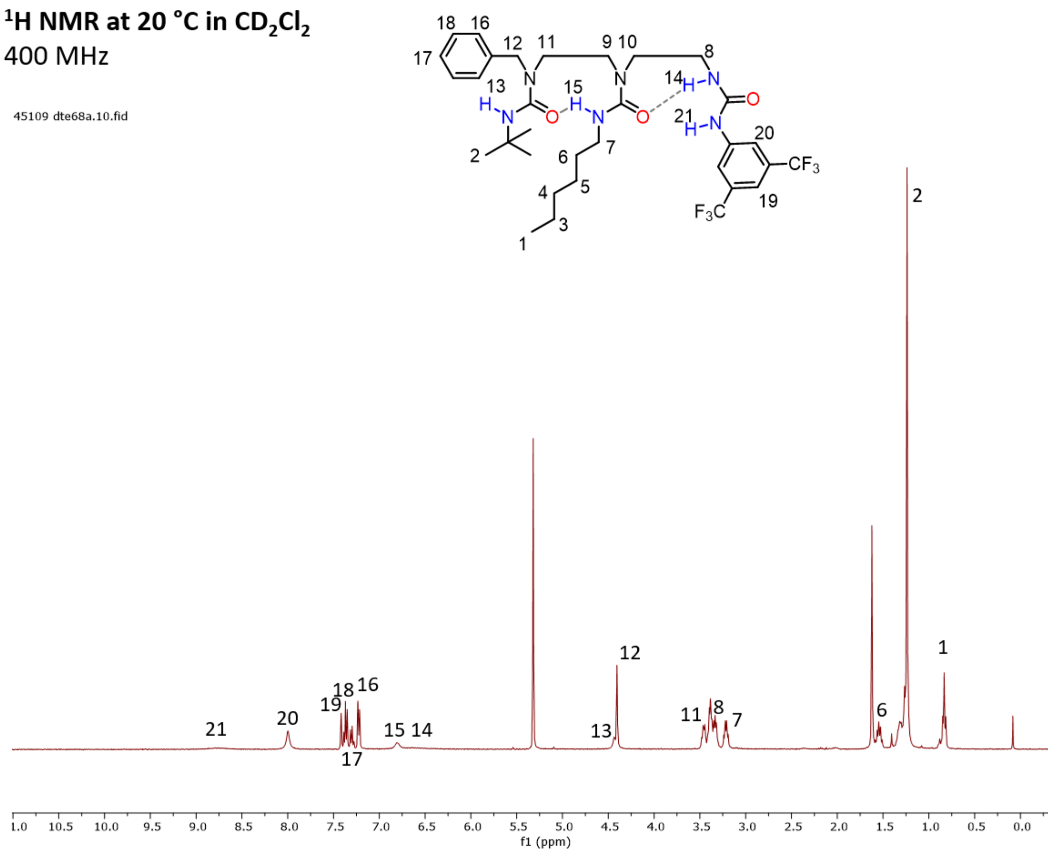

Figure S11: ^{1}H NMR spectrum of **2** in $CDCl_3$ at 25 °C (400 MHz).

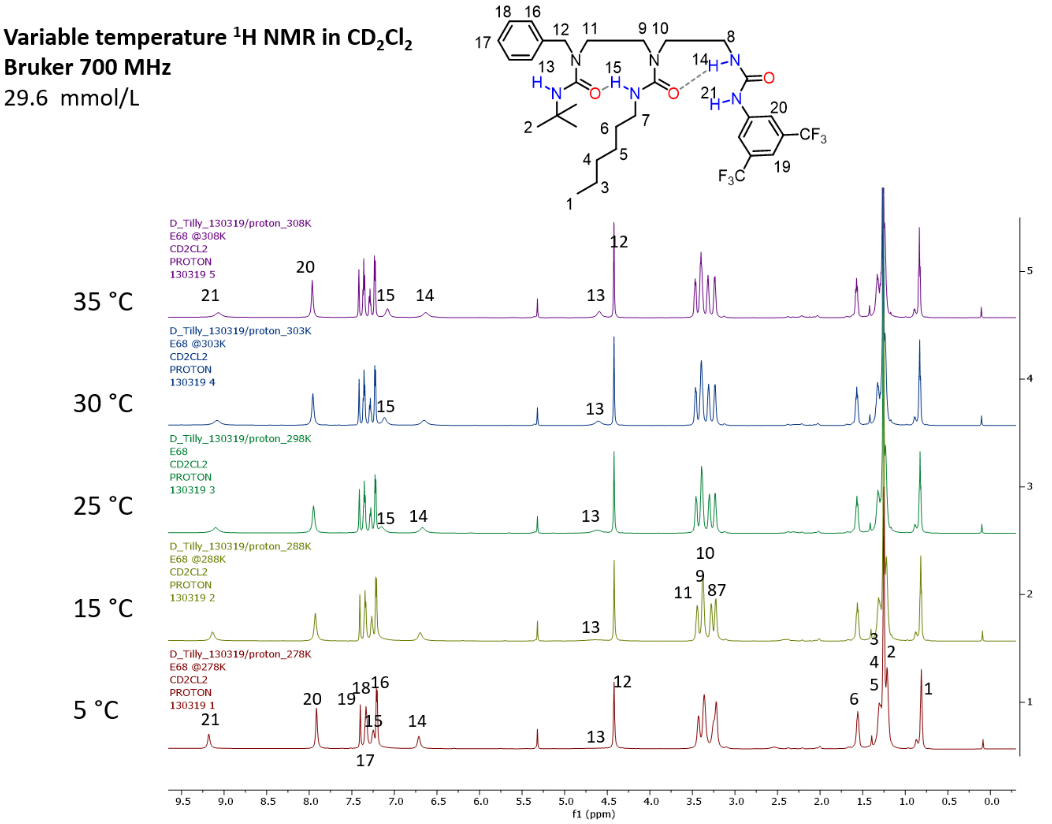

Figure S12: overlay of ^{1}H NMR spectra of **2** (29.6 mM in CD_2Cl_2) recorded (700 MHz) between 5 °C and 35 °C.

A-

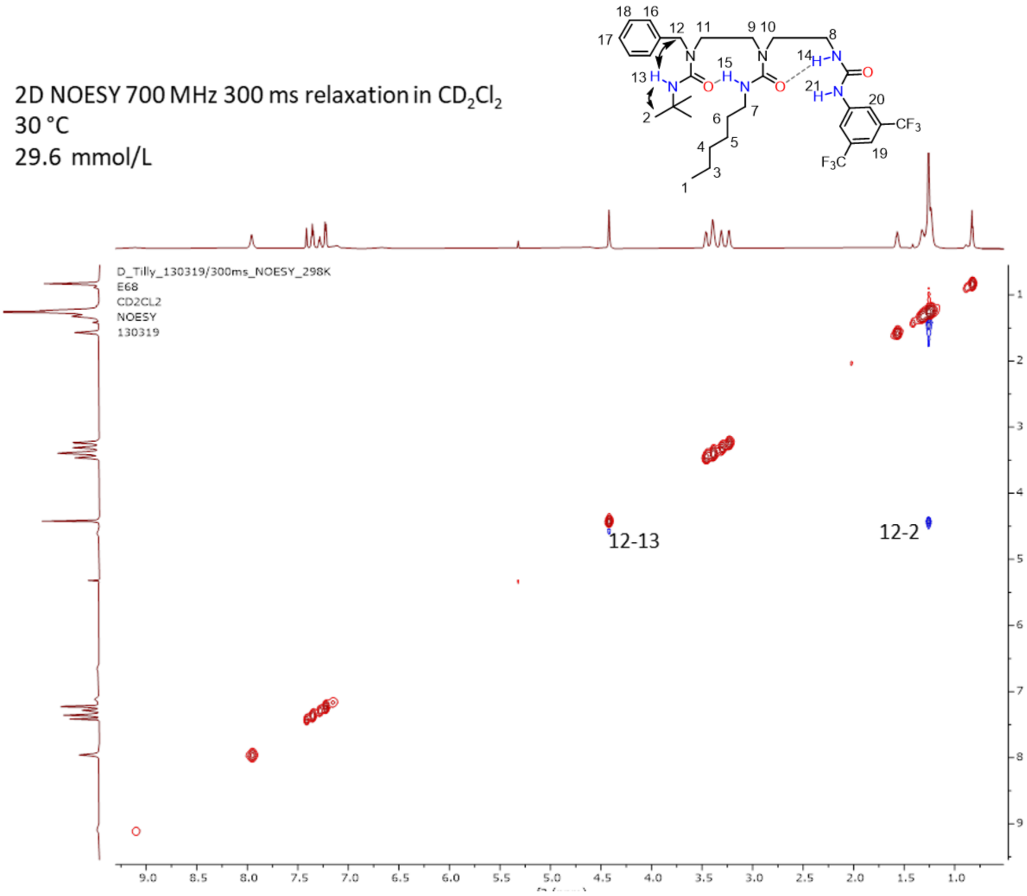

B-

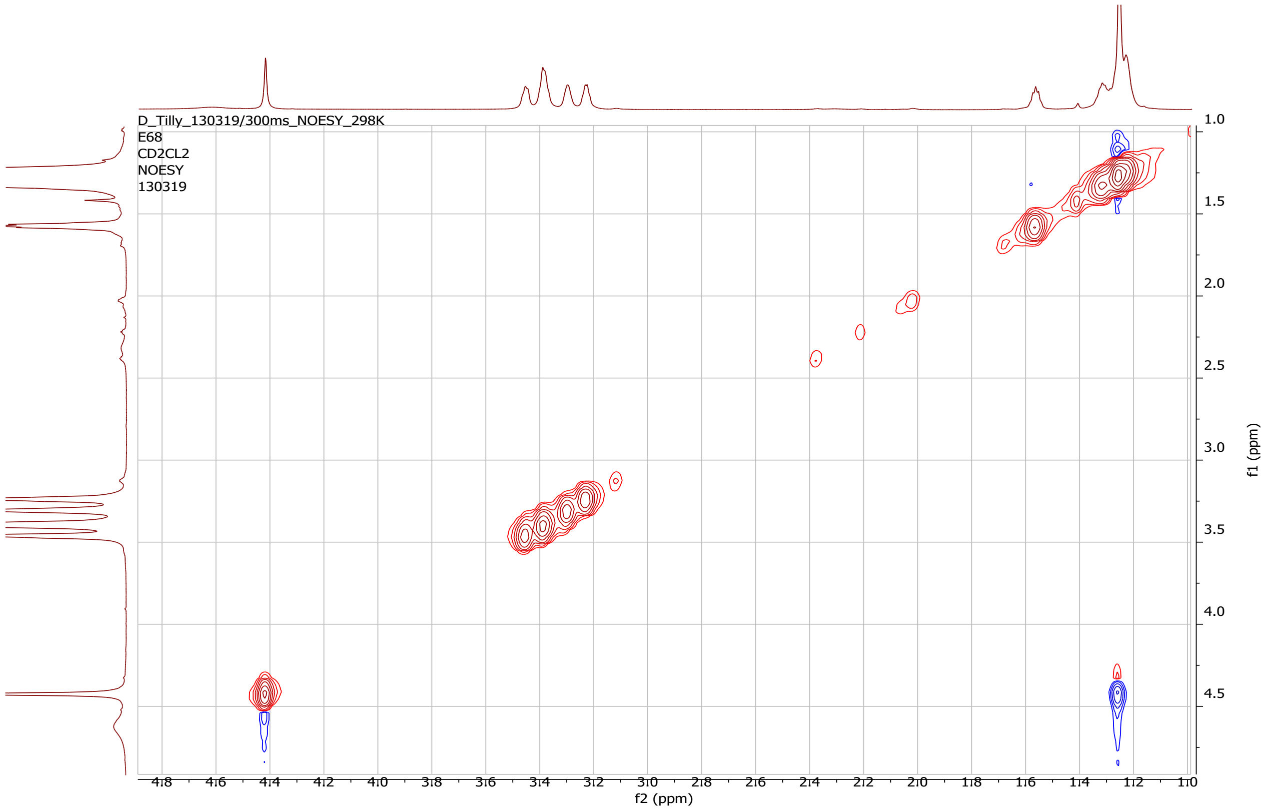

Figure S13: a) ^{1}H 2D NOESY NMR spectrum of **2** in CD_2Cl_2 at 30 °C (700 MHz), b) expansion at 1.2-4.8 ppm. The ^{1}H NMR signals for benzylic CH_2 (4.45 ppm) and terbutylureido NH are close at 298K: we used a 700 MHz spectrometer for

better result, optimised further by testing different temperatures of acquisition from 278 K to 308 K and adjusting the relaxation time, found 300 ms to be the best. The best resolution of NHtBu ^{1}H NMR signal was obtained at 308 K (benzylic CH_2 4.45 ppm, tBuNH broad signal at 4.6 ppm). The different phase of NOE signals (in blue) indicates NOESY signals between broad tBuNH and CH_2Ph (x/y 4.44ppm/4.6 ppm), between tBuNH and tBu (x/y 1.2 ppm/4.6 ppm), between CH_2Ph and tBu (x/y 1.2 ppm/4.44 ppm), all diagnostic of the conformation. ^{15}N NMR HSQC on a 700 MHz spectrometer confirmed the NHtBu signal at 4.6 ppm. Other experiments such as 1D selective gradient NOESY, TOCSY, and modifying the relaxation times from 100ms to 500 ms were carried out on the sample with no improvement.

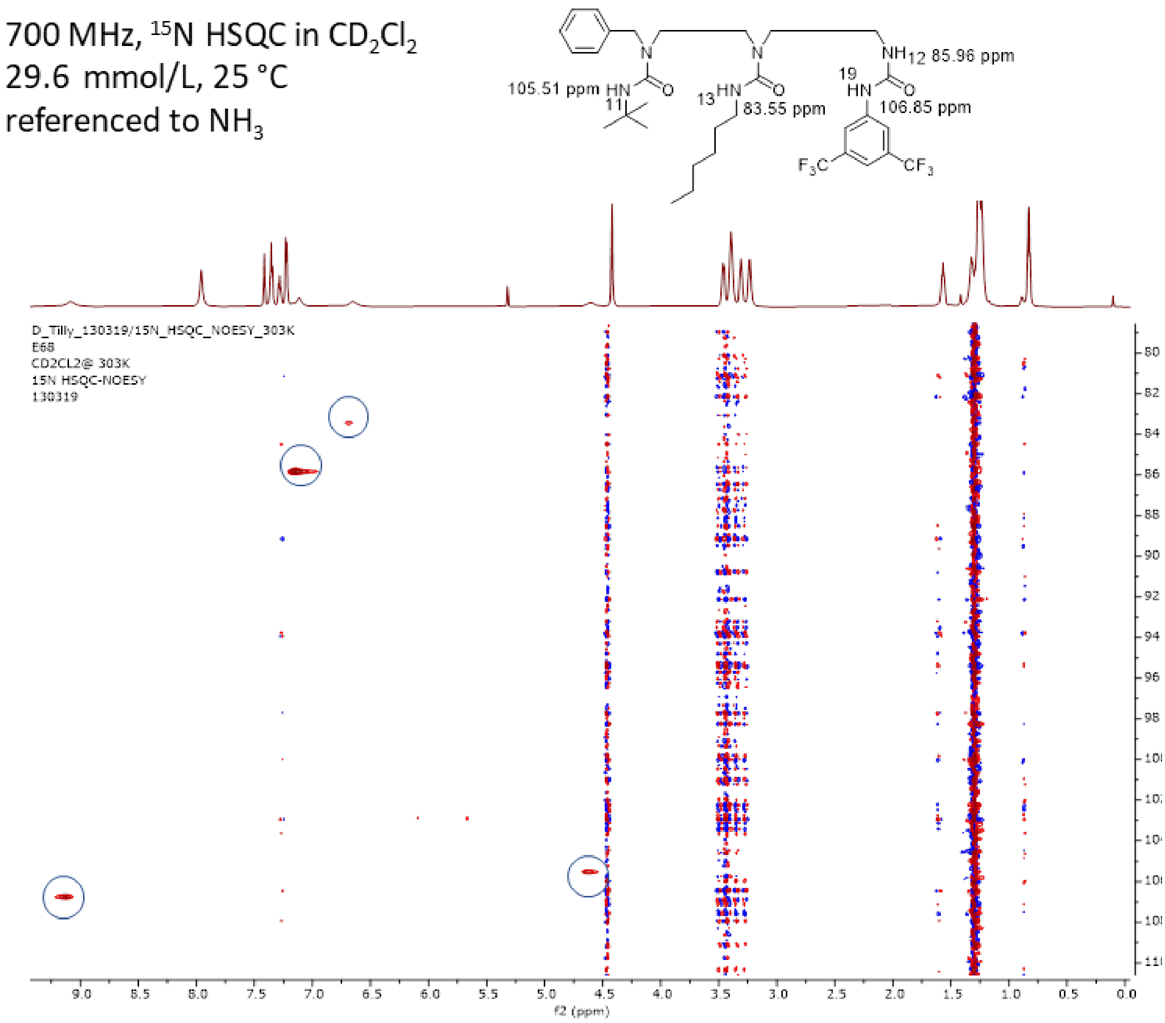

Figure S14: ^{1}H-^{15}N HSQC NMR spectrum of **2** (29.6 mM in CD_2Cl_2) at 25 °C (700 MHz) (referenced to NH_3).

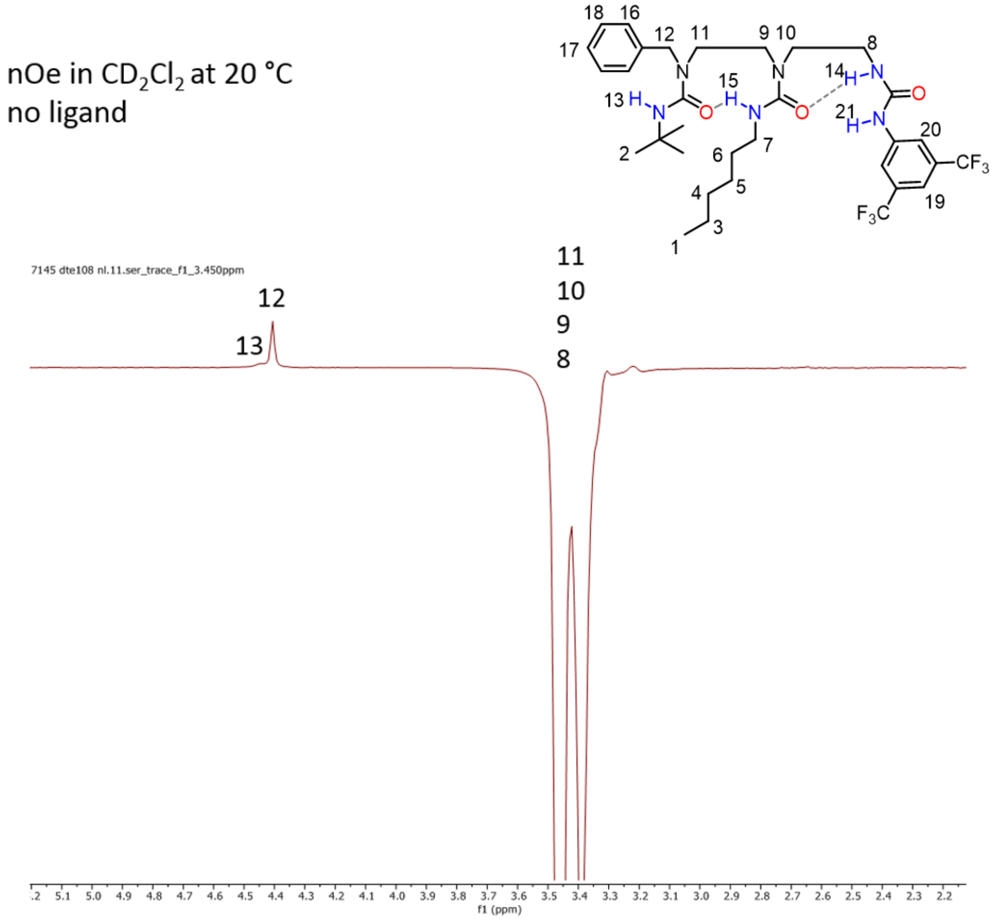

Figure S15: ^{1}H nOe NMR spectrum of **2** in CD_2Cl_2 at 25 °C (500 MHz), irradiation of H8/9/10/11.

5.2.2. Assignment of directionality in $CDCl_3$

The ^{1}H NMR chemical shift values of ureido NH^{21} (8.77 ppm), NH^{14} (6.80 ppm), NH^{15} (6.63 ppm), NH^{13} (4.44 ppm) at 25 °C in CD_2Cl_2 were compared to the ^{1}H NMR chemical shift values of published compounds having ureido NH in similar topological environments. The comparison indicates that NH^{13} is not involved in a direct hydrogen bond with a carbonyl while both NH^{15} and NH^{14} are in hydrogen bonding with a carbonyl. The data are consistent with a well-defined conformation with intramolecular hydrogen bonds. nOe experiment irradiating $H^{8/9/10/11}$ shows a correlation signal with H^{12} and a correlation signal of weak intensity with H^{13}, the weak intensity of it is indicative of a hydrogen bond directionality as drawn.

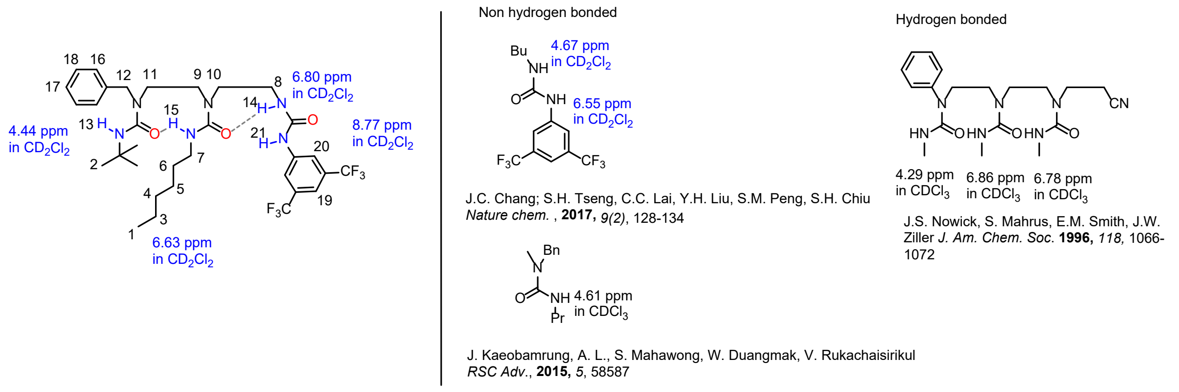

Figure S16: ^{1}H NMR chemical shift values of ureido NH for **2** in CD_2Cl_2 at 25 °C and published values of ureido NH in similar topological environments

5.2.3. Assignment of ^{1}H NMR signals and directionality in benzene d6

NMR data of **2** dissolved in benzene d6 were collected to evaluate the influence of the solvent on the directionality of the hydrogen bond chain. ^{1}H COSY experiment shows couplings between NH^{14} and H^8, between NH^{15} and H^7, allowing for the assignment of both NH^{14} and NH^{15} signals. 2D NOESY experiment shows correlation signals between NH^{21} and both NH^{14} and H^{20}, between NH^{13} and ^{t}butyl H^2, allowing for the assignment of NH^{21} and NH^{13}. HSQC and HMBC experiments confirmed the assignment of signals. 2D NOESY NMR data do not offer more information on the directionality of the hydrogen bond chain due to the broadness of the NH signals. The values of chemical shifts of the NH signals are comparable to the values obtained for the same NH signals in CD_2Cl_2, indicative of a continuous chain of hydrogen bonds with the same directionality in benzene as in CD_2Cl_2.

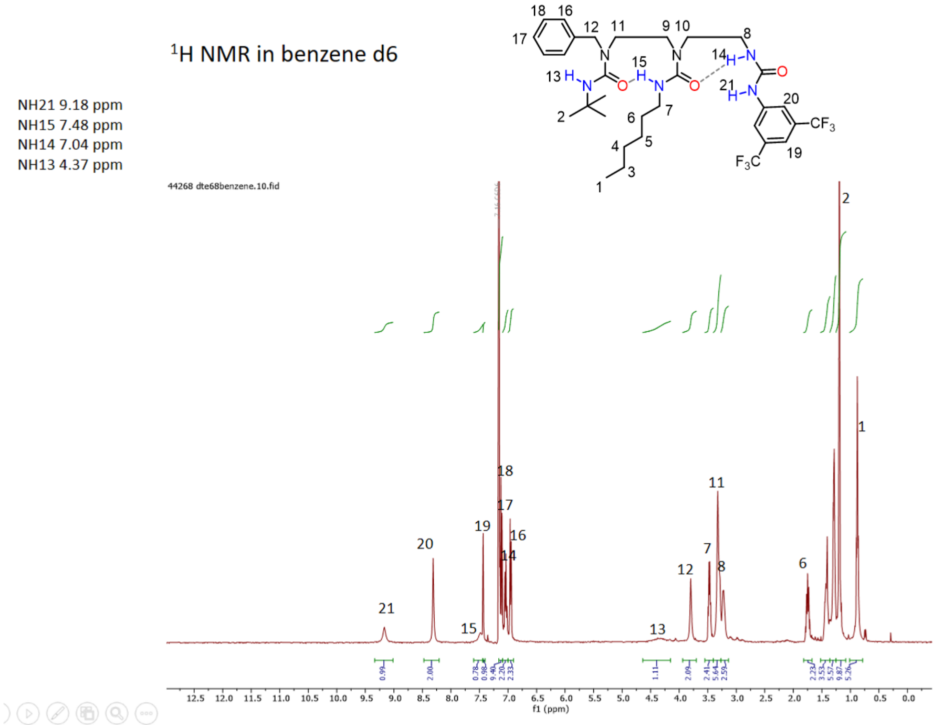

Figure S17: ^{1}H NMR spectrum of **2** in benzene d6 at 25 °C.

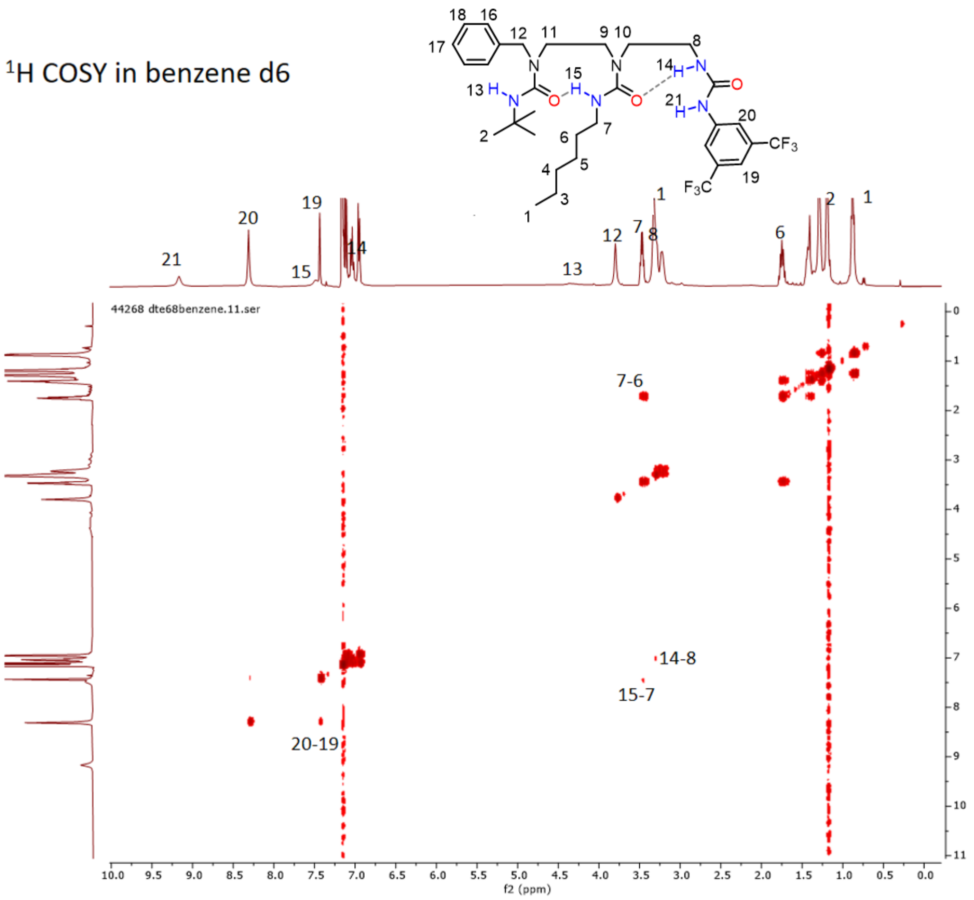

Figure S18: ¹H COSY NMR spectrum of **2** in benzene d6 at 25 °C.

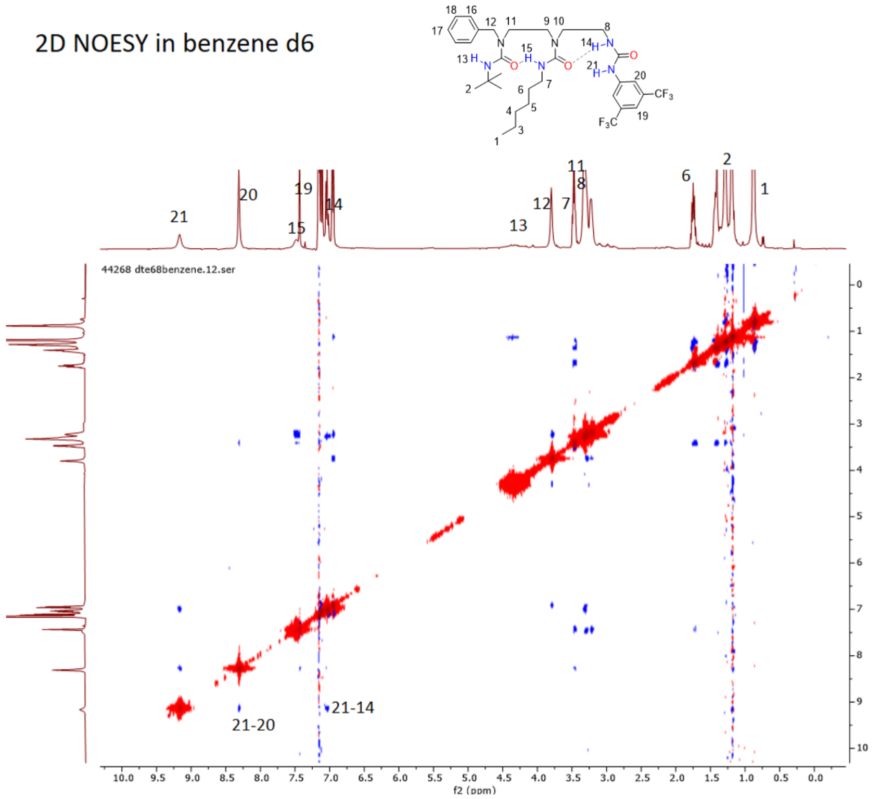

Figure S19: ¹H 2D NOESY NMR spectrum of **2** in benzene d6 at 25 °C.

5.2.4. Assignment of ^1H NMR signals in thf d8

^1H NMR spectrum of **2** was recorded in thf d8 to evaluate the influence of the solvent on the directionality of the hydrogen bond chain. ^1H COSY NMR experiment shows couplings between NH^{14} and H^8, allowing for the assignment of NH^{14} signal. 2D NOESY experiment shows correlation signals between NH^{21} and H^{20}, allowing for the assignment of NH^{21}. HSQC and HMBC experiments confirmed the assignment of signals. 2D NOESY NMR data do not offer information due to the broadness of the NH signals in that solvent.

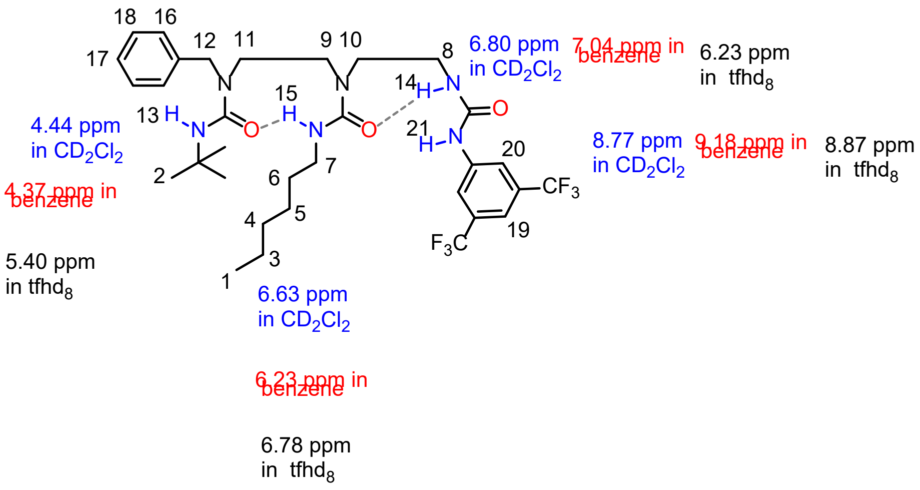

Figure S20: Comparison of the ^1H NMR chemical shift values of ureido NH signals for **2** in different solvents (CD_2Cl_2, benzene d6 and THF d8) at 25 °C.

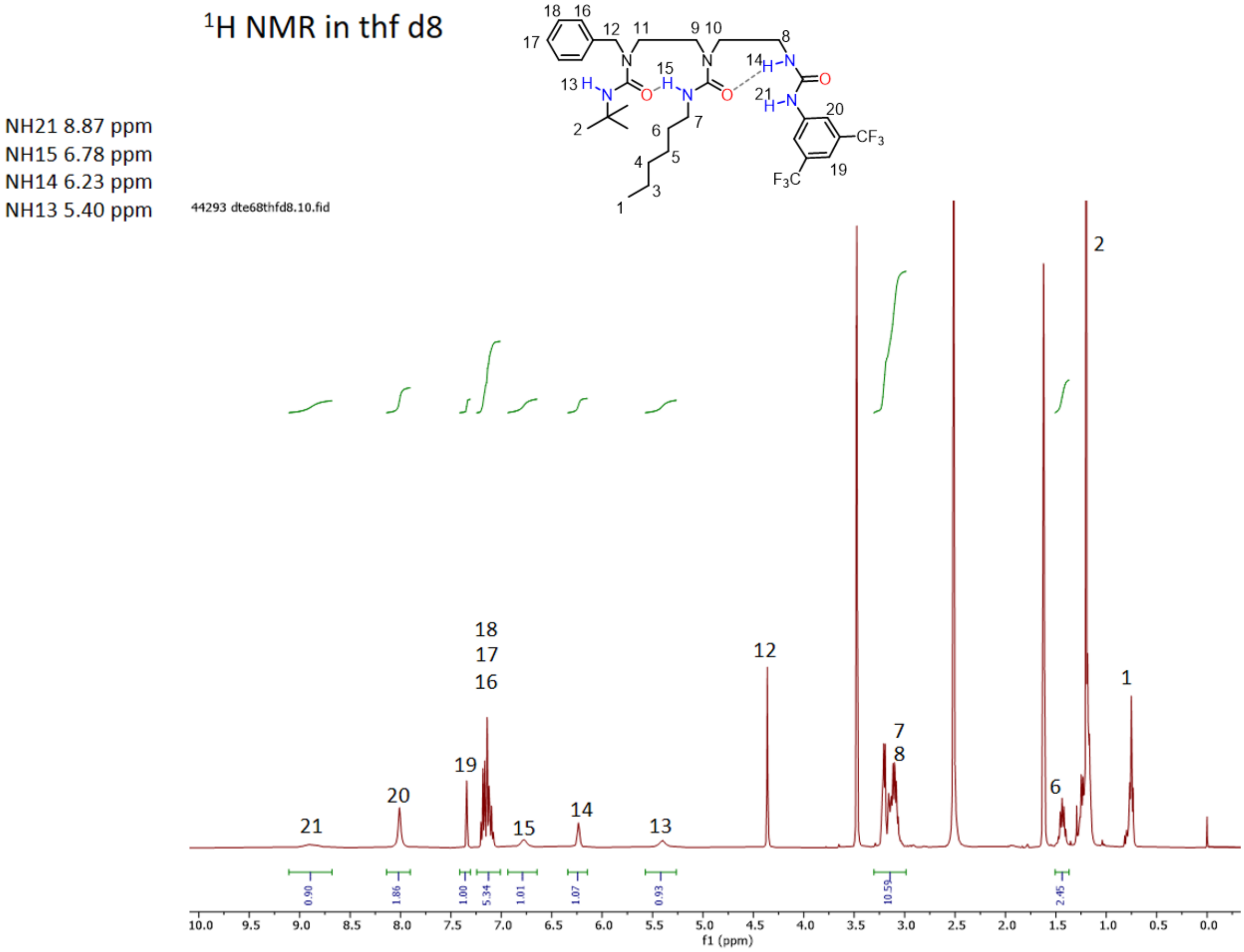

Figure S21: ^{1}H NMR spectrum of **2** in THF-d8 at 25 °C.

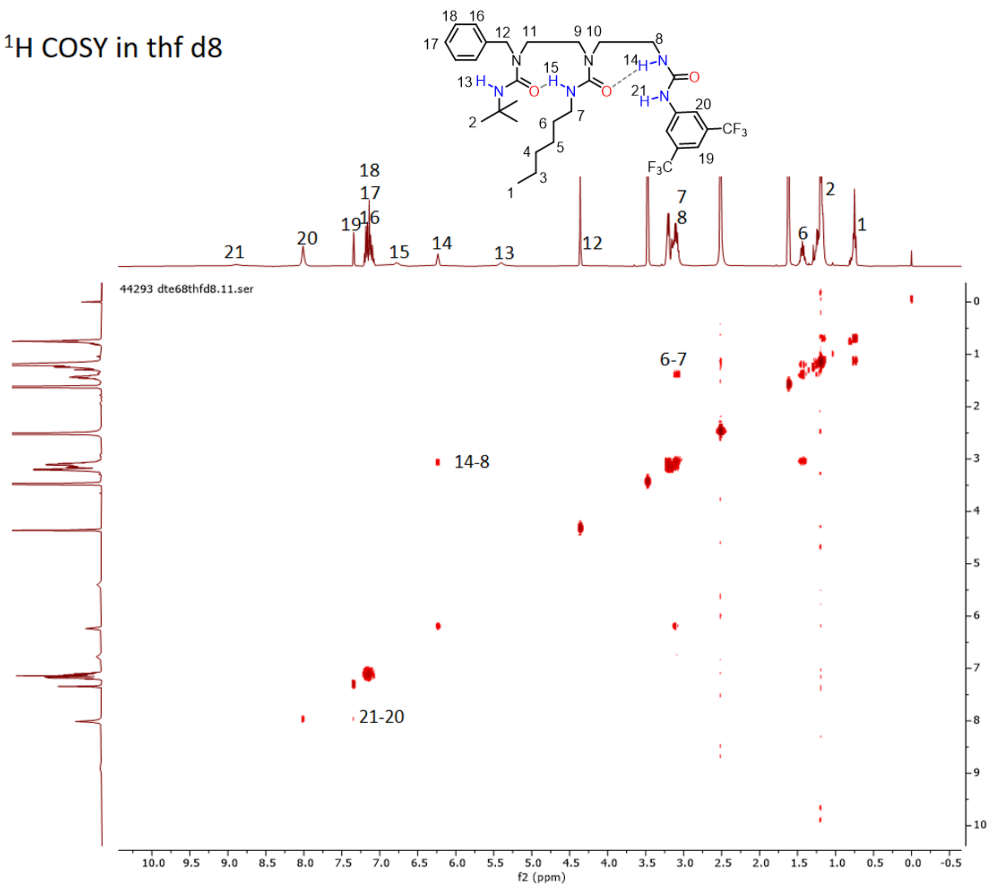

Figure S22: ^{1}H COSY NMR spectrum of **2** in THF-d8 at 25 °C.

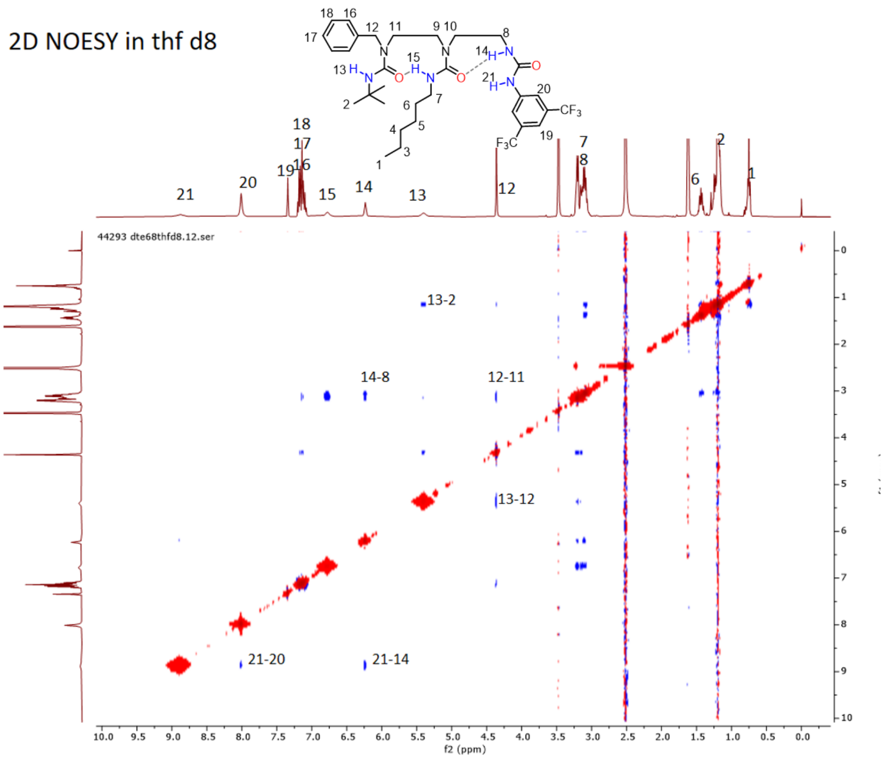

Figure S23: 2D NOESY NMR spectrum of **2** in thf d8 at 25 °C.

5.3. Compound 3

5.3.1. Assignment of ^{1}H NMR signals in CD_2Cl_2

^{1}H-^{1}H COSY NMR experiment shows coupling between H^6 and NH^{12} and between H^7 and NH^{13} allowing for the assignment of both NH^{13} and NH^{12} signals. NH^{11} gives a broad signal partially overlapping with CD_2Cl_2.

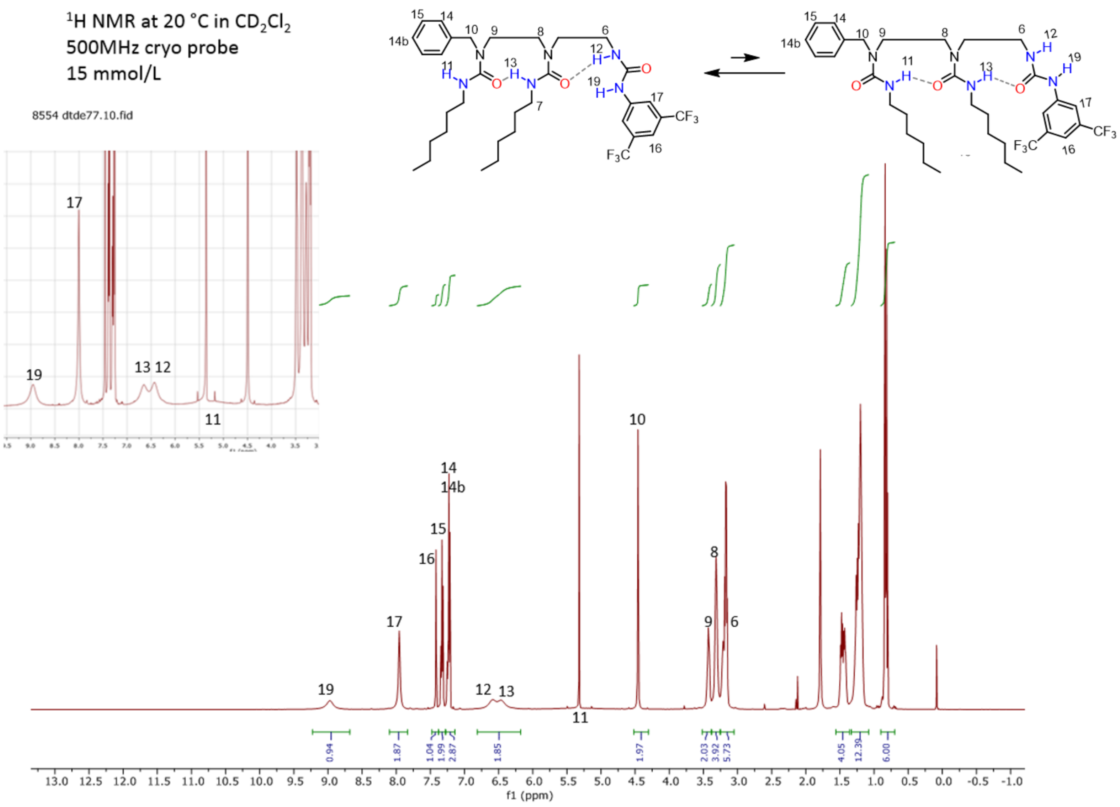

Figure S24: ^{1}H NMR spectrum of **3** (15 mM in CD_2Cl_2) at 25 °C (500 MHz).

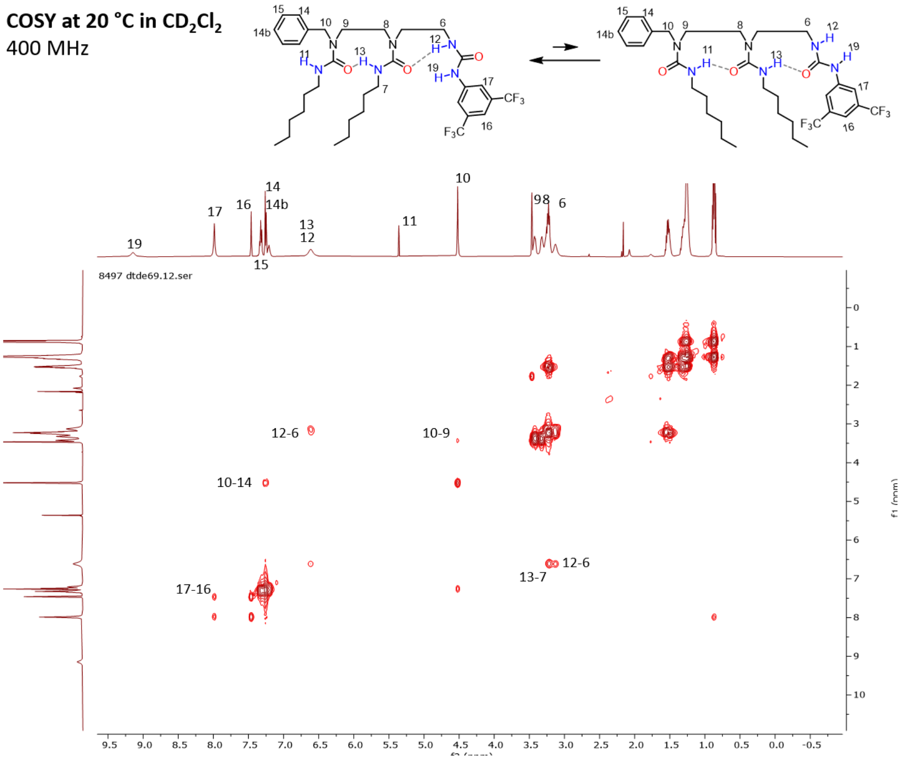

Figure S25: ^{1}H COSY NMR spectrum of **3** (15 mM in CD_2Cl_2) at 25 °C (400 MHz).

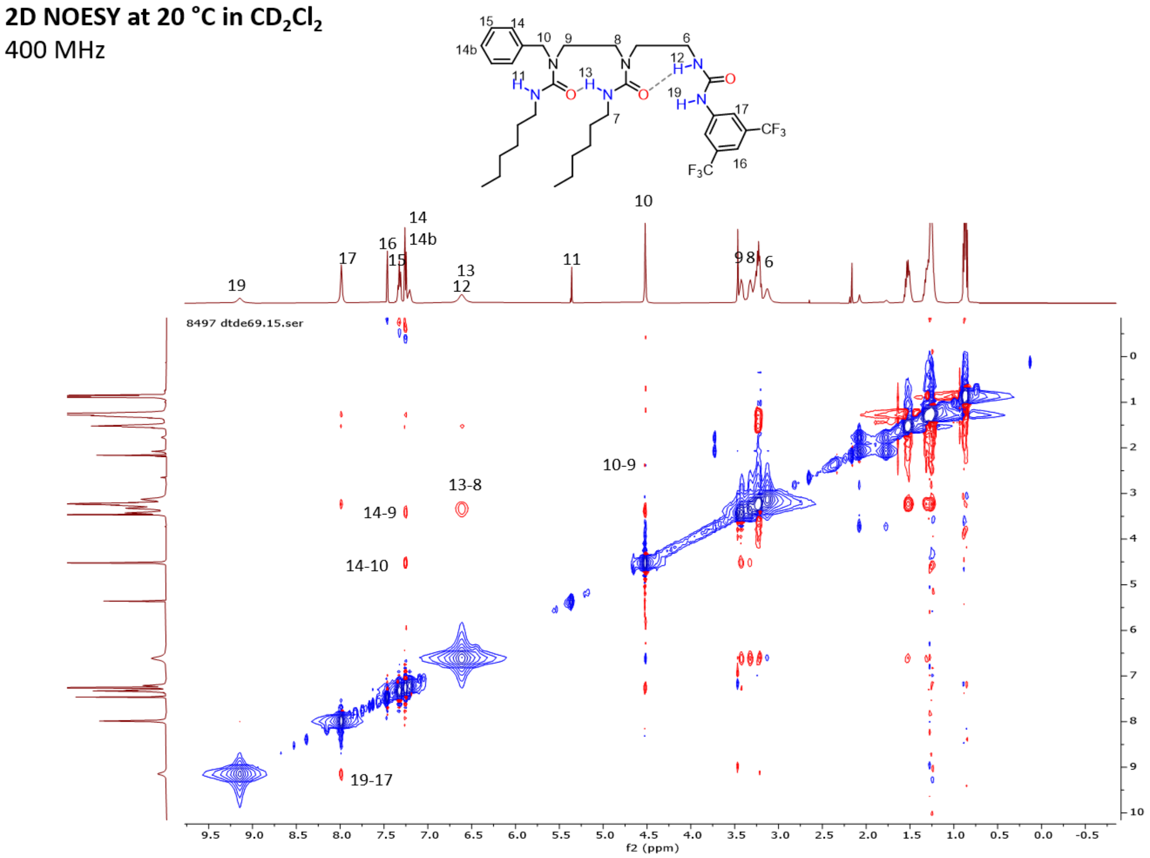

Figure S26: ^{1}H-^{1}H 2D NOESY NMR spectrum of **3** (15 mM in CD_2Cl_2) at 25 °C (400 MHz).

5.3.2. Assignment of directionality in CD_2Cl_2

The ^{1}H NMR chemical shift values of ureido NH^{11} (5.25 ppm), NH^{13} (6.44 ppm), NH^{12} (6.58 ppm), NH^{19} (8.97 ppm) at 25 °C in CD_2Cl_2 were compared to the ^{1}H NMR chemical shift values of non hydrogen-bonded and hydrogen-bonded ureido NH in similar topological environments. The values indicate that all the ureas in the chain are forming hydrogen bonds. Both NH^{13} and NH^{12} seem directly involved in hydrogen bonds with carbonyls while NH^{11} chemical shift is explained by indirect electronic perturbations caused by the hydrogen bond between its urea carbonyl with NH^{13}.

2D NOESY experiment shows correlation signal between NH^{13} and H^8, supporting a directionality as drawn. NH^{19} and NH^{11} signals are too broad to give nOe correlation signal at 25 °C. The last hexyl urea of the chain was replaced by a ^{i}Pr (4) or a ^{t}Bu urea (2) leading to sharper N*H*^{t}Bu NMR singlet signal instead of a triplet signal for N*H*CH_2.

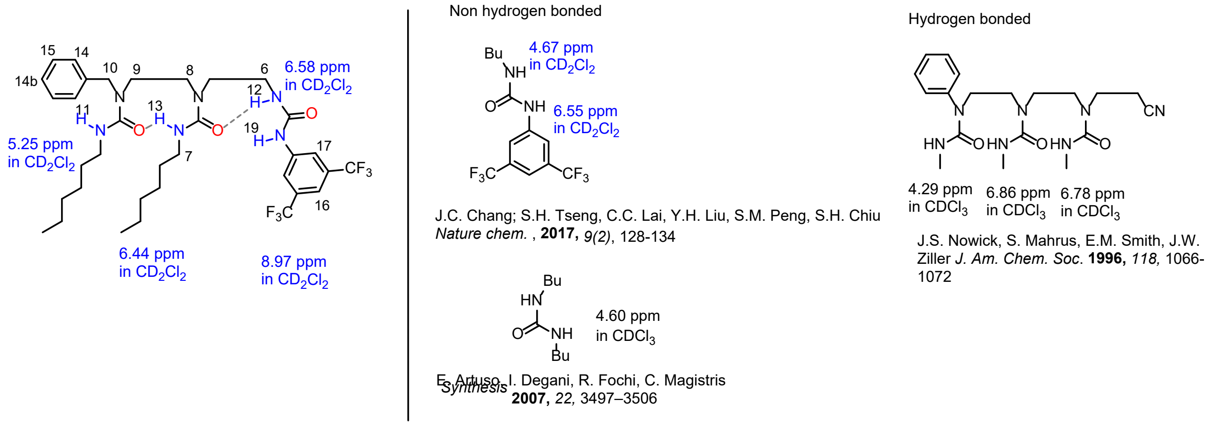

Figure S27: ^{1}H NMR chemical shift values of ureido NH for **3** in CD_2Cl_2 at 25 °C and published values of ureido NH in similar topological environments

5.4. Compound 4

5.4.1. Assignment of ^{1}H NMR signals in CD_2Cl_2

^{1}H-^{1}H COSY NMR experiment shows couplings between NH^m and H^f, NH^n and $H^{f'}$, allowing for the assignment of both NH^m and NH^n signals. ^{1}H-^{1}H 2D NOESY experiment shows correlation signals between NH^t and H^s, between NH^m and H^h allowing for assignment of NH^t signal and giving indication on the directionality of the hydrogen bond chain.

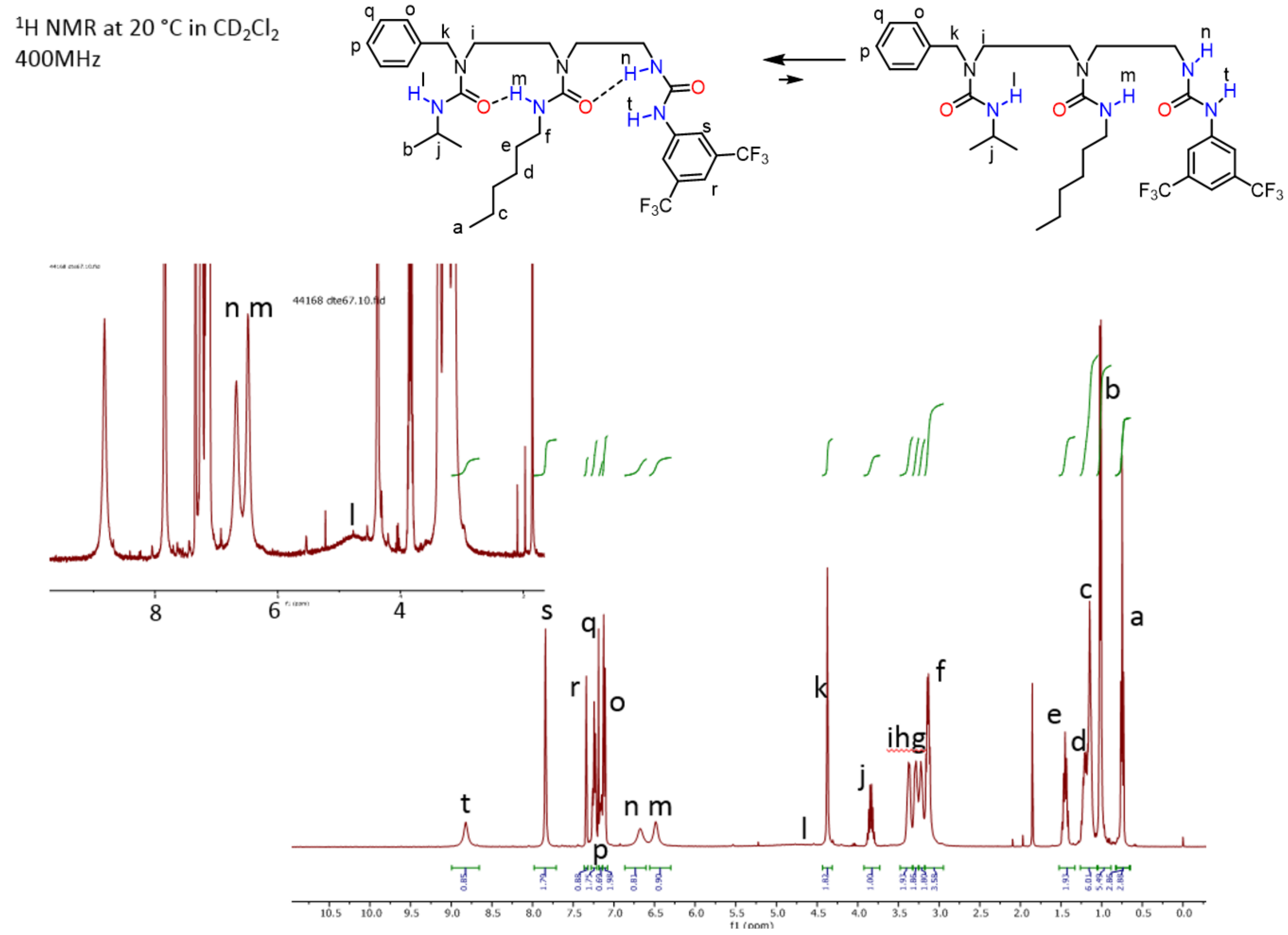

Figure S28: ^{1}H NMR spectrum of **4** in CD_2Cl_2 at 25 °C (400 MHz).

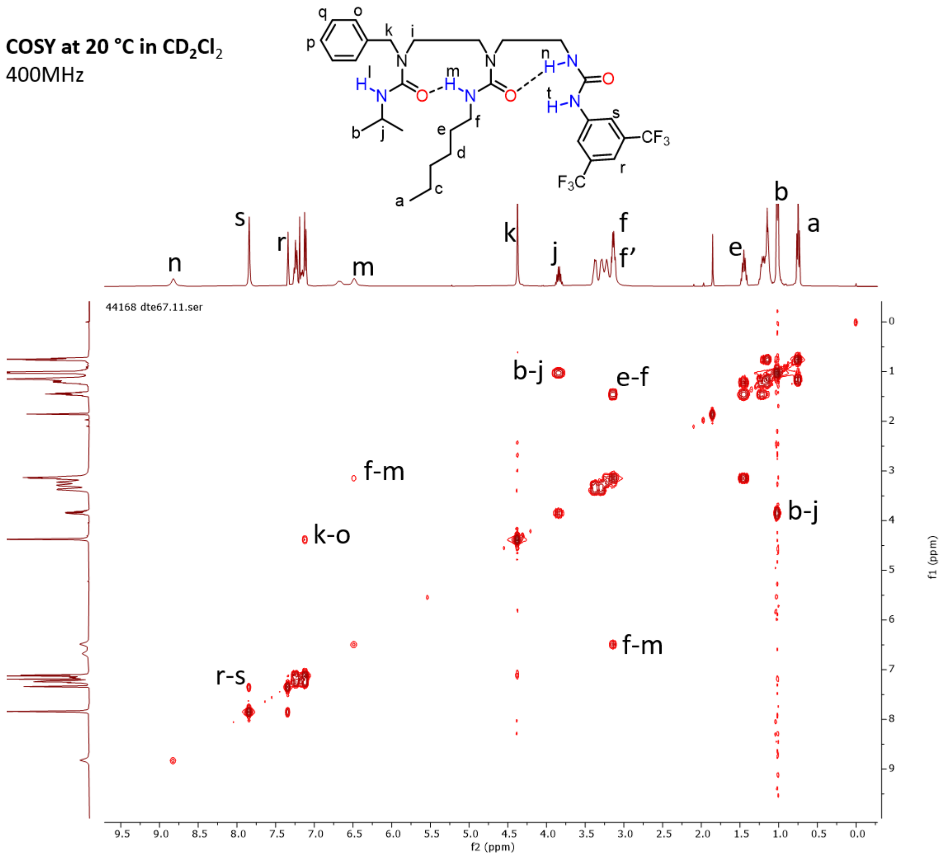

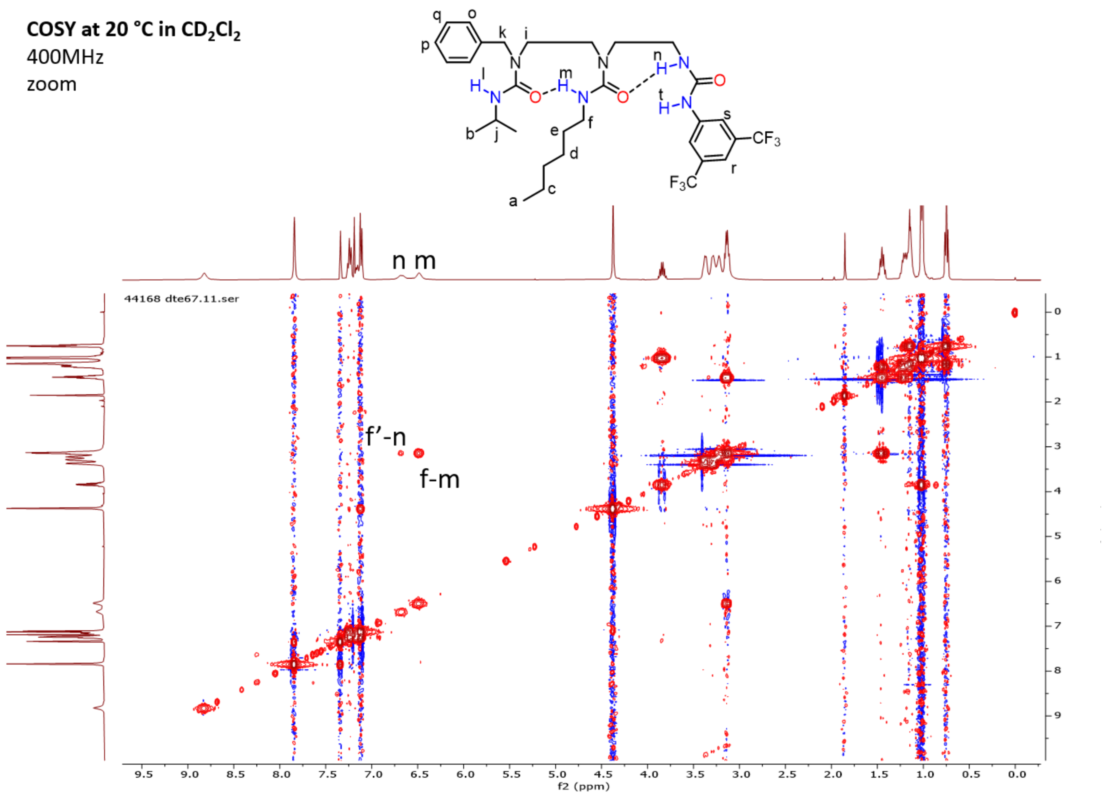

Figure S29: ^{1}H COSY NMR spectra of **4** in CD_2Cl_2 at 25 °C (400 MHz).

5.4.2. Assignment of directionality

The ^1H NMR chemical shift values of ureido NH for **4** (at 25 °C in CD_2Cl_2) (NH^t 8.81 ppm, NH^n 6.67 ppm, NH^m 6.48 ppm, NH^l 4.77 ppm) were compared with the published ^1H NMR chemical shift values of ureido NH in similar environments. It indicates that NH^l is not directly involved in a hydrogen bond, while both NH^m and NH^n are directly hydrogen bonded to carbonyls. A directionality of the hydrogen bond chain consistent with nOe correlation signal between NH^m and H^h is deduced. The signal for NH^l is too broad to see nOe correlation signal between NH^l and benzylic CH_2^k.

Dilution studies of compound **4** was carried out in CD_2Cl_2 at 25 °C, no significant change was observed in the values of ureido NH ^1H NMR chemical shifts: self-aggregation is low at those concentrations at 25 °C.

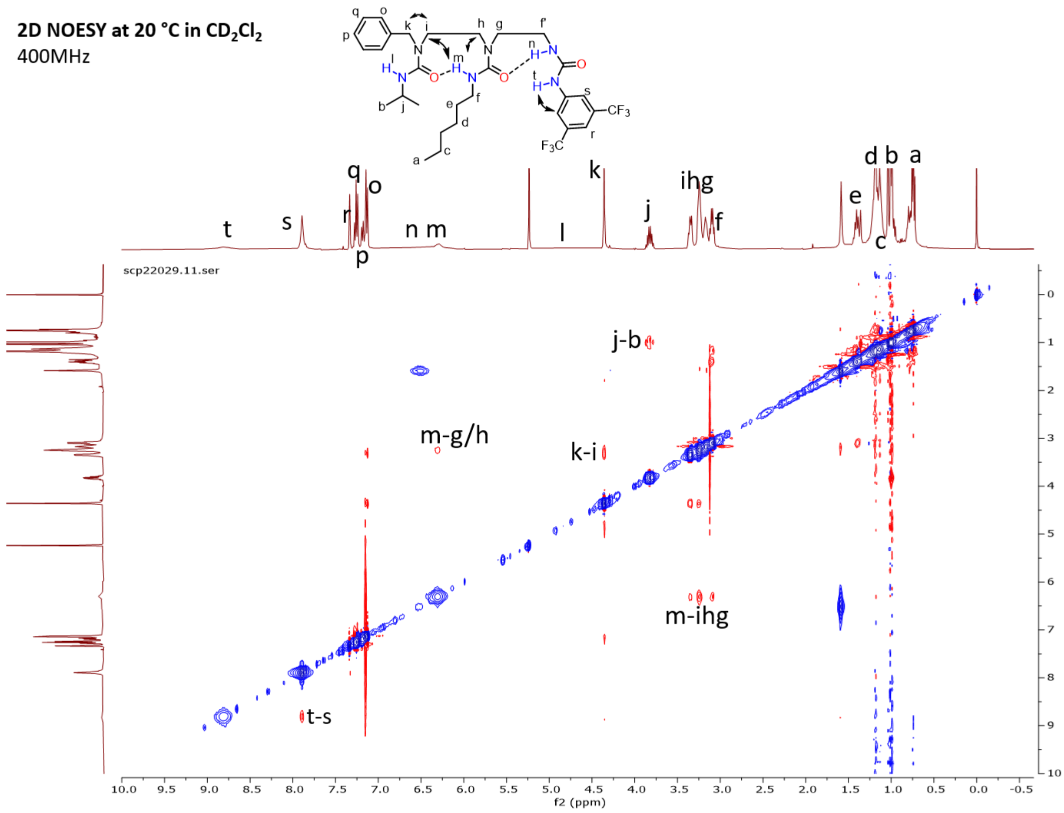

Figure S30: ^1H-^1H 2D NOESY NMR spectrum of **4** in CD_2Cl_2 at 25 °C (400 MHz).

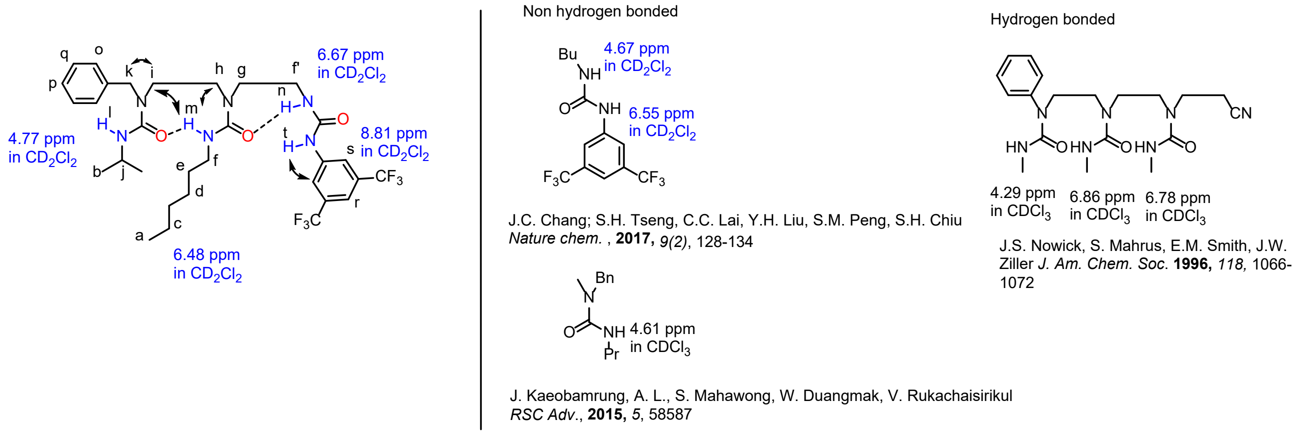

Figure S31: ^1H-^1H NMR chemical shift values of ureido NH for **4** in CD_2Cl_2 at 25 °C and published values of ureido NH in similar topological environments

5.4.3. Dilution study in CD_2Cl_2 at 25 °C

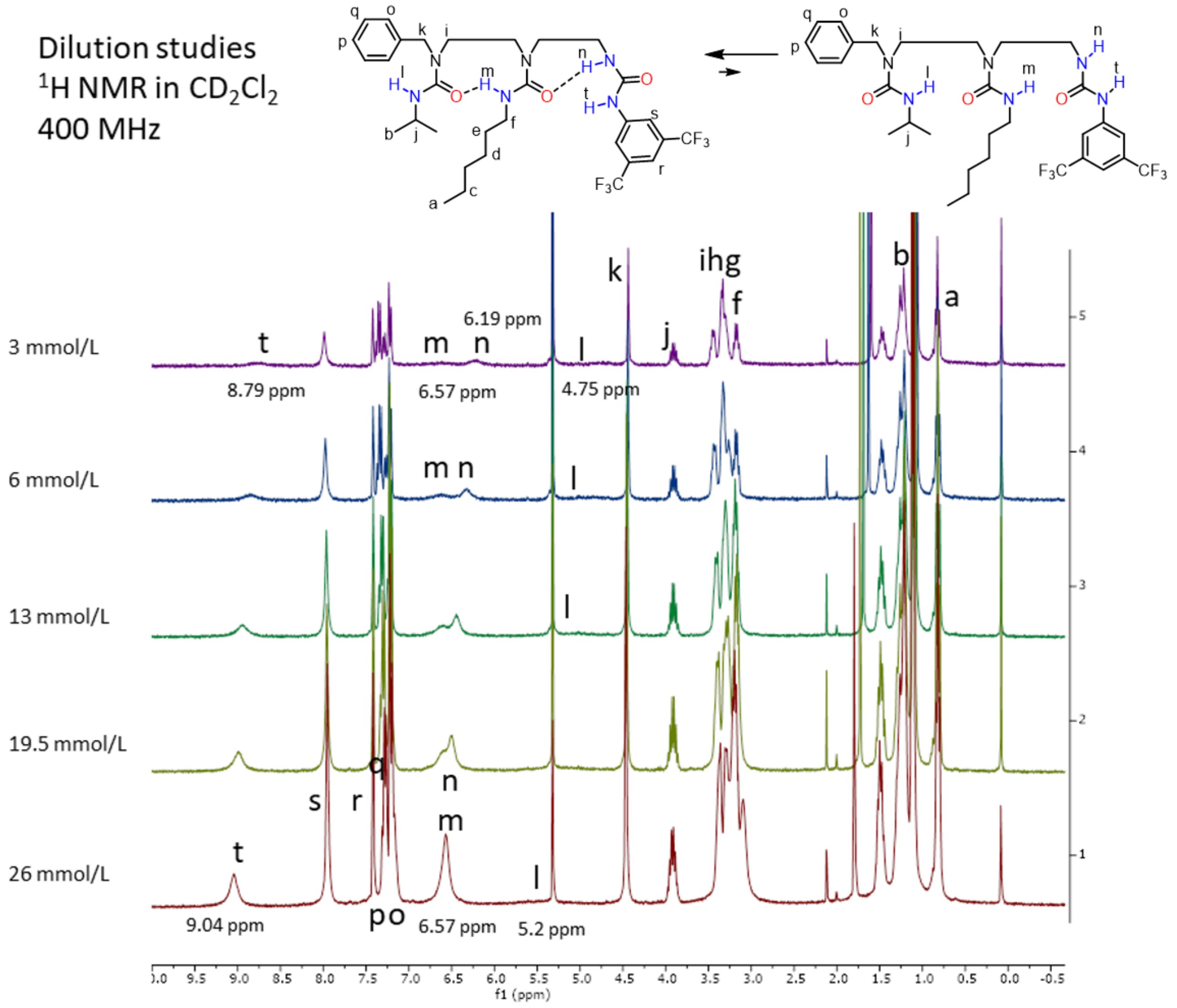

Figure S32: overlay of ^{1}H NMR spectra of **4** in CD_2Cl_2 at concentrations between 3 mM and 26 mM recorded (400 MHz) at 25 °C.

5.5. Compound 5

5.5.1. Assignment of ^{1}H NMR signals in CD_2Cl_2

^{1}H-^{1}H COSY NMR experiment shows couplings between NH signals at 7.05, 6.95, 6.76 ppm and CH_2 signals at 3.29 ppm, 3.20 ppm, NH^c signal at 6.27 ppm is too broad to show a coupling. 2D NOESY NMR experiment shows correlation signal between NH^e and NH^f, correlation signal between NH^e and H^{16} allowing for their assignment. Correlation signal between NH^a and ^{t}Bu, H^{15} and ^{t}Bu allow their assignment. The other HSQC, HMBC experiments confirm the assignment of the signals.

5.5.2. Assignment of directionality

The ^{1}H NMR chemical shifts values of the NH signals for **5** were compared with those measured for **2** (NH^f:6.77 ppm for **5** and 6.57 ppm for **2**, NH^a:4.30 ppm for **5** and 4.75 ppm for **2**, NH^e:9.1 ppm for **5**, 8.79 ppm for **2**). Those values are consistent with a directionality as drawn. nOe signals for **5** are too weak to be seen.

Dilution studies of compound **5** in CD_2Cl_2 from 12.3 mM to 4.1 mM shows little effect of dilution on the chemical shifts values of NH signals, the molecule is not self-aggregating at the concentrations of the study.

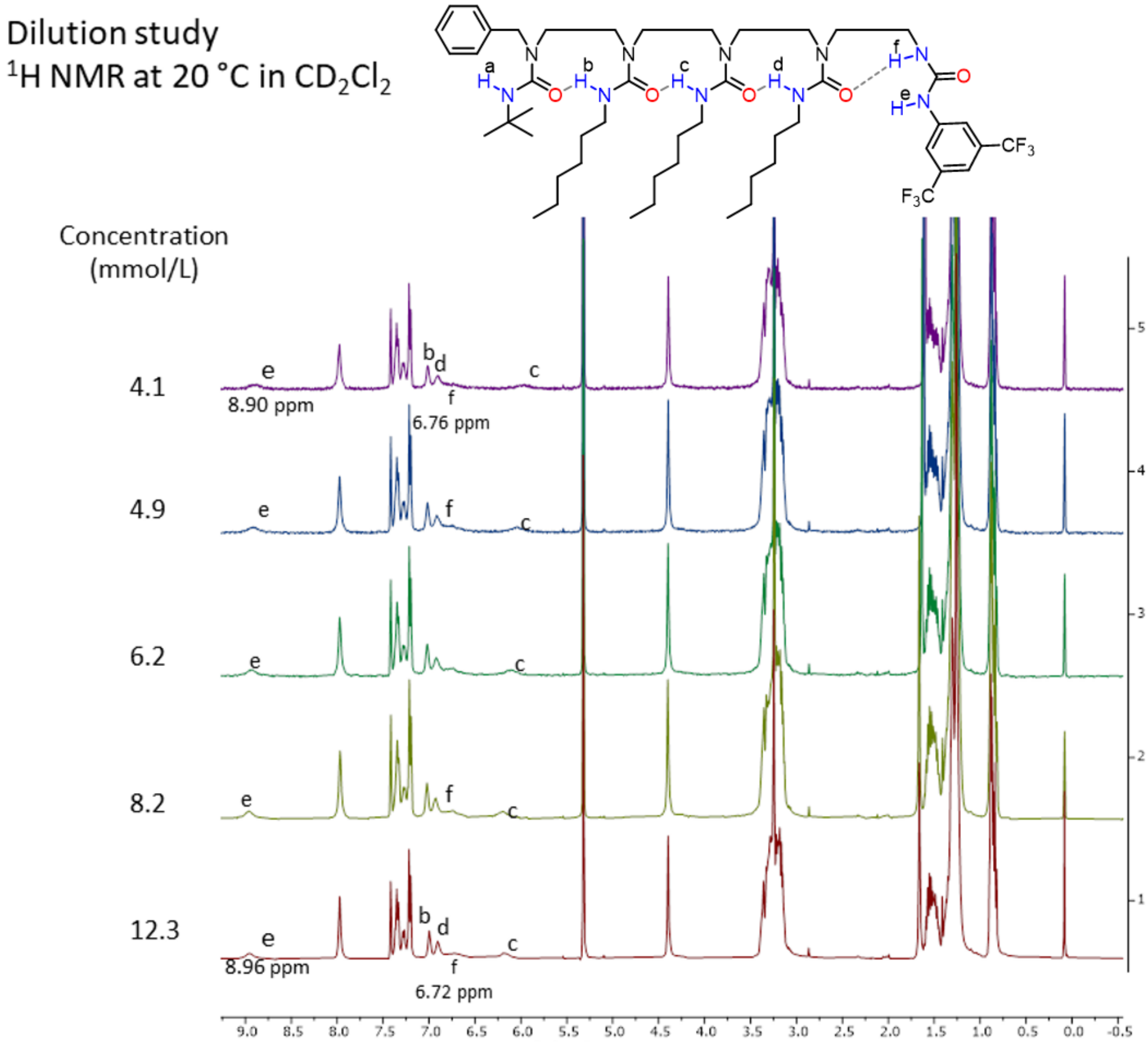

Figure S33: overlay of ^{1}H NMR spectra of **5** in CD_2Cl_2 at concentrations between 4.1 mM and 12.3 mM recorded (400 MHz) at 25 °C.

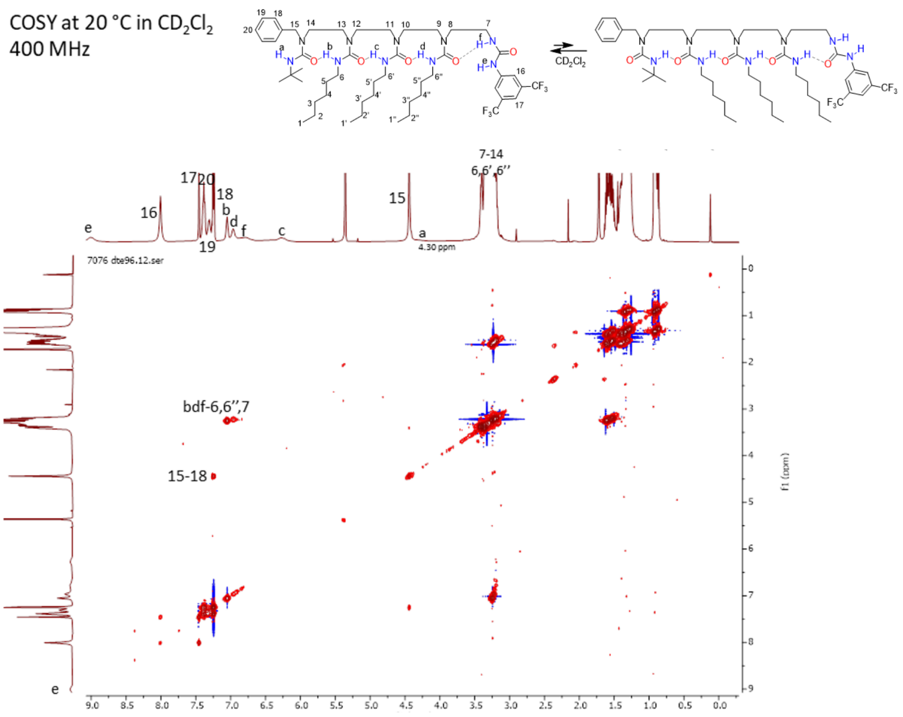

Figure S34: ^{1}H COSY NMR spectrum of **5** in CD_2Cl_2 at 25 °C (400 MHz)

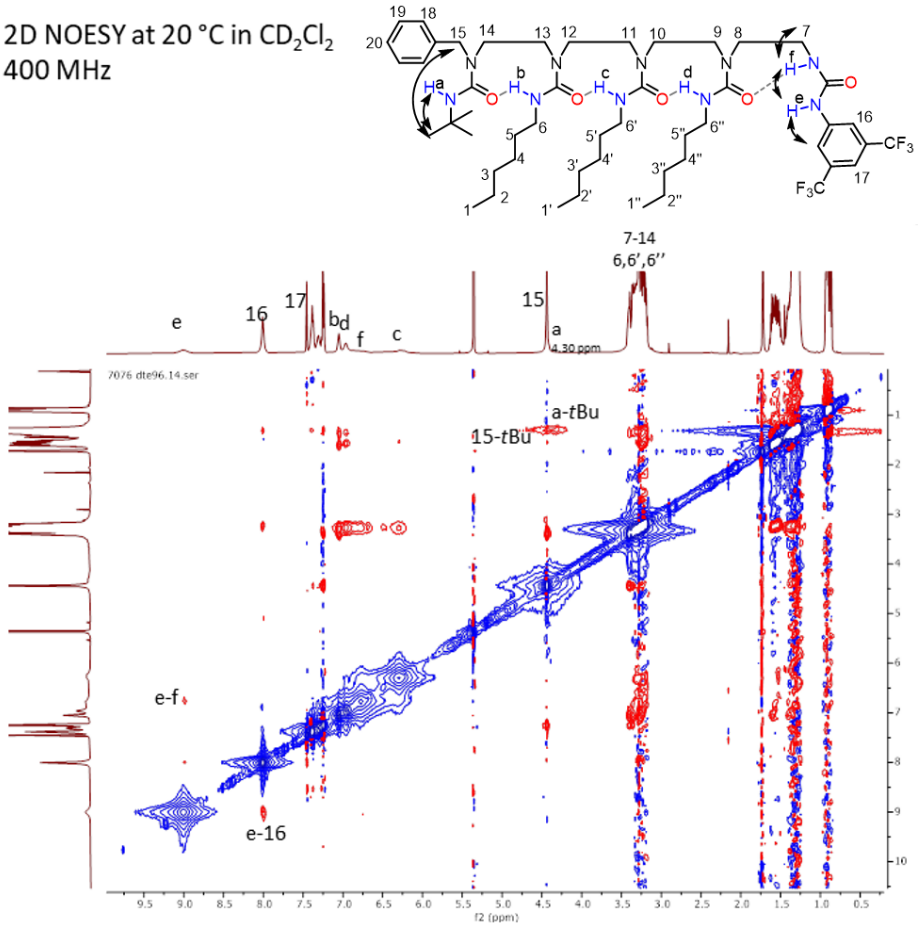

Figure S35: ^{1}H 2D NOESY NMR spectrum of **5** in CD_2Cl_2 at 25 °C (400 MHz)

6. Conformational response to anion binding

6.1. Compound 2

6.1.1. Titration with tetrabutylammonium diphenylphosphate in CD_2Cl_2 at 25 °C

- Monitoring by ^1H NMR experiment

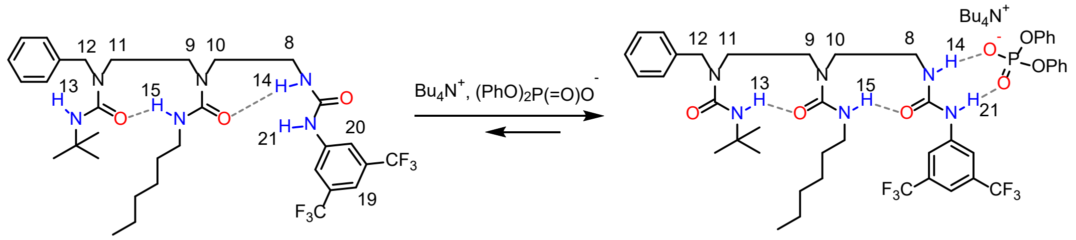

Titration of **2** (14.8 mM) in CD_2Cl_2 at 25 °C with increasing amounts of tetrabutylammonium diphenylphosphate (from 0 to 2.76 equivalents) was monitored by ^1H NMR (figure S36). The ligand was added as a solid so no variation of concentration occurs during the titration. The values of chemical shifts of the ureido NH of **2** upon addition of ligand are collected in Table SS1 and plotted in a graph as {chemical shift of NH = f(ligand added)} (figure S37).

Progressive downfield shift of both NH^{14} (CIS 1.01 ppm) and NH^{21} (CIS 1.64 ppm) signals are observed upon addition of ligand until 1 equivalent of ligand is added, the chemical shifts do not vary much after one equivalent of ligand is added. This is consistent with the formation of intermolecular hydrogen bonds between the ligand and the host at NH^{14} and NH^{21}.

The upfield chemical shift variations of NH^{15} (CIS 0.18 ppm) upon addition of ligand is smaller than the one observed for NH^{14} or NH^{21}, NH^{15} is involved in intramolecular hydrogen bonds with adjacent ureas no matter the directionality of the hydrogen bond chain so no large variation of chemical shift value for NH^{15} is expected upon change of hydrogen bond directionality.

The signal for NH^{13} progressively moves downfield (CIS 1.46 ppm) upon addition of 0 to 1 equivalent of ligand then the variation of chemical shift is minimal upon further addition of ligand. The observation is attributed to a global change of directionality of the hydrogen bond chain induced by the ligand binding at NH^{14} and NH^{21}.

An association constant was calculated for the binding of tetrabutylammonium diphenylphosphate to **2** (14.8 mM in CD_2Cl_2) by non-linear curve fitting analysis of the variation of chemical shift of ureido signal NH^{21} occuring during the titration, using a theoretical binding isotherm for 1:2 binding http://app.supramolecular.org/bindfit/view/d14901a0-d58b-4ea6-b0da-d83d385bfb13. Ureido NH^{14} was overlapped during most of the titration and could not be used. A value of binding constant of 480 +/- 29% was obtained. Titration at lower concentrations to determine more accurately a binding constant value was not possible as NH signals become difficult to see at lower concentrations.

An association constant was also calculated by non-linear curve fitting analysis of variation of relative intensities of nOe signals of NH^{13} and CH^{12} with irradiation at H^{11} using a theoretical binding isotherm for 1:1 binding http://app.supramolecular.org/bindfit/view/fcc64856-eac6-412e-b132-ef7b6c2e55f2. A value of association constant of 539 +/- 252% was found. The values or similar order of magnitude suggest a direct correlation between the binding event and the variation of nOe signal.

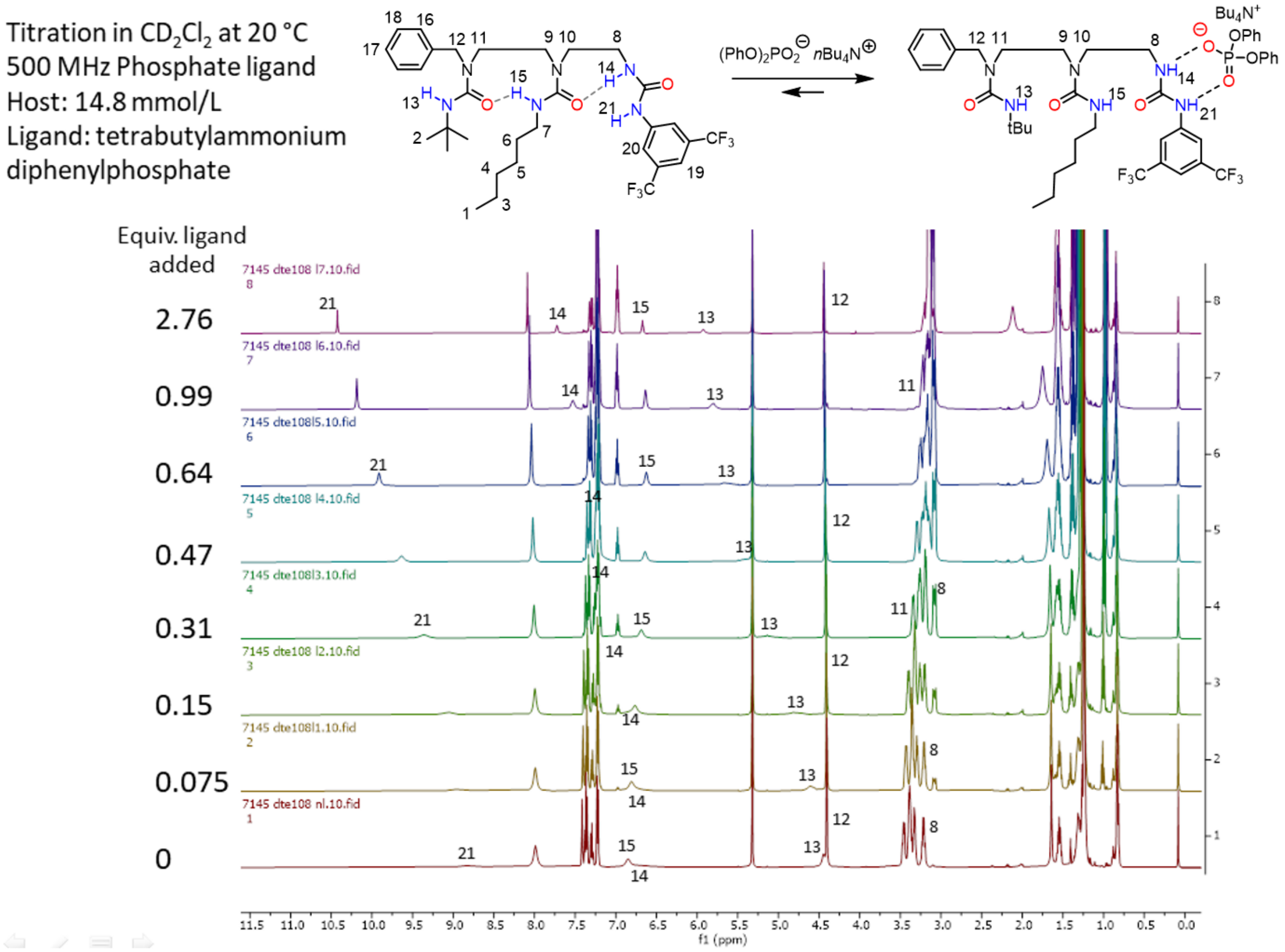

Figure S36: overlay of ^{1}H NMR spectra of **2** (14.8 mM in CD_2Cl_2) in the presence of increasing amounts of tetrabutylammonium diphenylphosphate from 0 to 2.76 equivalents, recorded at 25 °C (500 MHz)

Concentration of host (mol/L)	Concentration of phosphate ligand (mol/L)	Chemical shift NH^{21} (ppm)	Chemical shift NH^{14} (ppm)	Chemical shift NH^{15} (ppm)	Chemical shift NH^{13} (ppm)
0.0148	0	8.78	6.71	6.85	4.44
0.0148	0.00111	8.91	-	6.8	4.6
0.0148	0.00222	9.06	-	6.76	4.78
0.0148	0.004588	9.36	-	6.69	5.13
0.0148	0.006956	9.63	-	6.64	5.42
0.0148	0.009472	9.91	-	6.62	5.64
0.0148	0.014652	10.19	7.53	6.64	5.8
0.0148	0.040848	10.42	7.72	6.67	5.9
**CIS (ppm)**		**1.64**	**1.01**	**-0.18**	**1.46**

Table SS1. chemical shifts of ^{1}H NH signals recorded during the NMR titration of **2** (14.8 mM in CD_2Cl_2) with increments of tetrabutylammonium diphenylphosphate at 25 °C (500 MHz).

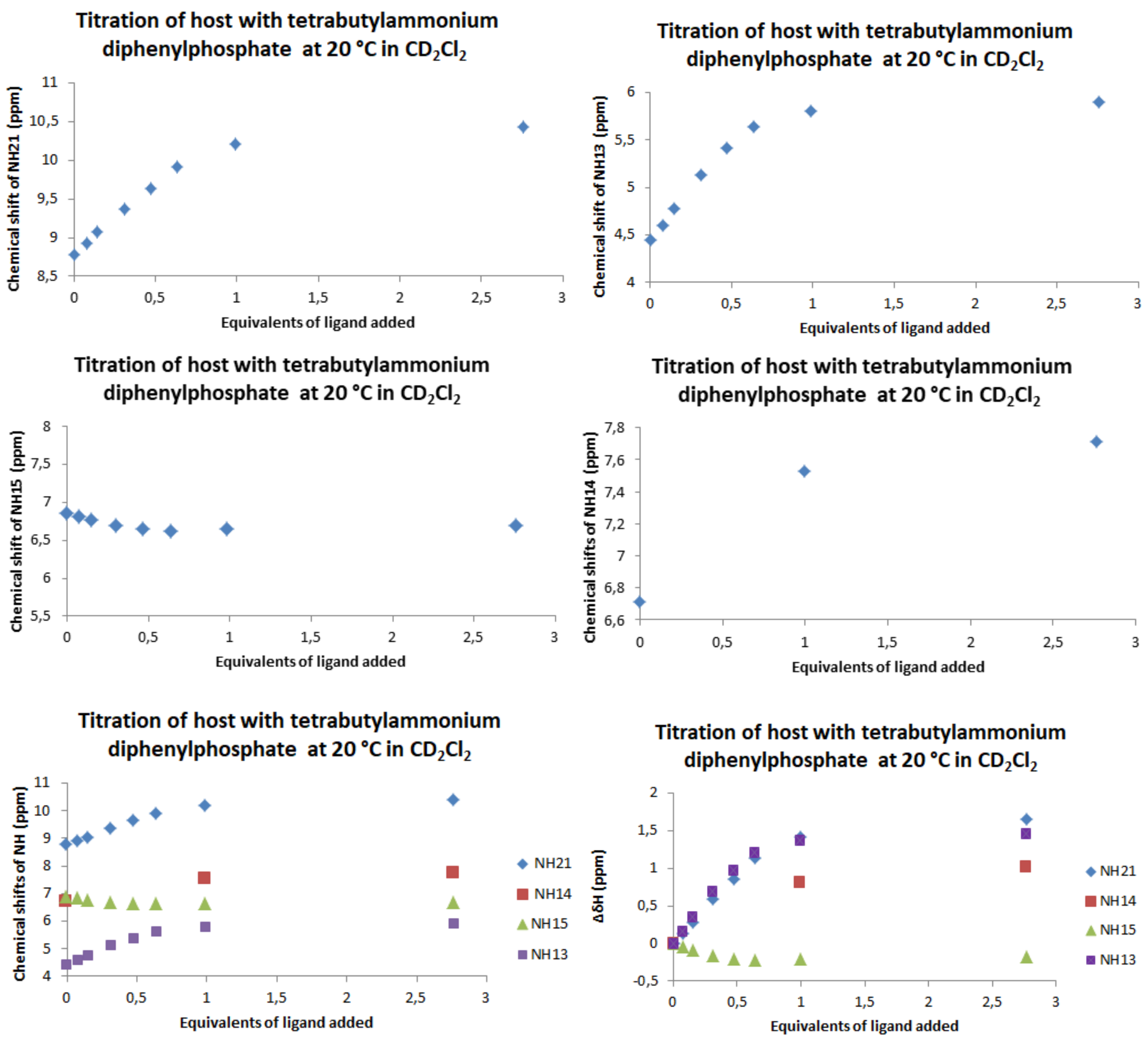

Figure S37. plots of variation of chemical shifts of NH signals and plot of chemical induced shifts of NH signals of **2** (14.8 mM in CD_2Cl_2) upon addition of increments of tetrabutylammonium diphenylphosphate, recorded by ^{1}H NMR at 25 °C (500 MHz).

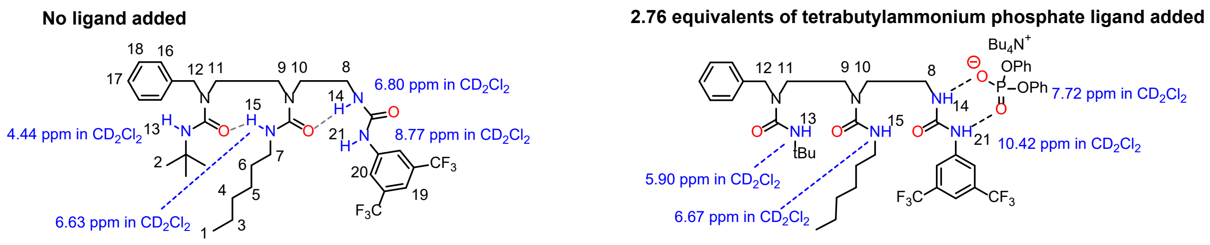

Figure S38: ^{1}H NMR chemical shifts of **2** NH signals (14.8 mM in CD_2Cl_2) before and after addition of 2.75 equivalents of tetrabutylammonium diphenylphosphate at 25 °C.

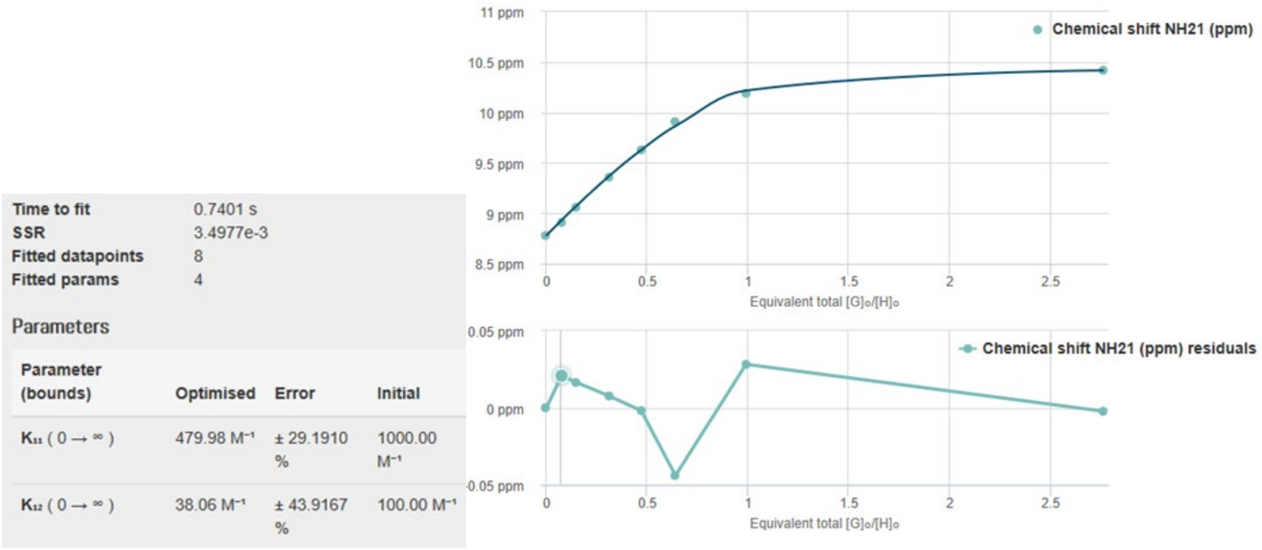

Figure 39: non-linear curve fitting analysis of the experimental titration data of **2** (14.8 mM in CD_2Cl_2) with increments of tetrabutylammonium diphenylphosphate recorded at 25 °C (400 MHz) using a theoretical binding isotherm for 1:2 binding http://app.supramolecular.org/bindfit/view/d14901a0-d58b-4ea6-b0da-d83d385bfb13

- Assignment of directionality change by NOE experiments

Further evidence of a global hydrogen-bond chain directionality switch was gathered using 2D NOESY (figure S40). 2D NOESY of **2** in CD_2Cl_2 at 25 °C without ligand displays a cross peak between NH^{13} and H^{12}, nOe experiment irradiating $H^{8/9/10/11}$ displays a cross peak with H^{12} and another cross peak of weak intensity between H^{11} with NH^{13}. In sharp contrast, 2D NOESY experiment of the same sample of 2 mixed with 2.76 equivalents of tetrabutylammonium diphenylphosphate shows no cross peak between NH^{13} and H^{12}, a cross peak of high intensity between H^{11} and NH^{13}. Those results offer strong support for a reversal in the global directionality of the hydrogen bond chain induced by addition of tetrabutylammonium diphenylphosphate.

The titration of 2 (14.8 mM) in CD_2Cl_2 at 25 °C with tetrabutylammonium diphenylphosphate was followed using nOe NMR experiment. H^{11} was irradiated and the variations of the intensities of nOe signals with H^{12} and with H^{13} were observed as a function of the increments of tetrabutylammonium diphenylphosphate added. A change in directionality of the hydrogen bond chain should result in a gradual decrease of the distance between H^{13} and H^{11}, leading to a gradual increase in the intensity of nOe signal with H13 compared to the constant intensity of nOe signal with H^{12}. The ratio (intensity of nOe signals of H^{11} with H^{13})/(intensity of nOe signal of H^{11} with H^{12}) as a function of the number of equivalents of ligand added is presented figure S41. With no ligand the ratio could not be measured due to overlap of signals hampering accurate integration. Whether possibilities of complications in integrating nOe signals involving acidic protons exist, such as a change in the rate of exchange of the NH due to addition of the ligand, or a change in the relaxation rates of the various nuclei in the system due to addition of the ligand (this may be seen as a broadening / sharpening of the NOE signal), our experiment shows a clear progressive increase of nOe signal intensity between H^{11} and NH^{13} upon addition of ligand. This result is consistent with a progressive global switch in the hydrogen bond directionality induced by coordination of phosphate anion at NH^{14} and NH^{21}.

-

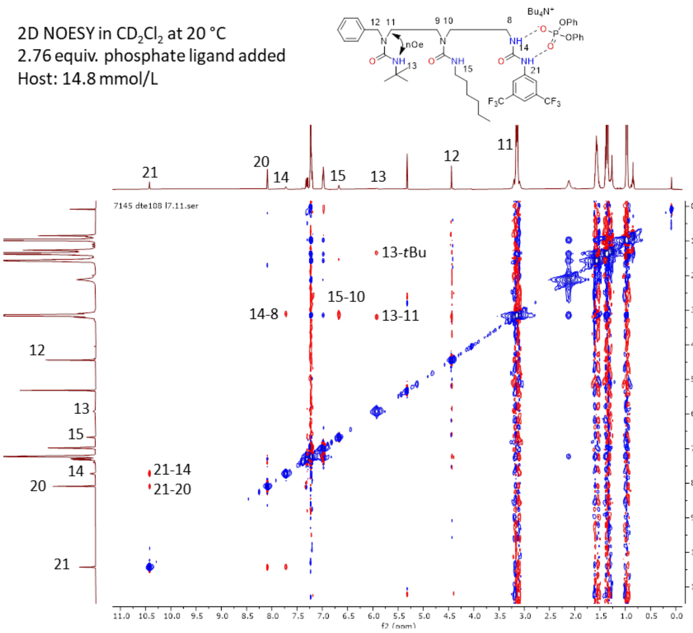

Figure S40: ^1H-^1H 2D NOESY NMR spectrum of **2** (14.8 mM in CD_2Cl_2) in the presence of 2.76 equivalents of tetrabutylammonium diphenylphosphate at 25 °C (500 MHz).

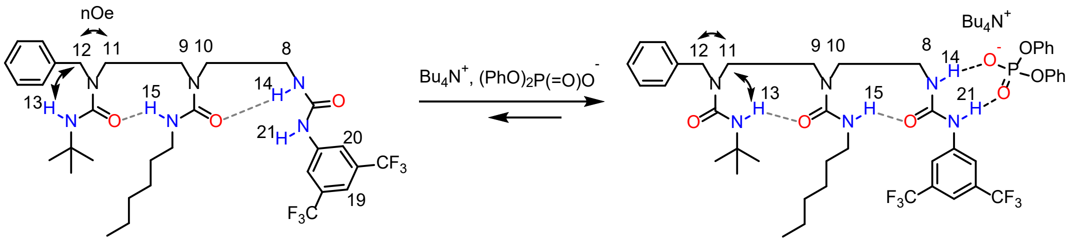

Equiv of ligand added	nOe intensity NH	nOe intensity CH_2Ph	Ratio
0			overlap
0.144	0.23	1	0.19
0.31	0.7	1	0.41
0.47	1.47	1	0.59
0.64	2.1	1	0.68
0.99	2.47	1	.72

Table SS2. relative intensities of ^1H nOe signals recorded during the NMR titration of **2** (14.8 mM in CD_2Cl_2) with increments of tetrabutylammonium diphenylphosphate at 25 °C (500 MHz).

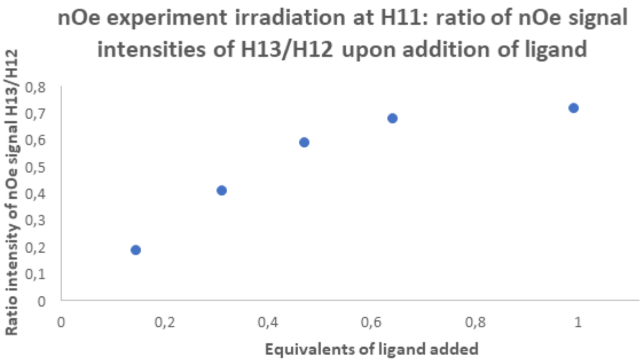

Figure S41: plots of variation of relative intensity of nOe signal of NH^{13} compared to nOe signal of NH^{12} in **2** (14.8 mM in CD_2Cl_2) upon addition of increments of tetrabutylammonium diphenylphosphate recorded by ^1H NMR at 25 °C (500 MHz) with irradiation at H^{11}

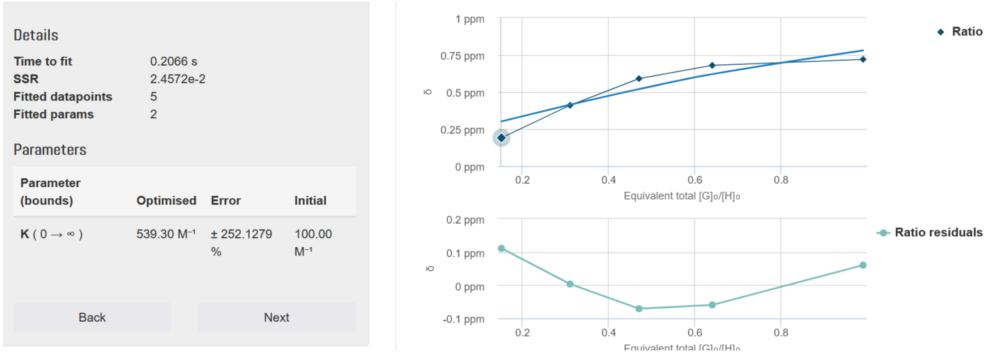

Figure S42: non-linear curve fitting analysis of variation of relative intensity of nOe signal of NH[13] compared to nOe signal of NH[12] in **2** (14.8 mM in CD_2Cl_2) upon addition of increments of tetrabutylammonium diphenylphosphate recorded by ^{1}H NMR at 25 °C (500 MHz) with irradiation at H[11] using a theoretical binding isotherm for 1:1 binding http://app.supramolecular.org/bindfit/view/fcc64856-eac6-412e-b132-ef7b6c2e55f2

- Characterisation of complex formation by DOSY and HRMS

Using a Varian VNMRS 500MHz spectrometer, DOSY OneShot NMR experiments were run at 25 °C on compound 2 in CD_2Cl_2 (17 mM) without ligand, then with 1.8 equivalents of tetrabutylammonium di(*p*-butoxyphenyl)phosphate added to the same NMR sample, to characterise the formation of a complex. The variation of the diffusion coefficient from 21.5.10^{-10} m^2.s^{-1} (no ligand) to 16.10^{-10} m^2.s^{-1} (1.8 equiv of ligand) is consistent with the formation of a complex between compound 2 and the ligand in CD_2Cl_2 at 20°C.

Mass spectrometry analysis of a sample of 2 + tetrabutylammonium diphenylphosphate ligand (1.5 equiv.) using Synapt G2S Waters nanospray TOF MS ES+ experiment also shows the formation of a 1:1 complex between the host and the ligand, **HRMS** (ES, positive ion mode) – *m/z* for $[C_{76}H_{126}F_6N_8O_7P]^+$ 1407.9391, observed 1407.9397 (figure S46).

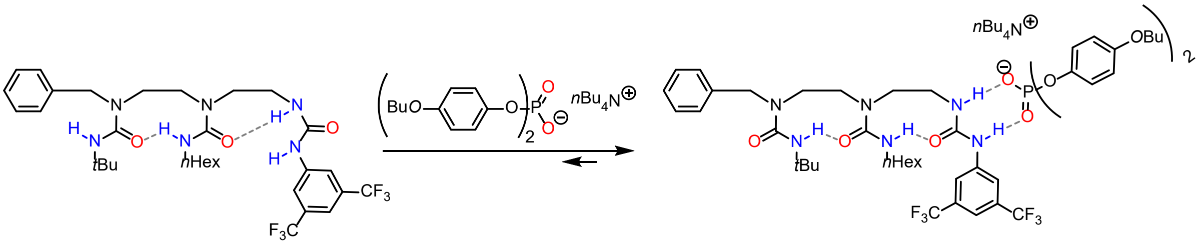

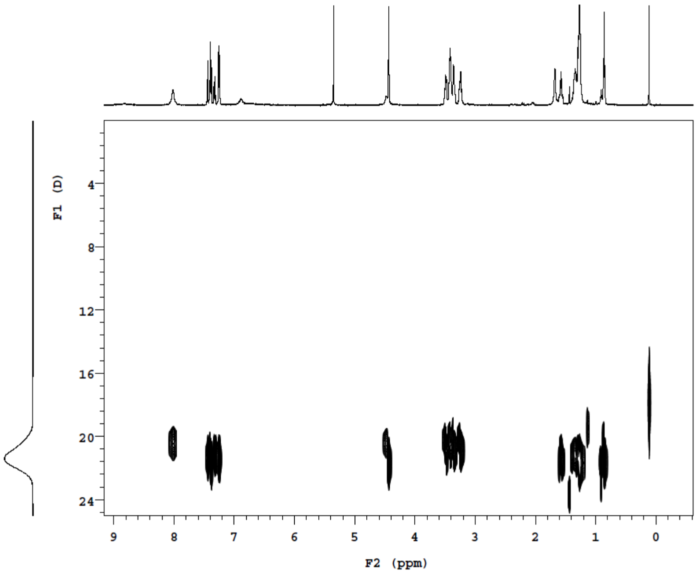

Figure S43: DOSY OneShot ^{1}H NMR spectrum of compound **2** (17 mM in CD_2Cl_2) at 25 °C (Varian VNMRS 500MHz spectrometer) with a measured diffusion coefficient of 21.5.10^{-10} m^2.s^{-1}

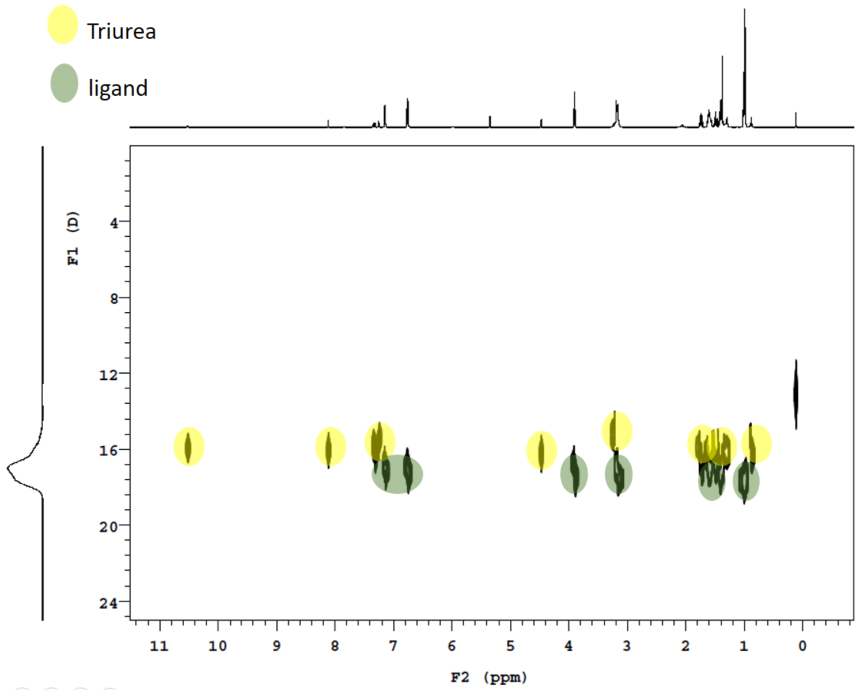

Figure S44: DOSY OneShot ^{1}H NMR spectrum of compound **2** (17 mM in CD_2Cl_2, same tube as previous analysis) in the presence of 1.8 equivalents of tetrabutylammonium di(*p*-butoxyphenyl)phosphate recorded at 25 °C (Varian VNMRS 500MHz spectrometer) with a measured diffusion coefficient of 15.5 $.10^{-10}$ $m^2.s^{-1}$. Binding of the ligand to 2 causes downfield chemical shifts of NHs.

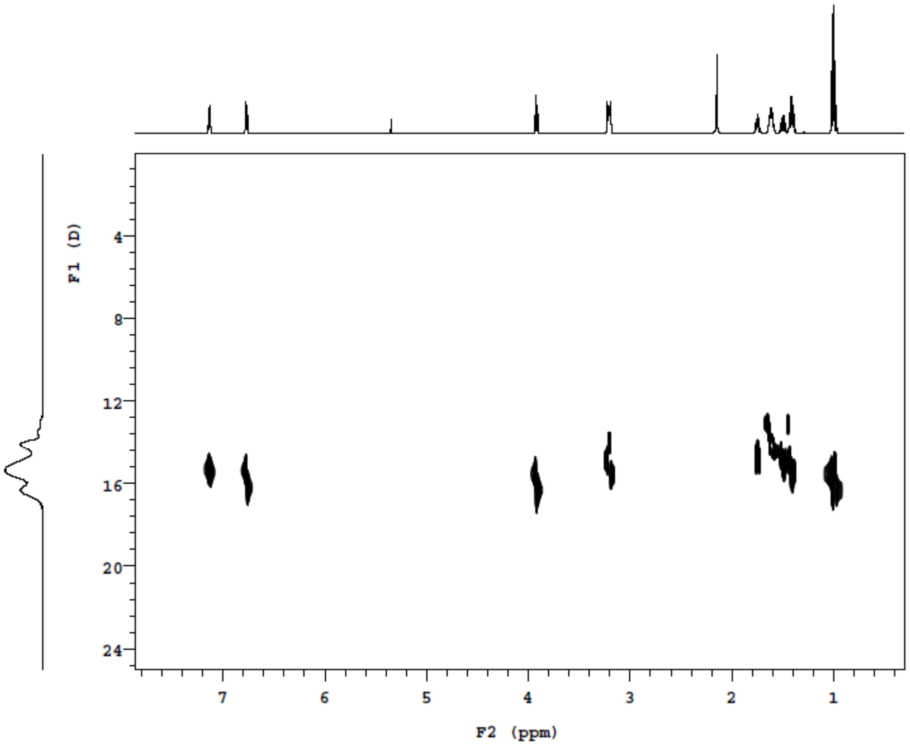

Figure 45: DOSY OneShot ^{1}H NMR spectrum of tetrabutylammonium di(*p*-butoxyphenyl)phosphate in CD_2Cl_2 recorded at 25 °C (Varian VNMRS 500MHz spectrometer) with a measured diffusion coefficient of $16.2.10^{-10}$ $m^2.s^{-1}$

a

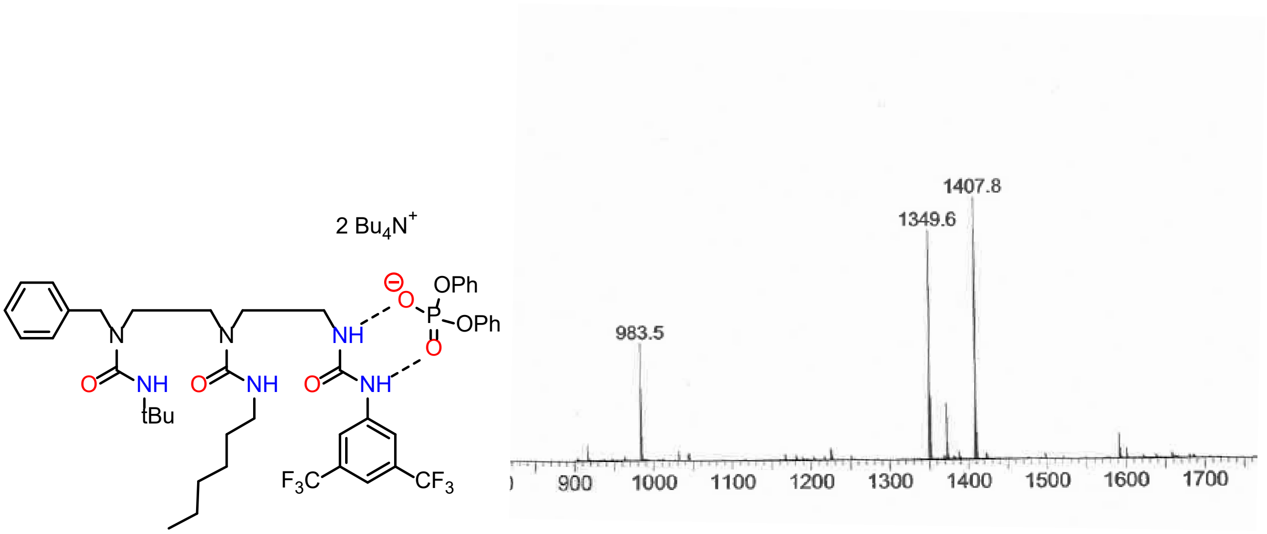

b

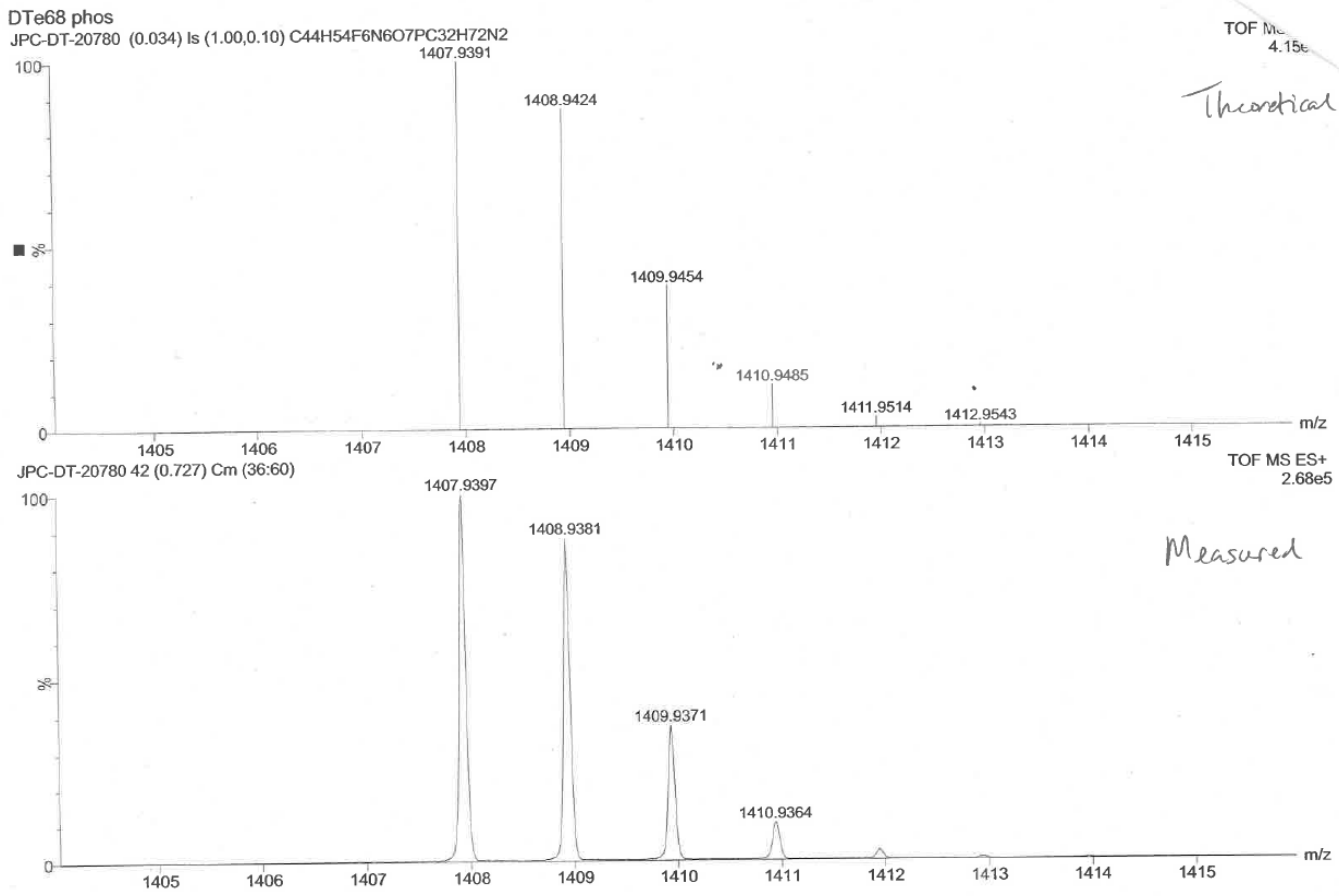

Figure S46: a ESI-MS (+ve) spectrum of 2 + $(ArO)_2PO_2^-$, 2 Bu_4N^+ **b** experimental and simulated isotopic distribution of mass spectrum of **2** – $(ArO)_2PO_2^-$, 2 Bu_4N^+

6.1.2. Titration with tetrabutylammonium acetate in CD_2Cl_2 at 25 °C

- Monitoring by ^{1}H NMR experiment

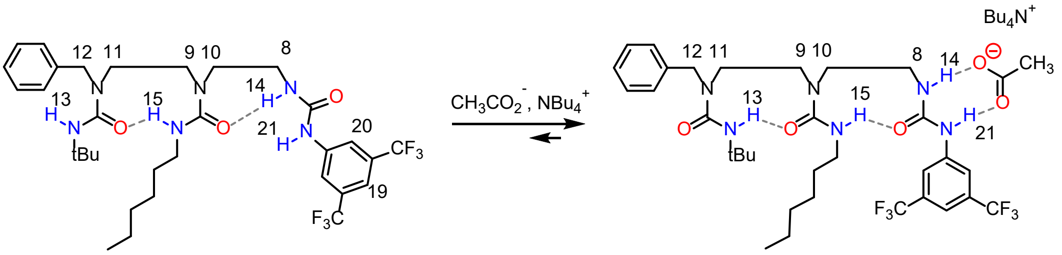

Titration experiment of **2** (17.8 mM) in CD_2Cl_2 at 25 °C with increasing amounts of tetrabutylammonium acetate (from 0 to 1.73 equivalents) was monitored by ^{1}H NMR (figure S47). The ligand was added as a solid so no variation of concentration occurs during the titration. The values of chemical shifts of the ureido NH upon addition of tetrabutylammonium acetate are collected in Table S3 and plotted in a graph as {chemical shift of NH = f(ligand added)} (figure S49).

Progressive downfield shift of the chemical shift values both NH^{14} (CIS 3.08 ppm) and NH^{21} (CIS 3.07 ppm) signals are observed upon addition of acetate ligand, the variations of chemical shifts occur essentially from 0 to 1 equivalent of ligand added, further addition of ligand has little effect on the chemical shifts. This observation is consistent with the formation of intermolecular hydrogen bonds between the ligand and the host at those positions.

The upfield chemical shift variations of NH^{15} (CIS 0.24 ppm) upon addition of ligand is comparatively smaller, NH^{15} is involved in intramolecular hydrogen bonds with adjacent ureas no matter the directionality of the hydrogen bond chain so no important variation of chemical shift value of NH^{15} is expected upon change of hydrogen bond directionality.

The signal for NH^{13} moves downfield (CIS 1.47 ppm), the chemical shifts essentially occur from 0 to 1 equivalent of ligand added. The observation is consistent with a change of global directionality of the hydrogen bond chain controlled by the binding of acetate at the terminus of the chain.

Without ligand, 2D NOESY NMR experiment on host 2 in CD_2Cl_2 at 25 °C shows a cross peak of high intensity between NH^{13} and H^{12}, and nOe experiment irradiating $H^{8/9/10/11}$ shows a cross peak with H^{12} and a cross peak of weak intensity with H^{13}. In contrast, after addition of 3.5 equivalents of tetrabutylammonium acetate to the same NMR sample, the 2D NOESY data displays a cross peak of weak intensity between NH^{13} and H^{12}, and a cross peak of high intensity between NH^{13} and H^{11}. Those results are consistent with a reversal in the global directionality of the hydrogen bond chain.

An association constant for the binding between the acetate ligand and both NH^{14} and NH^{21} was estimated by non-linear curve fitting analysis of the experimental titration data and comparing the results with theoretical binding isotherms for 1:1, 1:2, 2:1 binding modes using supramolecular.org, equation for a 1:2 binding gave $K_{1:1}$ = 841 +/- 25%, $K_{1:2}$ = 37 +/-38% (http://app.supramolecular.org/bindfit/view/79944229-d8c7-4b82-83cd-d2102bee6e0d). Titration at lower concentrations to determine more accurately a binding constant value was not possible as NH signals become difficult to see at lower concentrations.

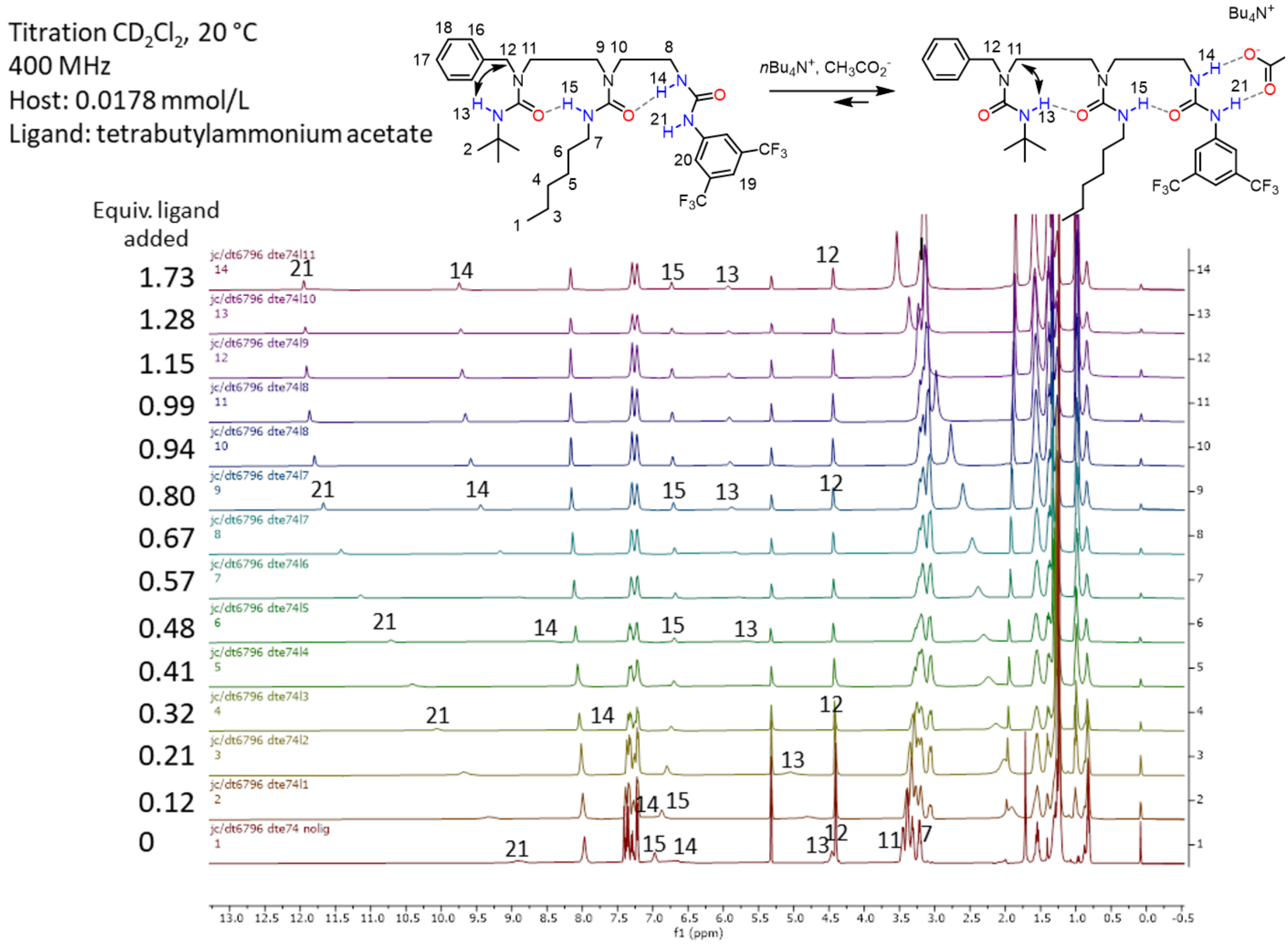

Figure S47: overlay of ^{1}H NMR spectra of **2** (17.8 mM in CD_2Cl_2) in the presence of increasing amounts of tetrabutylammonium acetate from 0 to 1.73 equivalents, recorded at 25 °C (400 MHz).

Concentration of host (mol/L)	Concentration of acetate ligand (mol/L)	Chemical shift NH^{21} (ppm)	Chemical shift NH^{14} (ppm)	Chemical shift NH^{15} (ppm)	Chemical shift NH^{13} (ppm)
0,0178	0	8.88	6.67	6.97	4,46
0,0178	0,002136	9.3	7.01	6.87	4,8
0,0178	0,003738	9,67		6,8	5,06
0,0178	0,005696	10,06		6,74	
0,0178	0,007298	10.41	8.09	6.7	5,49
0,0178	0,008544	10.71	8.43	6.69	5,62
0,0178	0,010146	11.15	8.86	6.68	5,76
0,0178	0,011926	11.42	9.16	6.69	5,83
0,0178	0,01424	11.67	9.44	6.71	5,8
0,0178	0,016732	11.8	9.58	6.74	5,9
0,0178	0,017622	11.87	9.66	6.72	5,92
0,0178	0,02047	11.91	9.7	6.73	5,92
0,0178	0,022784	11.93	9.72	6.73	5,92
0,0178	0,030794	11.95	9.75	6.73	5,93
**CIS (ppm)**		**3.07**	**3.08**	**-0.24**	**1.47**

Table S3. Chemical shifts of ^{1}H NMR NH signals recorded during the titration of **2** (17.8 mM in CD_2Cl_2) with increments of tetrabutylammonium acetate, recorded at 25 °C (400 MHz).

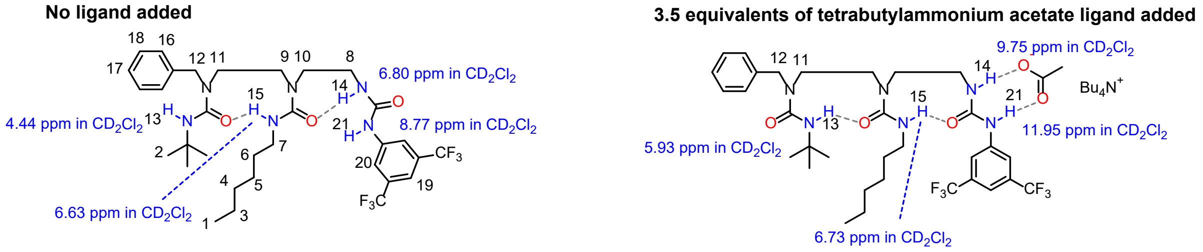

Figure S48: ^{1}H NMR chemical shifts of **2** NH signals (17.8 mM in CD_2Cl_2) before and after addition of 1.73 equivalents of tetrabutylammonium acetate at 25 °C.

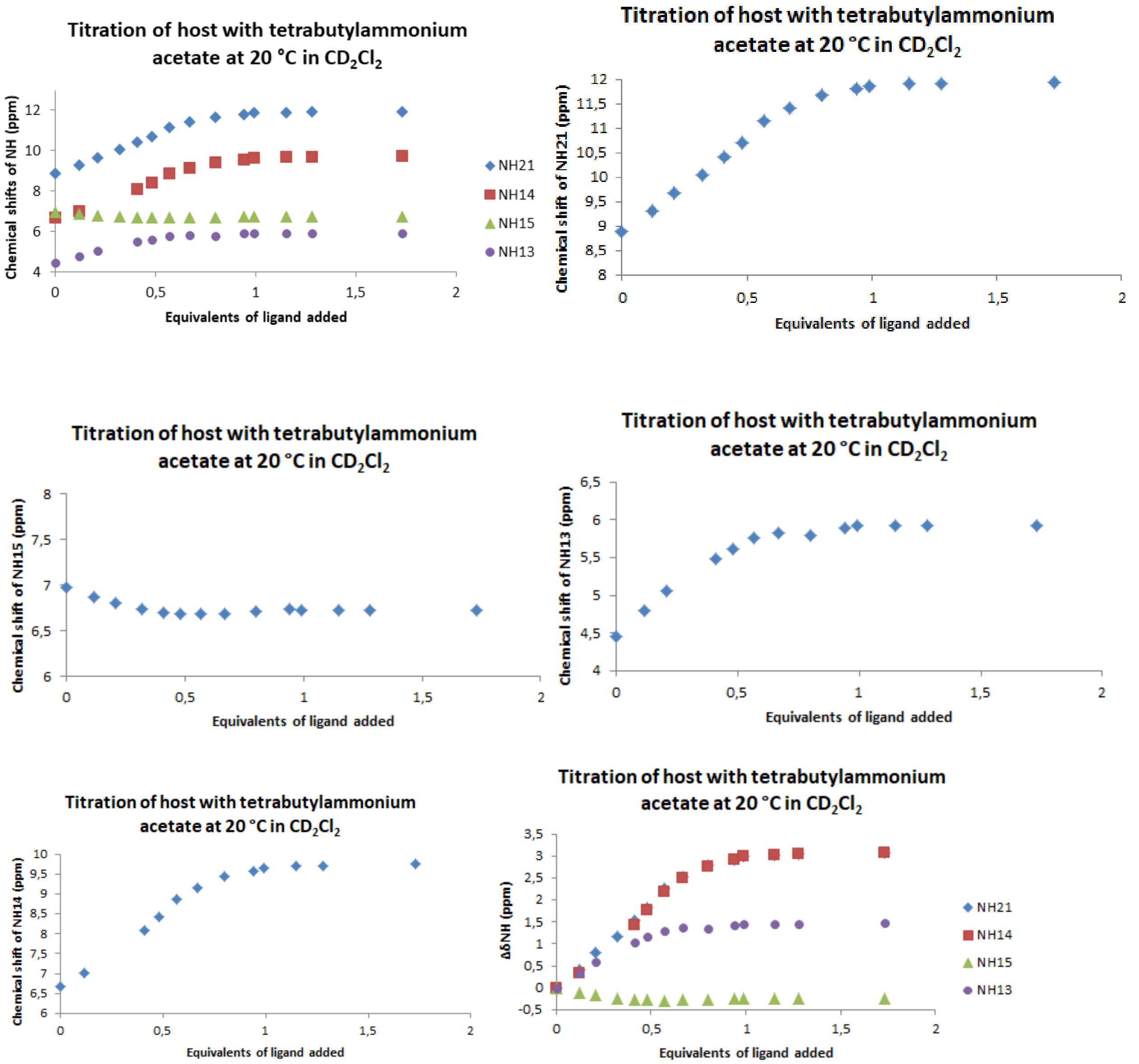

Figure S49: plots of variation of chemical shifts of NH signals, and plot of chemical induced shifts of NH signals of **2** (17.8 mM in CD_2Cl_2) upon addition of increments of tetrabutylammonium acetate, recorded by ^{1}H NMR at 25 °C (400 MHz).

Time to fit	0.9175 s
SSR	0.1912
Fitted datapoints	24
Fitted params	6

Parameters

Parameter (bounds)	Optimised	Error	Initial
K₁₁ (0 → ∞)	841.64 M⁻¹	± 25.7572 %	1000.00 M⁻¹
K₁₂ (0 → ∞)	37.36 M⁻¹	± 31.9559 %	100.00 M⁻¹

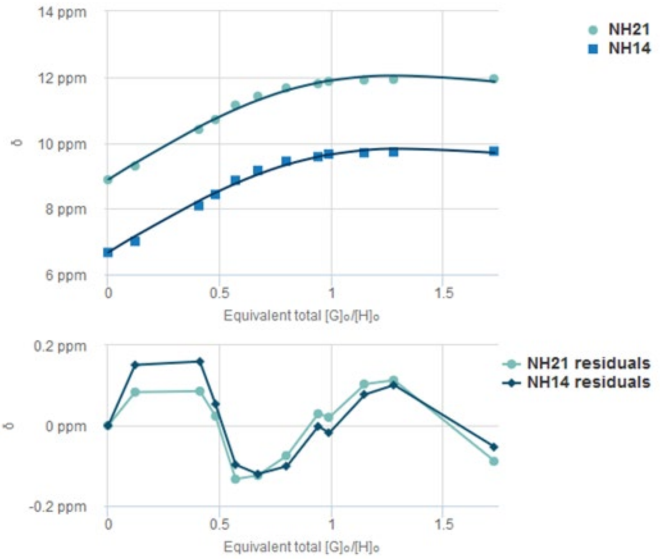

Figure S50: non-linear curve fitting analysis of the experimental titration data of **2** (17.8 mM in CD_2Cl_2) with increments of tetrabutylammonium acetate recorded at 25 °C (400 MHz) using a theoretical binding isotherm for 1:2 binding http://app.supramolecular.org/bindfit/view/79944229-d8c7-4b82-83cd-d2102bee6e0d

- Assignment of directionality change by NOE experiments

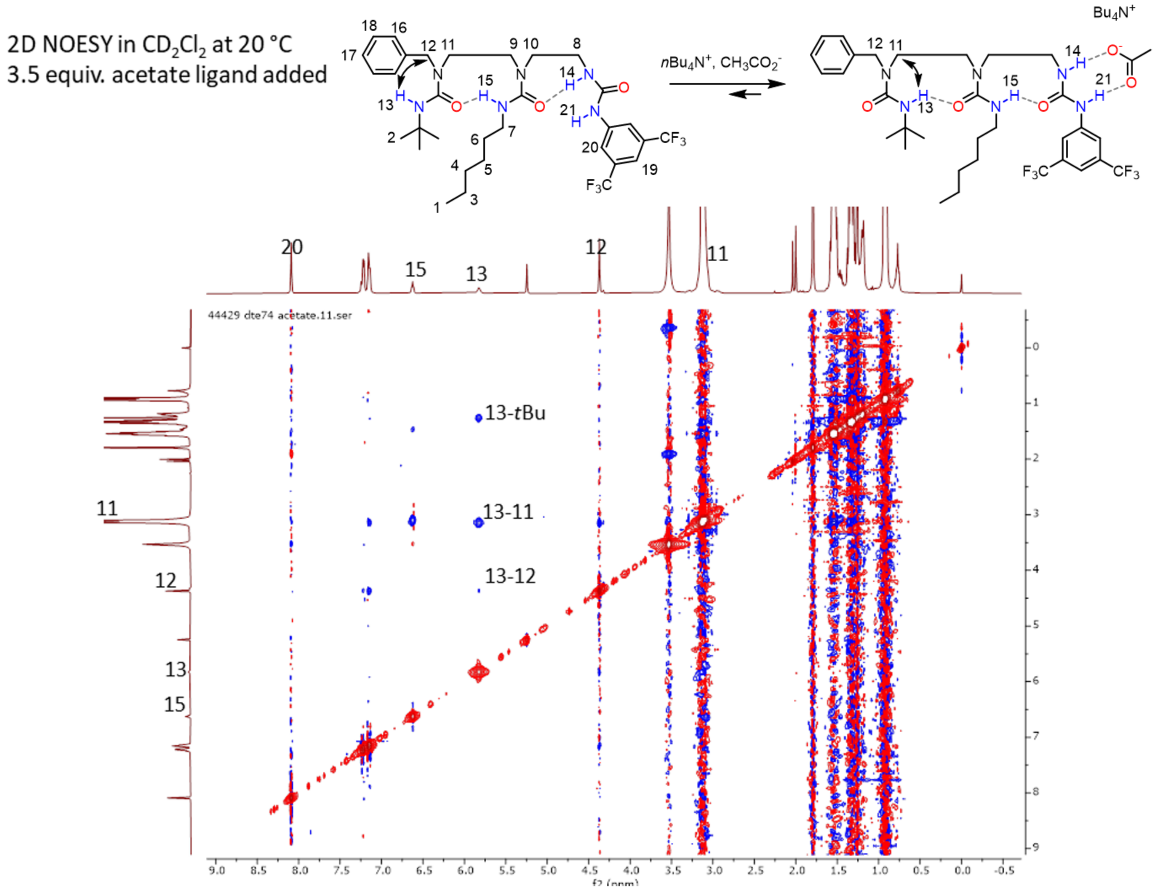

Figure S51: ^{1}H-^{1}H 2D NOESY spectrum of **2** (29.6 mM in CD_2Cl_2) in the presence of 3.5 equivalents of tetrabutylammonium acetate at 25 °C (500 MHz).

- Characterisation of complex formation by HRMS

Mass spectrometry analysis of 2 + tetrabutylammonium acetate (1.5 equiv.) using Synapt G2S Waters nanospray TOF MS ES+ experiment shows the formation of a 1:1 complex between the host and the ligand. **HRMS** (ES, positive ion mode) – *m/z* for $[C_{66}H_{119}F_6N_8O_7]^+$ 1217.9208, observed 1217.9213.

a

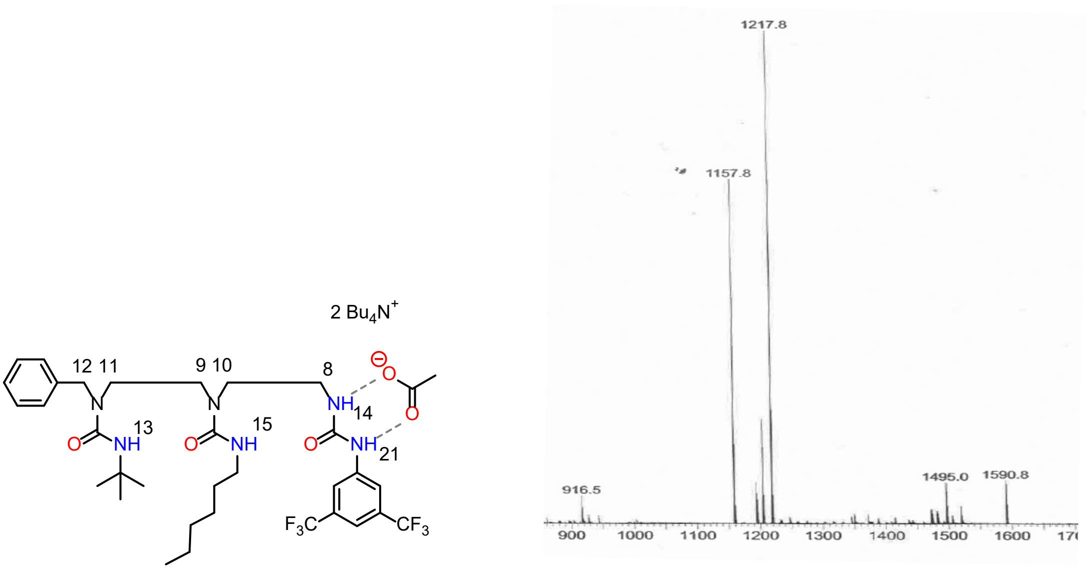

b

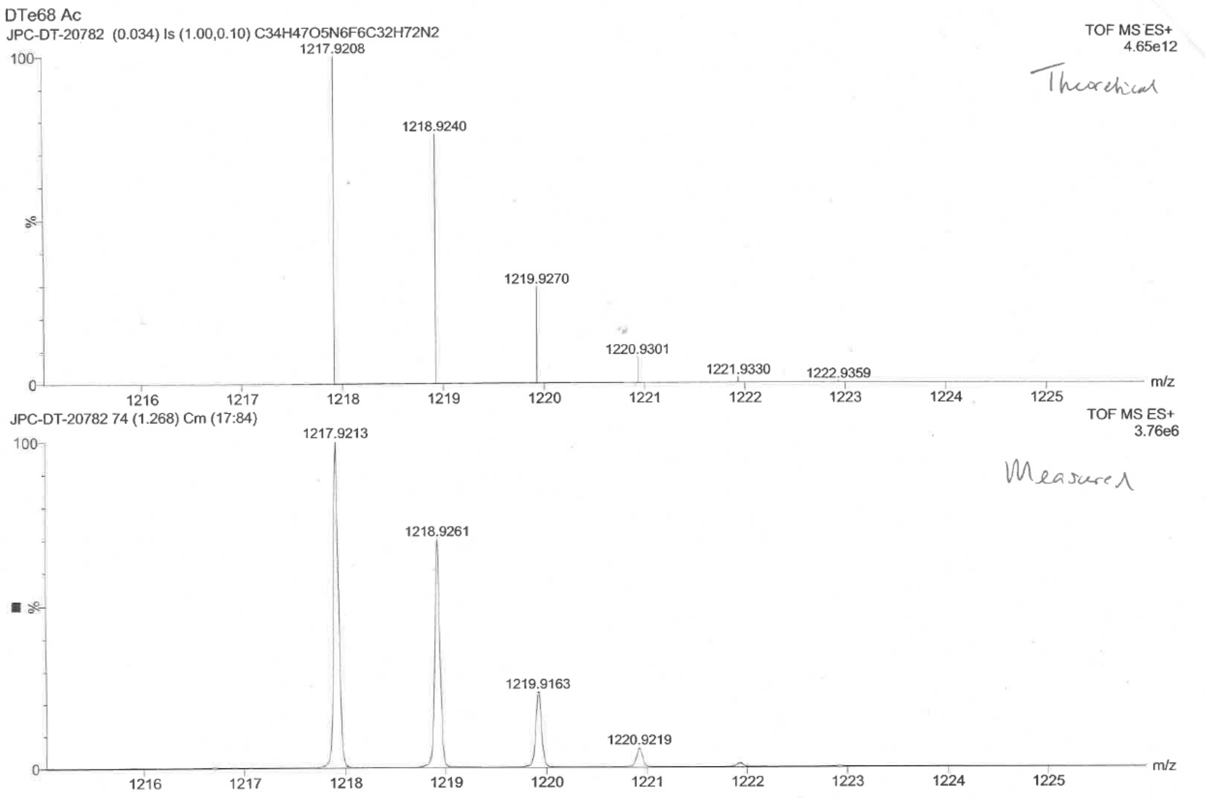

Figure S52: a ESI-MS (+ve) spectrum of **2** - $CH_3CO_2^-$, 2 Bu_4N^+, **b** experimental and simulated isotopic distribution of mass spectrum of 2 + $CH_3CO_2^-$, 2 Bu_4N^+

6.1.3. Titration with tetrabutylammonium chloride in CD_2Cl_2 at 25 °C

- Monitoring by ^{1}H NMR experiment

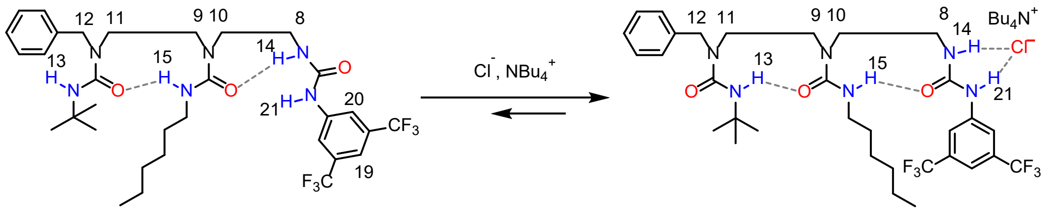

Titration experiment of **2** (11.9 mM) in CD_2Cl_2 at 25 °C with increasing amounts of tetrabutylammonium chloride (from 0 to 2.69 equivalents) was monitored by ^{1}H NMR (figure S53). The ligand was added as a solid so no variation of concentration occurs during the titration. The values of chemical shifts of the NH of 2 upon addition of tetrabutylammonium chloride are collected in Table S4 and plotted in a graph as {chemical shift of NH = f(ligand added)} (figure S55).

Progressive downfield shifts of both NH^{14} (CIS 0.94 ppm) and NH^{21} (CIS 1.67 ppm) signals are observed from 0 to 1 equivalent of ligand added, further addition of ligand has little effect on the chemical shifts.The observation is consistent with the formation of intermolecular hydrogen bonds between the ligand and the host at those positions.

The upfield chemical shift variations of NH^{15} (CIS 0.39 ppm) upon addition of ligand is comparatively smaller, NH^{15} is involved in intramolecular hydrogen bonds with adjacent ureas no matter the directionality of the hydrogen bond chain so no important variation of chemical shift value is expected upon change of global hydrogen bond directionality.

The signal for NH^{13} moves downfield (CIS 1.26 ppm), the chemical shifts vary from 0 to 1 equivalent of ligand added then stabilise even more ligand is added. It is consistent with a change of global directionality of the hydrogen bond chain controlled by the binding of chloride at the terminus of the chain.

Without ligand, 2D NOESY experiment on host **2** in CD_2Cl_2 at 25 °C shows a cross peak between NH^{13} and H^{12}, nOe experiment irradiating H8/9/10/11 displays a cross peak of weak intensity between H^{11} and NH^{13}, and a cross peak between H^{11} and H^{12} (figures S5 and S7). In contrast, after addition of 2.7 equivalents of tetrabutylammonium chloride to the same NMR sample, the 2D NOESY data show no cross peak is observed between NH^{13} and H^{12}, and a cross peak of high intensity between NH^{13} and H^{11} (figure s57). Those results are consistent with a reversal in the global directionality of the hydrogen bond chain.

An association constant for the binding event of chloride ligand to NH^{14} and NH^{21} was estimated by non-linear curve fitting analysis of the experimental titration curves and comparing the results with theoretical binding isotherms for 1:1, 1:2, 2:1 binding modes using supramolecular.org (figure S56. The best fit is obtained for a 1:2 binding mode with $K_{1.1}$ = 1256 +/-40% and $K_{1:2}$ = 121 $^{+/-}$ 50%. Titration at lower concentrations to determine more accurately a binding constant value was not possible as NH signals become difficult to see at lower concentrations.

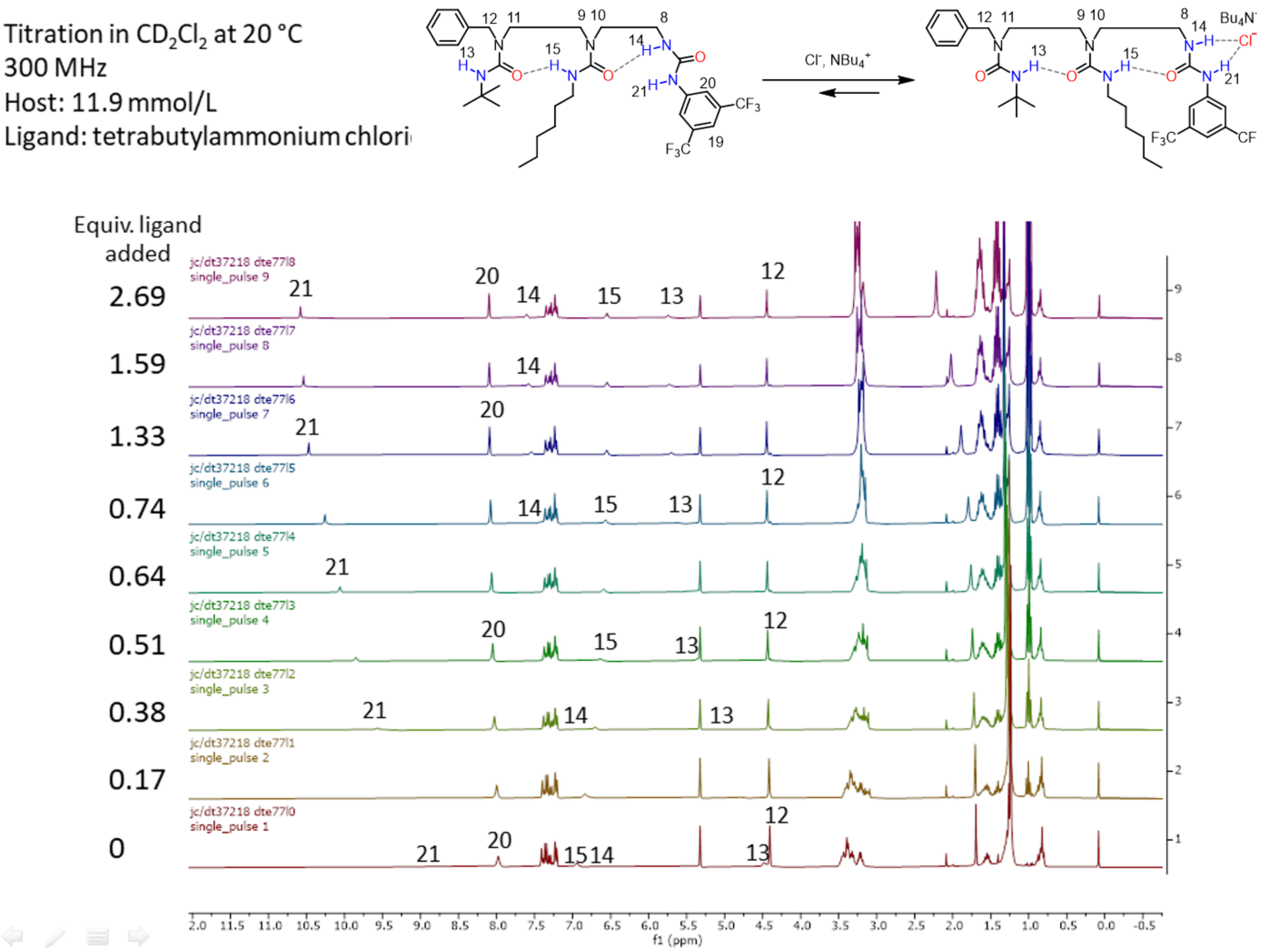

Figure S53: overlay of ^{1}H NMR spectra of **2** (11.9 mM in CD_2Cl_2) in the presence of increasing amounts of tetrabutylammonium chloride from 0 to 2.69 equivalents at 25 °C (300 MHz)

Concentration of host (mol/L)	Concentration of chloride ligand (mol/L)	Chemical shift NH21 (ppm)	Chemical shift NH14 (ppm)	Chemical shift NH15 (ppm)	Chemical shift NH13 (ppm)
0.01187	0	8.91	6.66	6.94	4.48
0.01187	0.002018	9.16	6.79	6.83	4.76
0.01187	0.00451	9.56	7.02	6.7	5.14
0.01187	0.00605	9.85	7.2	6.63	5.35
0.01187	0.0076	10.06	7.3	6.59	5.51
0.01187	0.0088	10.26	7.42	6.57	5.61
0.01187	0.0158	10.47	7.54	6.55	5.7
0.01187	0.0189	10.54	7.58	6.55	5.72
0.01187	0.032	10.58	7.6	6.55	5.74
**CIS (ppm)**		**1.66**	**0.94**	**-0.39**	**1.26**

Table S4. chemical shifts of ^{1}H NH signals recorded during the NMR titration of **2** (11.9 mM in CD_2Cl_2) with increments of tetrabutylammonium chloride from 0 to 2.69 equivalents at 25 °C (300 MHz).

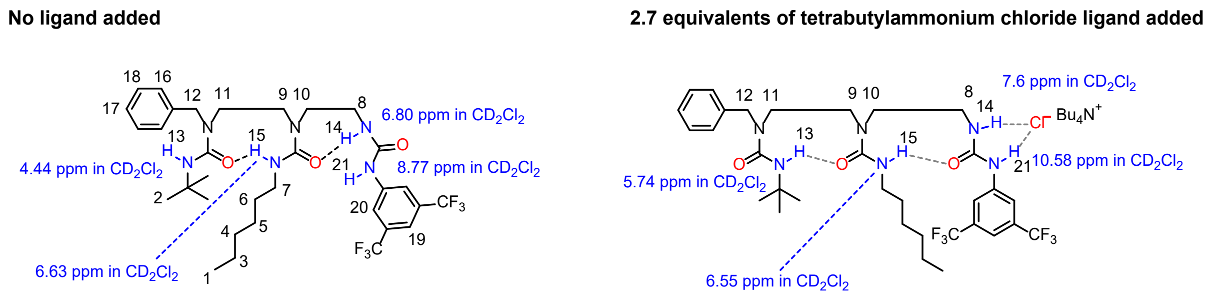

Figure S54: ^{1}H NMR chemical shifts of **2** NH signals (17.8 mM in CD_2Cl_2) before and after addition of 2.7 equivalents of tetrabutylammonium chloride at 25 °C.

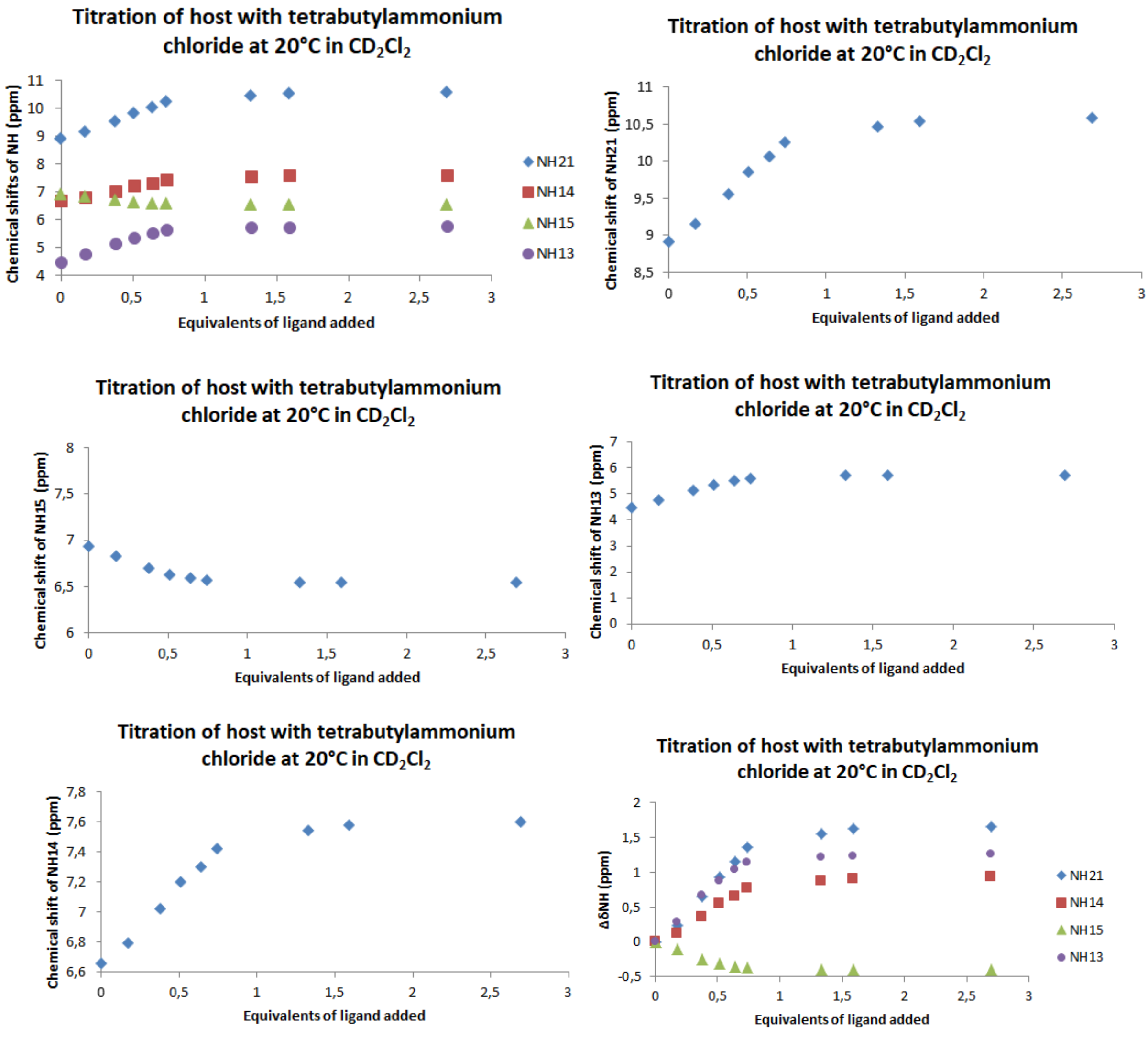

Figure S55: plots of variation of chemical shifts of NH signals of **2** (11.9 mM in CD_2Cl_2), and plot of variation of chemical induced shifts of NH signals upon addition of increments of tetrabutylammonium chloride from 0 to 2.69 equivalents, recorded by ^{1}H NMR at 25 °C (300 MHz).

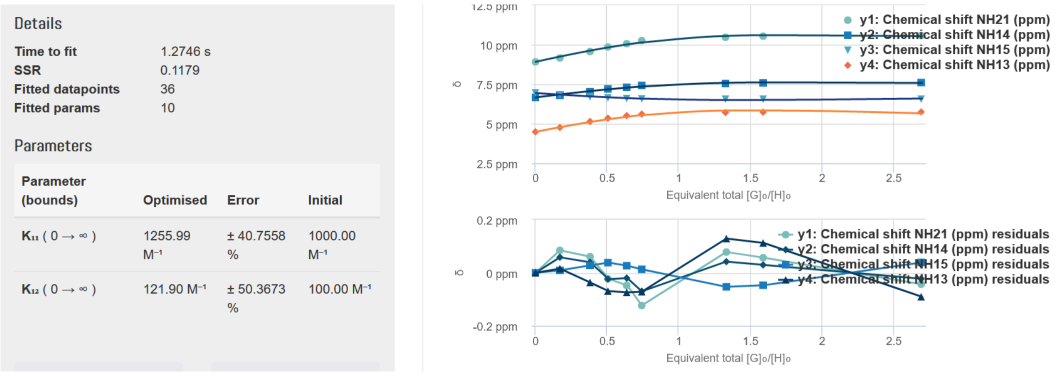

Figure S56: non-linear curve fitting analysis of the experimental titration data of **2** (11.9 mM in CD_2Cl_2) with increments of tetrabutylammonium chloride recorded at 25 °C (400 MHz) using a theoretical binding isotherm for 1:2 binding http://app.supramolecular.org/bindfit/view/92a35920-8c98-4d4b-878b-e79ce7193360

- Assignment of directionality change by NOESY

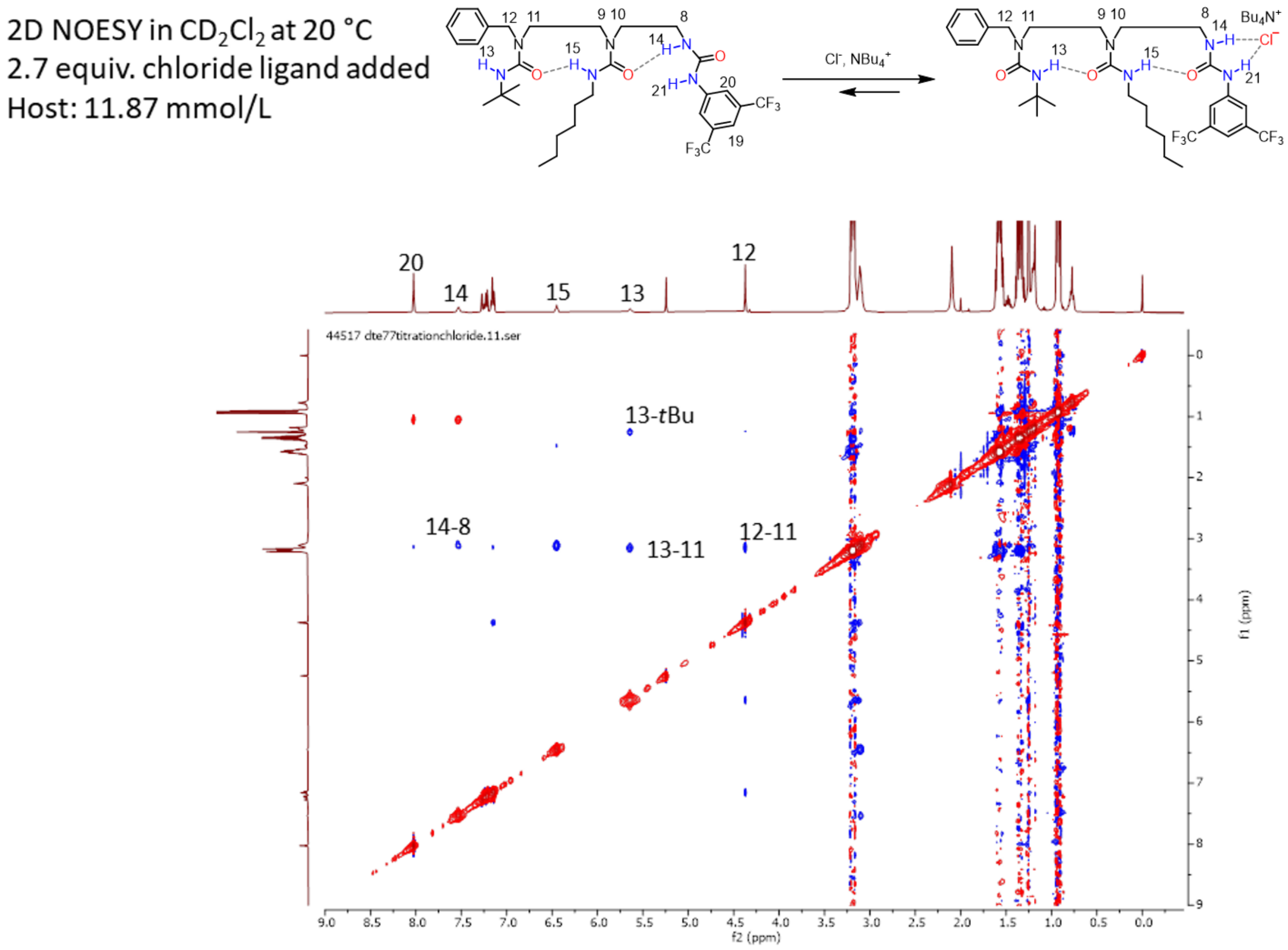

Figure S57: ^1H-^1H 2D NOESY NMR spectrum of **2** (11.9 mM in CD_2Cl_2) in the presence of 2.7 equivalents of tetrabutylammonium chloride at 25 °C (500 MHz).

- Characterisation of complex formation by HRMS

Mass spectrometry analysis of a sample of host + tetrabutylammonium chloride ligand (1.5 equiv.) using Synapt G2S Waters nanospray TOF MS ES+ experiment shows the formation of a 1:1 complex between the host and the ligand. **HRMS** (ES, positive ion mode) – *m/z* for $[C_{64}H_{116}F_6ClN_8O_3]^+$ 1193.8763, observed 1193.8768 (figure S58).

a

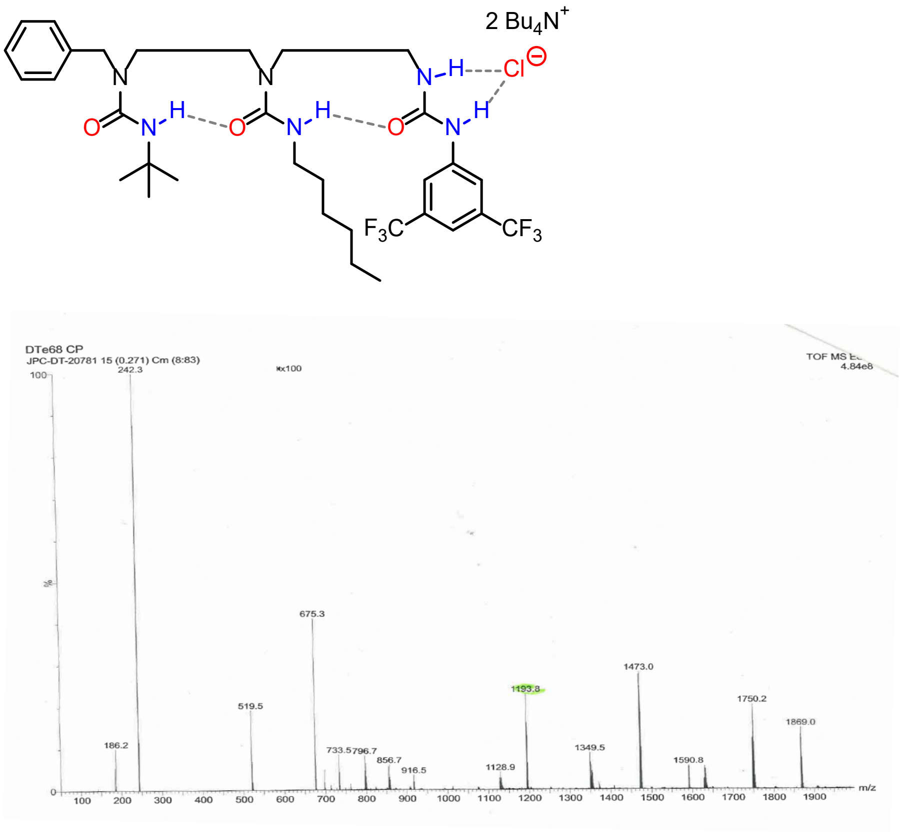

b

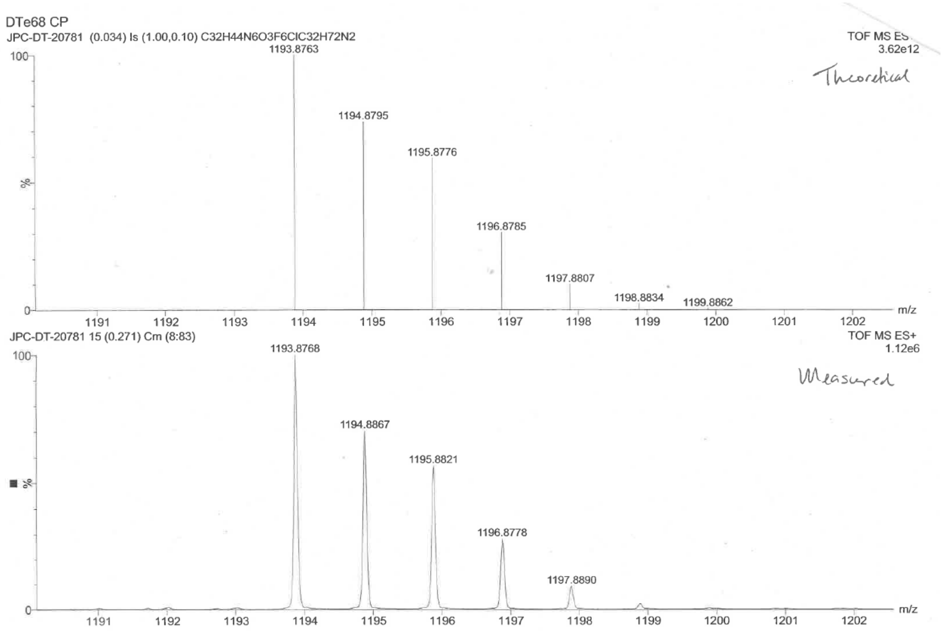

Figure S58: **a** ESI-MS (+ve) spectrum of **2** + Cl^-, 2 Bu_4N^+, **b** experimental and simulated isotopic distribution of mass spectrum of **2** + Cl^-, 2 Bu_4N^+

6.2. Compound 3

6.2.1. Titration with tetrabutylammonium diphenylphosphate in CD_2Cl_2 at 25 °C

- Monitoring by ^{1}H NMR experiment

Titration experiment of **3** (22.8 mM) in CD_2Cl_2 at 25 °C with increasing amounts of tetrabutylammonium diphenylphosphate (from 0 to 4.9 equivalents) was monitored by ^{1}H NMR (figure S59). The ligand was added as a solid so no variation of concentration occurs during the titration. The values of chemical shifts of the NH of **3** upon addition of tetrabutylammonium diphenylphosphate were collected in Table S5 and plotted in a graph as {chemical shift of NH = f(ligand added)} (figure S62).

Progressive downfield shifts of both NH^{19} (CIS 1.59 ppm) and NH^{12} (CIS 1.26 ppm) signals are observed, the variation of chemical shifts occur essentially from 0 to 1 equivalent of ligand added, further addition of ligand has little effect on the chemical shifts. This is consistent with the formation of intermolecular hydrogen bonds between the ligand and the host at those positions.

The chemical shift variations of NH^{13} (CIS 0.25 ppm downfield) upon addition of ligand is smaller, NH^{13} is involved in intramolecular hydrogen bonds with adjacent ureas no matter the directionality of the hydrogen bond chain so no important variation of chemical shift value is expected for NH^{13} upon change of global hydrogen bond directionality.

The signal for NH^{11} moves downfield (CIS 1.43 ppm), the chemical shifts vary from 0 to 1 equivalent of ligand added then stabilise even more ligand is added. It is attributed to a change of global directionality of the hydrogen bond chain controlled by the binding of phosphate at the terminus of the chain.

An association constant for the binding event of phosphate ligand to NH^{19} and NH^{12} was calculated by non-linear curve fitting analysis of the experimental titration curves and comparing the results with theoretical binding isotherms for 1:1, 1:2, 2:1 binding modes using supramolecular.org (figure S63), the best fit was obtained with a 1:1 binding model (K = 1008+/- 32%). Titration at lower concentrations to determine more accurately a binding constant value was not possible as NH signals become difficult to see at lower concentrations.

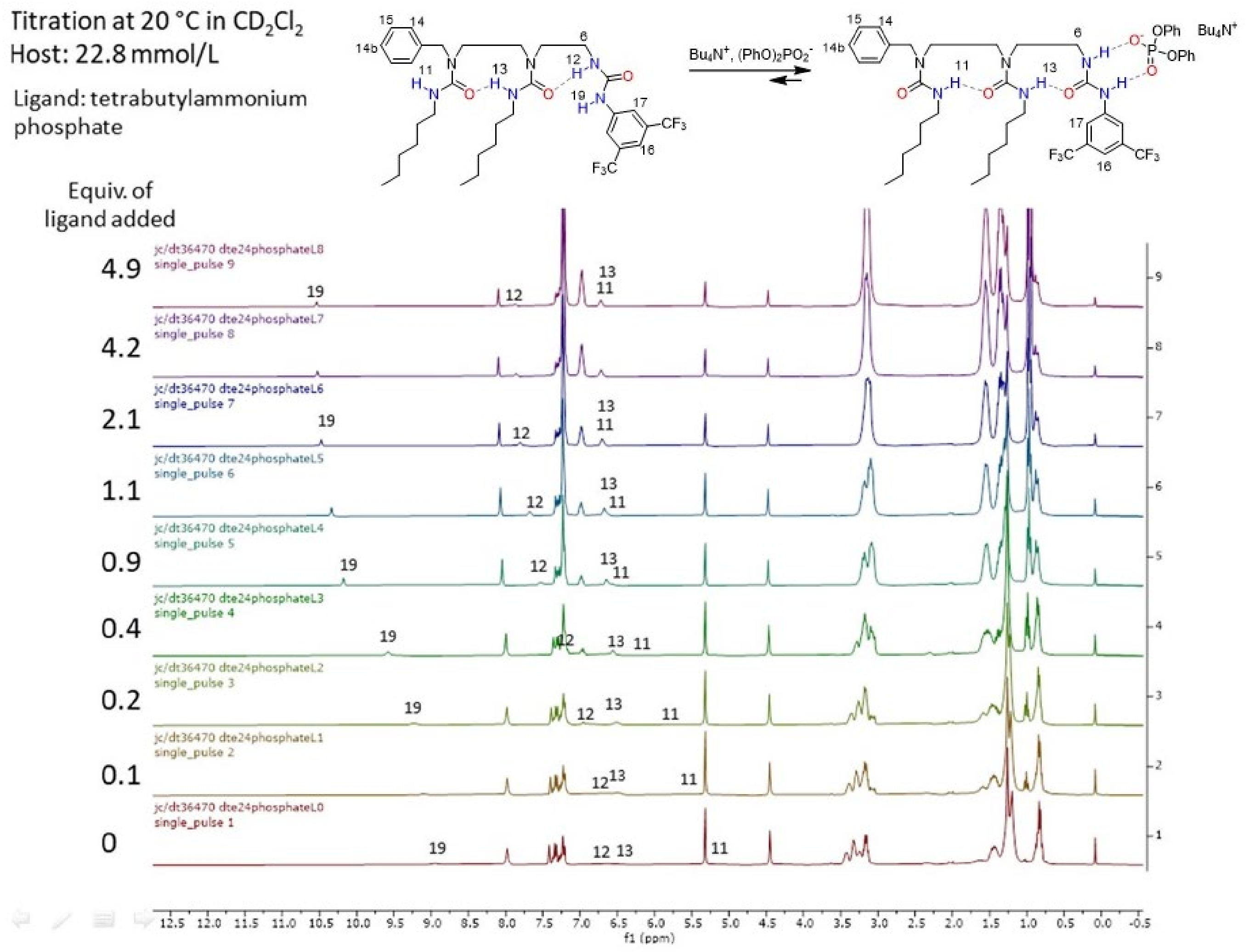

Figure S59: overlay of ^{1}H NMR spectra of **3** (22.8 mM in CD_2Cl_2) in the presence of increasing amounts of tetrabutylammonium diphenylphosphate from 0 to 4.9 equivalents at 25 °C (300 MHz)

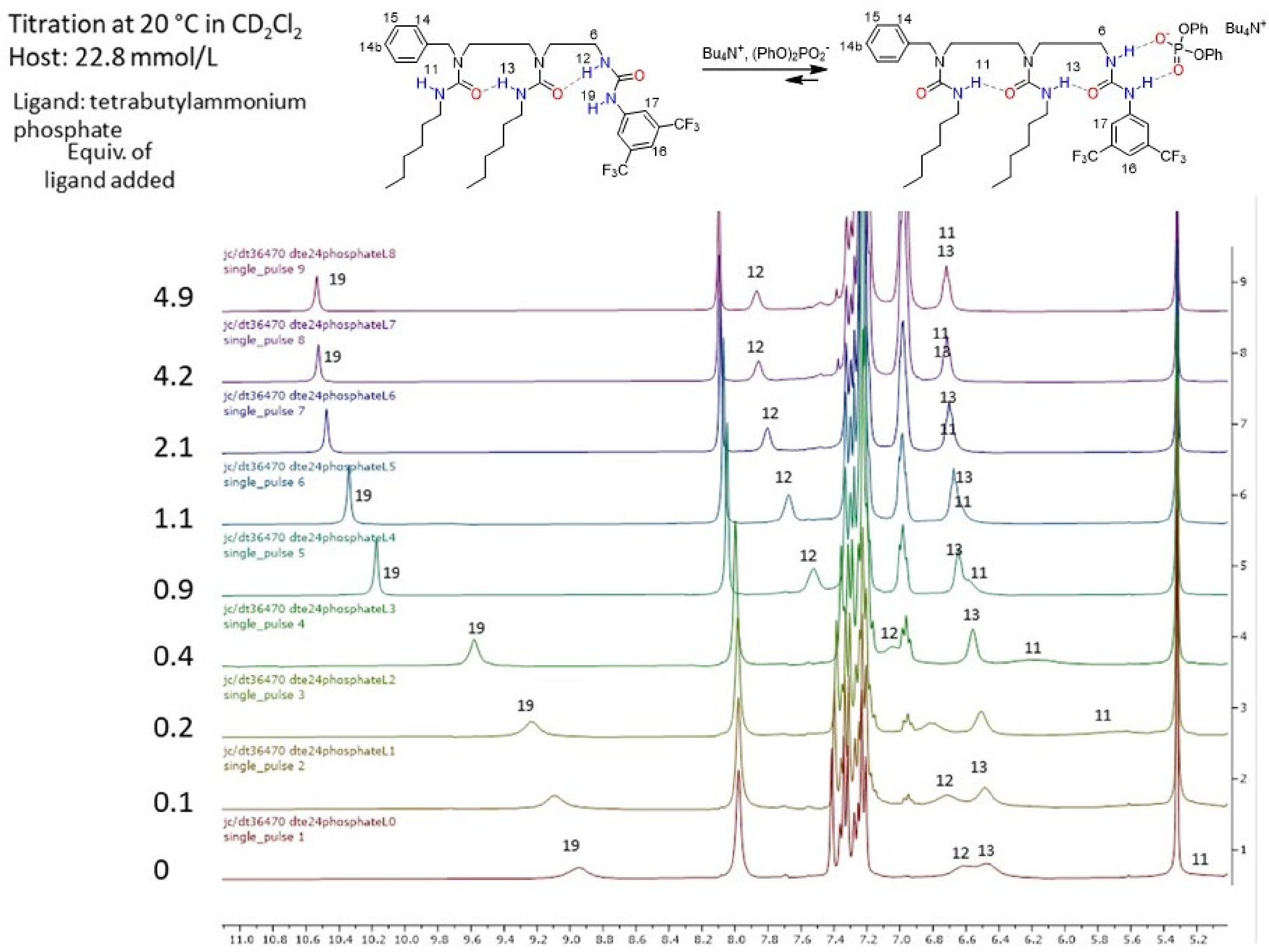

Figure S60: overlay of ¹H NMR spectra of **3** (22.8 mM in CD_2Cl_2) in the presence of increasing amounts of tetrabutylammonium diphenylphosphate from 0 to 4.9 equivalents at 25 °C (300 MHz) – enlargement of NH and aromatic region

Concentration of host (mol/L)	Equivalents of phosphate ligand	Chemical shift of NH12 (ppm)	Chemical shift of NH19 (ppm)	Chemical shift of NH13 (ppm)	Chemical shift of NH11 (ppm)	Ligand bonded (%)
0.0228	0	6.61	8.95	6.47	5.29	0
0.0228	0.15	6.72	9.09	6.49	5.52	0.08
0.0228	0.205	6.81	9.23	6.51	5.71	0.176
0.0228	0.41	7.06	9.58	6.56	6.15	0.396
0.0228	0.86	7.53	10.17	6.65	6.59	0.76
0.0228	1.15	7.68	10.34	6.68	6.64	0.874
0.0228	2.11	7.8	10.48	6.71	6.69	0.96
0.0228	4.18	7.86	10.53	6.72	6.72	0.96
0.0228	4.96	7.87	10.54	6.72	6.72	1
**CIS (ppm)**		**1.26**	**1.59**	**0.25**	**1.43**	

Table S5. chemical shifts of ¹H NH signals recorded during the NMR titration of **3** (22.8 mM in CD_2Cl_2) with increments of tetrabutylammonium diphenylphosphate at 25 °C (300 MHz).

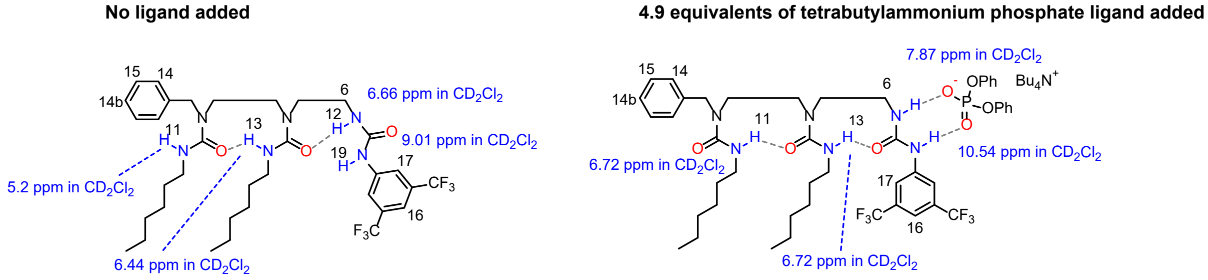

Figure S61: ^{1}H NMR chemical shifts of **3** NH signals (22.8 mM in CD_2Cl_2) before and after addition of 4.9 equivalents of tetrabutylammonium diphenylphospate at 25 °C.

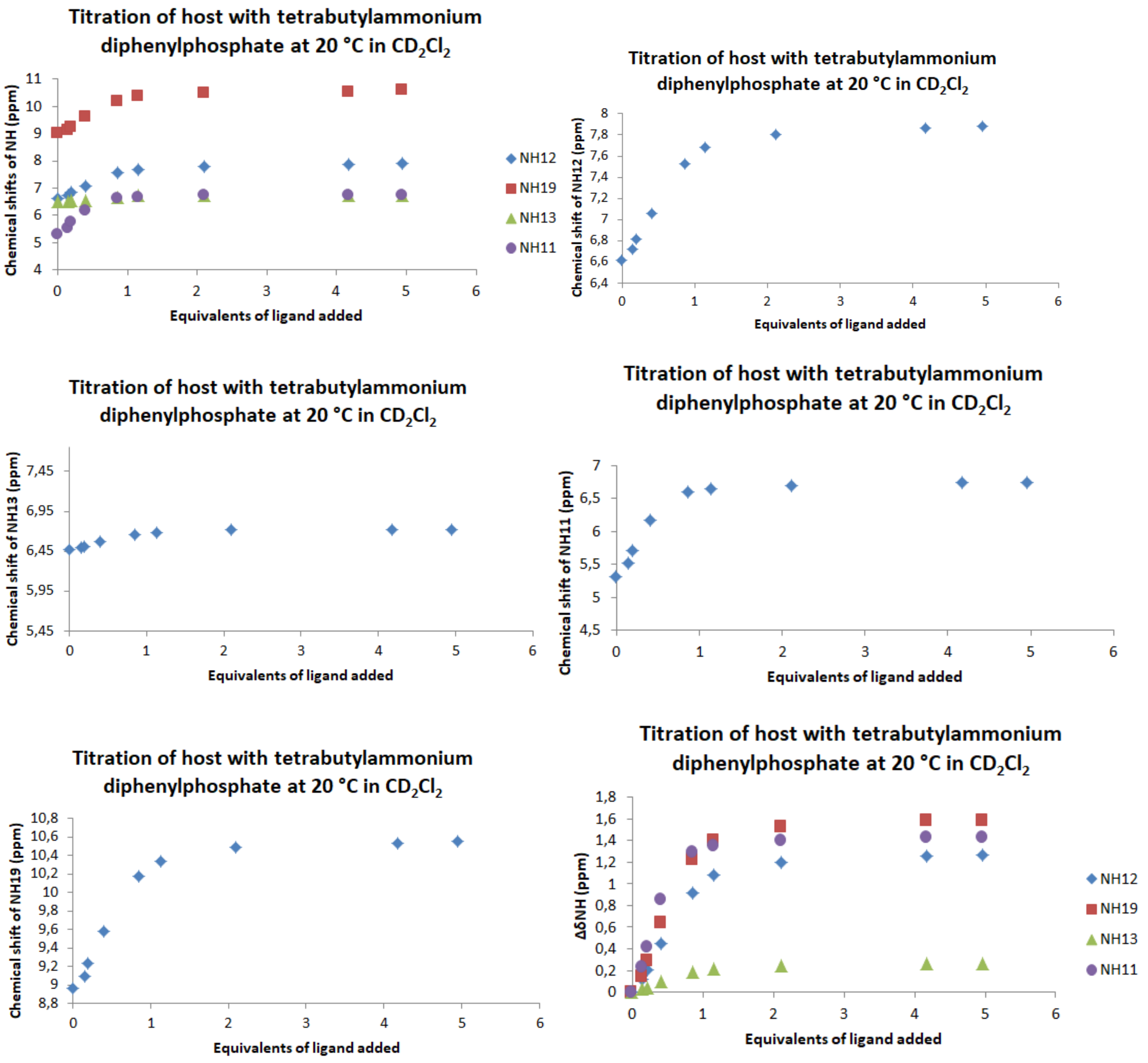

Figure S62: plots of variation of chemical shifts of NH signals of **3** (22.8 mM in CD_2Cl_2) and plot of variation of chemical induced shifts of NH signals upon addition of increments of tetrabutylammonium diphenylphosphate recorded by ^{1}H NMR at 25 °C (300 MHz).

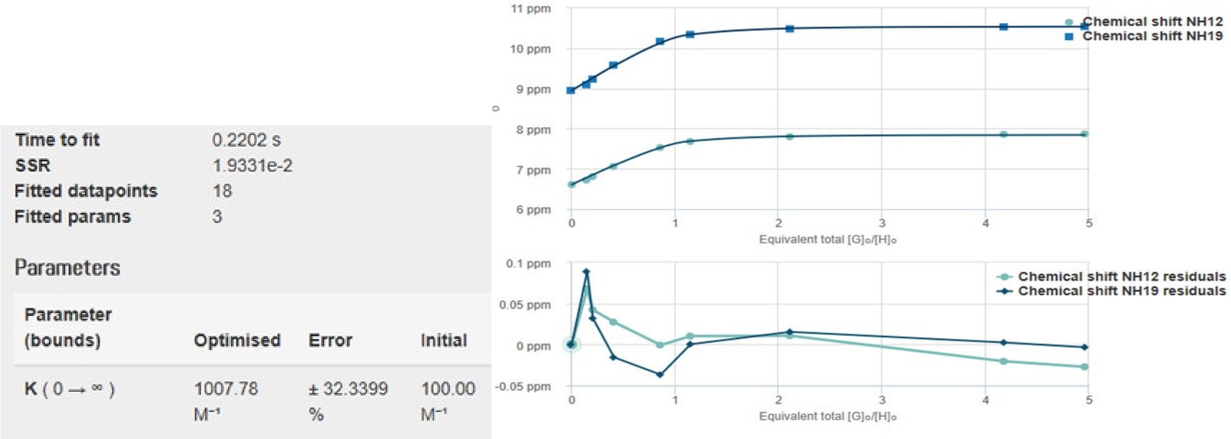

Figure S63: non-linear curve fitting analysis of the experimental titration data of **3** (22.8 mM in CD_2Cl_2) with increments of tetrabutylammonium diphenylphosphate recorded at 25 °C (400 MHz) using a theoretical binding isotherm for 1:1 binding http://app.supramolecular.org/bindfit/view/9d25c091-3be9-44d1-a3e5-2a9ee063a672

6.2.2. Titration with tetrabutylammonium acetate in CD_2Cl_2 at 25 °C

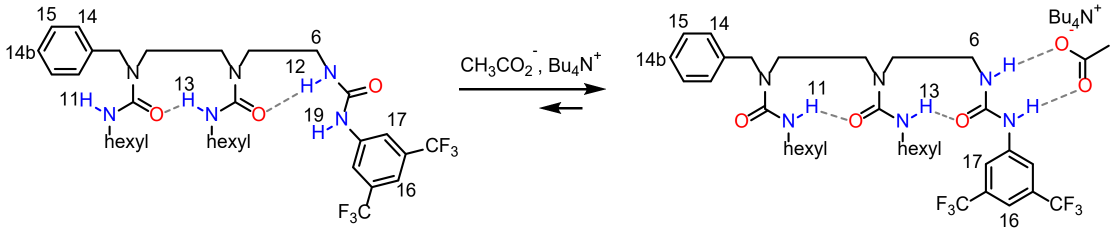

Titration experiment of **3** (15.4 mM) in CD_2Cl_2 at 25 °C with increasing amounts of tetrabutylammonium acetate (from 0 to 2.36 equivalents) was monitored by ^{1}H NMR (figure S64). The ligand was added as a solid so no variation of concentration occurs during the titration. The values of chemical shifts of the NH of **3** upon addition of tetrabutylammonium acetate were collected in Table S6 and plotted in a graph as {chemical shift of NH = f(ligand added)} (figure S66).

Progressive downfield shifts of both NH^{19} (CIS 2.66 ppm) and NH^{12} (CIS 2.81 ppm) signals are observed upon addition of ligand, the variation of chemical shifts occur essentially from 0 to 1 equivalent of ligand added, further addition of ligand has little effect on the chemical shifts. This is consistent with the formation of intermolecular hydrogen bonds between the acetate ligand and the host at positions NH^{19} and NH12.

The downfield chemical shift variations of NH^{13} (CIS 0.28 ppm) upon addition of ligand is comparatively smaller, NH^{13} is involved in intramolecular hydrogen bonds with adjacent ureas no matter the directionality of the hydrogen bond chain so no important variation of chemical shift value is expected for NH^{13} upon change of global hydrogen bond directionality.

The signal for NH^{11} moves downfield (CIS 1.38 ppm), its chemical shifts vary from 0 to 1 equivalent of ligand added then stabilise even more ligand is added. It is consistent with a change of dglobal irectionality of the hydrogen bond chain controlled by the binding of acetate at the terminus of the chain.

An association constant for the binding event of acetate ligand to NH^{19} and NH^{12} was estimated by non-linear curve fitting analysis of the experimental titration curves and comparing the results with theoretical binding isotherms for 1:1, 1:2, 2:1 binding modes using supramolecular.org (figure S67). The best fit is obtained for a 1:1 binding mode with K = 452 +/- 17%.[1] Titration at lower concentrations to determine more accurately a binding constant value was not possible as NH signals become difficult to see at lower concentrations.

[1] For NH^{19} alone using a 1:1 binding isotherm: K = 672 $^{+/-}$ 15% http://app.supramolecular.org/bindfit/view/a3cfe9e0-9834-4f3a-a05d-32a6541d8299; for NH^{12} alone using a 1:1 binding isotherm:K = 291 $^{+/-}$ 29% http://app.supramolecular.org/bindfit/view/c2c2a431-846e-482b-a14f-afa929835ade

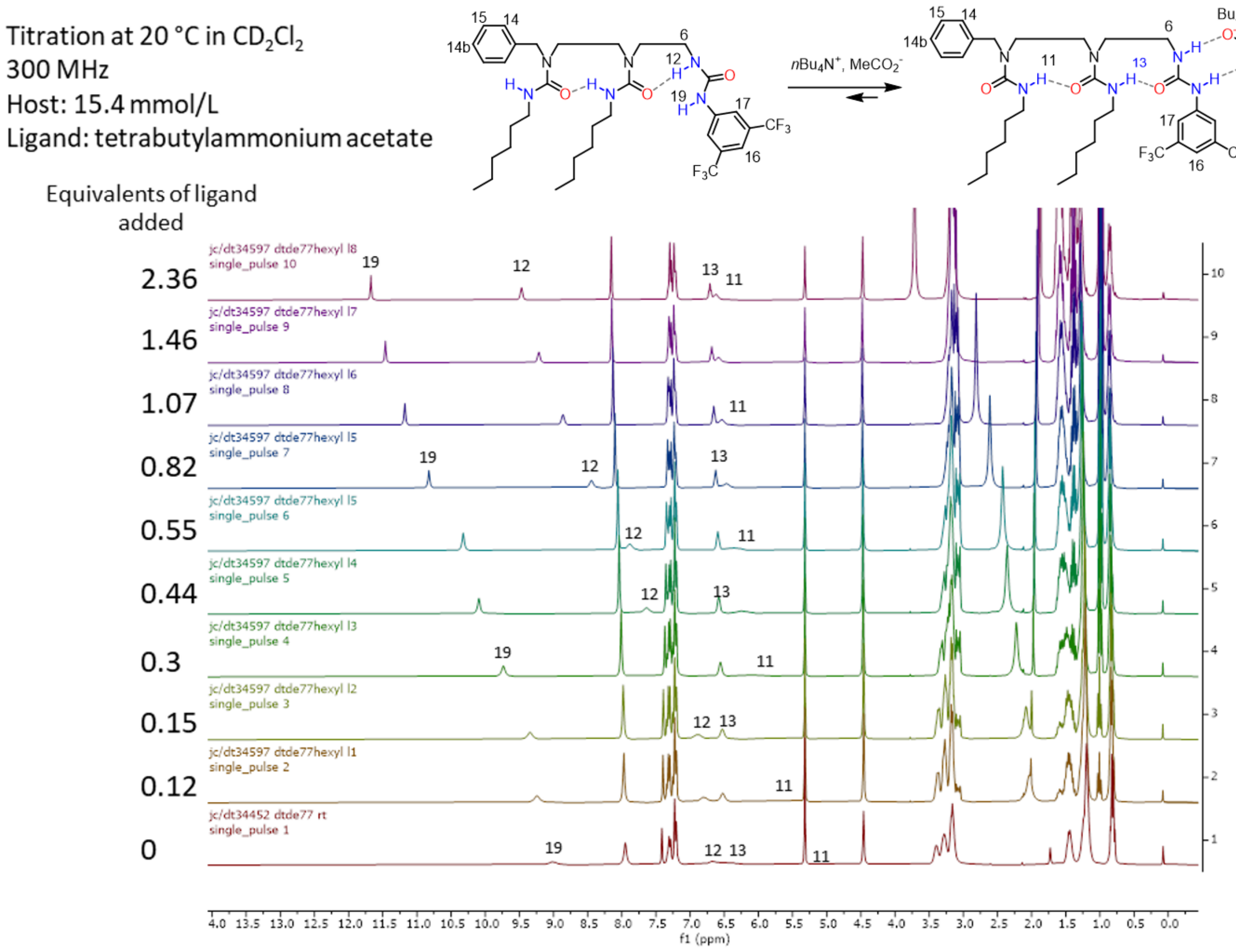

Figure S64: overlay of ^{1}H NMR spectra of **3** (15.4 mM in CD_2Cl_2) in the presence of increasing amounts of tetrabutylammonium acetate from 0 to 2.36 equivalents, recorded at 25 °C (300 MHz)

Concentration of host (mol/L)	Concentration of acetate ligand (mol/L)	Chemical shift NH^{19} (ppm)	Chemical shift NH^{12} (ppm)	Chemical shift NH^{13} (ppm)	Chemical shift NH^{11} (ppm)
0.0154	0	9.01	6.66	6.43	5.24
0.0154	0.001848	9.23	6.8	6.52	5.69
0.0154	0.00235	9.35	6.9	6.53	5.76
0.0154	0.00462	9.73	7.27	6.56	6.04
0.0154	0.006776	10.09	7.64	6.58	6.24
0.0154	0.00847	10.32	7.89	6.59	6.32
0.0154	0.01263	10.83	8.45	6.63	6.47
0.0154	0.0165	11.18	8.86	6.65	6.54
0.0154	0.0225	11.46	9.22	6.68	6.58
0.0154	0.0364	11.67	9.47	6.71	6.62
**CIS (ppm)**		**2.66**	**2.81**	**0.28**	**1.38**

Table S6. Chemical shifts of ^{1}H NH signals recorded during the NMR titration of **3** (15.4 mM in CD_2Cl_2) with increments of tetrabutylammonium acetate at 25 °C (300 MHz).

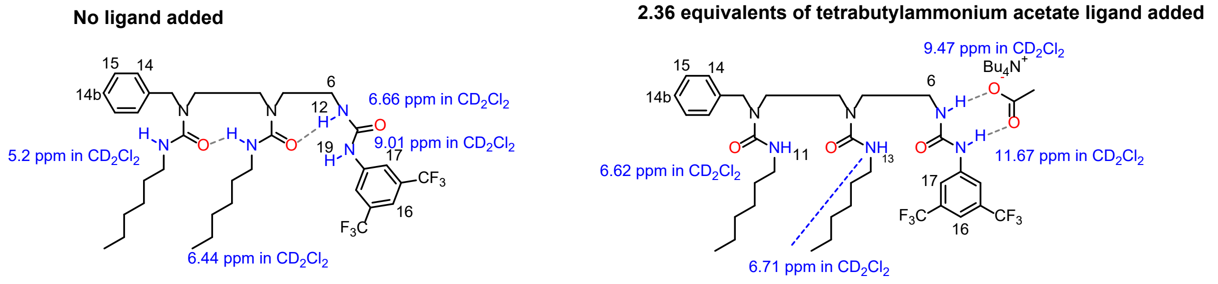

Figure S65: ^{1}H NMR chemical shifts of **3** NH signals (15.48 mM in CD_2Cl_2) before and after addition of 2.36 equivalents of tetrabutylammonium acetate at 25 °C.

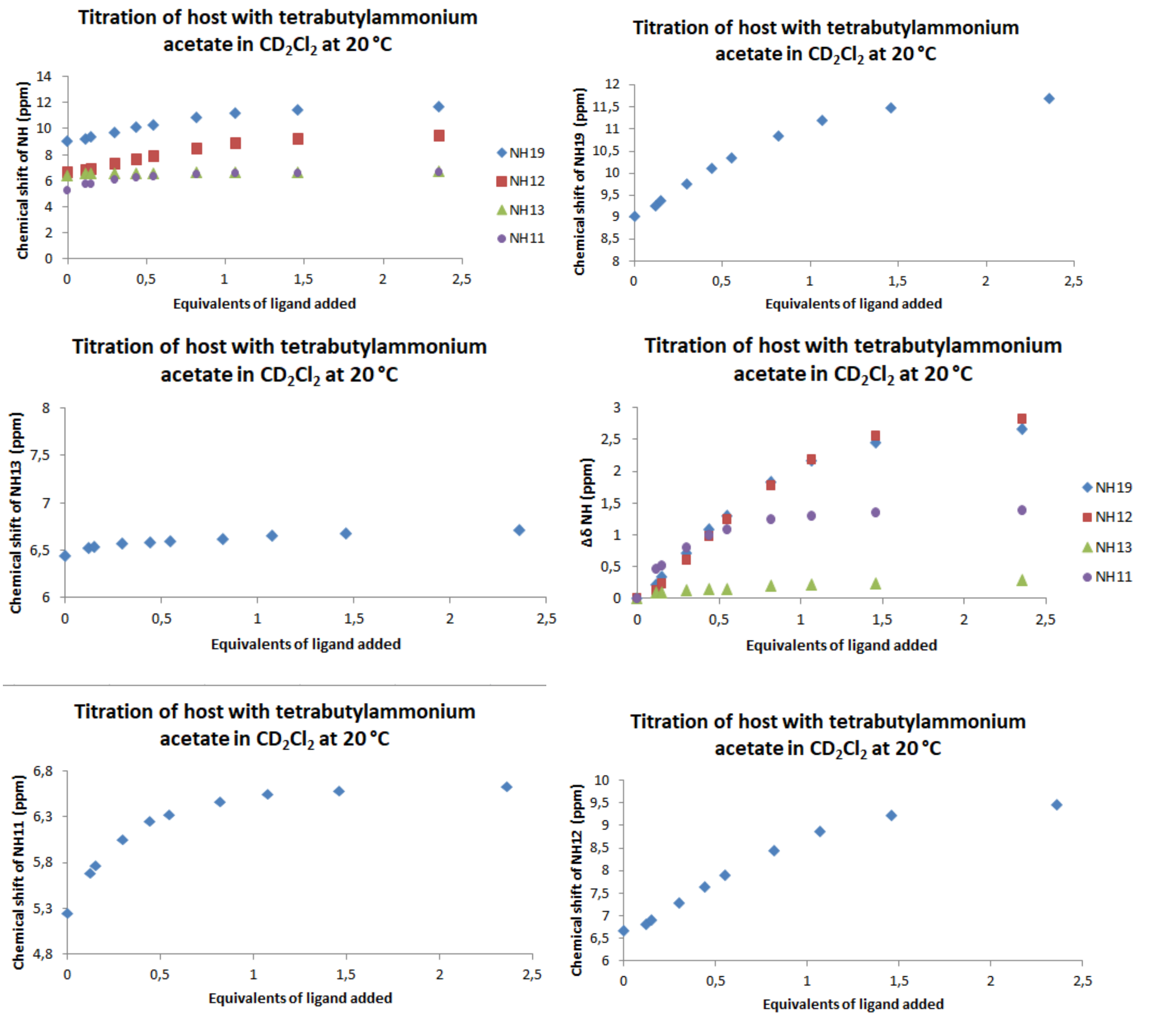

Figure S66: plots of variations of chemical shifts of NH signals of **3** (15.4 mM in CD_2Cl_2) and plot of variation of chemical induced shifts of NH signals upon addition of increments of tetrabutylammonium acetate, recorded by ^{1}H NMR at 25 °C (300 MHz).

Time to fit	0.2125 s
SSR	0.1278
Fitted datapoints	20
Fitted params	3

Parameters

Parameter (bounds)	Optimised	Error	Initial
K (0 → ∞)	452.70 M⁻¹	± 17.8678 %	100.00 M⁻¹

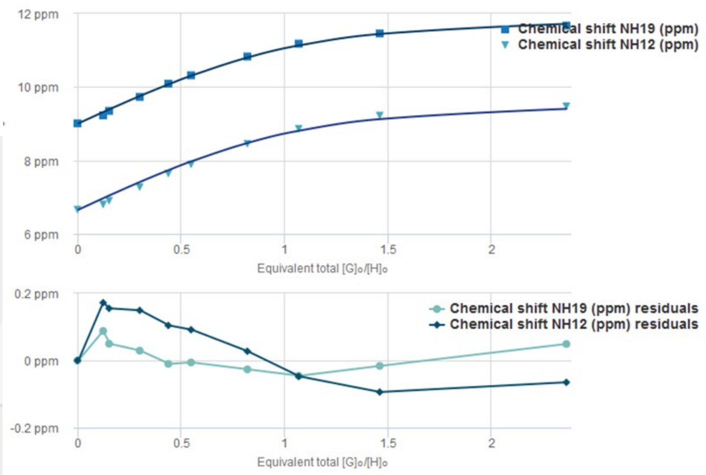

Figure S67: non-linear curve fitting analysis of the experimental titration data of **3** (15.48 mM in CD_2Cl_2) with increments of tetrabutylammonium acetate recorded at 25 °C (400 MHz) using a theoretical binding isotherm for 1:1 binding http://app.supramolecular.org/bindfit/view/98f65db1-c306-498f-9aa9-8d77b5ed48

6.3. Compound 9

6.3.1. Titration with tetrabutylammonium chloride in CD_2Cl_2 at 25 °C

^{1}H NMR spectrum of compound **9** (27.6 mM) in CD_2Cl_2 at 25 °C displays one signal at 5.36 ppm for the two ureido NH, the chemical shift value is the weighted average of the chemical shifts of ureido NH in hydrogen bonding and in non-hydrogen-bonding modes (rapid equilibrium between directionalities on the NMR timescale).

A titration of that solution with increasing amounts of tetrabutylammonium chloride (from 0 to 3.6 equivalents) was monitored by ^{1}H NMR spectroscopy (figure S68). The ligand was added as a solid so no variation of concentration occurs during the titration. Addition of tetrabutylammonium chloride causes only minimal variation of the chemical shift values of the ureido NH signals compared to the shifts observed during the titrations described above (complexation induced shift CIS for **9** at 1.4 equivalents of ligand added = 0.08 ppm, compared to downfield shifts in the order of 1.5 ppm observed for the terminal ureido NH during the titrations of the previous hosts with the same quantity of ligand).

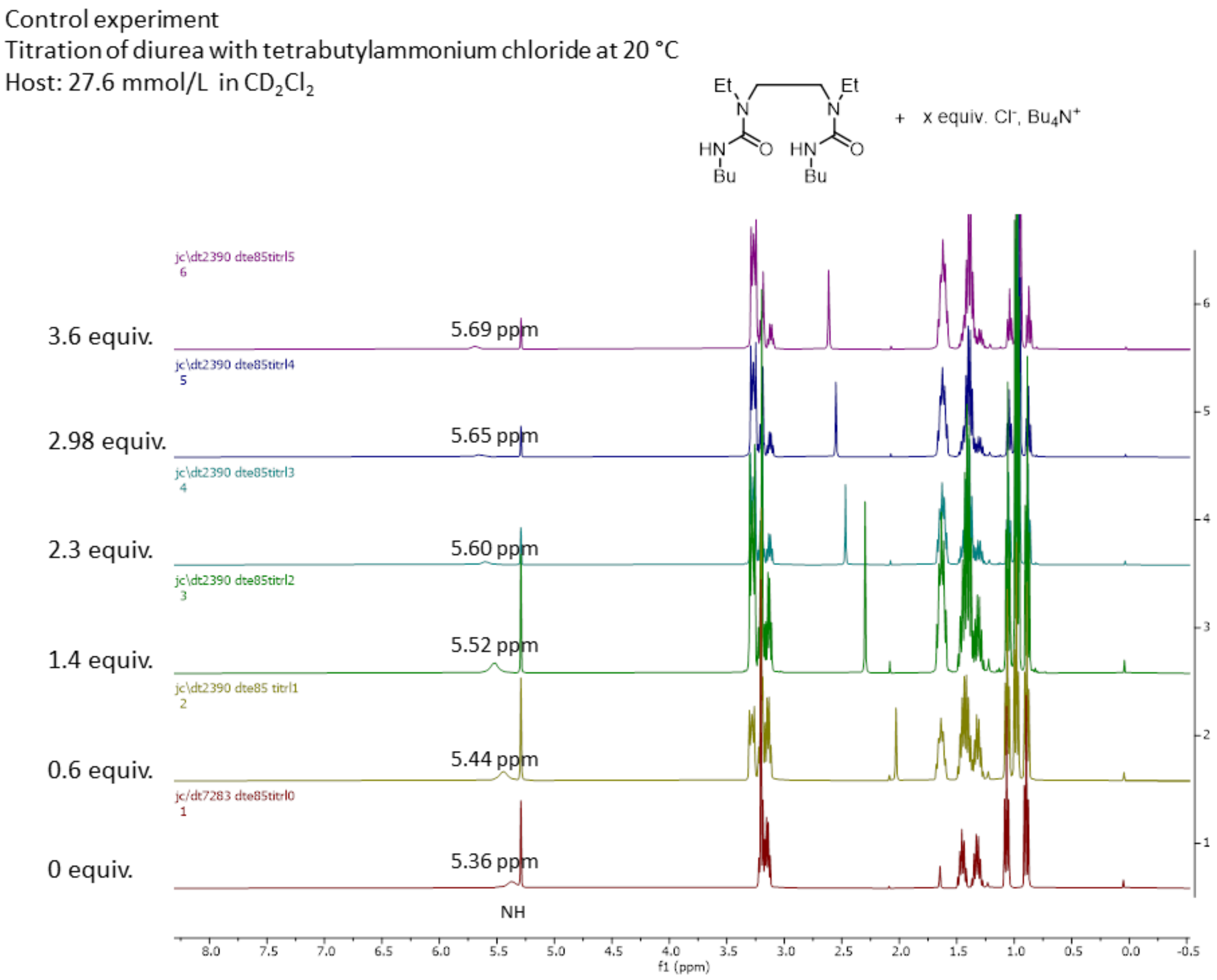

Figure S68: overlay of ^{1}H NMR spectra of **9** (27.6 mM in CD_2Cl_2) in the presence of increasing amounts of tetrabutylammonium chloride from 0 to 3.6 equivalents at 25 °C (300 MHz)

6.3.2. Titration with tetrabutylammonium acetate in CD_2Cl_2 at 25 °C

^1H NMR spectrum of compound **9** (24 mM) in CD_2Cl_2 at 25 °C displays one signal at 5.4 ppm for the two ureido NH. the chemical shift value is the weighted average of the chemical shifts of ureido NH in hydrogen bonding and in non-hydrogen-bonding modes (rapid equilibrium between directionalities on the NMR timescale). Titration of that solution with tetrabutylammonium acetate (from 0 to 4.2 equivalents) was monitored by ^1H NMR spectroscopy (figure S69). The ligand was added as a solid so no variation of concentration occurs during the titration. The addition of tetrabutylammonium acetate to the solution causes only minimal minimal variation of the chemical shift values of the NH signals compared to the shifts observed for the titrations described for the previous hosts (CIS at 1.2 equivalents of ligand added = 0.11 ppm for **9**, compared to downfield shifts in the order of 1.5 ppm for the terminal ureido NH during the titrations of triurea hosts with the same quantity of ligand).

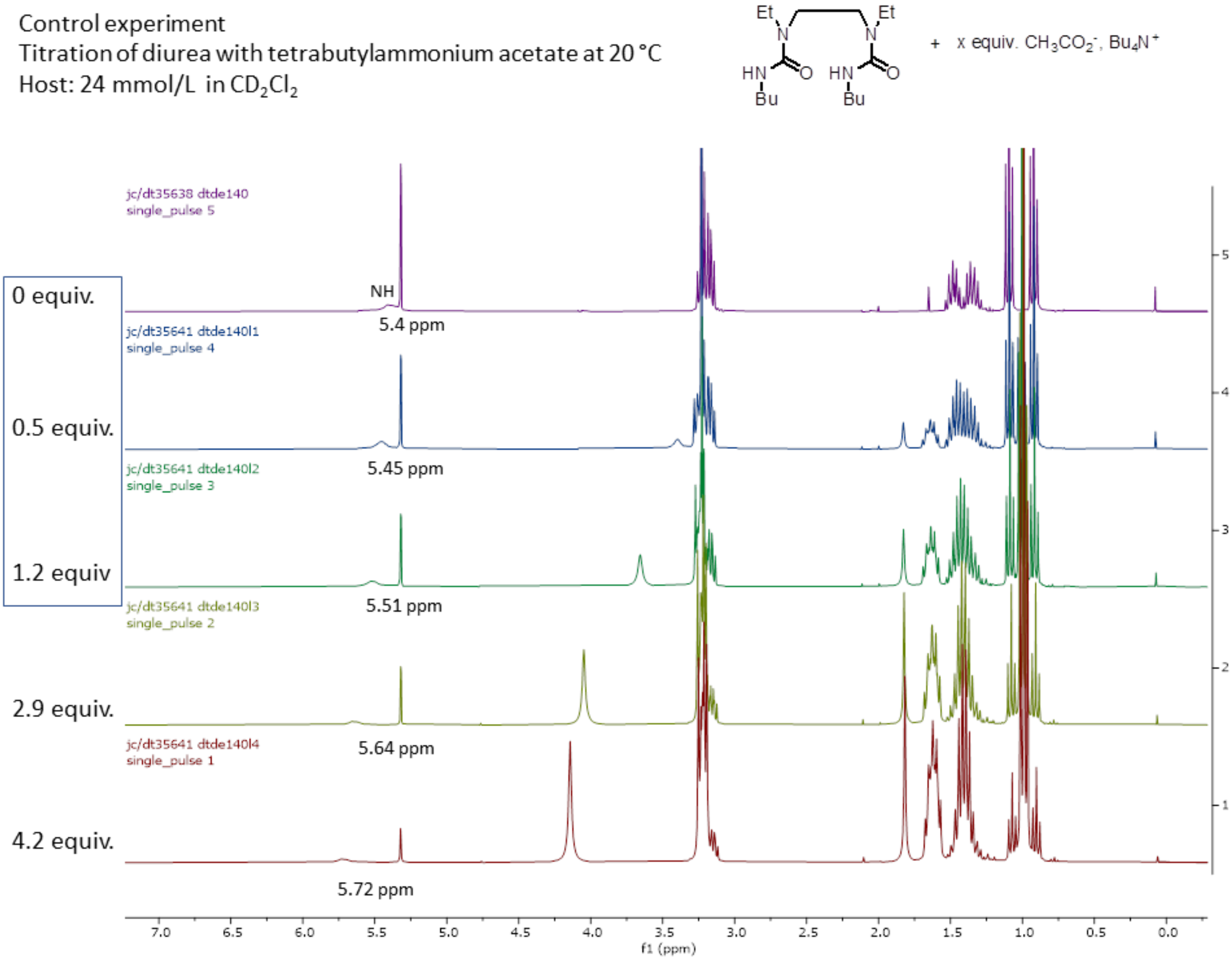

Figure S69: overlay of ^1H NMR spectra of **9** (24 mM in CD_2Cl_2) in the presence of increasing amounts of tetrabutylammonium acetate from 0 to 4.2 equivalents at 25 °C (300 MHz)

6.3.3. Titration with tetrabutylammonium diphenylphosphate in CD_2Cl_2 at 25 °C

^{1}H NMR spectrum of compound **9** (24.5 mM) in CD_2Cl_2 at 25 °C displays one signal at 5.41 ppm for the two ureas NH, the chemical shift value is the weighted average of the chemical shifts of ureido NH in hydrogen bonding and in non-hydrogen-bonding modes (rapid equilibrium between directionalities on the NMR timescale). Titration of that solution with tetrabutylammonium diphenylphosphate (from 0 to 3 equivalents) was monitored by ^{1}H NMR spectroscopy (figure S70). The ligand was added as a solid so no variation of concentration occurs during the titration. The addition of tetrabutylammonium diphenylphosphate to the solution causes only minimal chemical shift variation of the ureido NH signals compared to the shifts observed for the titrations described for the triurea hosts above (CIS at 1.1 equivalents of ligand added = 0.17 ppm for **9**, compared to downfield shifts in the order of 1.5 ppm for the terminal ureido NH during the titrations of tri urea hosts with the same quantity of ligand).

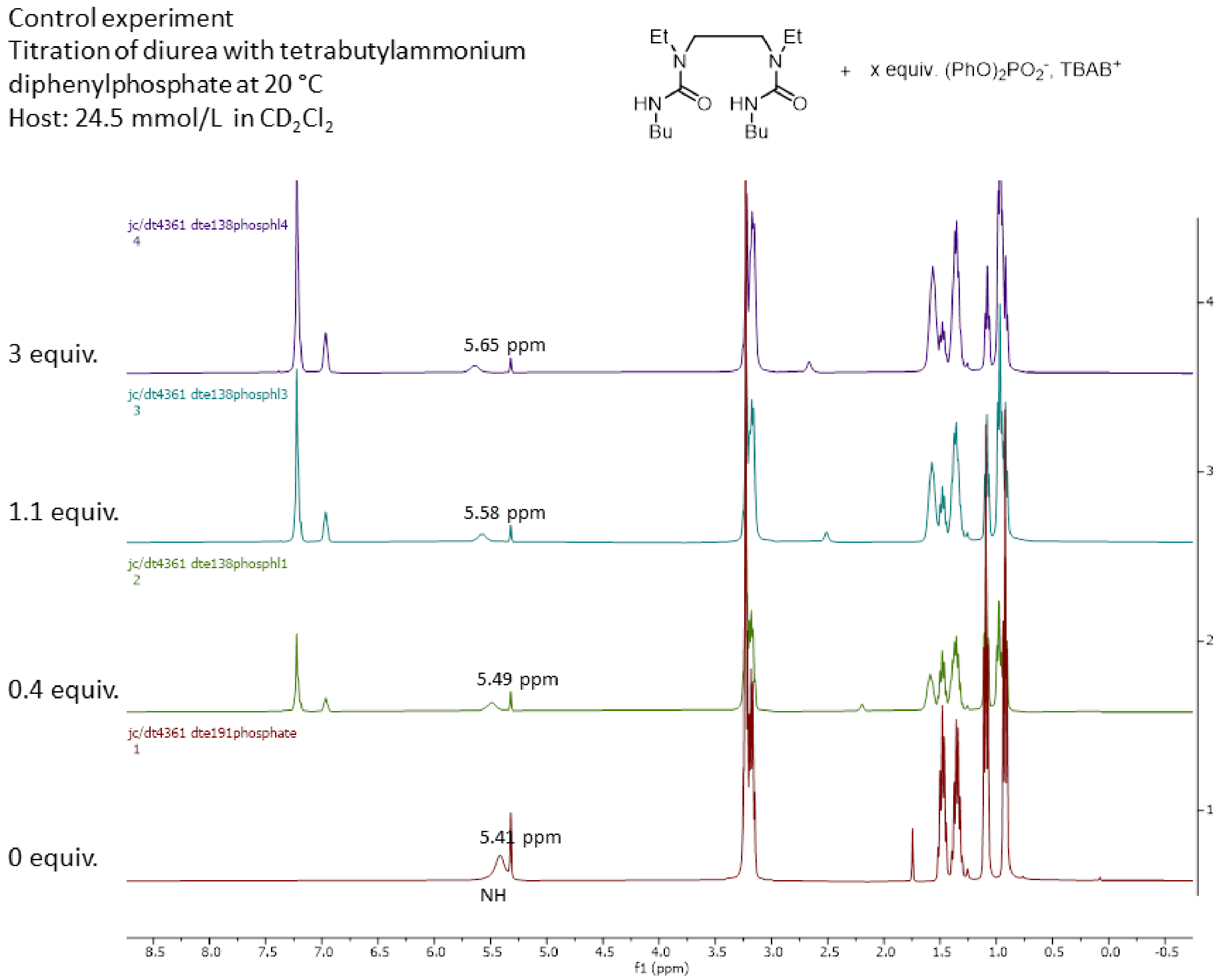

Figure S70: overlay of ^{1}H NMR spectra of **9** (24.5 mM in CD_2Cl_2) in the presence of increasing amounts of tetrabutylammonium diphenylphosphate from 0 to 3 equivalents at 25 °C (300 MHz)

6.4. Compound 10

6.4.1. Titration with tetrabutylammonium chloride in CD_2Cl_2 at 25 °C

^{1}H NMR spectrum of compound **10** (17.2 mM) in CD_2Cl_2 at 25 °C displays one signal at 5.38 ppm for the two ureido NH located at the termini of the hydrogen bond chain. The value of chemical shift is the weighted average of the chemical shifts of NH in hydrogen bonding, and in non-hydrogen-bonding modes (rapid equilibria between hydrogen bond directionalities on the NMR timescale at ambient temperature). Titration of that solution with tetrabutylammonium chloride (from 0 to 2.9 equivalents) was monitored by ^{1}H NMR spectroscopy (figure S71). The ligand was added as a solid so no variation of concentration occurs during the titration. The addition of tetrabutylammonium chloride to the solution causes only minimal chemical shift variation of the NH signals compared to the shifts observed for the titrations described for the triurea hosts above (complexation induced shift (CIS) at 0.9 equivalents of ligand added = 0.23 ppm for 10, compared to downfield shifts in the order of 1.5 ppm for the terminal ureido NH during the titrations of the tri urea hosts with the same quantity of ligand).

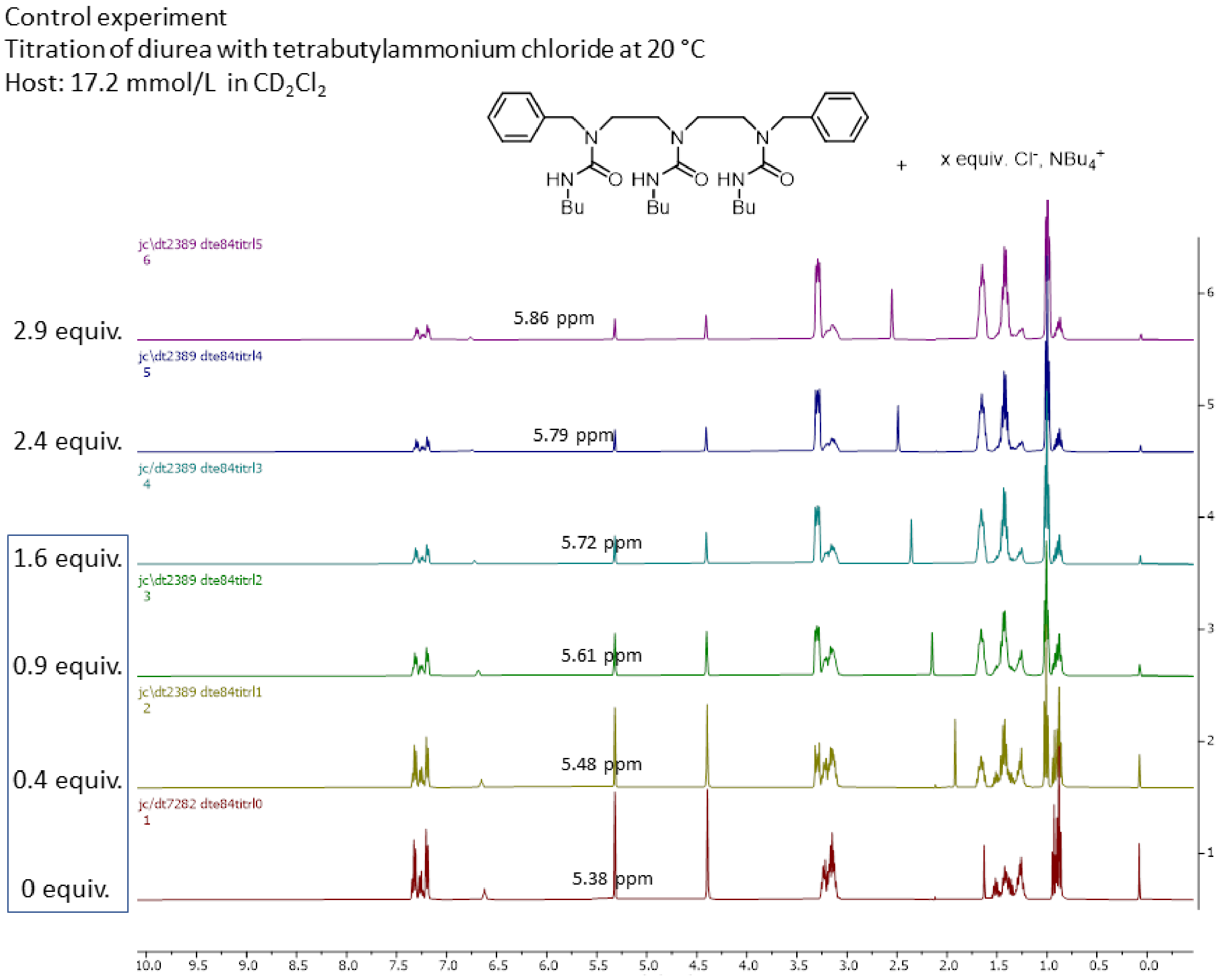

Figure S71: overlay of ^{1}H NMR spectra of **10** (17.2 mM in CD_2Cl_2) in the presence of increasing amounts of tetrabutylammonium chloride from 0 to 2.9 equivalents at 25 °C (300 MHz)

6.4.2. Titration with tetrabutylammonium acetate in CD_2Cl_2 at 25 °C

^{1}H NMR analysis of compound **10** (14.8 mM) in CD_2Cl_2 at 25 °C displays one signal at 5.38 ppm for the two ureas NH at the extremities of the hydrogen bond chain. The value of chemical shift is the weighted average of the chemical shifts of ureido NH in hydrogen bonding, and in non-hydrogen-bonding modes (rapid equilibria between hydrogen bond directionalities on the NMR timescale at ambient temperature). Titration of that solution with tetrabutylammonium acetate (from 0 to 3.9 equivalents) was monitored by ^{1}H NMR spectroscopy (figure S73). The ligand was added as a solid so no variation of concentration occurs during the titration. The addition causes only minimal chemical shift variation of the NH signals comparatively to the shifts observed for the titrations described above (complexation induced shift (CIS) at 1.25 equivalents of ligand added = 0.31 ppm for **10**, compared to downfield shifts in the order of 1.5 ppm for the terminal ureido NH during the titrations with the same quantity of ligand).

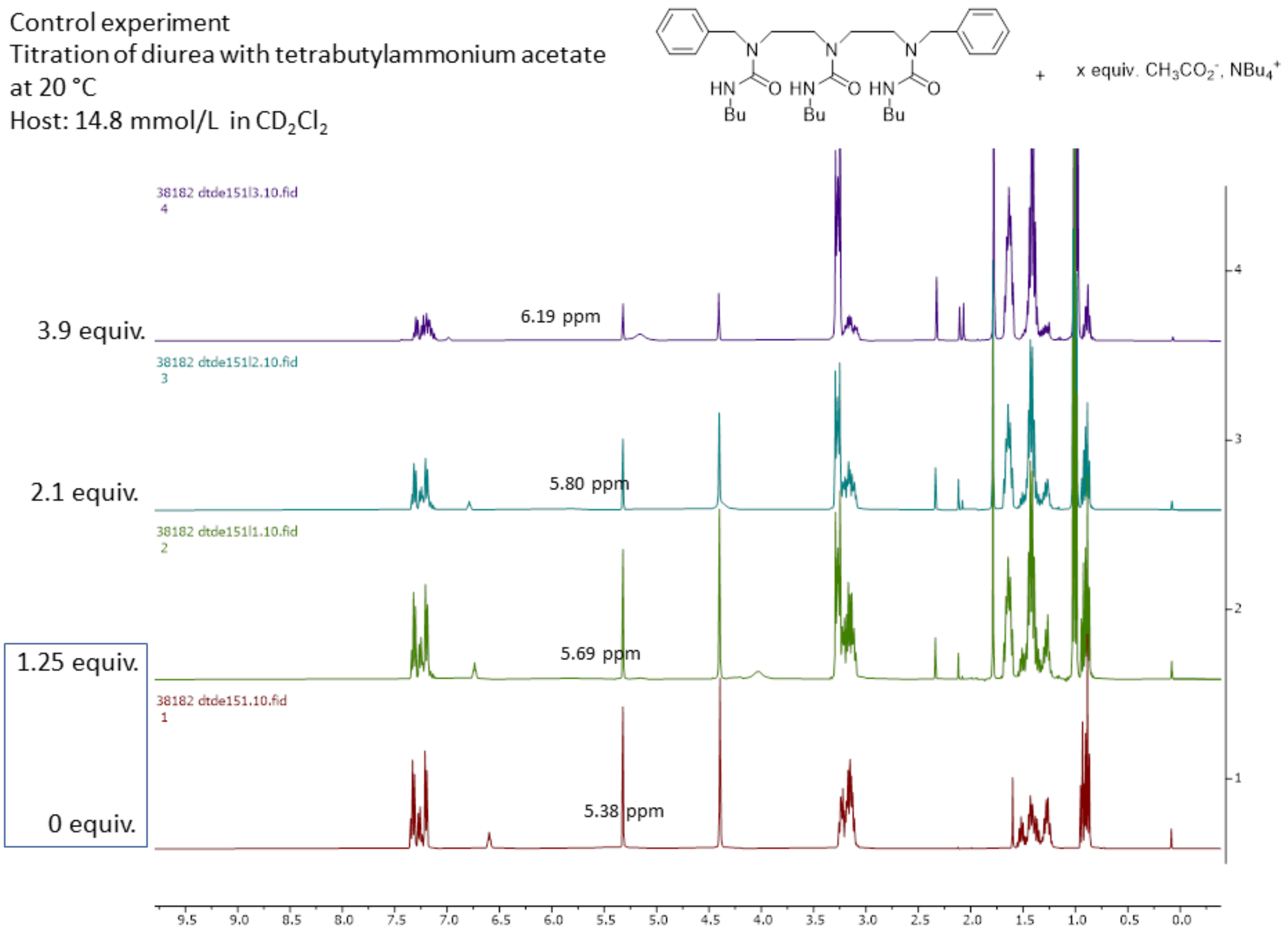

Figure S72: overlay of ^{1}H NMR spectra of **10** (14.8 mM in CD_2Cl_2) in the presence of increasing amounts of tetrabutylammonium acetate from 0 to 3.9 equivalents at 25 °C (300 MHz)

6.4.3. Titration with tetrabutylammonium diphenylphosphate in CD_2Cl_2 at 25 °C

^{1}H NMR spectrum of compound **10** (15 mM) in CD_2Cl_2 at 25 °C displays one signal at 5.32 ppm for the two ureas NH located at the extremities of the hydrogen bond chain. The value of chemical shift is the weighted average of the chemical shifts of NH in hydrogen bonding, and in non-hydrogen-bonding modes (rapid equilibria between hydrogen bond directionalities on the NMR timescale at ambient temperature). A signal for the central ureido NH is at 6.22 ppm. Titration of that solution with tetrabutylammonium diphenylphosphate (from 0 to 6.8 equivalents) was monitored by ^{1}H NMR spectroscopy (figure S72). The ligand was added as a solid so no variation of concentration occurs during the titration. The addition causes only minimal chemical shift variation of the NH signals comparated to the shifts observed for the titrations of the triurea hosts described above (complexation induced shift (CIS) at 1.1 equivalents of ligand added = 0.13 ppm for **10**, compared to downfield shifts in the order of 1.5 ppm for the terminal ureido NH during the titrations of the tri urea hosts with the same quantity of ligand).

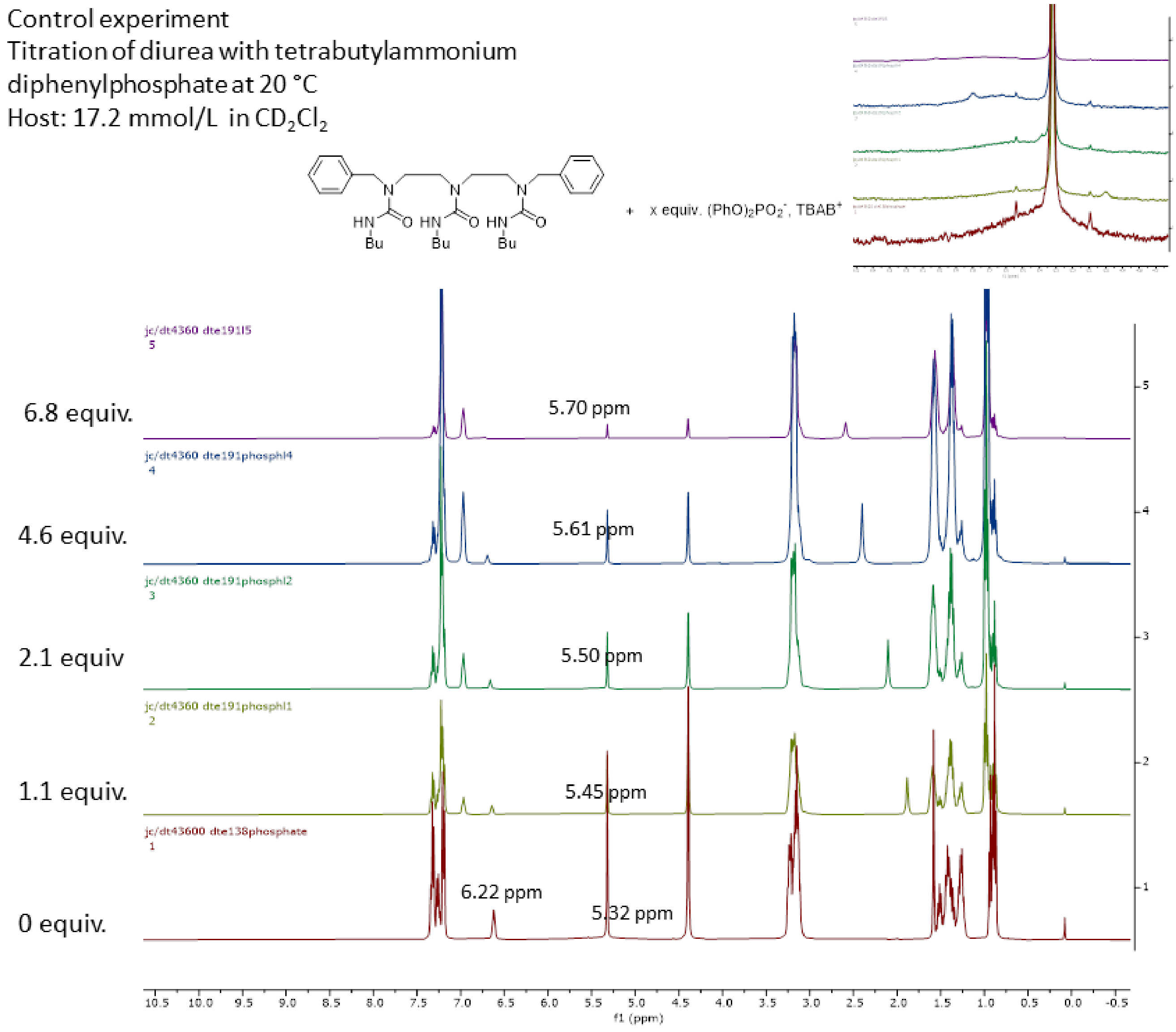

Figure S73: overlay of ^{1}H NMR spectra of **10** (17.2 mM in CD_2Cl_2) in the presence of increasing amounts of tetrabutylammonium diphenylphosphate from 0 to 6.8 equivalents at 25 °C (300 MHz)

6.5. Compound 5

6.5.1. Titration with tetrabutylammonium acetate in CD_2Cl_2 at 25 °C

Titration experiment of **5** (9.8 mM) in CD_2Cl_2 at 25 °C with increasing amounts of tetrabutylammonium acetate (from 0 to 1.57 equivalents) was monitored by ^{1}H NMR (figure S74). The ligand was added as a solid so no variation of concentration occurs during the titration. The values of chemical shifts of the NH of **5** upon addition of tetrabutylammonium acetate were collected in Table S7 and plotted in a graph as {chemical shift of NH = f(ligand added)} (figure S77).

Progressive downfield shifts of both NH^e (CIS 3.17 ppm) and NH^f (CIS 3.02 ppm) signals are observed, this is consistent with the formation of intermolecular hydrogen bonds between the ligand and the host at those positions. The variation of chemical shifts occur essentially from 0 to 1 equivalent of ligand added, further addition of ligand has little effect on the chemical shifts.

The chemical shift variations of NH^b (CIS 0.04 ppm), NH^c (0.48 ppm), NH^d (0.03 ppm) upon addition of ligand is smaller, those NH are involved in intramolecular hydrogen bonds with adjacent ureas no matter the directionality of the hydrogen bond chain so no important variation of chemical shift values of NH^b and NH^c is expected upon change of global hydrogen bond directionality. The signal for NH^a moves downfield (CIS 1.53 ppm), the chemical shifts vary from 0 to 1 equivalent of ligand added then stabilise even more ligand is added. It is consistent with a change of global directionality of the hydrogen bond chain controlled by the binding of acetate at the terminus of the chain.

An association constant for the binding event of acetate ligand to NH^e and NH^f was calculated by non-linear curve fitting analysis of the experimental titration curves and comparing the results with theoretical binding isotherms for 1:1, 1:2, 2:1 binding modes using supramolecular.org. The best fit was obtained with a 1:2 binding isotherm with K1:1 684 +/-29%, K1:2 = 76 +/-31%. Titration at lower concentrations to determine more accurately a binding constant value was not possible as NH signals become difficult to see at lower concentrations.

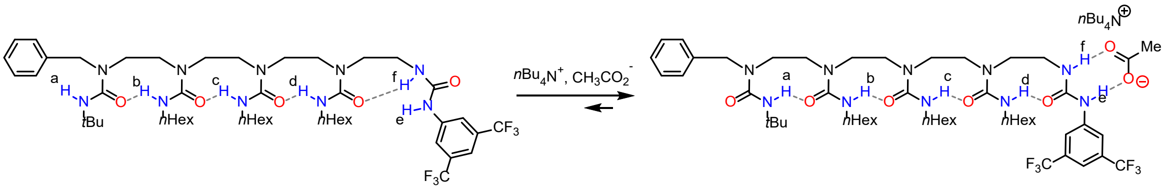

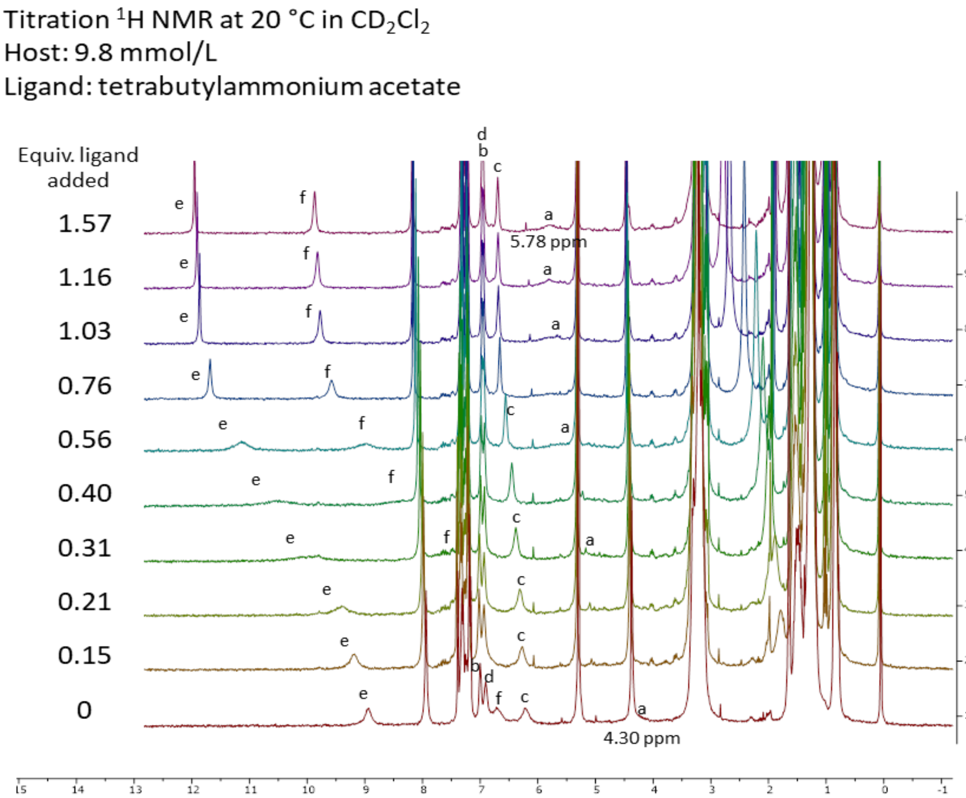

Figure S74: overlay of ^{1}H NMR spectra of **5** (9.8 mM in CD_2Cl_2) in the presence of increasing amounts of tetrabutylammonium acetate from 0 to 1.57 equivalents at 25 °C (300 MHz)

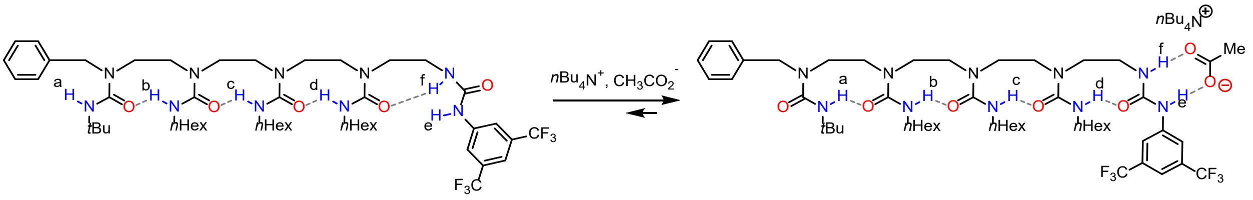

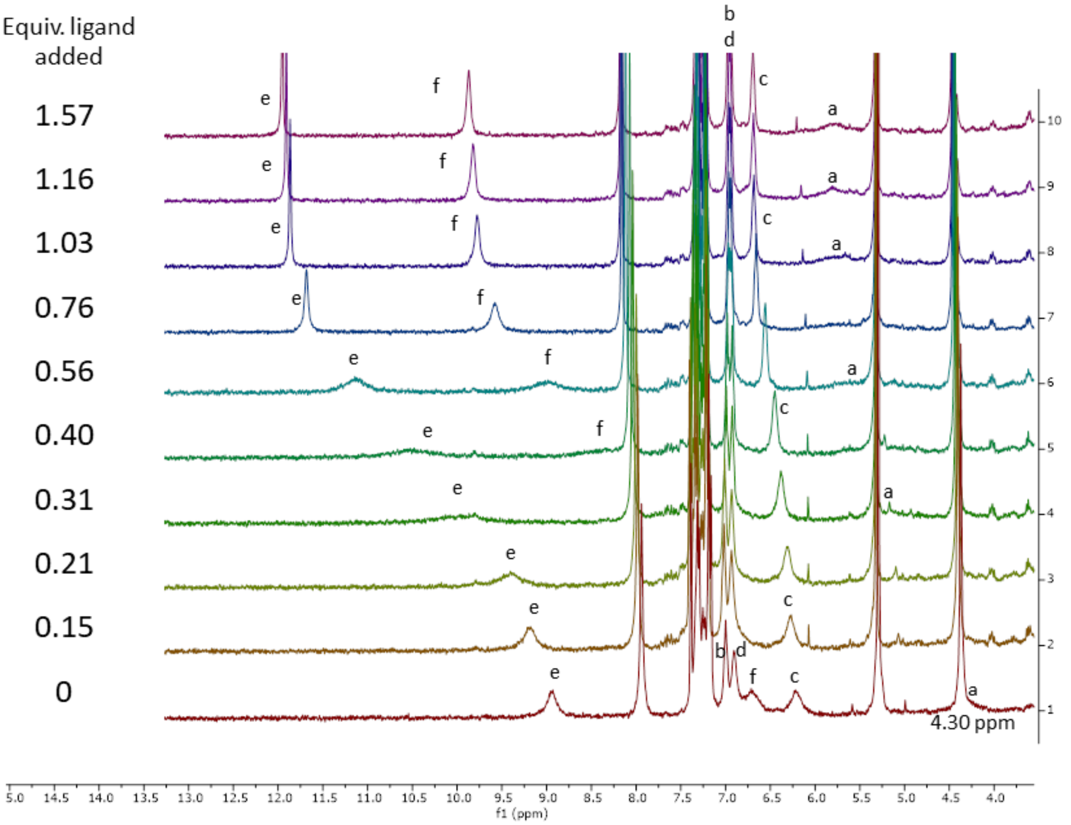

Figure S75: overlay of ^{1}H NMR spectra of **5** (9.8 mM in CD_2Cl_2) in the presence of increasing amounts of tetrabutylammonium acetate from 0 to 1.57 equivalents at 25 °C (300 MHz) – enlargement of aromatic and NH region

Concentration of ligand (mol/L)	Chemical shift NHe (ppm)	Chemical shift NHf (ppm)	Chemical shift NHa (ppm)	Chemical shift NHb (ppm)	Chemical shift NHc (ppm)	Chemical shift NHd (ppm)
0	8.93	6.7	4.25	7	6.21	6.91
0.00147	9.18	overlap	broad	7.02	6.27	6.93
0.002058	9.39	overlap	broad	7.01	6.31	6.93
0.003038	9.99	overlap	broad	6.99	6.38	6.93
0.00392	10.53	8.36	broad	6.98	6.45	6.93
0.005488	11.13	8.98	broad	6.97	6.56	6.93
0.007448	11.68	9.57	broad	6.96	6.66	6.94
0.010094	11.86	9.77	5.65	6.96	6.68	6.94
0.011368	11.91	9.82	5.77	6.96	6.69	6.94
0.015386	11.95	9.87	5.78	6.96	6.69	6.94
**CIS (ppm)**	**3.02**	**3.17**	**1.53**	**-0.04**	**0.48**	**0.03**

Table S7. chemical shifts of ^{1}H NMR NH signals recorded during the titration of **5** (9.8 mM in CD_2Cl_2) with tetrabutylammonium acetate, recorded at 25 °C (300 MHz).

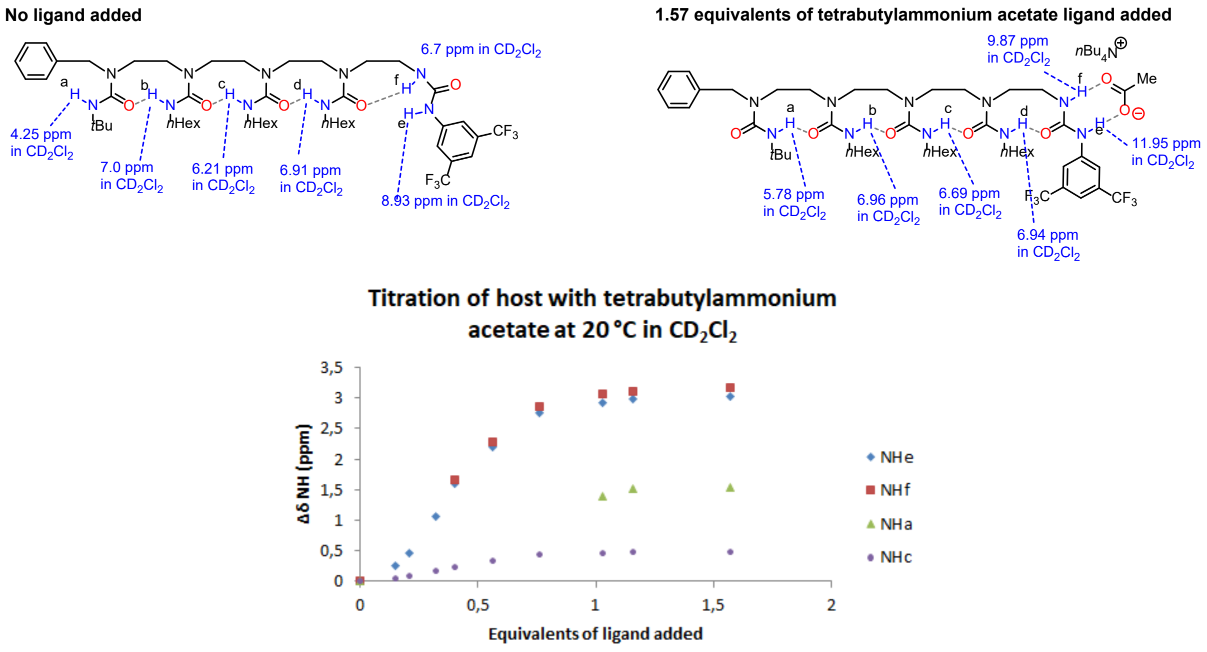

Figure S76: ^{1}H NMR chemical shifts of **5** NH signals (9.8 mM in CD_2Cl_2) before and after addition of 1.57 equivalents of tetrabutylammonium acetate at 25 °C.

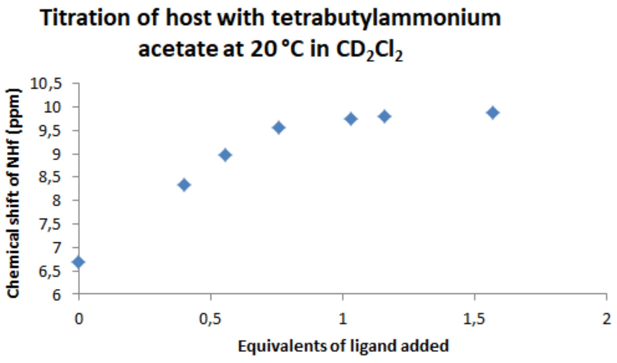

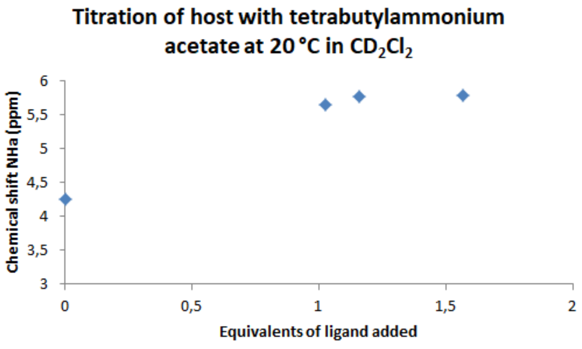

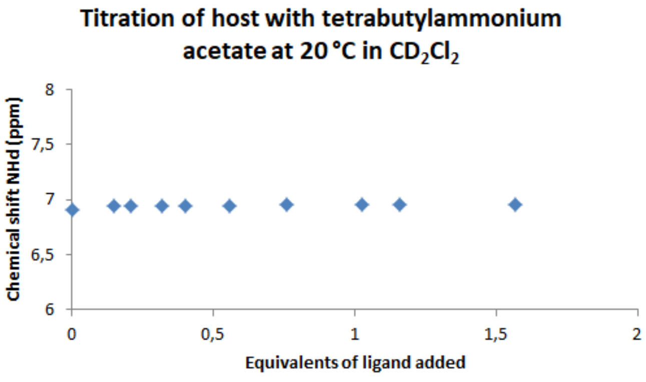

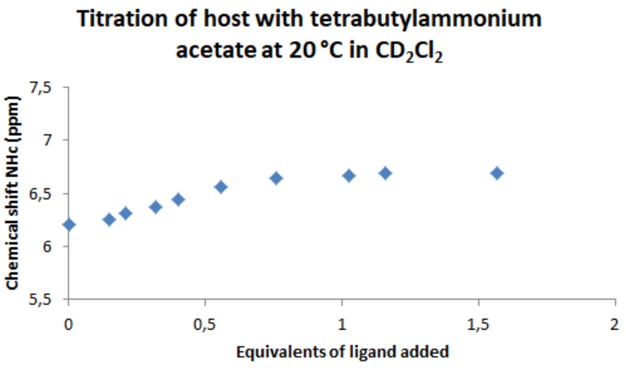

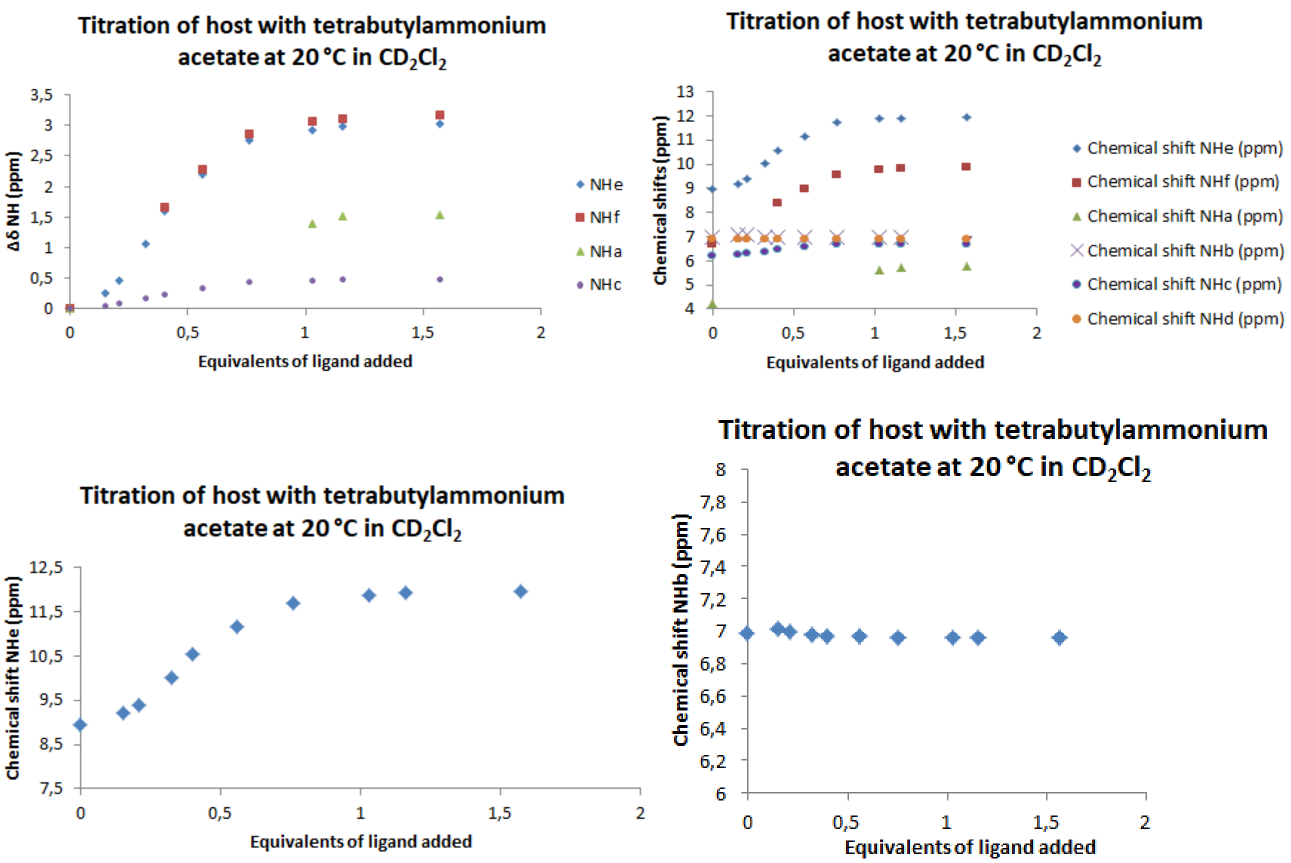

Figure S77: plots of variation of chemical shifts of NH signals of **5** (9.8 mM in CD_2Cl_2) and plot of variation of chemical induced shifts of NH signals upon addition of increments of tetrabutylammonium acetate recorded by ^{1}H NMR at 25 °C (300 MHz).

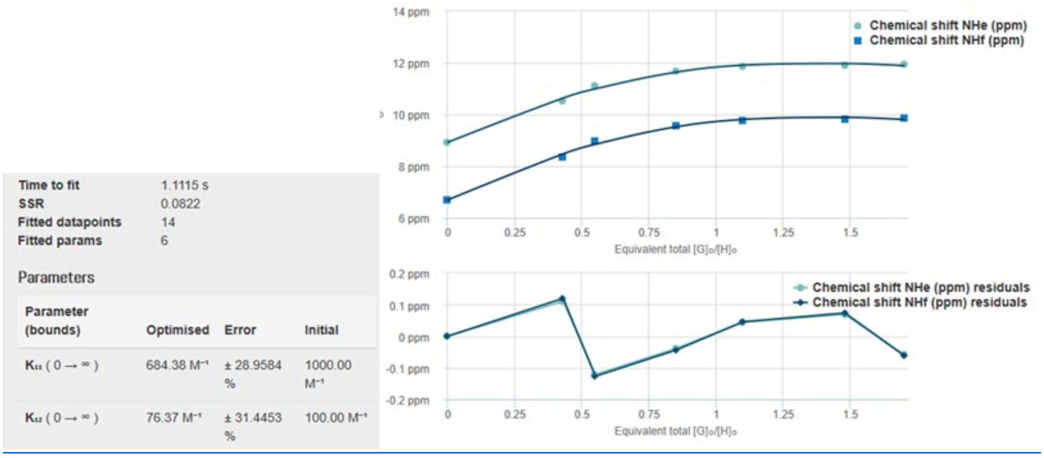

Time to fit	1.1115 s
SSR	0.0822
Fitted datapoints	14
Fitted params	6

Parameters

Parameter (bounds)	Optimised	Error	Initial
K_{11} (0 → ∞)	684.38 M^{-1}	± 28.9584 %	1000.00 M^{-1}
K_{12} (0 → ∞)	76.37 M^{-1}	± 31.4453 %	100.00 M^{-1}

Figure 78: non-linear curve fitting analysis of the experimental titration data of **5** (9.8 mM in CD_2Cl_2) with increments of tetrabutylammonium acetate recorded at 25 °C (400 MHz) using a theoretical binding isotherm for 1:2 binding http://app.supramolecular.org/bindfit/view/9afdc154-02b1-4594-b9f4-453437e118f7

6.5.2. Titration with tetrabutylammonium chloride in CD_2Cl_2 at 25 °C

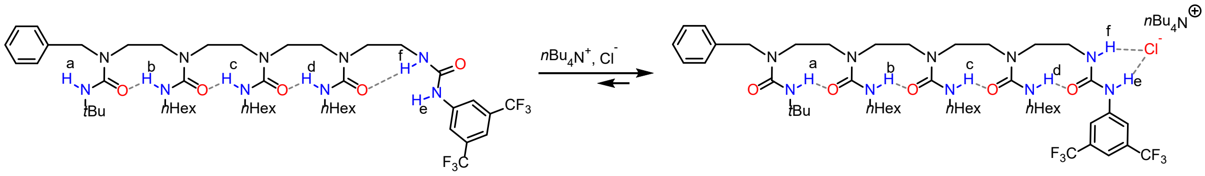

Titration experiment of **5** (9.8 mM) in CD_2Cl_2 at 25 °C with increasing amounts of tetrabutylammonium chloride (from 0 to 1.44 equivalents) was monitored by ^{1}H NMR (figure S79). The ligand was added as a solid so no variation of concentration occurs during the titration. The values of chemical shifts of the NH of **5** upon addition of tetrabutylammonium chloride were collected in Table S7 and plotted in a graph as {chemical shift of NH = f(ligand added)} (figure S81).

Progressive downfield shifts of both NH^e (CIS 1.51 ppm) and NH^f (CIS 0.91 ppm) signals are observed, this is consistent with the formation of intermolecular hydrogen bonds between the ligand and the host at those positions. The variation of chemical shifts occur essentially from 0 to 1 equivalent of ligand added, further addition of ligand has little effect on the chemical shifts. Comparatively, the chemical shift variations of NH^b (CIS 0.12 ppm), NH^c (0.32 ppm), NH^d (0.05 ppm) upon addition of ligand are smaller, those NH are involved in intramolecular hydrogen bonds with adjacent ureas no matter the directionality of the hydrogen bond chain so no important variation of chemical shift value is expected upon change of hydrogen bond directionality. NH^a signal was too broad to be seen by ^{1}H NMR.

An association constant for the binding event of chloride ligand to NH^e and NH^f was calculated by non-linear curve fitting analysis of the experimental titration curves and comparing the results with theoretical binding isotherms for 1:1, 1:2, 2:1 binding modes using supramolecular.org. The best fit was obtained using a 1:2 binding isotherm with association constants of K1:1 = 842 +/-28%, K1:2 = 50 +/-38%. Titration at lower concentrations to determine more accurately a binding constant value was not possible as NH signals become difficult to see at lower concentrations.

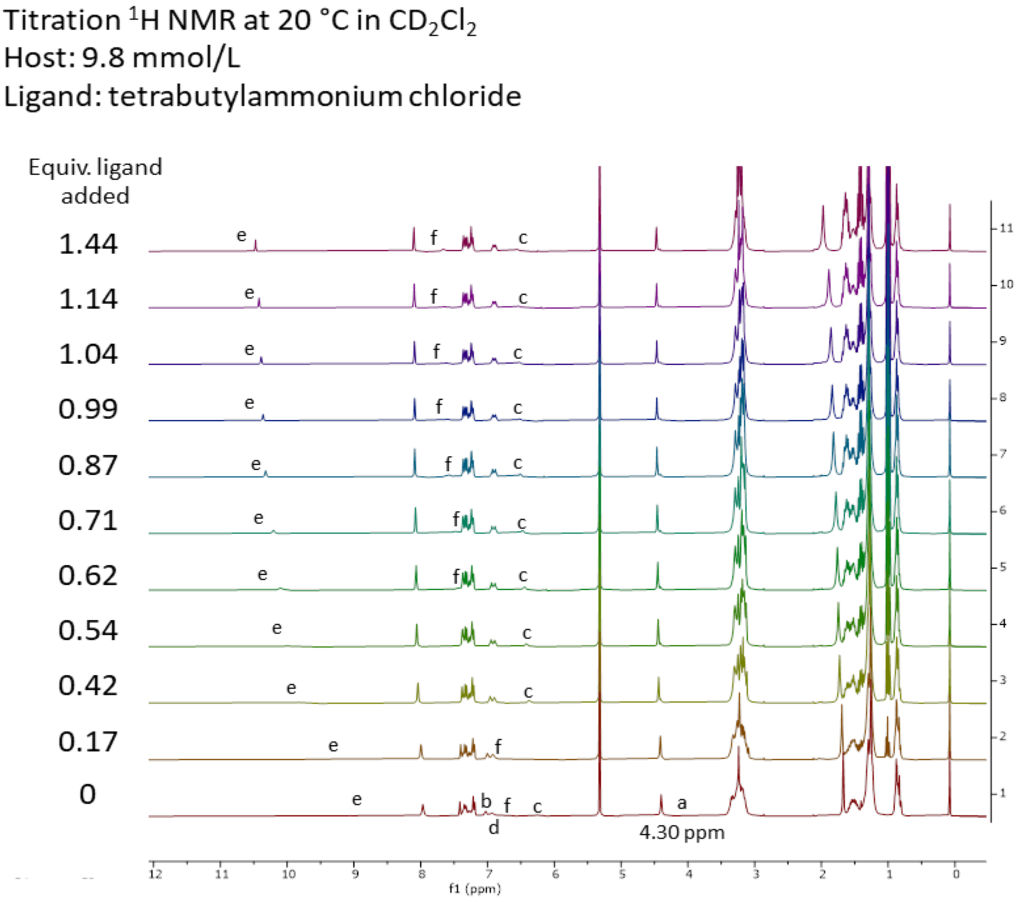

Figure S79: overlay of ^{1}H NMR spectra of **5** (9.8 mM in CD_2Cl_2) in the presence of increasing amounts of tetrabutylammonium chloride from 0 to 1.44 equivalents, recorded at 25 °C (300 MHz)

Concentration host (mol/L)	Equivalents of ligand	Concentration ligand (mol/L)	Chemical shift NH^e (ppm)	Chemical shift NH^f (ppm)	Chemical shift NH^a (ppm)	Chemical shift NH^b (ppm)	Chemical shift NH^c (ppm)	Chemical shift NH^d (ppm)
0.0098	0	0	8.97	6.75	4.3	7.03	6.23	6.93
0.0098	0.17	0.001666	9.29	6.87		7	6.29	6.92
0.0098	0.42	0.004116	9.8			6.96	6.37	6.9
0.0098	0.54	0.005292	9.98			6.95	6.42	6.89
0.0098	0.62	0.006076	10.11	7.43		6.94	6.45	6.89
0.0098	0.71	0.006958	10.2	7.49		6.93	6.48	6.89
0.0098	0.87	0.008526	10.32	7.57		6.92	6.51	6.88
0.0098	0.99	0.009702	10.36	7.61		6.92	6.53	6.88
0.0098	1.04	0.010192	10.39	7.61		6.92	6.53	6.88
0.0098	1.14	0.011172	10.42	7.63		6.92	6.54	6.88
0.0098	1.44	0.014112	10.48	7.66		6.91	6.55	6.88
	**CIS (ppm)**		**1.51**	**0.91**		**-0.12**	**0.32**	**-0.05**

Table S7. chemical shifts of ^{1}H NH signals recorded during the NMR titration of **5** (9.8 mM in CD_2Cl_2) with increments of tetrabutylammonium chloride at 25 °C (300 MHz).

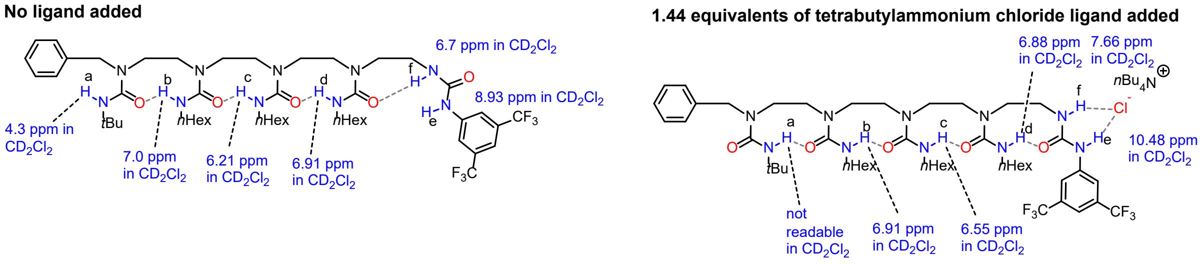

Figure S80: ^{1}H NMR chemical shifts of NH signals of **5** (9.8 mM in CD_2Cl_2) before and after addition of 1.57 equivalents of tetrabutylammonium chloride at 25 °C.

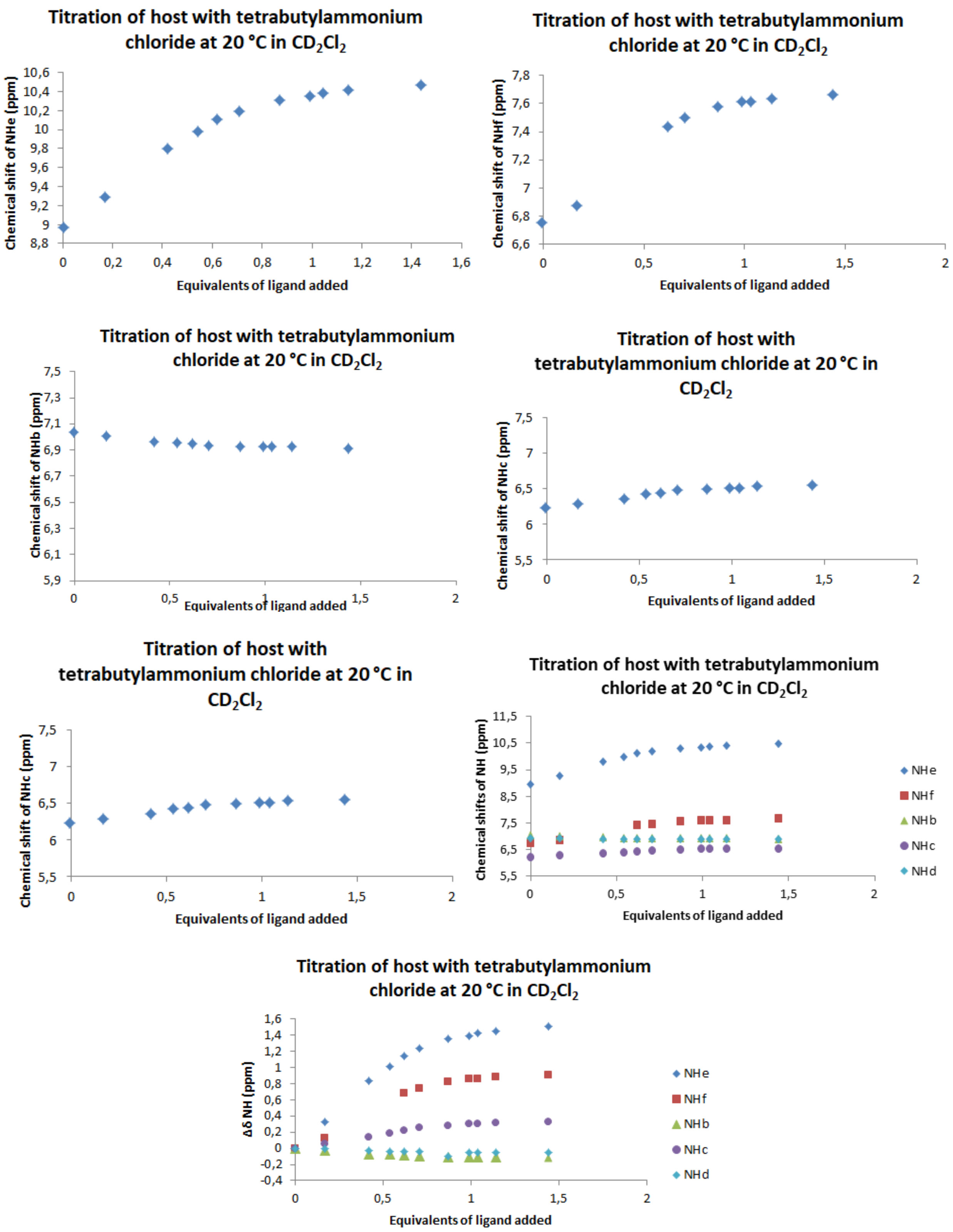

Figure S81: plots of variation of chemical shifts of NH signals of **5** (9.8 mM in CD_2Cl_2) and plot of variation of chemical induced shifts of NH signals upon addition of increments of tetrabutylammonium chloride recorded by ^{1}H NMR at 25 °C (300 MHz).

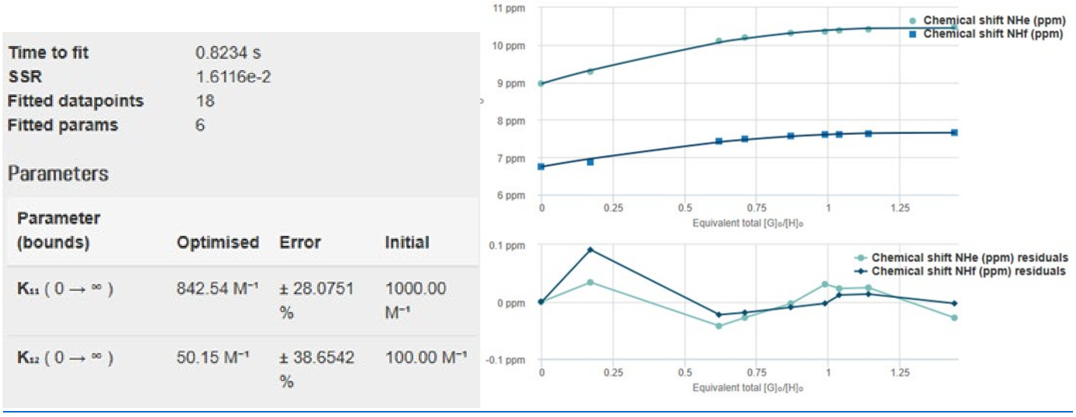

Figure S82: non-linear curve fitting analysis of the experimental titration data of **5** (9.8 mM in CD_2Cl_2) with increments of tetrabutylammonium chloride recorded at 25 °C (400 MHz) using a theoretical binding isotherm for 1:2 binding http://app.supramolecular.org/bindfit/view/f448b6f9-6a8e-4e9e-b54f-2d6bd63f335e

6.5.3. Titration with tetrabutylammonium diphenylphosphate in CD_2Cl_2 at 25 °C

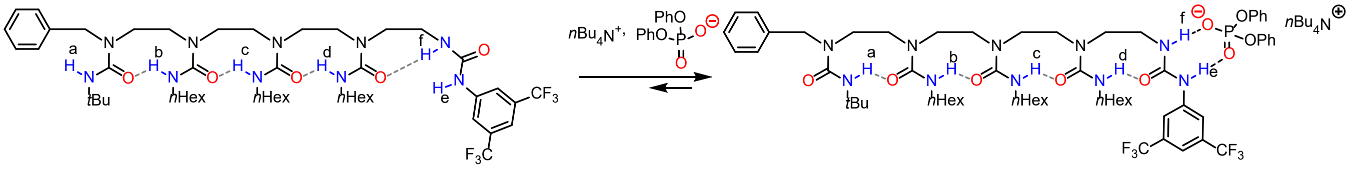

Titration experiments of **5** (9.8 mM) in CD_2Cl_2 at 25 °C with increasing amounts of tetrabutylammonium diphenylphosphate (from 0 to 2.2 equivalents) were monitored by ^1H NMR (figures S83-S84). The ligand was added as a solid so no variation of concentration occurs during the titration. The values of chemical shifts of the NH of **5** upon addition of tetrabutylammonium diphenylphosphate were collected in Table S7 and plotted in a graph as {chemical shift of NH = f(ligand added)} (figure S86).

Progressive downfield shifts of both NH^e (CIS 1.47 ppm) and NH^f (CIS 0.84 ppm) signals are observed, this is consistent with the formation of intermolecular hydrogen bonds between the ligand and the host at those positions. The variation of chemical shifts occur essentially from 0 to 1 equivalent of ligand added, further addition of ligand has little effect on the chemical shifts. Comparatively, the chemical shift variation of NH^c (CIS 0.5 ppm) upon addition of ligand is smaller, NH^b, NH^c and NH^d are involved in intramolecular hydrogen bonds no matter the global directionality of the hydrogen bond chain so no important variation of chemical shift value is expected upon change of global hydrogen bond directionality. The signal for NH^a moves downfield (CIS 1.5 ppm), the chemical shifts vary from 0 to 1 equivalent of ligand added then stabilise even more ligand is added. It is consistent with a change of global directionality of the hydrogen bond chain controlled by the binding of diphenylphosphate anion at the terminus of the chain.

Attempts to estimate an association constant for the binding event of tetrabutylammonium diphenylphosphate to NH^e and NH^f by non-linear curve fitting analysis of the experimental titration curves and comparing the results with theoretical binding isotherms for 1:1, 1:2, 2:1 binding modes using supramolecular.org, led to high errors. Titration at lower concentrations to determine more accurately a binding constant value was not possible as NH signals become difficult to see at lower concentrations.

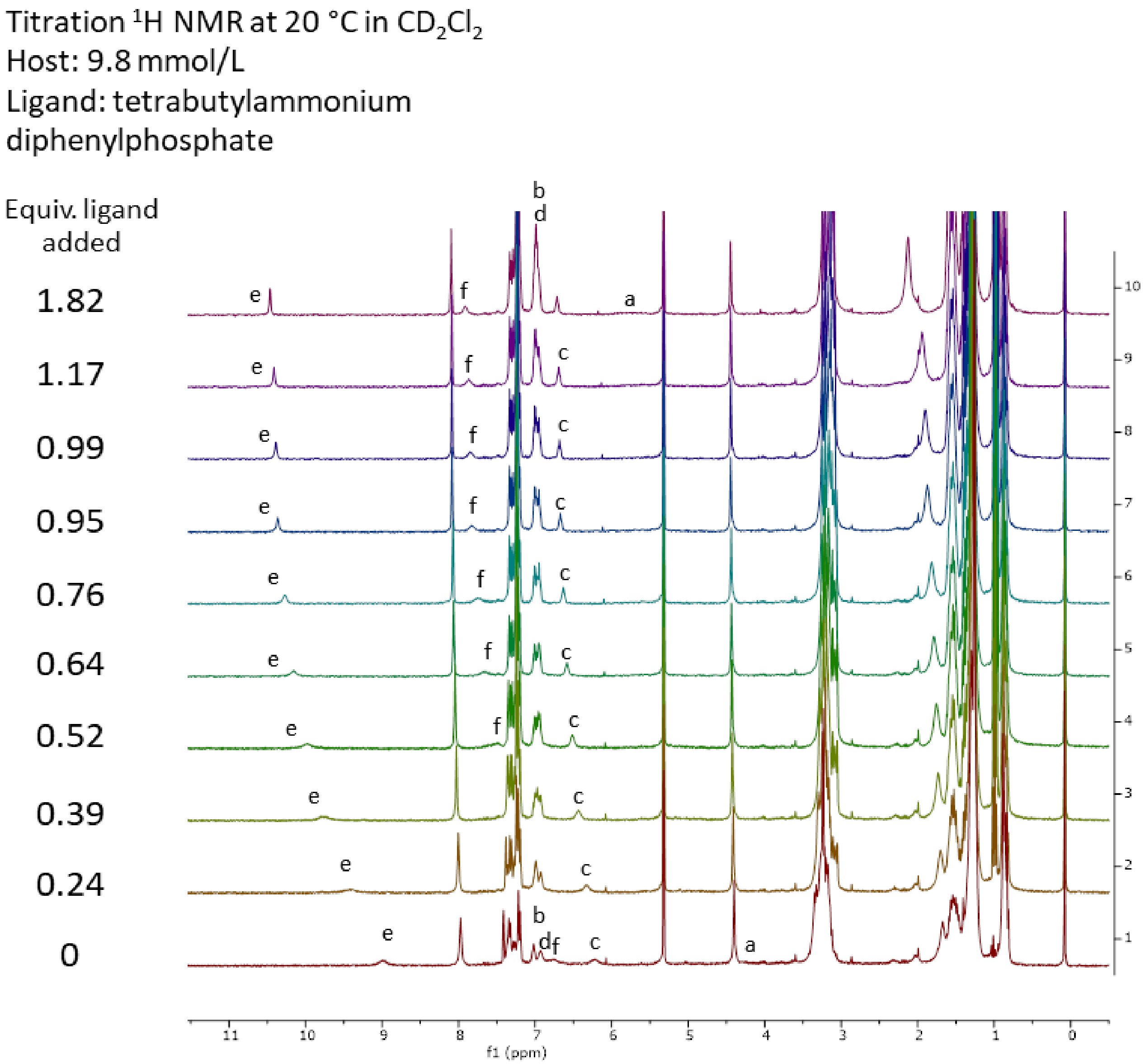

Figure S83: overlay of ^{1}H NMR spectra of **5** (9.8 mM in CD_2Cl_2) in the presence of increasing amounts of tetrabutylammonium diphenylphosphate from 0 to 1.82 equivalents, recorded at 25 °C (300 MHz)

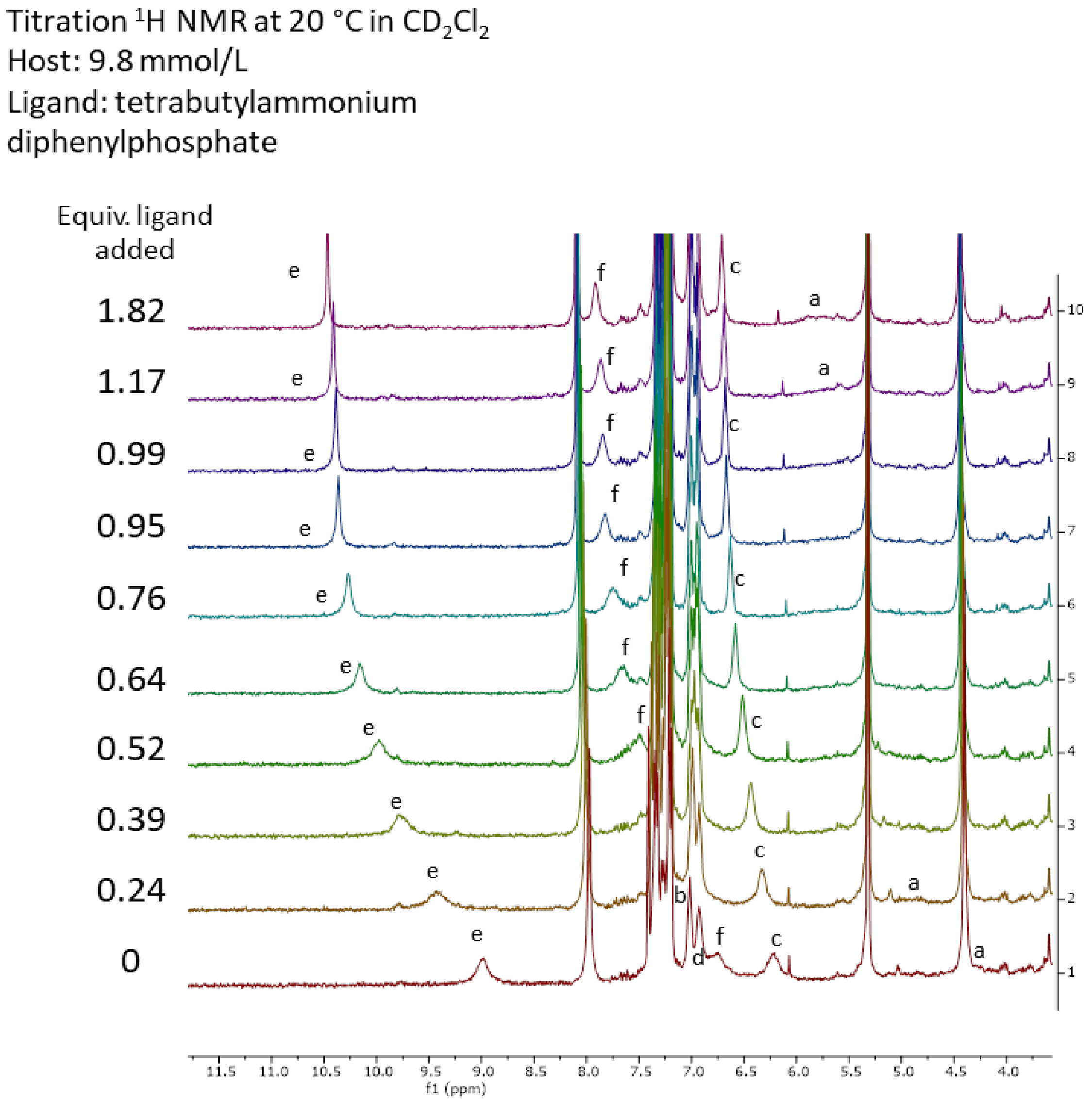

Figure S84: overlay of ^{1}H NMR spectra of **5** (9.8 mM in CD_2Cl_2) in the presence of increasing amounts of tetrabutylammonium diphenylphosphate from 0 to 1.82 equivalents recorded at 25 °C (300 MHz) – enlargement of aromatic and NH region

Concentration host (mol/L)	Equivalents ligand	Concentration ligand (mol/L)	Chemical shift NH[e] (ppm)	Chemical shift NH[f] (ppm)	Chemical shift NH[a] (ppm)	Chemical shift NH[b] (ppm)	Chemical shift NH[c] (ppm)	Chemical shift NH[d] (ppm)
0.0098	0	0	8.99	6.75	4.3	7.02	6.22	6.93
0.0098	0.121	0.0011858	9.42				6.33	
0.0098	0.387	0.0037926	9.78				6.43	
0.0098	0.495	0.004851	9.97	7.49			6.51	
0.0098	0.6087	0.00596526	10.16	7.66			6.58	
0.0098	1.02	0.009996	10.36	7.83			6.67	
0.0098	1.244	0.0121912	10.41	7.87			6.69	
0.0098	2.2	0.02156	10.46	7.91	5.8		6.71	
	**CIS (ppm)**		**1.47**	**1.16**	**1.5**		**0.49**	

Table S7. chemical shifts of ^{1}H NH signals recorded during the NMR titration of **5** (9.8 mM in CD_2Cl_2) with increments of tetrabutylammonium diphenylphosphate recorded at 25 °C (300 MHz).

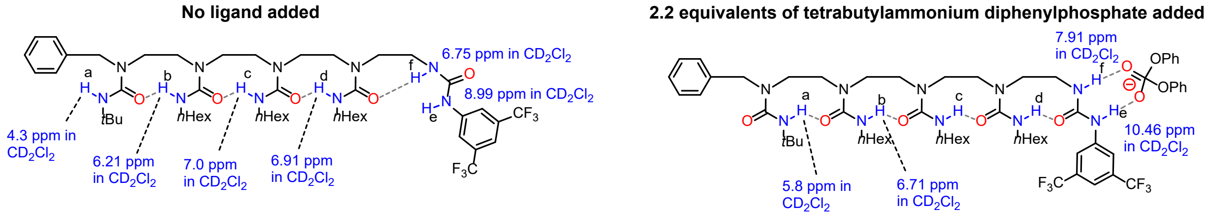

Figure S85: ^{1}H NMR chemical shifts of **5** NH signals (9.8 mM in CD_2Cl_2) before and after addition of 2.2 equivalents of tetrabutylammonium diphenylphosphate at 25 °C.

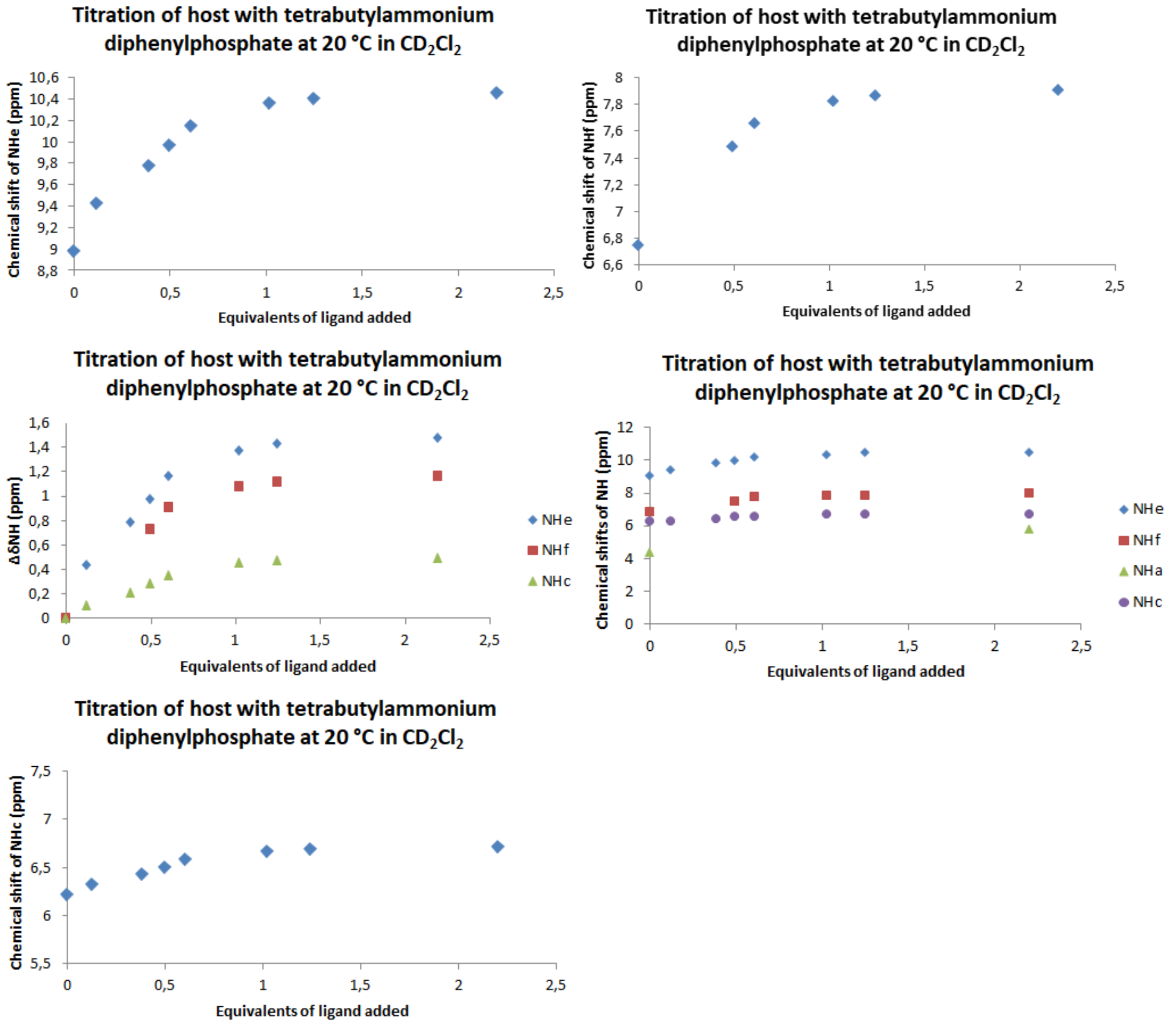

Figure S86: plots of variation of chemical shifts of NH signals of **5** (9.8 mM in CD_2Cl_2) and plot of variation of chemical induced shifts of NH signals upon addition of increments of tetrabutylammonium diphenylphosphate recorded by ^{1}H NMR at 25 °C (300 MHz).

6.6. Compound 1 - Titration with tetrabutylammonium acetate in CD_2Cl_2 at 25 °C

Titration experiment of **1** (14.2 mM) in CD_2Cl_2 at 25 °C with increasing amounts of tetrabutylammonium acetate (from 0 to 1.84 equivalents) was monitored by ^1H NMR (figure S87). The ligand was added as a solid so no variation of concentration occurs during the titration. The assignments of the proton signals during the titration were confirmed by ^1H-^1H COSY and 2D NOESY NMR experiments on the mixture {1 + ligand} (figures S88, S89). For example at 1.95 equivalents of tetrabutylammonium acetate added, ^1H-^1H COSY shows coupling between H^4 and NH^{11} allowing for the assignment of the signal NH^{11}; 2D NOESY displays cross peaks between NH^{20} with both H^{19} and NH^{11} allowing for unambiguous assignment of those signals; cross peak between benzylic H^{10} and H^7, between H^7 with NH^{22}, between NH^{22} with H^{17} also allow for assignment of NH^{22} signal.

The values of chemical shifts of the NH signals of 1 upon addition of tetrabutylammonium acetate were collected in Table S8 and plotted in a graph as {chemical shift of NH = f(ligand added)} (figure S90). Progressive downfield shifts of both NH^{11} (CIS 3.67 ppm) and NH^{20} (CIS 3.79 ppm) signals are observed, those downfield shifts occurred mostly from 0 to 1 equivalent of ligand added. This is consistent with the formation of intermolecular hydrogen bonds between the ligand and the host at those positions.

The variation of chemical shift of NH^{21} upon addition of ligand is comparatively smaller (CIS 0.35 ppm downfield), NH^{21} is involved in intramolecular hydrogen bonds with adjacent urea carbonyls no matter the directionality of the hydrogen bond chain, the variation of chemical shift value for inner ureido NH such as NH^{21} is expected to be less upon change of hydrogen bond directionality.

The signal for NH^{22} moves downfield (CIS 0.8 ppm), which is consistent with a global change of directionality of the hydrogen bond chain.

2D NOESY NMR experiment on the mixture {1 + 1.95 equivalents ligand} (figure S89) displays cross peak between NH^{21} with both H^5 and H^{18}, no cross peak is observed between NH^{22} and benzylic H^{10}. Overall the data support a change of hydrogen bond chain directionality as drawn.

An association constant between the acetate ligand and NH^{11} and NH^{20} was estimated by non-linear curve fitting analysis of the experimental titration data and comparing the results with theoretical binding isotherms for 1:1, 1:2, 2:1 binding modes using supramolecular.org, a 1:1 binding model was fitting best with an estimated value of K association = 1231 +/- 22%. A more accurate measurement of a binding constant for that model was not necessary as the native directionality of the hydrogen bond chain did not correspond to our selection criteria for our design of molecular device.

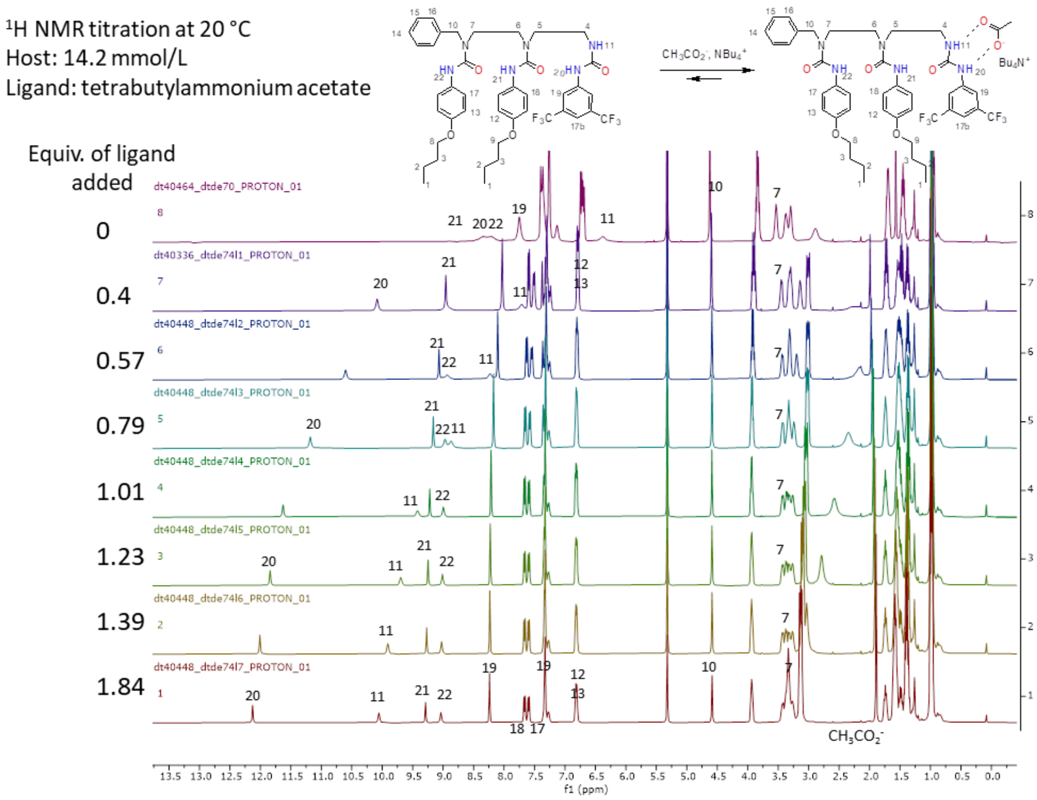

Figure S87: overlay of ^{1}H NMR spectra of **1** (14.2 mM) in the presence of increasing concentrations of tetrabutylammonium acetate, recorded in CD_2Cl_2 at 20°C (300 MHz).

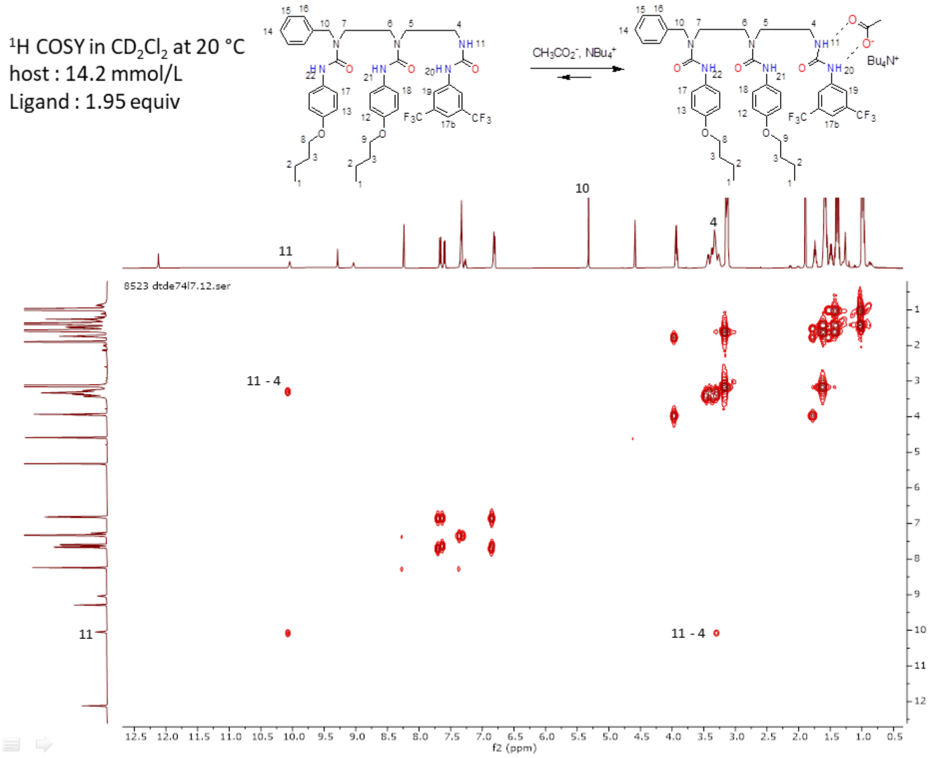

Figure S88: ^{1}H-^{1}H COSY NMR spectrum of **1** (14.2 mM in CD_2Cl_2) in the presence of tetrabutylammonium acetate (1.95 equivalents), recorded at 25 °C (500 MHz).

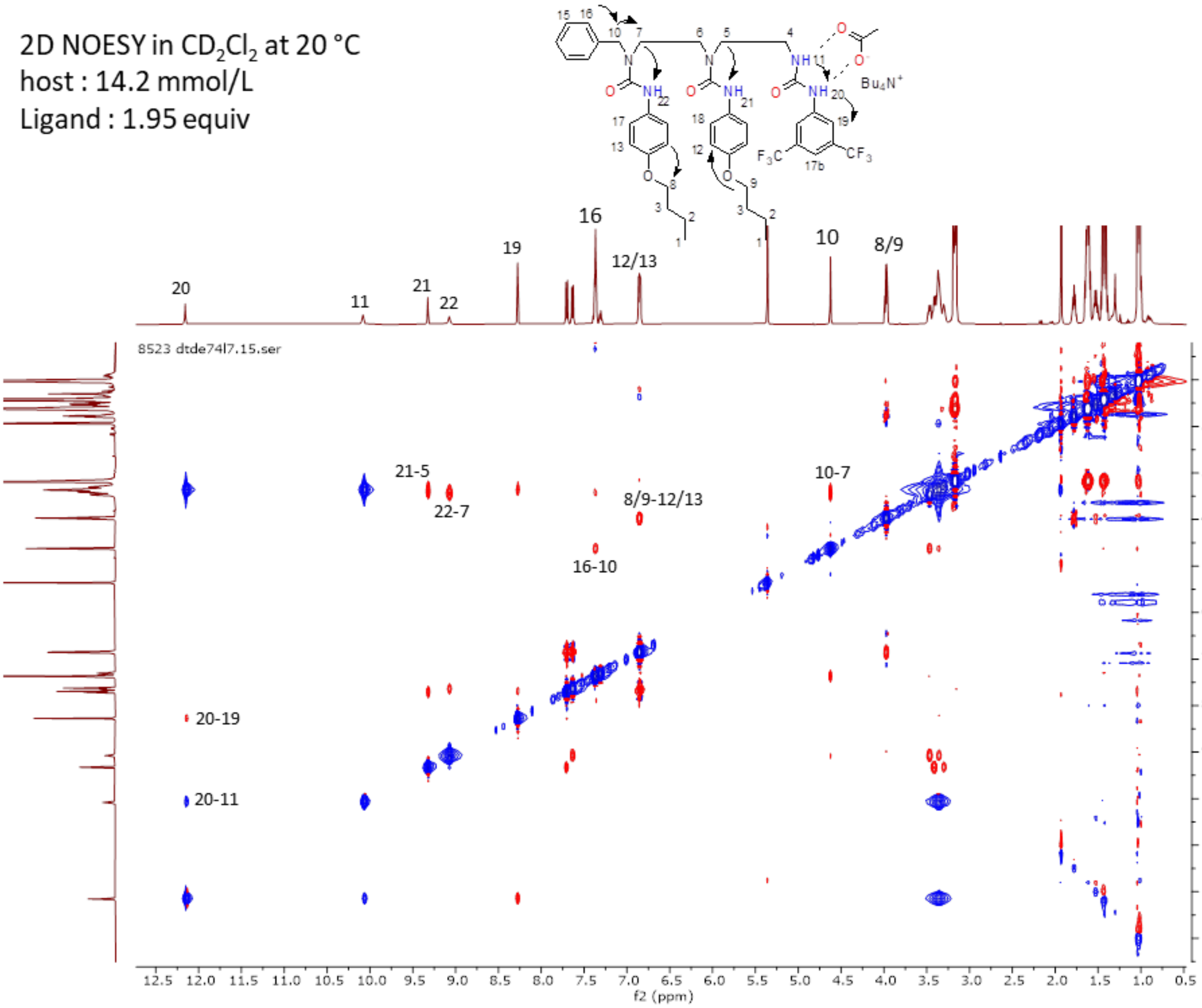

Figure S89: ^1H-^1H 2D NOESY NMR spectrum of **1** (14.2 mM in CD_2Cl_2) in the presence of tetrabutylammonium acetate (1.95 equivalents), recorded at 25 °C (500 MHz).

Concentration of host (mol/L)	Equivalents of acetate ligand	Concentration of acetate (mol/L)	Chemical shift of NH^{11} (ppm)	Chemical shift of NH^{20} (ppm)	Chemical shift of NH^{22} (ppm)	Chemical shift of NH^{21} (ppm)
0.0142	0	0	6.38	8.34	8.24	8.94
0.0142	0.4	0.00567	7.71	10.09	8.89	8.96
0.0142	0.57	0.0081	8.23	10.6	8.93	9.07
0.0142	0.79	0.0112	8.86	11.18	8.97	9.16
0.0142	1.01	0.0143	9.42	11.63	8.99	9.22
0.0142	1.23	0.0174	9.7	11.85	9.01	9.25
0.0142	1.39	0.0197	9.91	12.01	9.03	9.27
0.0142	1.84	0.0261	10.05	12.13	9.04	9.29
**CIS (ppm)**			**3.67**	**3.79**	**0.8**	**0.35**

Table S8. chemical shifts of ^1H NH signals recorded during the NMR titration of **1** (14.2 mM in CD_2Cl_2) with increments of tetrabutylammonium acetate at 25 °C (400 MHz).

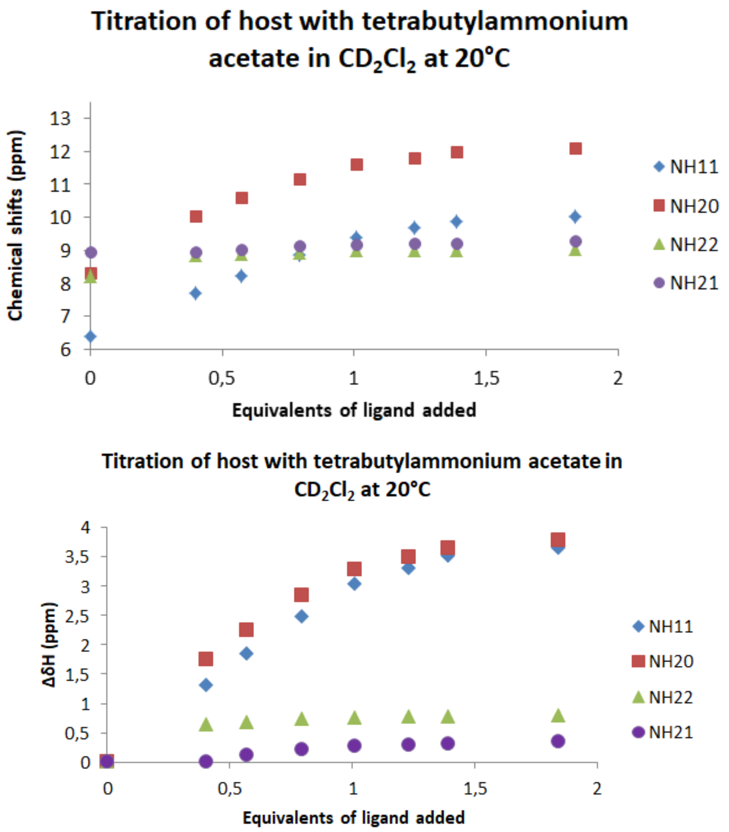

Figure S90: plot of variation of chemical shifts of NH signals of **1** (14.2 mM in CD_2Cl_2) upon addition of increments of tetrabutylammonium acetate, recorded by ^{1}H NMR at 25 °C (400 MHz); and plot of the chemical shift variations of NH signals of 1 (14.2 mM in CD_2Cl_2) upon addition of increments of tetrabutylammonium acetate.

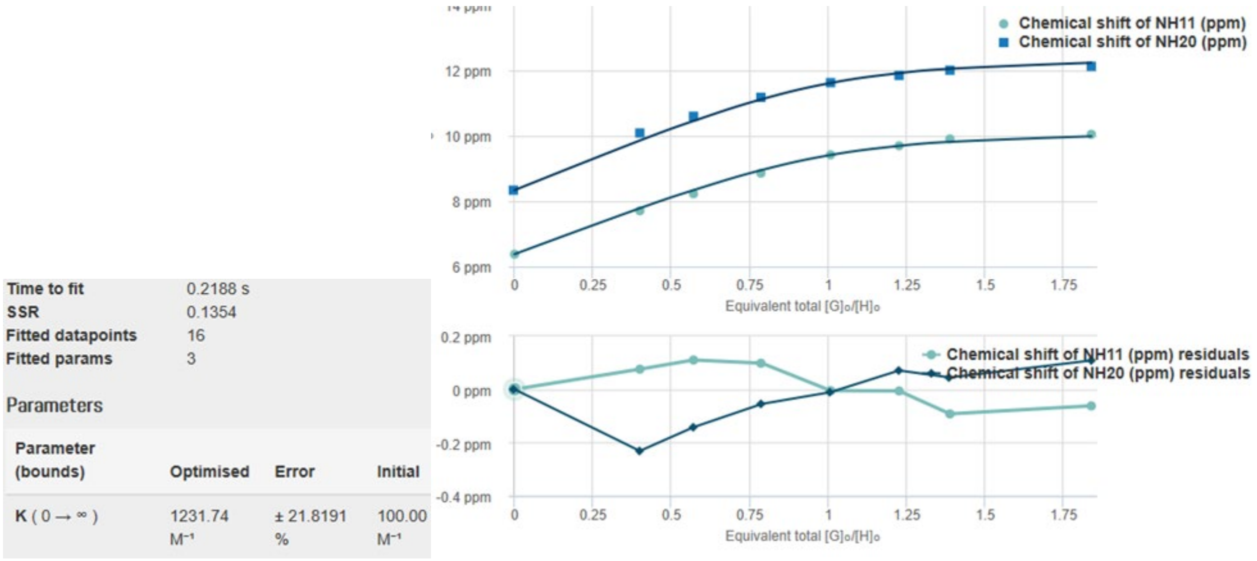

Figure S91: non-linear curve fitting analysis of the experimental titration data of **1** (14.2 mM in CD_2Cl_2) with increments of tetrabutylammonium acetate, recorded at 25 °C (400 MHz) using a theoretical binding isotherm for 1:1 binding http://app.supramolecular.org/bindfit/view/8c4696ee-d748-46d7-8bdf-e1f16d7c14d3

7. Remote fluorescence response

7.1. Compound **6**

7.1.1. Assignment of native directionality by ^{1}H NMR spectroscopy in CD_2Cl_2 at 25 °C

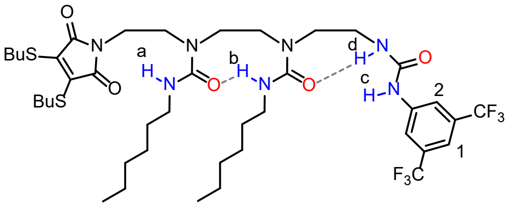

Compound **6** was dissolved in CD_2Cl_2 (11.7 mM at 25 °C), the assignments of NMR signals are done using ^{1}H COSY, 2D NOESY, HSQC, HMBC experiments and using the values of chemical shifts by analogy with previous parent compounds. The value of chemical shift of NH^a (5.96 ppm) is likely due to some weak H-bonding with the carbonyl of the fluorescent reporter. NMR data are consistent with the hydrogen bond directionality preference as drawn.

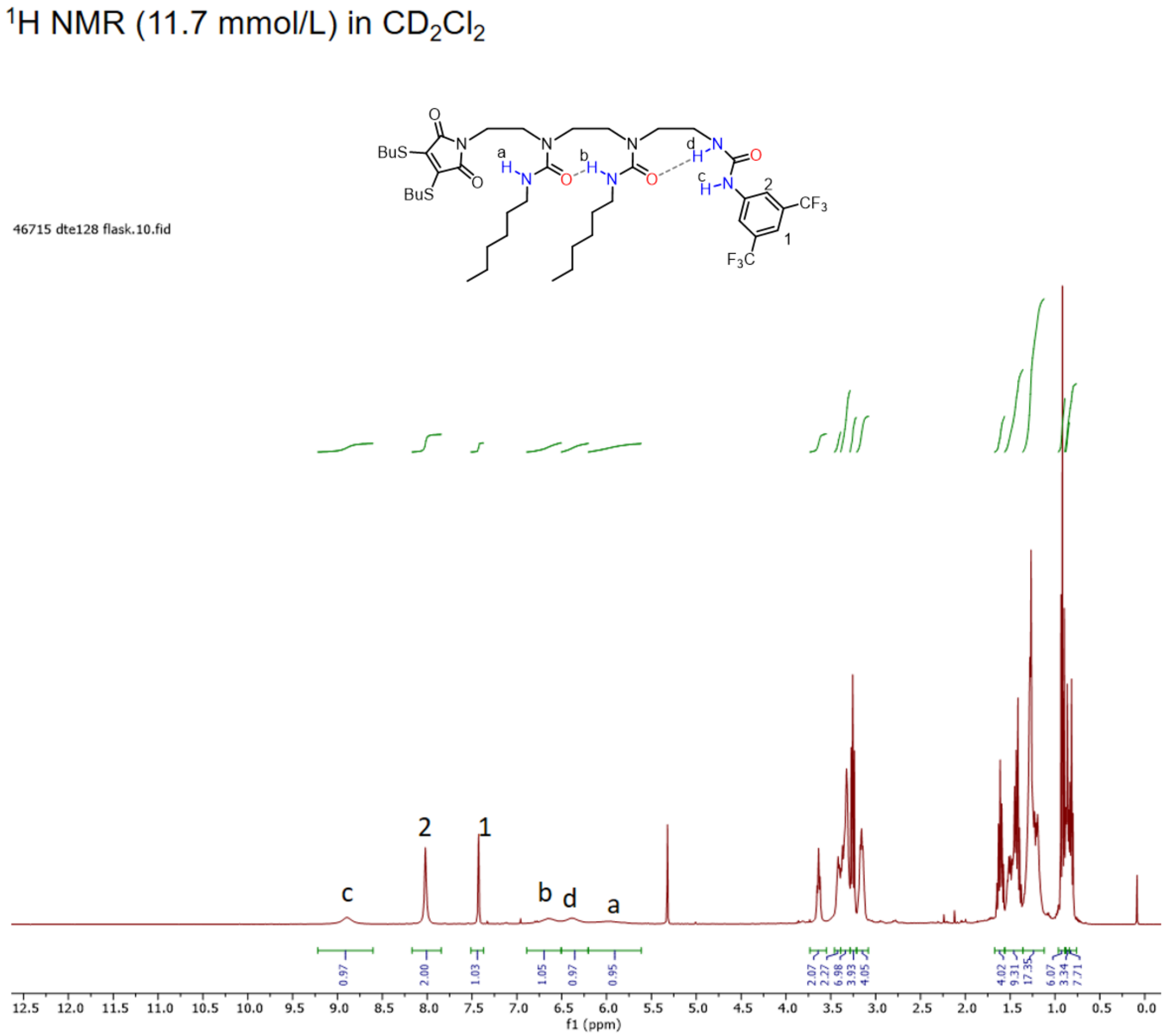

Figure S92: ^{1}H NMR spectrum of **6** in CD_2Cl_2 recorded at 25 °C (400 MHz).

	NH^a (ppm)	NH^b (ppm)	NH^c (ppm)	NH^d (ppm)
**6** (R^2 = fluorophore) 11.7 Mm in CD_2Cl_2	5.96	6.63	8.89	6.37
**3** (R^2 = Bn) 15 mM	5.25	6.44	8.97	6.58

Table S9. ^{1}H NMR chemical shift values of ureido N*H* of **6** and parent compound **3**

7.1.2. Titration with tetrabutylammonium diphenylphosphate in CD_2Cl_2 at 25 °C

- Monitoring of directionality change by ^{1}H NMR spectroscopy

Addition of 3.8 equivalents of tetrabutylammonium diphenylphosphate (as a solid) to a sample of **6** in CD_2Cl_2 (11.7 mM, 5 mg in 0.5 mL CD_2Cl_2) shifted the ureido NH signals of **6** (figure S93). The variations of the ^{1}H NMR chemical shift values of ureido NH signals are collected in Table S10. The observed downfield shifts for NH^c (CIS 1.56 ppm) and NH^d (CIS 1.47 ppm) are consistent with the formation of intermolecular hydrogen bonds between the ligand and the host at those positions. NH^b is involved in intramolecular hydrogen bonds no matter the global directionality of the hydrogen bond chain so no important variation of chemical shift value is expected for NH^b upon change of hydrogen bond chain directionality. The measured chemical induced shift for NH^b is 0 ppm. The ^{1}H NMR signal for NH^a moves downfield (CIS 0.72 ppm), it is consistent with a change of global directionality of the hydrogen bond chain, as NH^a forms a hydrogen bond with the carbonyl of the adjacent hexyl urea with better hydrogen bond acceptor properties than the carbonyl of the fluorescent probe. Overall, the data are consistent with the switch of global hydrogen bond directionality upon addition of ligand. The introduction of the fluorescent reporter at one terminus of the chain does not alter the conformational behaviour of the hydrogen bond chain when compared to **2**.

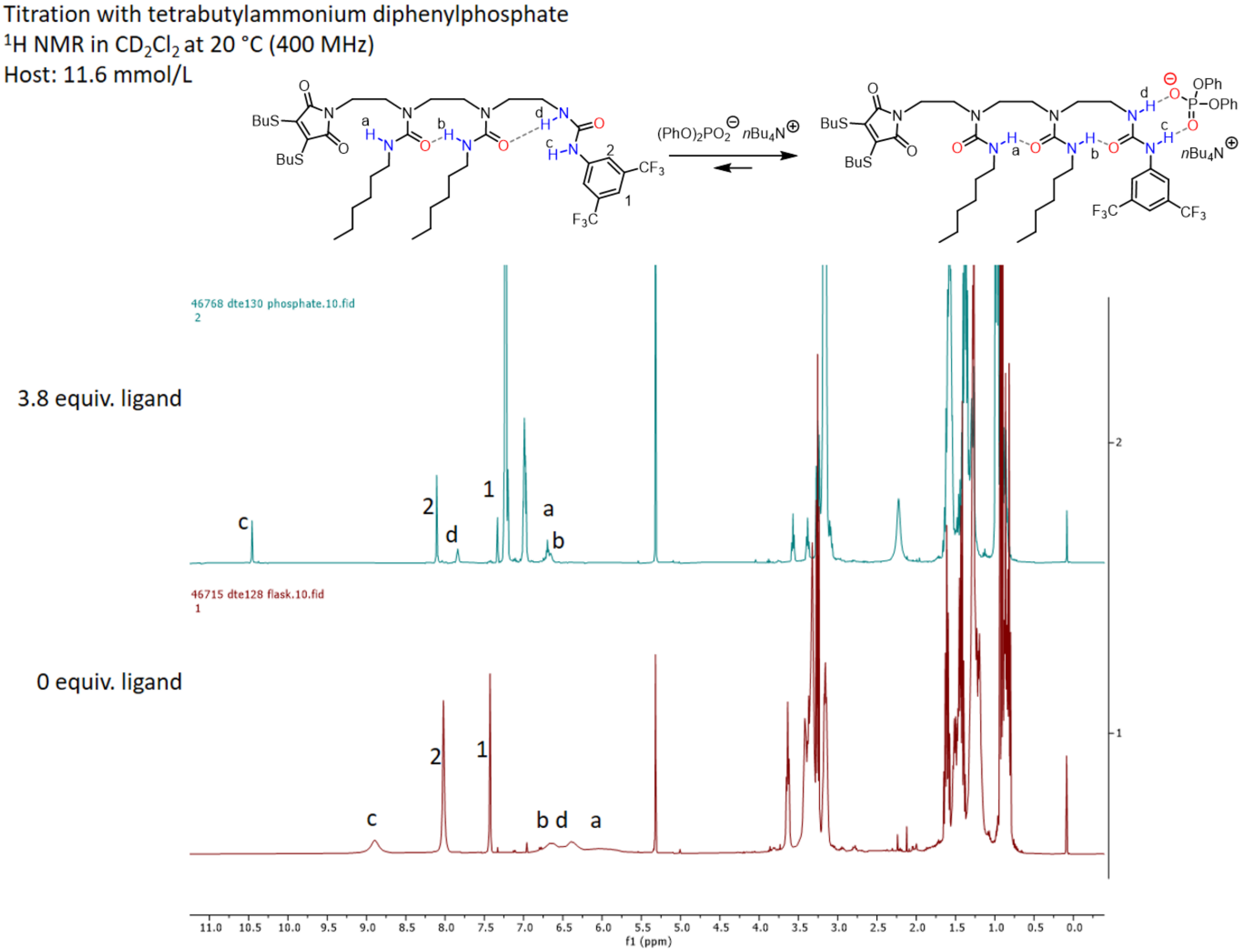

Figure S93: overlay of ^{1}H NMR spectra of **6** (11.6 mM in CD_2Cl_2) without and in the presence of tetrabutylammonium diphenylphosphate (3.8 equivalents) at 25 °C (400 MHz)

Equivalents of ligand	Chemical shift NH[a] (ppm)	Chemical shift NH[b] (ppm)	Chemical shift NH[c] (ppm)	Chemical shift NH[d] (ppm)	Wavelength of maximum emission (nm)
0	5.97	6.63	8.89	6.37	526
3.8	6.69	6.63	10.45	7.84	521
**CIS (ppm)**	**0.72**	**0**	**1.56**	**1.47**	

Table S10. chemical shifts of ^{1}H NH signals of **6** (11.6 mM in CD_2Cl_2) without and in the presence of tetrabutylammonium diphenylphosphate (3.8 equivalents) at 25 °C (400 MHz) and wavelength of maximum fluorescence emission (nm)

- **Fluorescence study**

To a solution of **6** in CH_2Cl_2 (1 mM) at 25 °C was added tetrabutylammonium diphenylphosphate (0 to 3 equivalents) and fluorescence spectra were recorded using a PerkinElmer LS45 fluorimeter after each addition of tetrabutylammonium diphenylphosphate (excitation at 405 nm). The wavelength corresponding to the maximum fluorescence emission shifts from 529 nm (no ligand) to 522 nm (with 1 equivalent of tetrabutylammonium diphenylphosphate). Addition of more ligand did not change the wavelength of maximum fluorescence emission. Increase in the relative emission signal upon addition of ligand is also observed.

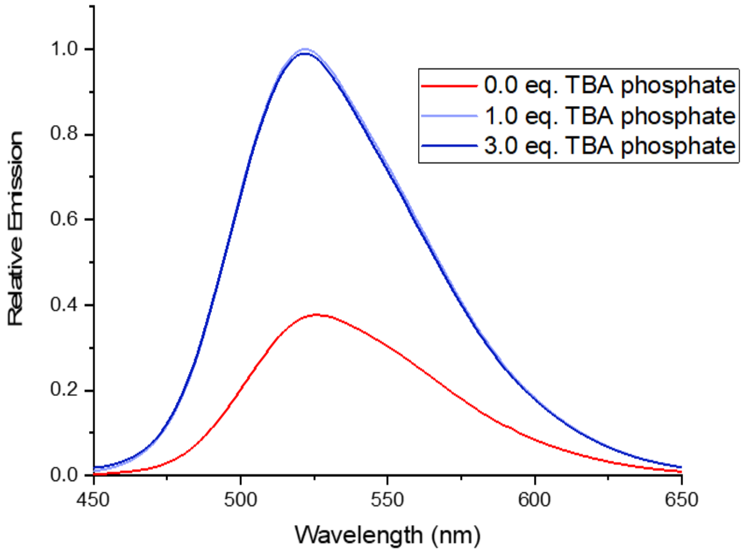

Figure S94: variation of fluorescence emission spectra of compound **6** in CH_2Cl_2 (1 mM) at 25 °C upon addition of increments of tetrabutylammonium diphenylphosphate (excitation at 405 nm)

7.1.3. Titration with tetrabutylammonium acetate in CD_2Cl_2 at 25 °C

- Monitoring of directionality change by ^{1}H NMR spectroscopy

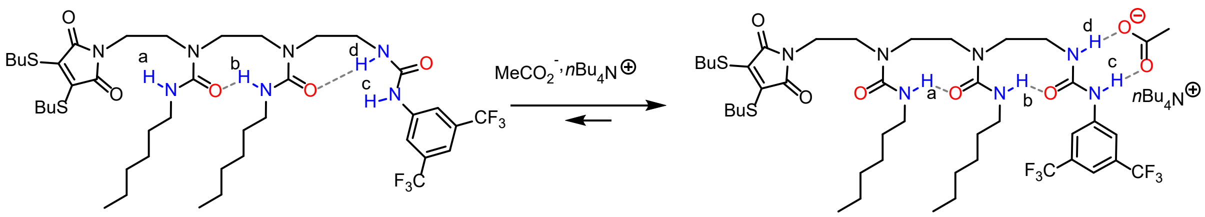

Addition of 2 equivalents of tetrabutylammonium acetate to a sample of **6** (5 mg, 5.8 mmol) dissolved in 0.5 mL CD_2Cl_2 shifted the ureido NH signals of **6** (figure S95), the variations in the chemical shift values of NH signals are collected in Table SS11. The observed downfield shifts for NH[c] (CIS 3.06 ppm) and NH[d] (CIS 3.44 ppm) are consistent with the formation of intermolecular hydrogen bonds between the ligand and the host at those NH positions. NH[b] is involved in intramolecular

hydrogen bonds no matter the directionality of the hydrogen bond chain so no important variation of chemical shift value is expected for NH^b upon change of global hydrogen bond directionality. The measured chemical induced shift for NH^b is 0.1 ppm. The signal for NH^a moves downfield (CIS 0.69 ppm), which is consistent with a change of global directionality of the hydrogen bond chain. NH^a forms a hydrogen bond with the carbonyl of the adjacent hexyl urea with better hydrogen bond acceptor properties than the carbonyl of the fluorescent probe. Overall, the data are consistent with the switch of hydrogen bond directionality upon addition of ligand.

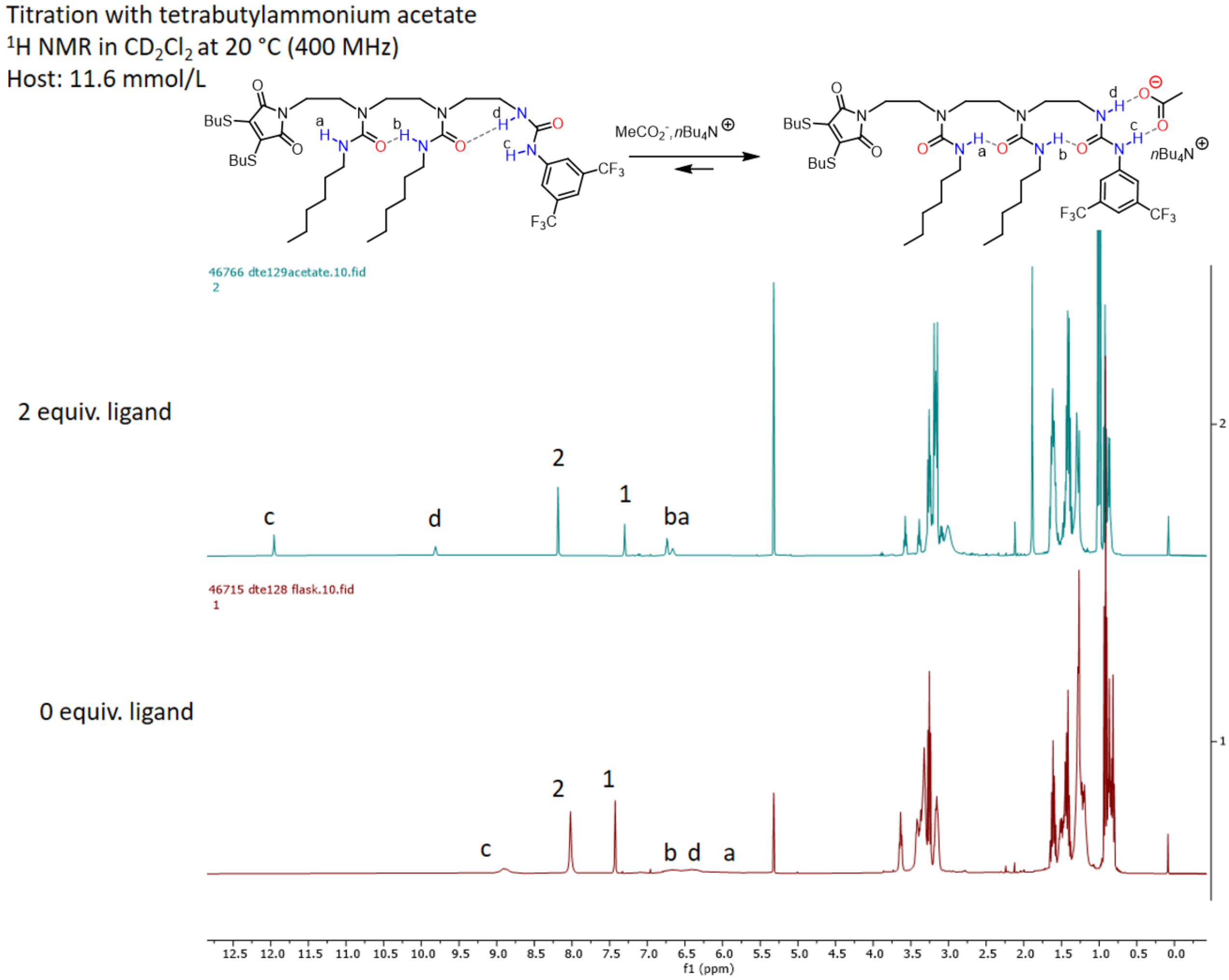

Figure S95: overlay of ^{1}H NMR spectra of **6** (11.6 mM in CD_2Cl_2) without and in the presence of tetrabutylammonium acetate (2 equivalents) at 25 °C (400 MHz)

Equivalents of ligand added	Chemical shift NH^a (ppm)	Chemical shift NH^b (ppm)	Chemical shift NH^c (ppm)	Chemical shift NH^d (ppm)	Wavelength of maximum emission (nm)
0	5.97	6.63	8.89	6.37	527
2	6.66	6.73	11.95	9.81	521
**CIS (ppm)**	**0.69**	**0.1**	**3.06**	**3.44**	

Table SS11. chemical shifts of ^{1}H NH signals of **6** (11.6 mM in CD_2Cl_2) without and in the presence of tetrabutylammonium acetate (2 equivalents) at 25 °C (400 MHz) and wavelength of maximum fluorescence emission (nm)

- Fluorescence study

To a solution of 6 (800 µmol/L) in CH_2Cl_2 at 25 °C was added tetrabutylammonium acetate (0 to 2 equivalents) and fluorescence spectra were recorded using a PerkinElmer LS45 fluorimeter after each addition of tetrabutylammonium acetate (excitation at 405 nm). The wavelength corresponding to the maximum fluorescence emission shifted from 529 nm (no ligand) to 522 nm (with 1 equivalent of tetrabutylammonium acetate). Addition of more ligand did not change the wavelength of maximum fluorescence emission. The emission signal increased upon addition of tetrabutylammonium acetate.

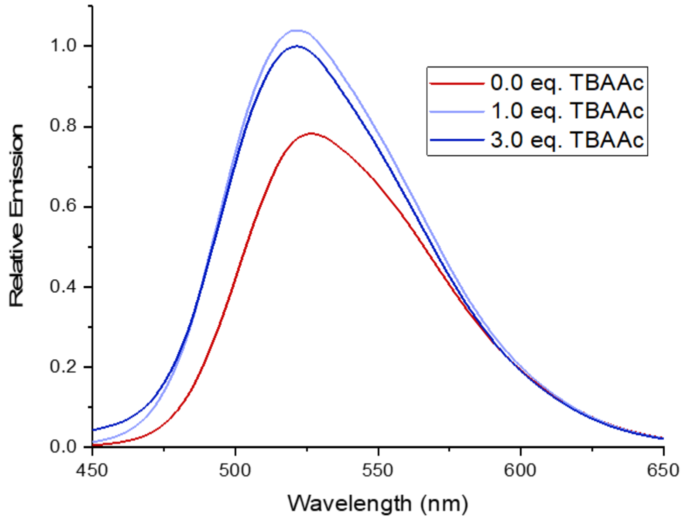

Figure S96: variation of fluorescence emission spectra of compound **6** (800 µmol/L) in CH_2Cl_2 at 25 °C upon addition of increments of tetrabutylammonium acetate (excitation at 405 nm)

7.1.4. Titration with tetrabutylammonium chloride in CD_2Cl_2 at 25 °C

- Monitoring of directionality change by ^{1}H NMR spectroscopy

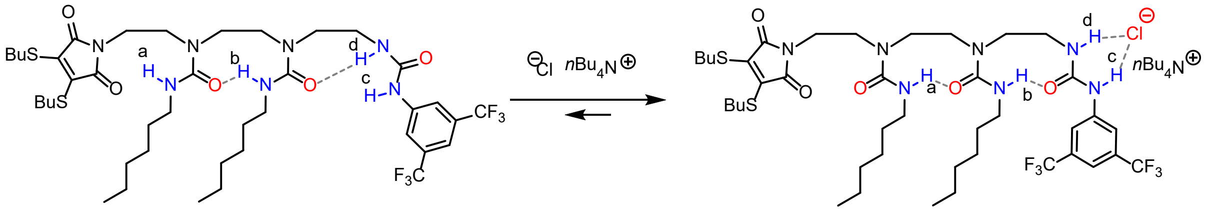

Addition of 2.58 equivalents of tetrabutylammonium chloride to a sample of 6 (5 mg, 5.8 mmol) dissolved in 0.5 mL CD_2Cl_2 shifted the ureido NH signals of **6** (figure S97), the variations in the chemical shift values of NH signals are collected in Table SS12. The observed downfield shifts for NH^c (CIS 1.69 ppm) and NH^d (CIS 1.32 ppm) are consistent with the formation of intermolecular hydrogen bonds between the ligand and the host at those NH positions. NH^b is involved in intramolecular hydrogen bonds no matter the directionality of the hydrogen bond chain so no important variation of chemical shift value is expected for NH^b upon change of global hydrogen bond directionality. The measured upfield chemical induced shift for NH^b is 0.1 ppm. The signal for NH^a moves downfield (CIS 0.52 ppm), it is consistent with a change of global directionality of the hydrogen bond chain. NH^a forms a hydrogen bond with the carbonyl of the adjacent hexyl urea with better hydrogen bond acceptor properties than the carbonyl of the fluorescent probe. Overall, the data are consistent with the switch of hydrogen bond directionality upon addition of ligand.

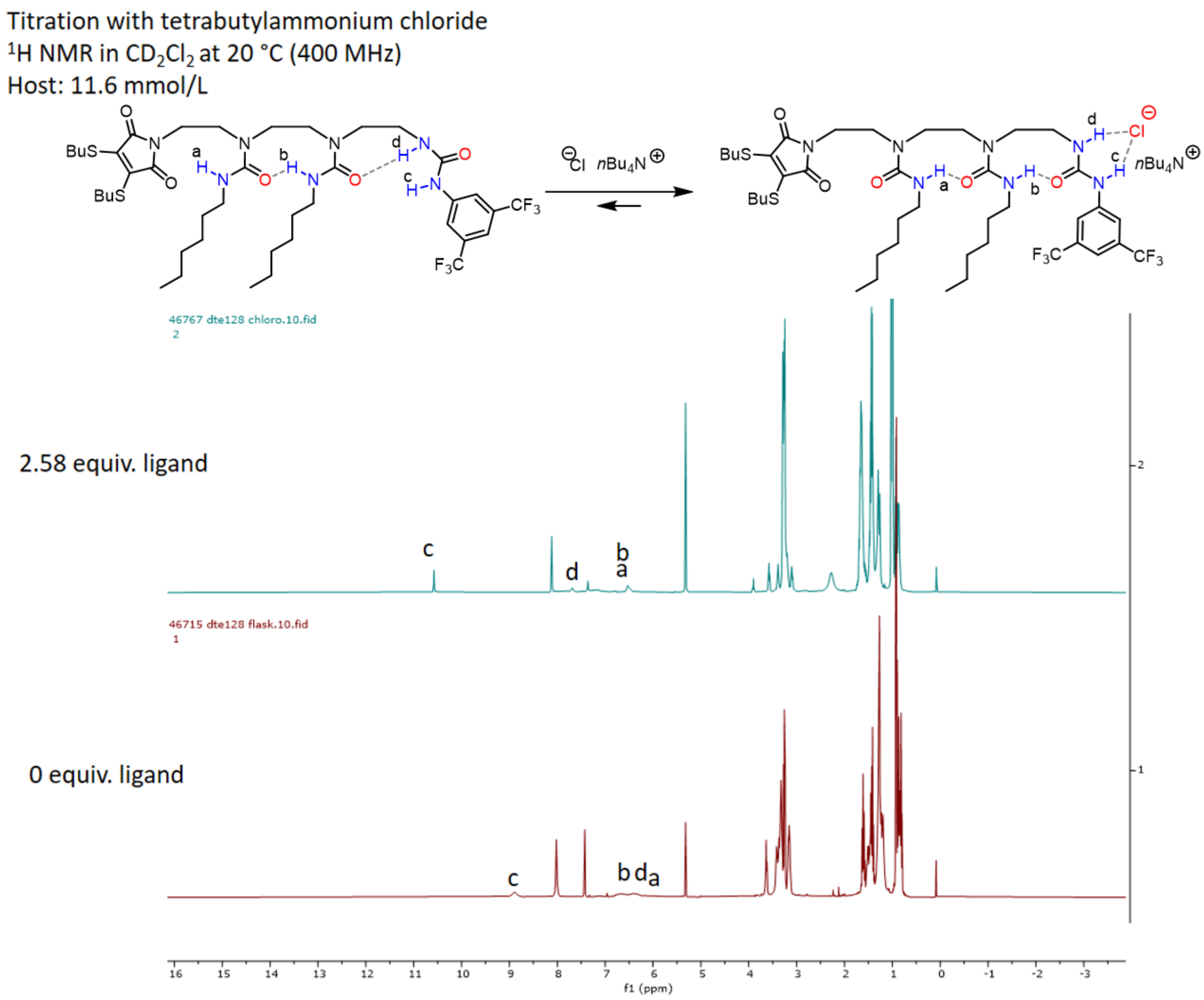

Figure S97: overlay of ^{1}H NMR spectra of **6** (11.6 mM in CD_2Cl_2) without and in the presence of tetrabutylammonium chloride (2.58 equivalents) at 25 °C (400 MHz)

Equivalents of ligand added	Chemical shift NH^a (ppm)	Chemical shift NH^b (ppm)	Chemical shift NH^c (ppm)	Chemical shift NH^d (ppm)	Wavelength of maximum emission (nm)
0	5.97	6.63	8.89	6.37	527
2.6	6.49	6.53	10.58	7.69	521
**CIS (ppm)**	**0.52**	**0.1**	**1.69**	**1.32**	

Table SS12. chemical shifts of ^{1}H NH signals of **6** 11.6 mM in CD_2Cl_2) without and in the presence of tetrabutylammonium chloride (2.6 equivalents) at 25 °C (400 MHz) and wwavelengths of maximum fluorescence emission (nm)

- **Fluorescence study:**

To a solution of **6** (1mM) in CH_2Cl_2 at 25 °C was added tetrabutylammonium chloride (0 to 3 equivalents), fluorescence emission spectra were recorded after each addition of tetrabutylammonium chloride (excitation at 405 nm). The wavelength corresponding to the maximum fluorescence emission shifted from 527 nm (no ligand) to 521 nm (with one equivalents of tetrabutylammonium chloride). Addition of more ligand did not change the wavelength of maximum fluorescence emission. Increase of the fluorescence emission signal was observed upon addition of ligand.

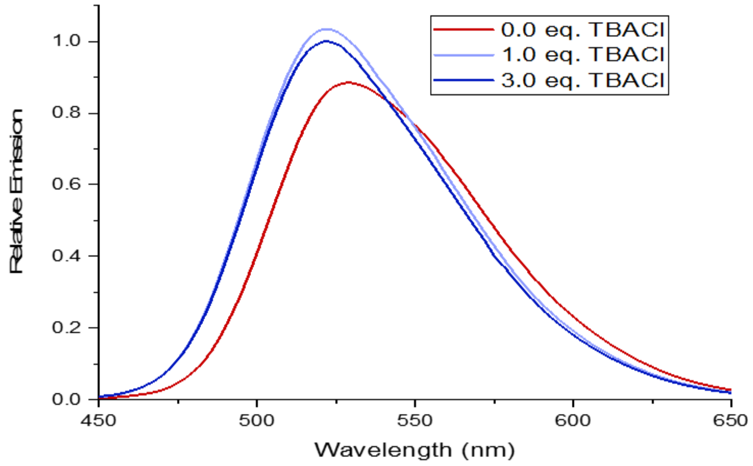

Figure S98: variation of fluorescence emission spectra of compound **6** (1 mM) in CH_2Cl_2 at 25 °C upon addition of increments of tetrabutylammonium chloride (excitation at 405 nm)

7.2. Compound 7

- Fluorescence study

To a solution of **7** (1 mM) in CH_2Cl_2 at 25 °C was added tetrabutylammonium acetate (0 to 2 equivalents) and fluorescence spectra were recorded after each addition of tetrabutylammonium acetate (excitation at 405 nm). The wavelength corresponding to the maximum fluorescence emission shifted from 526 nm (no ligand) to 521 nm with one equivalents of tetrabutylammonium acetate. Addition of more ligand did not change the wavelength of maximum fluorescence emission.

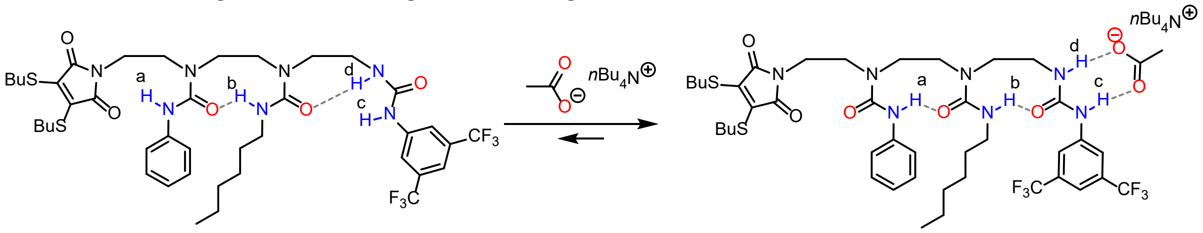

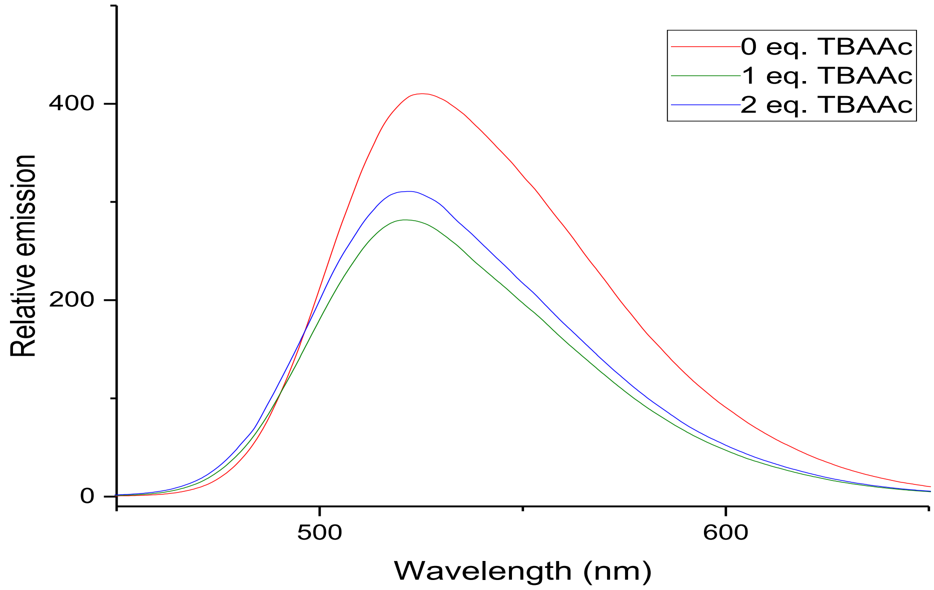

Figure S99: variation of fluorescence emission spectra of compound **7** (1mM) in CH_2Cl_2 at 25 °C upon addition of increments of tetrabutylammonium acetate (excitation at 405 nm)

7.3. Control experiments

7.3.1. Fluorescence study on compound 11 in the absence and presence of ligands

To a solution of **11** (1 mM) in CH_2Cl_2 at 25 °C was added tetrabutylammonium acetate (3 equivalents), or tetrabutylammonium chloride (3 equivalents), or tetrabutylammonium diphenylphosphate (3 equivalents) and fluorescence spectra were recorded (excitation at 405 nm). The wavelength corresponding to the maximum fluorescence emission remained constant before and after addition of ligands 529 nm.

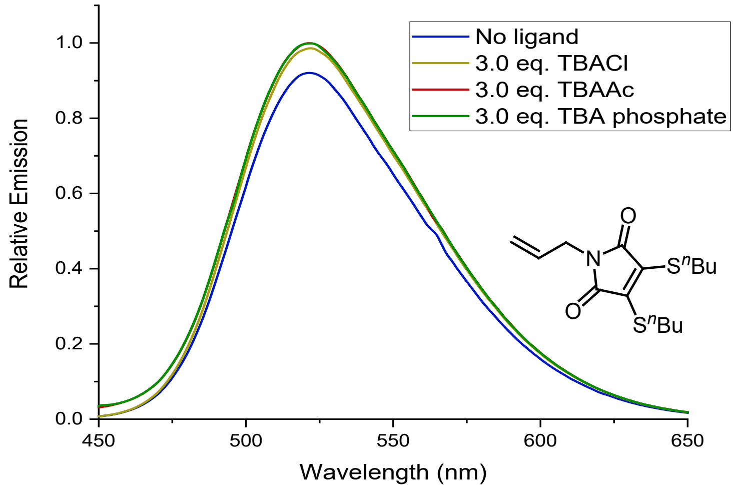

Figure S100: Fluorescence emission spectra of compound **11** in CH_2Cl_2 at 25 °C with no ligand and after addition of 3 equivalents of tetrabutylammonium diphenylphosphate, acetate or chloride (wavelength of maximum emission 529 nm).

7.3.2. Fluorescence study on compound 12 in the absence and presence of ligands

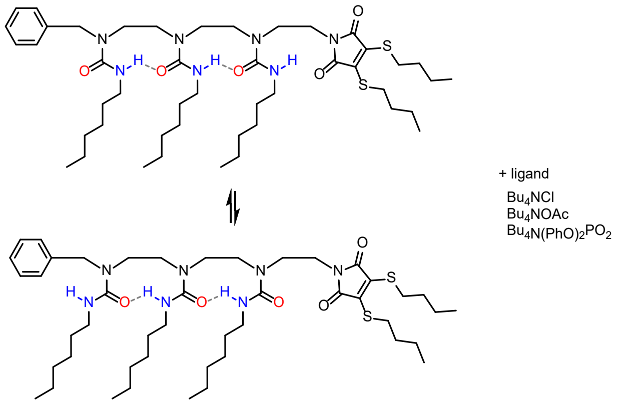

Fluorescence emission spectra of compound **12** (1 mM) in dry CH_2Cl_2 at 25 °C were recorded with no ligand and after addition of increments of tetrabutylammonium diphenylphosphate, or tetrabutylammonium acetate, or tetrabutylammonium chloride from 0 to 2 equivalents (excitation at 405 nm). The wavelengths corresponding to the maximum fluorescence emission were measured after each addition of ligand: minimal variation in the wavelength was observed upon addition of ligands to **12** (Table SS13).

Equiv ligand	Bu_4NOAc	Bu_4NCl	$Bu_4N(PhO)_2PO_2$
0	538	536	538.5
0.2	539	537.5	537.5
0.4	538	538	540
0.6	538	538.5	539
0.8	538	540	538.5
1	539	538	538.5
1.5	537.5	537.5	539
2	537	538	539

Table S13. Wavelength of maximum emission signal of **12** (1 mM in dry dichloromethane) during the fluorescence titration (excitation at 405 nm at 25 °C) with either tetrabutylammonium diphenylphosphate, acetate, or chloride from 0 to 2 equivalents.

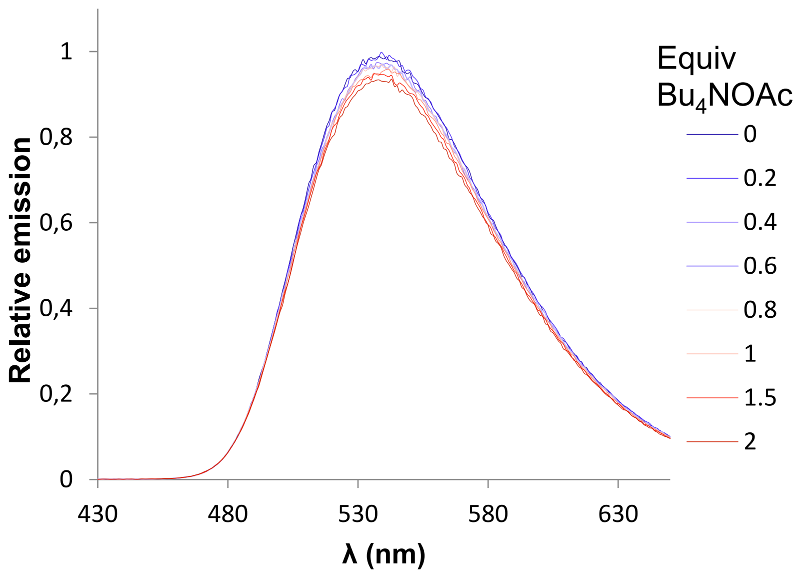

Figure S101: variation in fluorescence emission spectra of compound **12** (1 mM) in CH_2Cl_2 at 25 °C upon addition of increments of tetrabutylammonium acetate

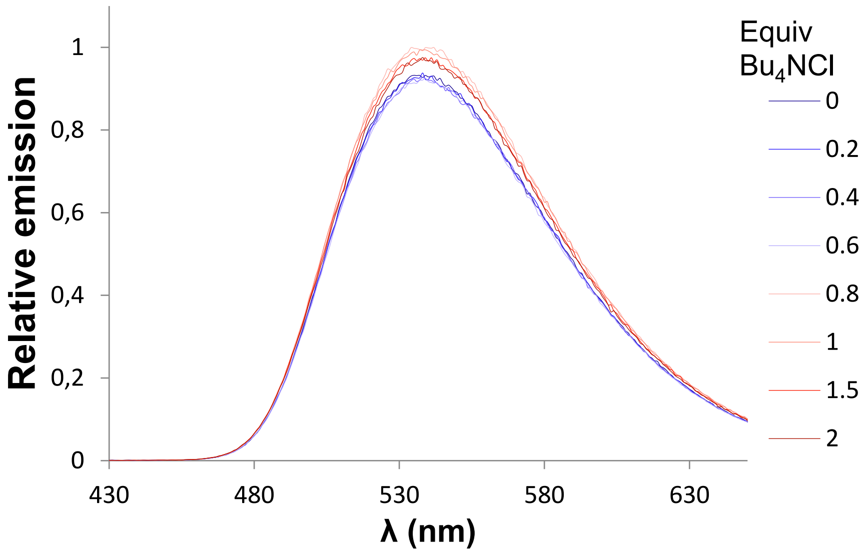

Figure S102: variation in fluorescence emission spectra of compound **12** (1 mM) in CH_2Cl_2 at 25 °C upon addition of increments of tetrabutylammonium chloride

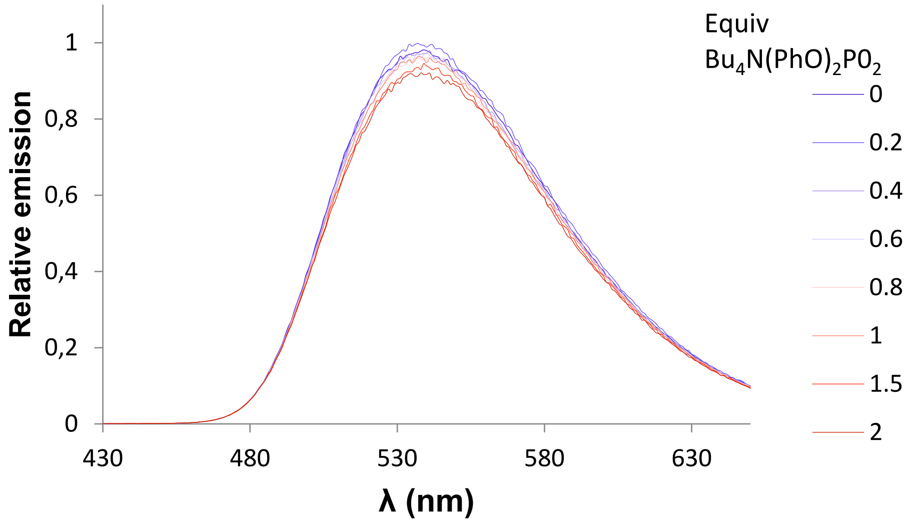

Figure S103: variation in fluorescence emission spectra of compound **12** (1 mM) in CH_2Cl_2 at 25 °C upon addition of increments of tetrabutylammonium diphenylphosphate

Fluorescence excitation spectra of compound **12** (1 mM) in dry CH_2Cl_2 at 25 °C were recorded with no ligand and after addition of increments of tetrabutylammonium diphenylphosphate, or tetrabutylammonium acetate, or tetrabutylammonium chloride from 0 to 2 equivalents (emission 530 nm). The wavelengths corresponding to the maximum fluorescence excitations were measured after each addition of ligand: minimal variation in the wavelength was observed upon addition of ligands to **12**.

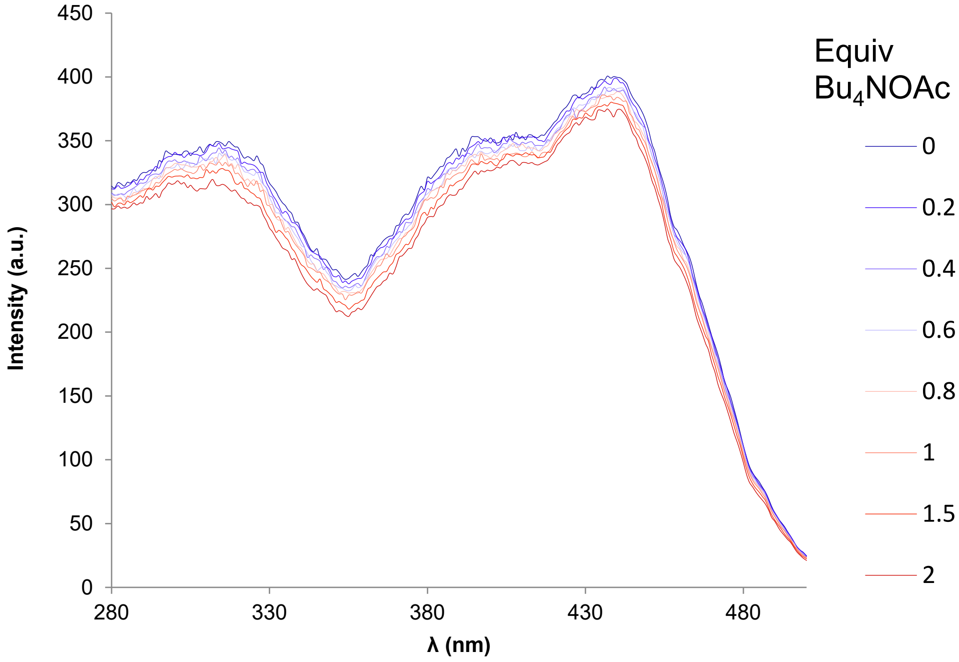

Figure S104: variation in fluorescence excitation spectra of compound **12** (1 mM) in CH_2Cl_2 at 25 °C upon addition of increments of tetrabutylammonium acetate

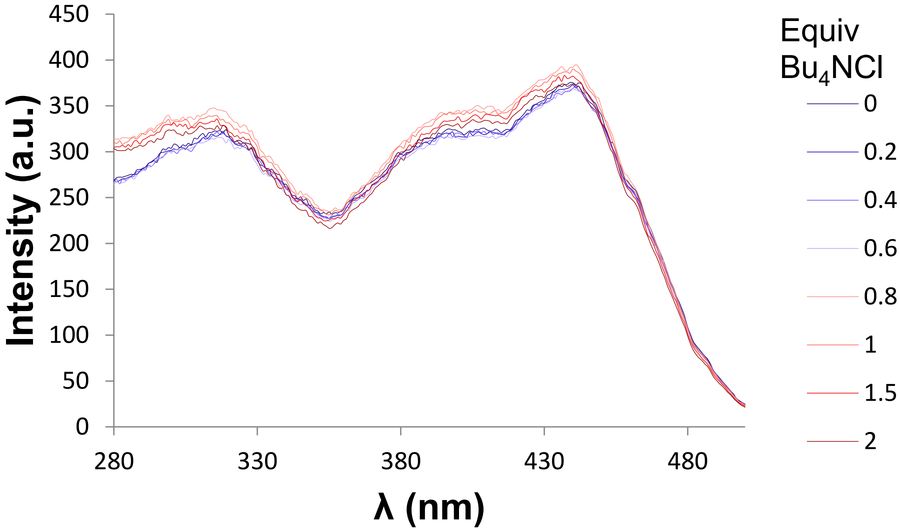

Figure S105: variation in fluorescence excitation spectra of compound **12** (1 mM) in CH_2Cl_2 at 25 °C upon addition of increments of tetrabutylammonium chloride

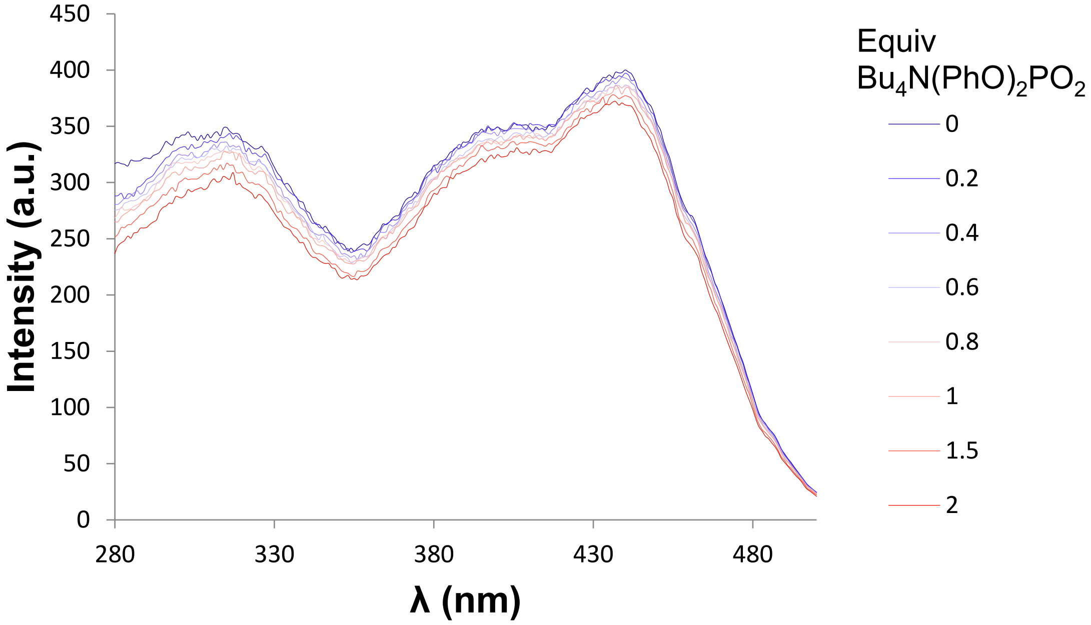

Figure S106: variation in fluorescence excitation spectra of compound **12** (1 mM) in CH_2Cl_2 at 25 °C upon addition of increments of tetrabutylammonium diphenylphosphate

7.4. Compound 8

7.4.1. ^{1}H NMR study in CD_2Cl_2 at 25 °C

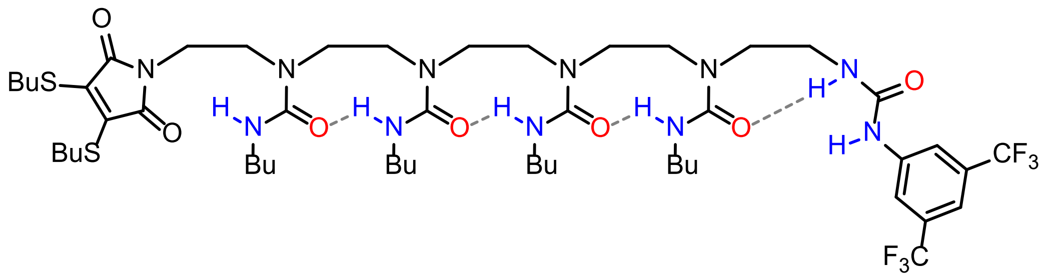

The ^{1}H NMR chemical shift values for the ureido NH of **8** in $CDCl_3$ at 25 °C are similar to the one measured for shorter analogues **5** and **6** in the same conditions: the ^{1}H NMR data are consistent with a directionality of the hydrogen bond chain identical to the shorter analogues. nOe cross peaks were not observed due to a broadening of the NH signals in **8**, likely due to the flexibility of the alkyl chains. The wavelength of maximum fluorescence emission of **8** in CH_2Cl_2 (1 mM at 25 °C) is 546 nm (another fluorimeter was used to record the spectra for this sample and a slightly different calibration of the apparatus leads to wavelength variations ranging from 546 nm to 534 nm during these titrations instead of the previous range from 530 to 521 nm measured in the titrations above).

The titration of **8** with increments of ligands (tetrabutylammonium acetate, chloride or diphenylphosphate) was initially monitored by ^{1}H NMR, the spectra recorded after each addition of ligands were not informative due to the broadness of the NH peaks.

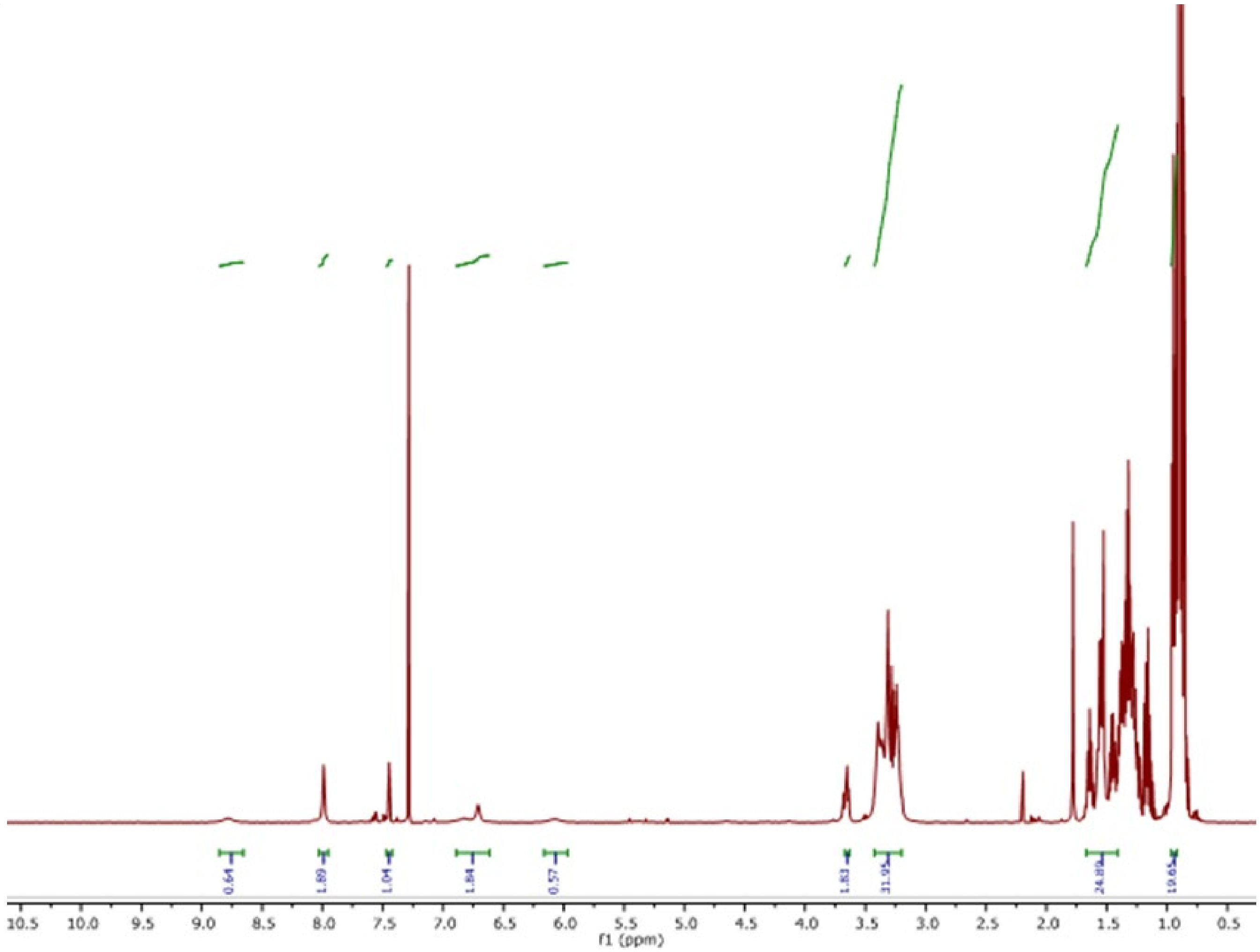

Figure S107: ^{1}H NMR spectra of **8** in $CDCl_3$) at 25 °C (500 MHz)

7.4.2. Fluorescence study in the presence of ligands

To a solution of **8** (1mM) in CH_2Cl_2 at 25 °C was added tetrabutylammonium acetate, chloride or diphenylphosphate and fluorescence spectra were recorded after each addition (excitation at 405 nm, emission 530 nm, figures S108-s114).

The wavelength corresponding to the maximum fluorescence emission shifted from 546 nm (no ligand) to 535 nm with 1 equivalent of ligands, this shift was concomitant with a hyperchromic shift of the emission signal. Addition of more than 1 equivalent of ligand ligand did not alter the wavelength of maximum fluorescence emission (Table S14) yet caused a slight hypochromic shift for tetrabutylammonium acetate and chloride. Progressive addition of tetrabutylammonium diphenylphosphate to **8** from 0 to 1 equivalent led to stronger hyperchromic effects while adding more than one equivalent of that ligand led to a more rapid hypochromic effect on the signal (Figure S113).

The excitation fluorescence spectra recorded during the titration of **8** with tetrabutylammonium acetate show a gradual blue-shift of the S0-S1 excitation maximum from 444 nm at 0 equivalent of ligand to 431 nm after 1 equivalent of acetate was added (Figure S110). Minimal spectral changes were observed upon addition of more than 1 equivalent of acetate as only a slight hypochromic effect was observed. Similarly, the excitation fluorescence spectra recorded during the titration of **8** with tetrabutylammonium chloride show a gradual blue-shift of the S0-S1 excitation maximum from 441 nm at 0 equivalent of ligand to 433 nm after 1 equivalent of chloride was added then minimal spectral changes were observed upon addition of more than 1 equivalent, a slight hypochromic effect was observed on the fluorescence signal during the entire titration (Figure S112). When tetrabutylammonium diphenylphosphate was used as the titrating ligand, the excitation fluorescence spectra show a gradual blue-shift of the S0-S1 excitation maximum from 444 nm at 0 equivalent of ligand to 429 nm after 1 equivalent of diphenylphosphate along with a hyperchromic effect on the fluorescence signal, then a rapid and strong hypochromic effect was observed on the fluorescence signal upon addition of more ligand (Figure S114).

	Wavelength of maximum emission (nm)		
Equiv ligand	Bu_4NOAc	Bu_4NCl	$Bu_4N(PhO)_2PO_2$
0	545.5	546	546
0.125	544	544	545
0.25	544.5	543.5	544.5
0.375	540.5	543.7	543
0.5	540.5	540	540.5
0.625	538	540	539
0.75	537	537	536
0.875	535	537	535.5
1	535.5	535.5	535.5
1.25		534	535.5
1.375	534.5		
1.5			534
1.75	534	534	534
2		535	
2.5			535
3	534.5		

Table S14. Wavelength of maximum fluorescence emission measured during the titration of **8** (1 mM in dichloromethane at 25 °C) with increments of tetrabutylammonium acetate, chloride or diphenylphosphate

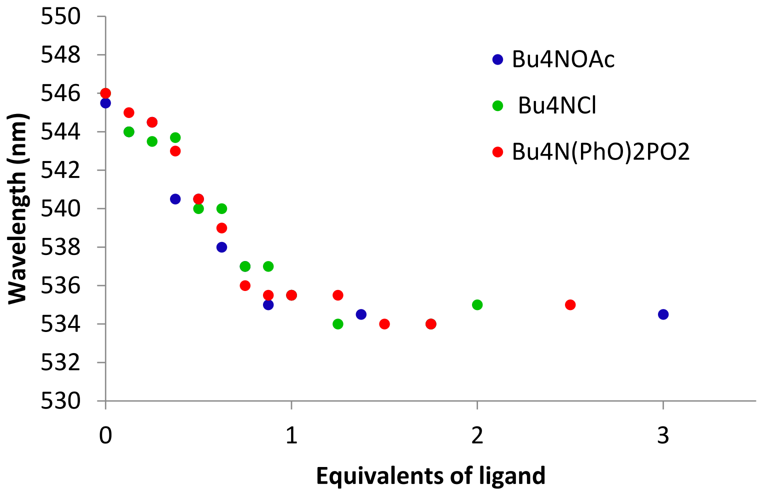

Figure S108. Wavelength of maximum fluorescence emission measured during the titration of **8** (1 mM in dichloromethane at 25 °C) as a function of increments of tetrabutylammonium acetate, chloride or diphenylphosphate

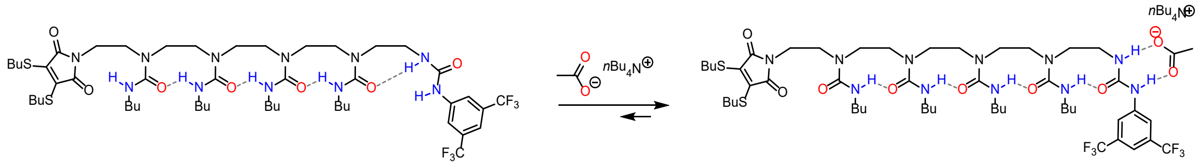

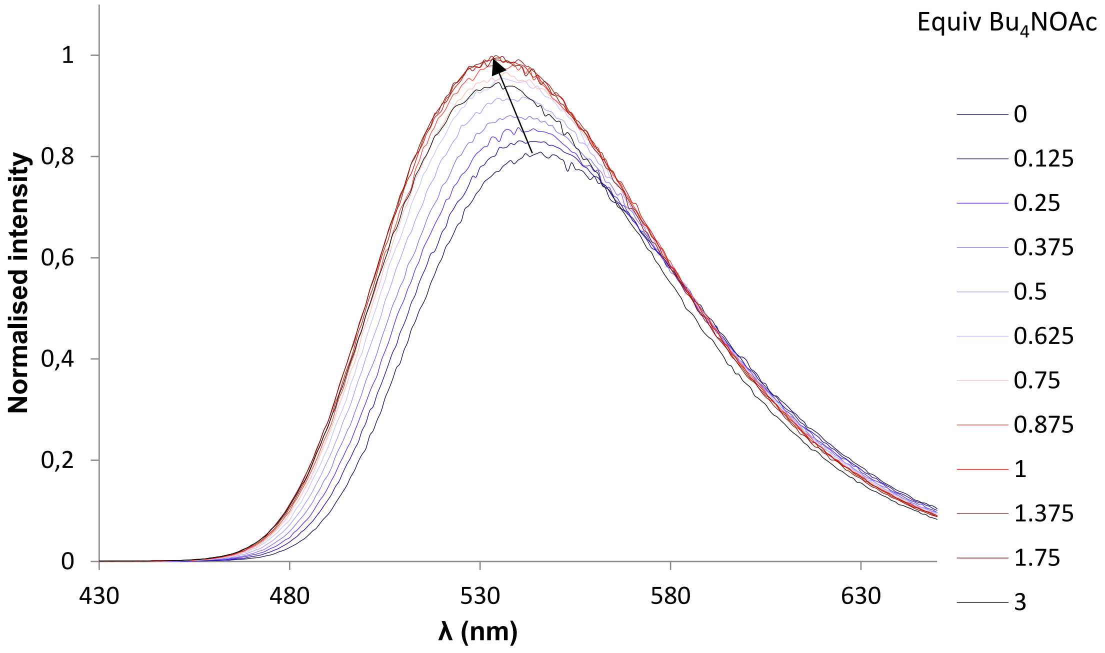

Figure S109: Fluorescence emission spectra of compound **8** in CH_2Cl_2 (1 mM) at 25 °C with no ligand and after addition of increments of tetrabutylammonium acetate from 0 to 3 equivalents (excitation at 405 nm, 20 °C).

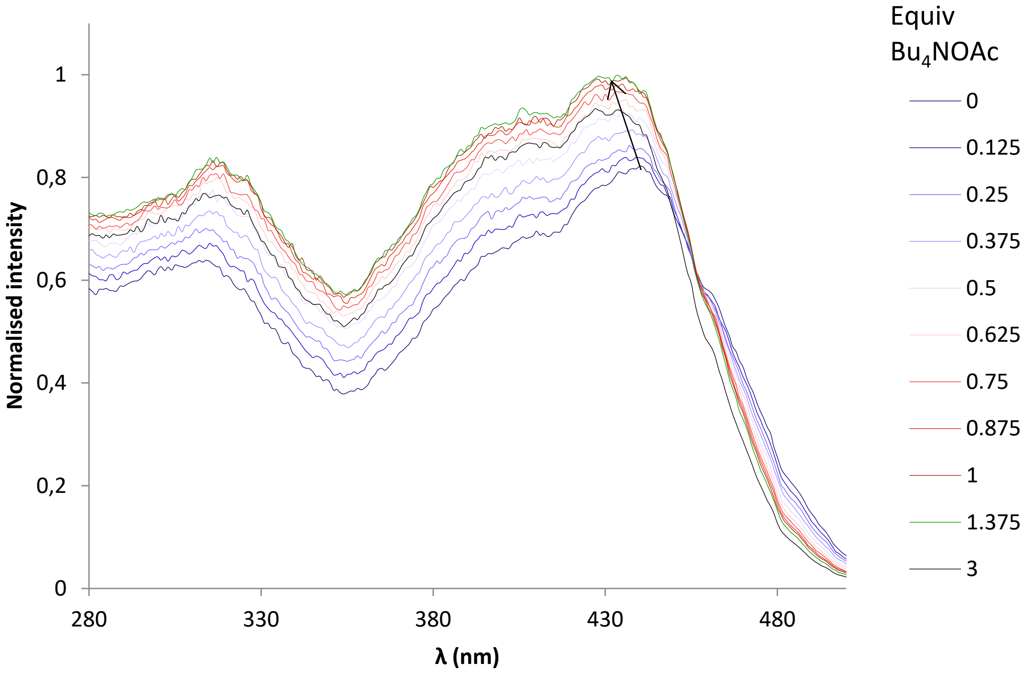

Figure S110: Fluorescence excitation spectra of compound **8** in CH_2Cl_2 (1 mM) at 25 °C with no ligand and after addition of increments of tetrabutylammonium acetate from 0 to 3 equivalents (emission at 530 nm, 20 °C). The intensity of signal increases upon addition of up to one equivalent of ligand with a shift of the wavelength of maximum fluorescence emission from 446 nm to 432 nm, addition of further amount of ligand leads to a decrease in the fluorescence emission signal intensity.

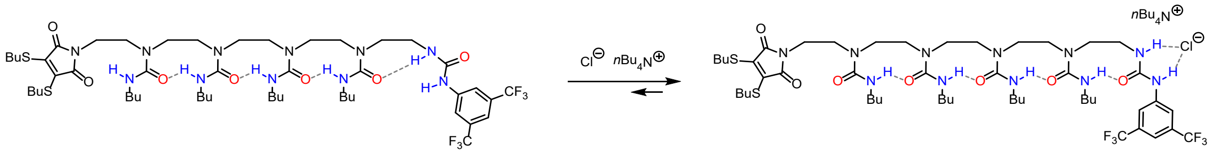

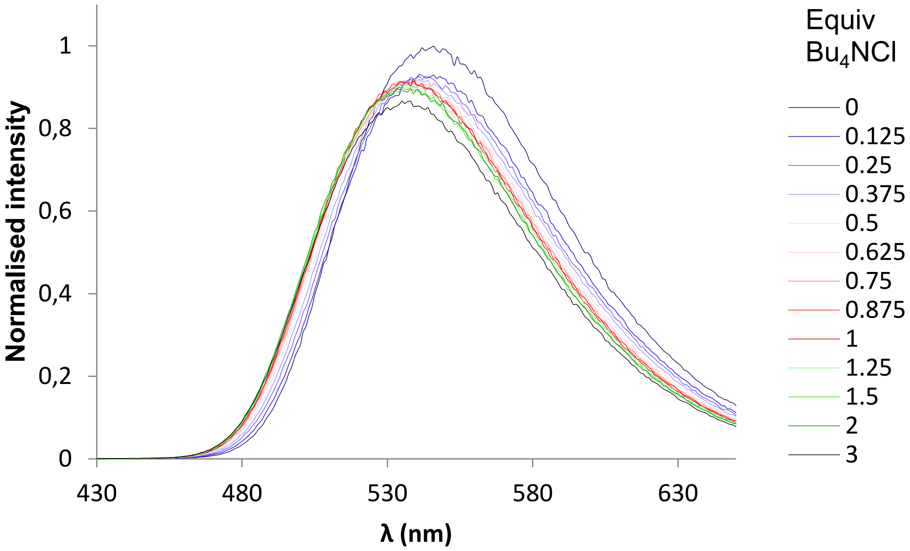

Figure S111: Fluorescence emission spectra of compound **8** in CH_2Cl_2 (1 mM) at 25 °C with no ligand and after addition of increments of tetrabutylammonium chloride from 0 to 3 equivalents (excitation at 405 nm, 20 °C).

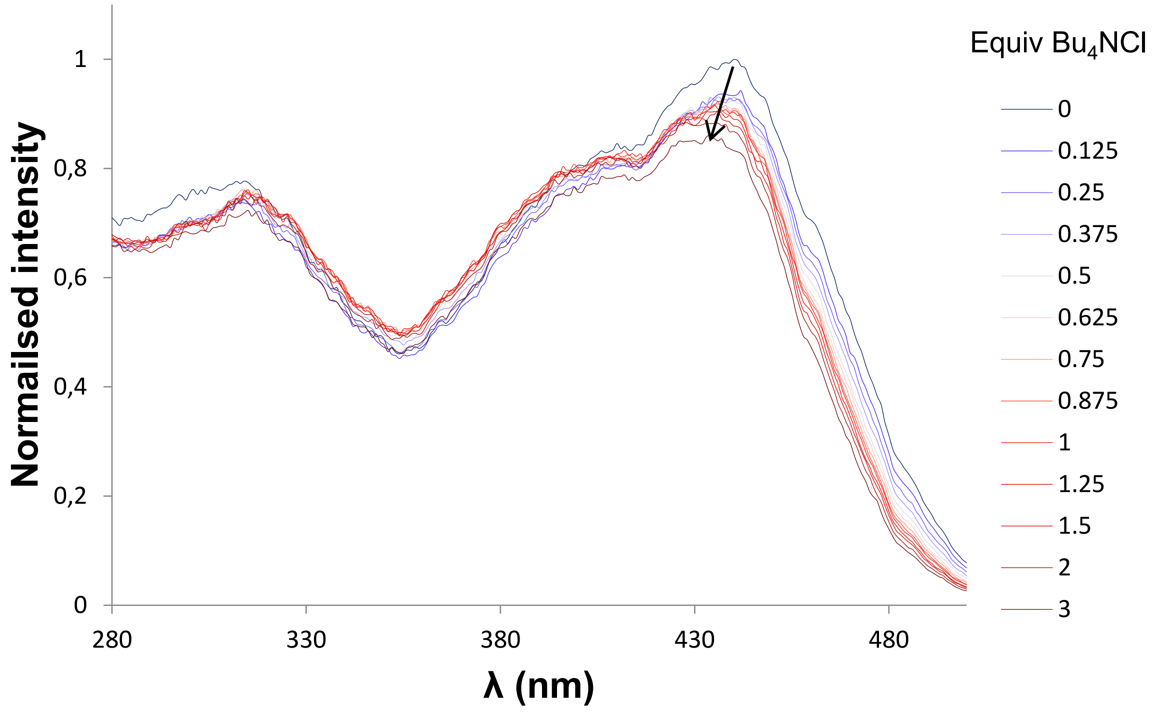

Figure S112: Fluorescence excitation spectra of compound **8** in CH_2Cl_2 (1 mM) at 25 °C with no ligand and after addition of increments of tetrabutylammonium chloride from 0 to 3 equivalents (emission at 530 nm, 20 °C).

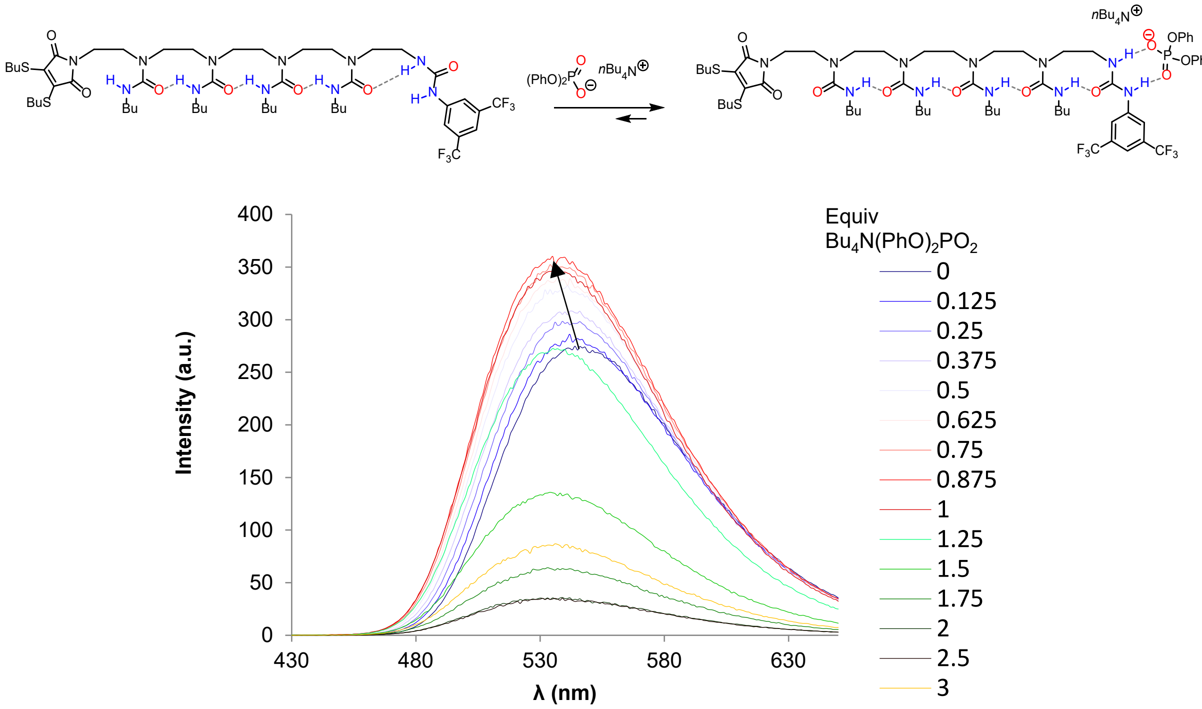

Figure S113: Fluorescence emission spectra of compound **8** in CH_2Cl_2 (1 mM) at 25 °C with no ligand and after addition of increments of tetrabutylammonium diphenylphosphate from 0 to 3 equivalents (excitation at 405 nm, 20 °C). The intensity of signal increases upon addition of up to one equivalent of ligand with a shift of the wavelength of maximum fluorescence emission from 546 nm to 535 nm, addition of further amount of ligand leads to a decrease in the fluorescence emission signal intensity.

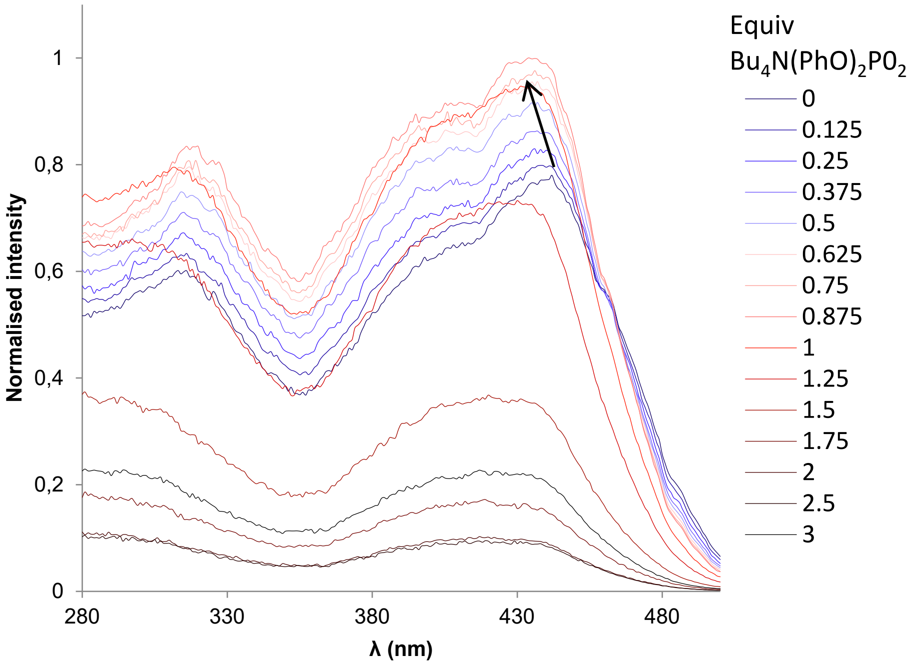

Figure S114: Fluorescence excitation spectra of compound **8** in CH_2Cl_2 (1 mM) at 25 °C with no ligand and after addition of increments of tetrabutylammonium diphenylphosphate from 0 to 3 equivalents (emission at 530 nm, 20 °C). The intensity of signal increases upon addition of up to one equivalent of ligand with a shift of the wavelength of maximum fluorescence emission from 446 nm to 431 nm, addition of further amount of ligand leads to a decrease in the fluorescence emission signal intensity.

---

[1] D. P. Tilly, J. P. Heeb, S. J. Webb, J. Clayden, *Nature Commun.* **2023**, *14*, 2647.

[2] D. P. Tilly, M. Žabka, I. Vitorica-Yrezabal, H. A. Sparkes, N. Pridmore, J. Clayden, *Chem. Sci.,* **2022**, *13*, 13153-13159.

[3] D. T. J. Morris, S. M. Wales, D. P. Tilly, E. H. E. Farrar, M. N. Grayson, J. W. Ward, J. Clayden, *Chem,* **2021**, *7*, 2460-2472.

[4] J. C. Chang, S. H. Tseng, C. C. Lai, Y. H. Liu, S. M. Peng, S. H. Chiu, *Nature chem*., **2017**, *9(2)*, 128-134.
